# Blood virosphere in febrile Tanzanian children

**DOI:** 10.1080/22221751.2021.1925161

**Published:** 2021-05-28

**Authors:** Samuel Cordey, Florian Laubscher, Mary-Anne Hartley, Thomas Junier, Kristina Keitel, Mylène Docquier, Nicolas Guex, Christian Iseli, Gael Vieille, Philippe Le Mercier, Anne Gleizes, Josephine Samaka, Tarsis Mlaganile, Frank Kagoro, John Masimba, Zamzam Said, Hosiana Temba, Gasser H. Elbanna, Caroline Tapparel, Marie-Celine Zanella, Ioannis Xenarios, Jacques Fellay, Valérie D’Acremont, Laurent Kaiser

**Affiliations:** a Division of Infectious Diseases, Geneva University Hospitals, Geneva, Switzerland; b Laboratory of Virology, Division of Infectious Diseases and Division of Laboratory Medicine, University Hospitals of Geneva & Faculty of Medicine, University of Geneva, Geneva, Switzerland; c Centre for Primary Care and Public Health (Unisanté), University of Lausanne, Lausanne, Switzerland; d Intelligent Global Health, Machine Learning and Optimization Laboratory, EPFL, Lausanne, Switzerland; e Global Health Institute, School of Life Sciences, EPFL, Lausanne, Switzerland; f SIB Swiss Institute of Bioinformatics, Lausanne, Switzerland; g Swiss Tropical and Public Health Institute, University of Basel, Basel, Switzerland; h Department of Paediatric Emergency Medicine, Department of Pediatrics, Inselspital, Bern University Hospital, University of Bern, Bern, Switzerland; i iGE3 Genomics Platform, University of Geneva, Geneva, Switzerland; j Department of Genetics and Evolution, University of Geneva, Geneva, Switzerland.; k Bioinformatics Competence Center, University of Lausanne and EPFL, Lausanne, Switzerland; l SwissProt group, SIB Swiss Institute of Bioinformatics, Geneva, Switzerland; m Ifakara Health Institute, Dar es Salaam, Tanzania; n Department of Microbiology and Molecular Medicine, University of Geneva Medical School, Geneva, Switzerland; o Health2030 Genome Center, Geneva, Switzerland; p Agora Center, University of Lausanne, Lausanne, Switzerland; q Precision Medicine Unit, Lausanne University Hospital and University of Lausanne, Lausanne, Switzerland; r Geneva Centre for Emerging Viral Diseases, Geneva University Hospitals, Geneva, Switzerland

**Keywords:** Blood virome, virosphere, metagenomic next-generation sequencing, fever, children

## Abstract

Viral infections are the leading cause of childhood acute febrile illnesses motivating consultation in sub-Saharan Africa. The majority of causal viruses are never identified in low-resource clinical settings as such testing is either not part of routine screening or available diagnostic tools have limited ability to detect new/unexpected viral variants. An in-depth exploration of the blood virome is therefore necessary to clarify the potential viral origin of fever in children. Metagenomic next-generation sequencing is a powerful tool for such broad investigations, allowing the detection of RNA and DNA viral genomes. Here, we describe the blood virome of 816 febrile children (<5 years) presenting at outpatient departments in Dar es Salaam over one-year. We show that half of the patients (394/816) had at least one detected virus recognized as causes of human infection/disease (13.8% enteroviruses (enterovirus A, B, C, and rhinovirus A and C), 12% rotaviruses, 11% human herpesvirus type 6). Additionally, we report the detection of a large number of viruses (related to arthropod, vertebrate or mammalian viral species) not yet known to cause human infection/disease, highlighting those who should be on the radar, deserve specific attention in the febrile paediatric population and, more broadly, for surveillance of emerging pathogens.

**Trial registration:** ClinicalTrials.gov identifier: NCT02225769.

## Introduction

Humans are infected by a large number of eukaryotic DNA and RNA viruses that, together with prokaryotic phages and endogenous viral elements, comprise what we define as the human virome. Eukaryotic viruses are a diverse group of DNA and RNA viral agents replicating in a variety of human cells and tissues and engaging in a vast permutation of interactions with the human immune system. Consequently, the presentation of human viral disease depends on both viral and host factors, where a virus may only be pathological in a certain situation (e.g. following immunosuppression), whilst being considered (perhaps over simplistically) as commensal or merely an incidental finding in a “healthy” situation.

The composition of the human virome does not only vary between these populations but it also changes over time within individuals according to their personal history of exposure and immunity and thus, their geographic location, access to vaccination and their genetic make-up. Metagenomic next-generation sequencing (mNGS) has the ability to detect any viral RNA or DNA sequence in a given sample and thus, it represents the most appropriate tool with which to explore the human virome [1].

It is recognized that we have only uncovered a fraction of the so-called virosphere [2, 3]. While many human viruses have emerged from animal origin, shotgun surveillance in sentinel animals generates a lot of noise by reporting a majority of viruses unlikely to infect humans. Thus, we hypothesize that pathogen surveillance would be extremely informative to public health interventions if focused first on humans. Specifically, there is a need for investigations that could reveal not only the presence of known and expected viruses, but also divergent or unexpected ones. Characterization of the human virome could help to identify new sources of infections, possibly predict viral emergence or identify the potential deleterious effects of viruses considered as commensal.

The human virome has been described in stool, skin, nasopharyngeal swabs or breast milk [4]. Investigations were also undertaken on blood from individuals representing the general population [5], blood donors [6–9], and in subjects with various conditions such as transplant recipients [10–12], individuals living with HIV [13], and febrile adult and paediatric patients [1, 14–18]. However, most of these studies were limited by the scope of their analyses (e.g. addressing only RNA or DNA viruses, pooled samples) or in the time frame of included subjects.

**Figure 1.**
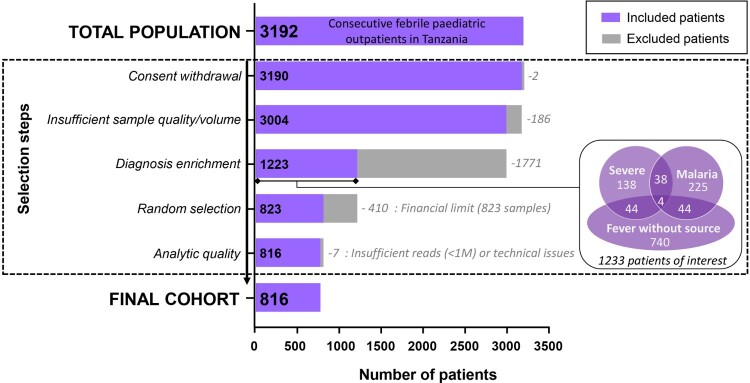
Flowchart of study selection.

Infections and fever in sub-Saharan Africa represent the most frequent motivation for paediatric outpatient consultation [19]. The majority of causal viruses, however, are never identified in low-resource clinical settings. In this study, we seek to characterize the blood virome in a cohort of 823 children consulting at nine outpatient departments in Dar es Salaam, Tanzania, for acute fever of unknown origin and/or severe outcomes (Figure 1) [20]. We tested each sample retrospectively by mNGS and analysed them using (a) a bioinformatic pipeline based on a novel viral reference database [21] to report vertebrate viruses and (b) by *de novo* assembly. We report the full diversity of their blood virome, including novel viruses and those unexpected or not previously reported in humans that deserve specific attention.

## Materials and methods

### Study design

The serum samples tested by mNGS come from a larger cohort of 3192 consecutively paediatric patients (2–59 months of age), recruited between December 2014 and February 2016, at nine outpatient clinics in Dar es Salaam, Tanzania, with the inclusion criteria of “acute febrile illness” (axillary temperature ≥ 37.5°C). The detailed characteristics of this cohort are described in Keitel et al [20].

A sub-cohort of interest comprising 1233 patients was then selected (Figure 1) based on three diagnoses which carried the highest theoretical probability of detecting blood viruses with significant clinical consequences and for which mNGS would most increase the diagnostic evidence for the aetiology of the illness. These diagnoses were obtained using by the clinical decision algorithms of the e-POCT study [20]: (1) “Fever without focus” representing children who would most benefit from further diagnostics; (2) “Severe illness,” representing children in whom the impact of blood viruses may be investigated. (3) “Malaria,” in whom blood viruses may interact with this important and frequent parasite and/or be less likely as the cause of the febrile episode.

A total of 823 patients (resulting in 823 sera samples) were then randomized from this sub-cohort of interest and submitted retrospectively to the entire process of mNGS (detailed below). Only samples with a minimum of one million reads for both RNA and DNA libraries were considered for further bioinformatic analysis, resulting in the exclusion of 4 samples. Three samples were further excluded; two consecutive to a technical problem encountered during the library procedures, and one due to an overrepresentation of insect sequences after analysis of raw sequencing data [22]. Therefore, 816 of the 823 initial selected samples were considered for analysis.

### Ethical approval

This study was approved by the local IRB in Tanzania by the Ifakara Health Institute and the National Institute for Medical research (paediatric cohort: IHI/IRB/EXT/16-2015 and NIMR/HQ/R.8a/Vol. IX/1789). IRB approval was also granted for analyses undertaken in Switzerland by the Ethikkommission Nordwest- und Zentralschweiz (EKNZ UBE-15/03). Written informed consent was obtained from the parent or guardian of the patients before enrolment. The trial was registered in ClinicalTrials.gov, identifier NCT02225769.

### Sample extraction and metagenomic next-generation sequencing

Two hundred twenty microliters (ul) of each serum sample was centrifuged 10,000× *g* for 10 min. Then, 200 ul are transferred in a new collection tube and treated with 40 U of Turbo DNAse (2U/ul) + 24 ul of 10X DNAse Buffer (Ambion, Rotkreuz, Switzerland). Of the total resulting 244, 240 μl were recovered to perform the nucleic acid extraction. Two nucleic acid extraction procedures were then carried out separately for RNA and DNA virus genome extraction (i.e. RNA and DNA protocols, respectively) as previously described [23]. Briefly, RNA genome extraction (RNA protocol) was isolated with TRIzol (Invitrogen, Carlsbad, CA, USA), taking care to collect only the upper aqueous layer to avoid DNA contaminations from the inter-/lower phases. DNA virus genome extraction (DNA protocol) was performed with the NucliSens easyMAG magnetic bead system (bioMérieux, Geneva, Switzerland) followed by a double-stranded DNA synthesis with DNA polymerase I, Large Fragment (Klenow) (New England BioLabs, Ipswich, MA, USA). RNA and DNA pellets were then resuspended in respectively 10 and 5 ul of nuclease-free water (Promega, Dübendorf, Switzerland), and quantified using the Qubit 3.0 Fluorometer (Life Technologies, Carlsbad, CA, USA) and adjusted at 2 ng/ul.

For RNA virus detection (RNA protocol), ribosomal RNA was removed using the Ribo-Zero Gold depletion kit (Illumina, San Diego, US) prior to library preparation. RNA libraries were prepared with the TruSeq total RNA preparation protocol (Illumina) before being multiplexed by four on the HiSeq 2500 platform (Illumina) using the 2 × 100 bp paired-end protocol. The mean of total number of read pairs obtained was 4.45E7 (range from 2E6 to 1.09E8).

For DNA virus detection (DNA protocol), libraries were prepared with the Illumina Nextera XT protocol (12 PCR cycles) before being multiplexed by six on the HiSeq 4000 platform (Illumina) using the 2 × 100 bp paired-end protocol. The mean of total number of read pairs obtained was 5.46E7 (range from 2E6 to 2.52E8).

Both RNA and DNA library concentrations were measured using Qubit (Life Technologies). The size distribution of fragments was controlled using a 2200 TapeStation (Agilent, Santa Clara, CA, USA).

To assess the presence of potential mNGS contaminants (i.e. false positive results), a “no-template” control (NTC) submitted to the whole procedure was included to each sequencing run. In order to assess the entire process efficiency (i.e. from sample preparation to bioinformatics analysis) each sequencing run included a virus-spiked positive control (PC). This latter is also useful in addition to the NTC samples to evaluate the presence of potential sporadic mNGS contaminants. For the RNA virus procedure, the PC consists of a canine distemper virus (CDV)-spiked positive control (RNA-PC), whereas for the DNA virus procedure, a baculovirus (GenScript, Piscataway, NJ, USA) harboring 793 nucleotides of the CDV fusion gene (corresponding to positions 5505 to 6297 from the GenBank KY971529 reference) was used as spiked-positive controls (DNA-PC).

### Bioinformatic analysis

For each sample, the reads generated by the Illumina platforms (RNA and DNA libraries) were processed for virus detection using a bioinformatics pipeline (*FeVir*) designed to detect all vertebrate viruses and a *de novo* assembly sequencing approach.

### Bioinformatics pipeline for vertebrate virus detection (*FeVir*)

Paired reads were quality filtered and mapped against Virosaurus (version V90v_2018_11) [21], a curated database for all known vertebrate viruses, using virusscan 1.0 [24]. The mapping was performed allowing up to 12 mismatches per tag but at most 15 mismatches for the pair, ultimately retaining the lowest possible mismatch. Sequence pairs whose lowest mismatch count mapped equally well onto more than 512 distinct viruses entries were discarded. Non-redundant mapping results were used to compute metrics for mapped reads and coverage. Then for each virus species (or genus for anelloviridae) detected, results with the best total genome coverage value were kept. To minimize false positives, only results with coverage ≥300 nucleotides (i.e. a minimal of 3 independent and non-overlapping reads) were considered as positive [23, 25].

Index hopping/lane cross-contamination was removed using a 1% threshold. An important challenge in the interpretation of mNGS data is to distinguish clinically significant viruses from those that are known to be medically irrelevant and also to differentiate novel viruses (not previously described in humans) from those that are more likely to be environmental or mNGS reagent contaminants [26]. Therefore, results for viruses detected in processes controls and endogenous retrovirus were not reported.

For segmented viruses, results for each segment were summed. For *Herpesviridae*, each gene’s results were summed using a non-redundant and non-overlapping list of genes.

Viral hits were classified into 3 groups:

1. Those of “recognized clinical significance” including viruses known to infect humans and well recognized in clinical practice as disease-causing agents. As for most pathogens the spectrum of associated illness associated with this group of viruses ranges from mild symptoms (or even asymptomatic infection) to life-threatening disease.
2. Commensal viruses of “undetermined clinical significance” that comprise viruses that infect human but for which the association with a specific disease has not yet been established. Most of these viruses however have shown to interact with the human immune response and have been referred to by some as commensal agents. The two main representatives are *Anelloviridae* and human pegivirus-1.
3. Those of “unknown significance” that represent viral agents not usually recognized as infecting humans and/or have never fulfilled Koch’s postulates that would attribute a theorical pathogenic role [27]. Thus, it could reflect transient or aborted infections, environmental spill over, contaminants from skin or other body fluids or spurious mNGS reagent contaminants. We consider this group of particular interest to surveillance efforts, since it could signal contact with potential emerging human pathogens and guide targeted screening in the future.

### De novo analysis

The *de novo* analysis was performed as previously published [28]. First, reads were trimmed to remove low-quality and adapter sequences using Trimmomatic (v0.33). Human reads were removed by mapping reads against the human genome and transcriptome (hg38, gencode.V23) using the SNAP nucleotide aligner program [29]. Then, *de novo* assembly was performed using IDBA-UD (v.1.1.3) [30] and the generated contigs of greater than 2000 nucleotides were blasted (blastx, v.2.3.0+) [31] against the U-RVDBv12.2 viral database [32]. *Anelloviridae* contigs were filtered out and processed separately. The newly obtained sequences were then cross-checked by BLAST (blastn, blastx) against GenBank (Nucleotid collection (nr/nt), Non-redundant protein sequences (nr)) and NCBI conserved domain tool. Novel virus contigs with a presumed bacterial or fungi host were not considered for further analyses.

Sample and control FASTQ files were additionally filtered for low-complexity sequences, using tagdust (v2.31). Remaining reads were mapped against assembled genomes that were not already detected by the previously described *FeVir* pipeline, using SNAP. For each new hit, mapped reads and coverage metrics were then computed. Only results with ≥300 nucleotides of coverage were considered. Results for viruses detected in processes controls were not reported.

### *Anelloviridae* sequence analysis

*Anelloviridae* contigs with a complete ORF1 larger than 600 amino acids were separated into Alpha-, Beta- and Gamma-torquevirus genera using multiple alignment (muscle v3.8.31) with the reference sequences of ORF1. Alternative “ACG” initiation codon was also used to detect Alphatorquevirus ORF1.

To estimate the proportion of assembled contigs that was “new” compared to reported sequences in GenBank (Query: alphatorquevirus [Organism] OR betatorquevirus [Organism] OR gammatorquevirus [Organism] OR unclassified Anelloviridae [Organism], downloaded in March 2019), we first excluded non-primate sequences, then filtered as above and added to the new assembled sequences. For the three genera, complete ORF-1 sequences were separated in clusters of 80% nucleotide identity, using cdhit-est (CD-HIT v4.6). For each cluster, sequences of 90% identity were selected using cdhit-est. Then representative sequences were aligned two by two using muscle (v3.8.1551), when two clusters contain reference sequences that shared more than 80% nucleotide identity, clusters were merged.

To determine the number of distinct *Anelloviridae* of the same genus present in each sample, the filtered FASTQ files were mapped to the ORF1 representative sequence using SNAP, for each cluster, positive detection was considered if 50% or more of the ORF1 was covered.

Additionally, for each of the assembled *Anelloviridae* contigs that contained a complete ORF1, circular genomes were linearized starting after the GC rich region and incomplete genomes were trimmed the same way. Finally, representative genomes were selected at 90% nucleotide identity with cdhit-est. Then filtered FASTQ files were mapped against the representative genomes with SNAP. Metrics were computed and results were processed as described above (≥300 nucleotides, 1% index-hopping/lane cross-contamination threshold). Results with the best total genome coverage value for each genus were reported if previously found negative with the *FeVir* pipeline.

## Results

In this large population of febrile paediatric outpatients recruited in East Africa, we report the detection of a large diversity of RNA and DNA virus sequences that we classify into 3 groups (Supplementary Figure 1, https://www.unige.ch/virology/en/krona/); those known to cause disease in humans and of “recognized clinical significance” (Supplementary Figure 1(a)), those known to be present in humans and of “undetermined clinical significance” (Supplementary Figure 1(b)) and a third group of viruses not usually described in humans and of “unknown significance” (Supplementary Figure 1(c)).

### Viruses of “recognized clinical significance”

Of the 816 children, 394 (48.3%) harboured RNA and/or DNA sequences specific for a virus recognized as causing human diseases (Supplementary Table 1). This group was dominated by RNA viruses, predominantly from members of the *Picornaviridae* family such as human enteroviruses (EV) (13.8% of all cases, 113/816) and rotaviruses (12% of all cases, 98/816) (Supplementary Figure 1(a)).

**Figure 2.**
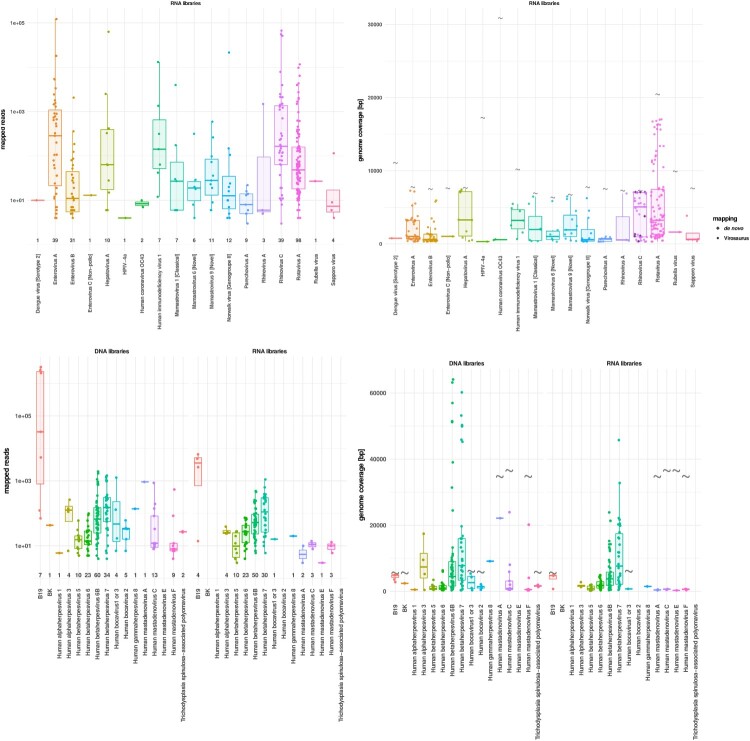
mNGS metrics for viruses of recognized clinical significance. (a) Distribution of the mapped reads for RNA viruses. Each dot represents a positive sample. (b) RNA virus genome coverages. Viral sequences detected by *de novo* only are represented by grey diamonds. Each dot represents one sample. Viral full genome sizes (bp) are indicated by grey corrugated symbols. (c) Distribution of the mapped reads for DNA viruses detected in DNA (left side) and RNA (right side) libraries. Each dot represents a positive sample. (d) DNA virus genome coverages in DNA (left side) and RNA (right side) libraries. Each dot represents one sample. Viral full genome sizes (bp) are indicated by grey corrugated symbols (except for herpesviruses that have too long genomes to figure in the panel). The horizontal lines in the box plots denote medians.

Species from various genera of the *Picornaviridae* family were detected, including EV-A, B and C (*n* = 39, 31 and 1, respectively), rhinovirus (RV)-A and C (*n* = 3 and 39, respectively), hepatovirus A (*n* = 10) and parechovirus A (*n* = 9) (Figure 2(a)). Within the different EV species, various coxsackievirus A and echovirus genotypes were observed (Supplementary Table 1), as well as one EV-A71 case that may represent a new EV-A71 genogroup (Supplementary Figure 2, SAfia-57/TZA/2016 MN727165.1). Among EV-C, poliovirus-related sequences were not observed. A large diversity of RV-C were detected (Supplementary Figure 3), nine of which were only detectable by *de novo* analysis (Figure 2(b)), highlighting the absence of similar sequences in any available database. The 17 RV-C for which a complete VP1 sequence was recovered, could be clustered into 9 distinct RV types (including one that may, tentatively, be categorized as a new type). One of the genomes (SAfia-94/TZA/2015 MN727266.1) is an RV-C45/RV-C11 recombinant similar to a variant described in a wild chimpanzee in Uganda [33].

A total of 98/816 (12%) samples had detectable rotavirus sequences, and only one corresponded to the vaccine G1P [8] strain (Supplementary Figure 1(a)). Human astroviruses (HAstV) were the 3rd most frequently detected among RNA viruses from this group. The classical HAstV (i.e. HAstV serotypes 1-8) that are a well-known cause of gastroenteritis, represented only a minority of cases (*n* = 7) whereas the novel HAstV (i.e. HAstV MLB/VA) were observed in 17 cases (4 MLB1, 2 MLB2, 5 VA1 and 6 VA3). We also identified Norwalk virus (*n* = 12), human immunodeficiency virus 1 (*n* = 7), Sapporo virus (*n* = 4), human coronavirus OC43 (*n* = 2), human parainfluenza virus type 4a (*n* = 1) and the Wistar RA 27/3 rubella vaccine strain in one case. Finally, only one patient had detectable sequences of dengue virus (serotype 2), which corroborates clinical diagnoses and on-site rapid testing, that the study did not occur during a dengue virus outbreak.

Concerning the DNA viruses, by far the most frequent ones were members of the *Betaherpesvirinae* subfamily followed by mastadenovirus species. Notably, a significant proportion of these agents were also recovered at the RNA level (i.e. RNA protocol).

Among *Betaherpesvirinae* (Supplementary Figure 1(a)), human herpesvirus type 6 (HHV-6) was the most frequent (*n* = 90; 63 belonging to the HHV-6 B species and 27 remaining untyped), followed by human herpesvirus type 7 (HHV-7) (*n* = 36) and cytomegalovirus (CMV) (*n* = 15) (Figure 2(c, d)). In addition, the following herpesviruses were identified in a few samples: varicella zoster virus (*n* = 4), herpes simplex type 1 virus (*n* = 1), and human herpesvirus 8 (*n* = 1). Among all HHV-6, HHV-7 and CMV positive samples listed above, a large proportion (73.3%, 75.7% and 33.3%, respectively) were detected in both DNA and RNA libraries suggesting the presence of ongoing genome replication. Of note, 7 HHV-6, 2 HHV-7 and 5 CMV positive cases were detected in the RNA libraries only, which means that, overall, 81.1% of HHV-6 (*n* = 73/90), 83.3% of HHV-7 (*n* = 30/36) and 66.6% of CMV (*n* = 10/15) positive cases were detected in the RNA libraries.

Specific sequences for human mastadenovirus (HAdV) were recovered in 28 samples and encompassed species A, C, E and F. Human bocavirus, parvovirus B19, trichodysplasia spinulosa-associated polyomavirus and BK virus were recovered in 10 or less samples each (Figure 2(c)). A fraction of these DNA viruses was also detected in the RNA libraries only (five HAdVs (one each for species A, E and F, two for the species C) and one human bocavirus) (Figure 2(c)).

### Commensal viruses of “undetermined clinical significance”

**Figure 3.**
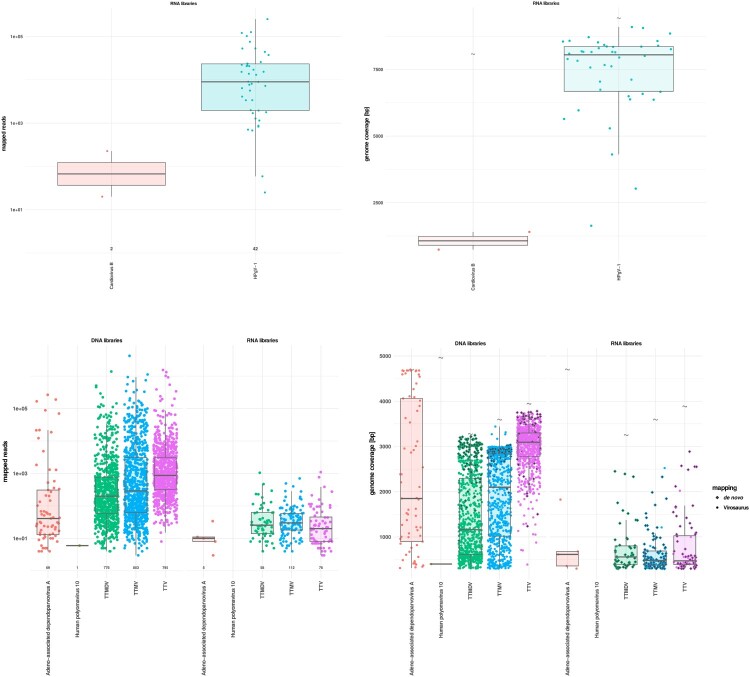
mNGS metrics for viruses of undetermined clinical significance. (a) Distribution of the mapped reads for RNA viruses. Each dot represents a positive sample. (b) RNA virus genome coverages. Each dot represents one sample. Viral full genome sizes (bp) are indicated by grey wave symbols. (c) Distribution of the mapped reads for DNA viruses detected in DNA (left side) and RNA (right side) libraries. Each dot represents a positive sample. (d) DNA virus genome coverages in DNA (left side) and RNA (right side) libraries. Each dot represents one sample. Viral full genome sizes (bp) are indicated by grey corrugated symbols. Viral sequences detected by *de novo* only are represented by grey diamonds. The horizontal lines in the box plots denote medians.

**Figure 4.**
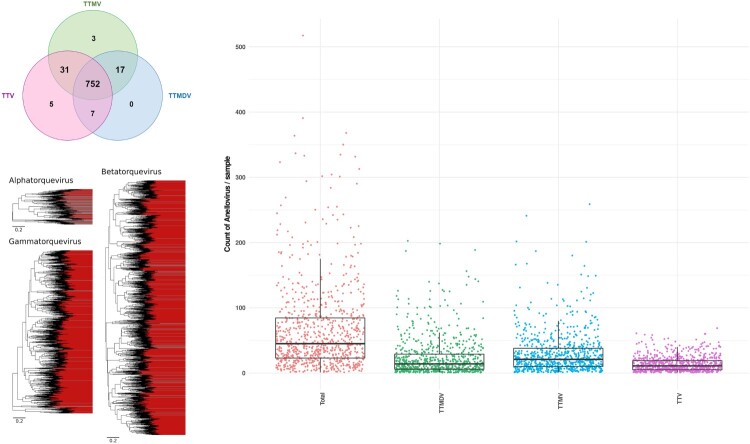
Representation of *Anelloviridae* co-detection. (a) Venn plot showing the distribution of Alpha-, Beta- and Gammatorquevirus genus among positive samples. (b) Phylogenetic diversity of primate anellovirus. Alpha-, Beta- and Gammatorquevirus genus are represented by three Maximum Likelihood trees based on complete ORF1 nucleotide sequences. Each branch is a representative sequence of a cluster, each cluster shows at least 20% nucleotide diversity with others. Clusters whose contain previously known complete ORF1 sequences are shaded grey, clusters with only sequences assembled in this study are shaded red. Scale is in number of substitutions per site. (c) Number of distinct anelloviruses of the same genus (i.e. Alpha-, Beta- and Gammatorquevirus) or in total co-detected in each positive sample. The horizontal lines in the box plots denote medians. Each dot represents one sample.

*Anelloviridae* sequences were detected in all except one clinical sample (Supplementary Figure 1(b) and Figure 3). Co-infection with multiple genera was the rule (99.0%), with 752/816 samples found positive for all 3 genera (Figure 4(a)). Torque teno mini virus (TTMV, *n* = 803) was the most frequent, followed by Torque teno virus (TTV, *n* = 795), and Torque teno midi virus (TTMDV, *n* = 776) (Figure 3(c, d) and Figure 4(a)). Sequence analyses revealed a very high genetic heterogeneity (Figure 4(b)) with an extremely large number of divergent and/or different genotypes from the same genus present in a single sample (Figure 4(c)). As an example, up to 69 different TTV, 259 TTMV and 189 TTMDV genotypes were observed in a single clinical sample (Supplementary Table 1, sample ID 25161).

In addition**,** we detected sequences corresponding to adeno-associated dependoparvovirus A (*n* = 69), human pegivirus-1 (HPgV-1) (*n* = 42), cardiovirus B (*n* = 2), and human polyomavirus 10 (*n* = 1) (Supplementary Figure 1(b) and Figure 3(a–d)).

### Viruses of “unknown significance”

**Figure 5.**
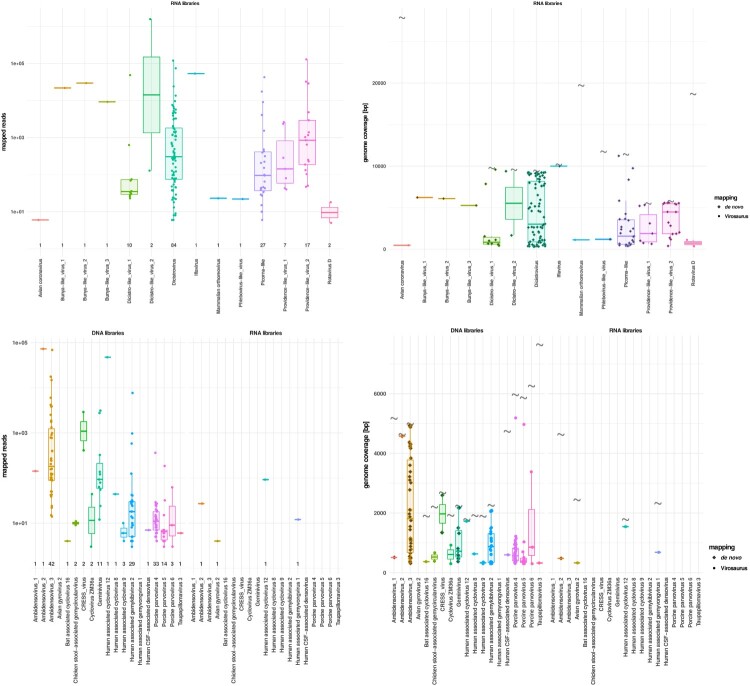
mNGS metrics for viruses of yet unknown significance. (a) Distribution of the mapped reads for RNA viruses. Each dot represents a positive sample. (b) RNA virus genome coverages. Each dot represents one sample. Viral full genome sizes (bp) are indicated by grey corrugated symbols (except for the segmented bunya-like virus 1–3). Viral sequences detected by *de novo* only are represented by grey diamonds. (c) Distribution of the mapped reads for DNA viruses detected in DNA (left side) and RNA (right side) libraries. Each dot represents a positive sample. (d) DNA virus genome coverages in DNA (left side) and RNA (right side) libraries. Each dot represents one sample. Viral full genome sizes (bp) are indicated by grey corrugated symbols. The horizontal lines in the box plots denote medians.

A large number of sequences related to arthropod, vertebrate or mammalian viruses (DNA and RNA-based) that are not recognized as a cause of infection in humans were detected using either the *FeVir* pipeline and/or *de novo* analysis (Supplementary Figure 1(c) and Figure 5). We describe a tentative novel *Dicistroviridae* genus (a family of viruses known to infect arthropods) identified in 84 samples and reported separately in Cordey et al [34]. Porcine parvoviruses 4–6 (*n* = 50) were also detected, including two nearly complete sequences (Figure 5(c, d)). Ambidensoviruses 1–3, known to infect insects and other non-vertebrates, were present (*n* = 44) with a significant number of reads detected in the DNA libraries (Figure 5(c, d)).

Several additional viruses for which no RNA or DNA sequences were detected in the NTC and PC samples were observed. At the RNA level, several viruses were detected that could be reported as “-like” known viruses, without however matching closely to them. This included sequences for picorna-like virus (*n* = 27), providence-like viruses 1–2 (*n* = 24), dicistro-like viruses 1–2 (*n* = 12), bunya-like virus 1–3 (*n* = 3), and phlebovirus-like virus (*n* = 1) (Figure 5(a, b)). Others were rotavirus D (*n* = 2), avian coronavirus (*n* = 1), iflavirus (*n* = 1), and mammalian orthoreovirus (*n* = 1) (Figure 5(a, b)). Among DNA viruses, sequences were also detected for human associated gemykibivirus (*n* = 29), geminivirus (*n* = 11), human associated cycloviruses (*n* = 5), chicken stool-associated gemycircularvirus (*n* = 2), CRESS virus (*n* = 2), cyclovirus ZM36a (*n* = 2), avian gyrovirus 2 (*n* = 1, RNA library only), bat associated cyclovirus 16 (*n* = 1), human associated gemyvongvirus 1 (*n* = 1, RNA library only), human CSF-associated densovirus (*n* = 1), and taupapillomavirus 3 (*n* = 1) (Figure 5(c, d)).

## Discussion

The aim of this study was to provide a comprehensive characterization of the blood virome (i.e. viral landscape) in a population of young children with fever of unknown origin and/or severe outcomes who are living in Tanzania to pave the way of future clinical and diagnostic investigations. Beyond the well-recognized human viruses known to cause diseases and for some of them screened in routine clinical practice, or widely considered as “commensal,” we identify numerous novel, divergent and unexpected viruses that show mNGS to be a powerful tool for the surveillance of unrecognized or emerging viral infections. Among these unexpected viruses, were a large proportion never before described in humans, of mammalian, arthropod or other invertebrate origin. As the detection of a viral sequence does not necessarily reflect the cause of the disease, further matched studies on a broader geographic, demographic and clinical scope would be required to better differentiate between background noise, abortive infections or novel causes of clinical disease.

Many previous investigations have surveyed viruses in the animal kingdom, including non-vertebrates, in order to extrapolate a probabilistic list of viruses that could emerge in humans [35–37]. However, this generates noise by reporting a vast majority of viruses which would not succeed in the stochastic process of a zoonotic transfer event or have not yet acquired the mutations with which to do. We propose that such surveillance should be complemented by large sentinel human virome populations that is much more likely to identify viral signatures of interest, that could guide future extended investigations.

We conducted two bioinformatics analyses in parallel for each sample: In addition to traditional *de novo* analysis, the raw data were analysed with the *FeVir* pipeline (specifically designed for this study and including the novel and particularly exhaustive *Virosaurus* reference database). The mNGS analysis reported the detection of a large diversity of RNA and DNA virus sequences. Of the 816 children, 394 (48.3%) were found positive for at least one virus of “recognized clinical significance.” *Anelloviridae* were detected in all except one sample and up to 92.2% of samples were found co-infected by all three anellovirus genera (TTV, TTMV and TTMDV). In addition, a large number of sequences were detected that related to 33 arthropod, vertebrate or mammalian virus species that are so far unknown to cause human infections; the most frequent being dicistroviruses, porcine parvoviruses 4–6 and ambidensoviruses.

To assess the presence of potential environmental or laboratory reagent contaminants, NTC and PC submitted to the whole procedure were included to each series. Despite this, mNGS data must be interpreted with caution (especially when the number of reads and/or positive samples is low) as sporadic contaminations cannot be ruled out e.g. the mNGS analysis reported in the blood for so-called respiratory viruses only one HPIV-4a positive sample with 4 reads detected as well as two human coronavirus OC43 positive samples with ≤10 reads each (although they were collected in two different districts, Supplementary Figure 4) (Supplementary Table 1).

Concerning the viruses considered as being of “recognized clinical significance,” sequences related to *Herpesviridae*, *Picornaviridae* and the *Reoviridae* family members were the most frequently detected among the 4 DNA (*Herpesviridae* > *Adenoviridae* > *Parvoviridae* > *Polyomaviridae*) and 9 RNA (*Picornaviridae* > *Reoviridae* > *Astroviridae*, *Caliciviridae* > *Retroviridae* > *Coronaviridae* > *Flaviviridae* > *Paramyxoviridae* > *Togaviridae*) virus families observed (Supplementary Figure 1(a)). Indeed, among the 35 viral species detected and considered of clinical significance, we observed a predominance of human enteroviruses (13.8%), rotaviruses (12%), HHV- 6 (11%) and HHV-7 (4.4%). Overall, rotavirus A sequences were the most frequently detected and confirm that rotavirus infections frequently result in viremia in paediatric patients [38, 39]. Similarly to HHV-6, HHV-7 was also frequently detected (*n* = 36) in our cohort. Although commercial or in-house molecular assays are available, HHV-7 is rarely (if ever) tested even in high-resource settings. Although great care was taken during the RNA virus genome extraction (RNA protocol), we cannot rule out rare DNA contaminations from inter-/lower phases after phase separation. However, given their significant proportions, the detection of RNA for HHV-6 and HHV-7 in the RNA libraries suggest ongoing viral replication in the majority of positive cases. Some viruses are also notable for their absence, for instance, Epstein–Barr was not detected by mNGS analysis in any children. We also show the wide circulation of EV in East Africa, and report a potential new EV-A71 genogroup H. Additional investigations on the prevalence and global distribution of this genogroup as well as its associated disease will give potentially important information about its neurotropic potential. Continuous genomic studies on the evolution of EV-A71 are important to detect new mutants or recombinants with epidemic potential [40]. Such surveillance is necessary, particularly as there is currently no treatment against this virus.

Interestingly, although its relationship with clinical pathology is currently difficult to establish, among different *Picornaviridae* family members we observed two samples harbouring Cardiovirus B. This virus was isolated in cell culture from stool samples collected from a child with fever of unknown origin and named the Saffold virus [41]. Since then, the virus has shown a wide circulation in many countries [42] and has been isolated from clinical specimens of children with gastrointestinal and respiratory symptoms as well as from cerebrospinal fluid samples of patients with aseptic meningitis. Recently, Ramesh and colleagues also reported the detection of Saffold virus sequences with mNGS in the serum of a febrile paediatric patient in Uganda [17]. Among the EV genera, one RV-C45/RV-C11 recombinant was observed. This recombinant was previously reported in human respiratory specimen collected in US [43] and our data suggest its circulation across Africa. Interestingly, nine of the 39 RV-C were detected by the *de novo* analysis only. This demonstrates that, although more time-consuming, a *de novo* approach is complementary to database-oriented pipelines and recommended in such surveillance studies to detect highly divergent viruses. To support this statement, 13 RV-C and 13 RV-A genomes were *de novo* assembled by Ramesh A and colleagues in their mNGS study [17].

Our results further highlight that HAstV has a significant circulation in the global population, with a higher prevalence of novel HAstV (2.1%) than classical HAstV (0.9%). This observation is in agreement with previous reports and underscores the clinical pertinence of novel HAstV in the paediatric population [44–46].

The potential clinical implication of *Anelloviridae* is still debated [47]. Among sera samples analysed in this study, 98.4%, 97.4% and 95.1% were found positive for TTMV, TTV and TTMDV, respectively, which support a very early acquisition in life [48] with increased TTV and TTMDV prevalence in the paediatric population until 19–24 months of age before decreasing (modest age-related effect observed for TTMV) [49]. *Anelloviridae* are considered major members of the human blood virome and possibly one of the most abundant virus in blood samples [50]. Reflecting their genetic heterogeneity [51], anellovirus mixed populations are frequently observed in blood samples [52, 53]. In agreement with these previous studies, we report a very high intra- and inter-individual genetic heterogeneity for TTV, TTMV and TTMDV, with a co-detection rate of 99.0% (Figure 4). This is exemplified here by the finding that up to 69 different TTV, 259 TTMV and 189 TTMDV were observed in the serum from a single individual. Similar to HPgV-1, TTV are known to interact with the immune system; yet the impact of these interactions is not well defined.

A wide range of RNA and DNA viruses previously unknown to infect humans were observed. These deserve specific attention in further studies in the febrile paediatric population in Africa. Indeed, the aim of this study was to characterize the diversity of the viral landscape in the blood of febrile Tanzanian children. However, while these viruses were discovered in febrile children, the absence of a non-febrile comparison group means that we cannot definitively associate the febrile episode to the presence of the viral sequences. While this specific question is beyond the scope of our study, the associations between the considerable diversity of metagenomics data and clinical features is yet to be explored. Therefore, although extremely challenging, it would be important to perform a similar mNGS survey analysis on blood samples from an asymptomatic paediatric cohort with comparable demographic and epidemiological characteristics to better clarify any potential clinical causality.

In conclusion, this blood virome study on a population of young children with fever of unknown origin and/or severe outcomes living in Tanzania highlighted several DNA and RNA viral sequences, and provides a large list of novel viruses that should be considered as a potential cause of human infections, some certainly being candidates for future fever investigations.

## Supplementary Material

Supplemental Material

## Data Availability

The raw sequence data were deposited in the NCBI Sequence Read Archive under BioProject accession number PRJNA666535. Partial viral sequences ≥ 2000 nucleotides long obtained in this study were submitted to GenBank (accession no. MN727142-MN727295 and MN765166-MN780477 for RNA and DNA viruses, respectively). The custom scripts that were used in this study are available at: https://github.com/SAFIA-HTS/VIRAL-HTS.
