# Supplementary material for: Blood virosphere in febrile Tanzanian children
Source: Emerg Microbes Infect. 2021 May 28;10(1):982–93. doi: 10.1080/22221751.2021.1925161 (PMC8171259; doi:10.1080/22221751.2021.1925161)
Supplement: Supplemental Material [file TEMI_A_1925161_SM1971.zip › Suppl files/Supplementary Table S1.docx]

| **sample ID** | **sample collection date** | **library_ID** | **run_lane** | **virus** | **family** | **% coverage** | **depth** | **Mapped reads** | **Covered (bp)** | **Genome (bp)** | **genome_ID** | **mapping** | **Library** |
| --- | --- | --- | --- | --- | --- | --- | --- | --- | --- | --- | --- | --- | --- |
| 21053 | 2/6/2015 | 104_D | L007-r49 | Adeno-associated dependoparvovirus A | Parvoviridae | 39.08 | 2 | 51 | 1829 | 4680 | NC_001401 | Virosaurus | DNA library |
| 21074 | 2/19/2015 | 107_D | L003-r49 | Adeno-associated dependoparvovirus A | Parvoviridae | 8.85 | 1 | 10 | 414 | 4680 | NC_001401 | Virosaurus | DNA library |
| 21105 | 3/2/2015 | 120_D | L001-r49 | Adeno-associated dependoparvovirus A | Parvoviridae | 47.18 | 1 | 41 | 2208 | 4680 | NC_001401 | Virosaurus | DNA library |
| 21148 | 3/13/2015 | 126_D | L008-r49 | Adeno-associated dependoparvovirus A | Parvoviridae | 62.42 | 1 | 63 | 2921 | 4680 | NC_001401 | Virosaurus | DNA library |
| 21158 | 3/17/2015 | 127_D | L001-r49 | Adeno-associated dependoparvovirus A | Parvoviridae | 17.52 | 2 | 15 | 820 | 4680 | NC_001401 | Virosaurus | DNA library |
| 21176 | 3/16/2015 | 131_D | L006-r49 | Adeno-associated dependoparvovirus A | Parvoviridae | 8.55 | 1 | 5 | 400 | 4680 | NC_001401 | Virosaurus | DNA library |
| 21258 | 4/15/2015 | 217_D | L006-r55 | Adeno-associated dependoparvovirus A | Parvoviridae | 23.06 | 1 | 14 | 1079 | 4680 | NC_001401 | Virosaurus | DNA library |
| 21333 | 5/11/2015 | 229_D | L004-r61 | Adeno-associated dependoparvovirus A | Parvoviridae | 21.43 | 1 | 13 | 1003 | 4680 | NC_001401 | Virosaurus | DNA library |
| 21339 | 5/12/2015 | 230_D | L005-r61 | Adeno-associated dependoparvovirus A | Parvoviridae | 100 | 445 | 21356 | 4680 | 4680 | NC_001401 | Virosaurus | DNA library |
| 21353 | 5/13/2015 | 234_D | L002-r61 | Adeno-associated dependoparvovirus A | Parvoviridae | 9.36 | 1 | 5 | 438 | 4680 | NC_001401 | Virosaurus | DNA library |
| 21377 | 5/18/2015 | 238_D | L006-r61 | Adeno-associated dependoparvovirus A | Parvoviridae | 18.21 | 1 | 12 | 852 | 4680 | NC_001401 | Virosaurus | DNA library |
| 21441 | 5/25/2015 | 248_D | L001-r61 | Adeno-associated dependoparvovirus A | Parvoviridae | 31.35 | 2 | 43 | 1467 | 4680 | NC_001401 | Virosaurus | DNA library |
| 21447 | 5/25/2015 | 249_D | L002-r61 | Adeno-associated dependoparvovirus A | Parvoviridae | 50.96 | 2 | 55 | 2385 | 4680 | NC_001401 | Virosaurus | DNA library |
| 21508 | 6/1/2015 | 263_D | L002-r61 | Adeno-associated dependoparvovirus A | Parvoviridae | 93.85 | 6 | 308 | 4392 | 4680 | NC_001401 | Virosaurus | DNA library |
| 21521 | 6/4/2015 | 268_D | L008-r61 | Adeno-associated dependoparvovirus A | Parvoviridae | 21.92 | 1 | 13 | 1026 | 4680 | NC_001401 | Virosaurus | DNA library |
| 21542 | 6/8/2015 | 274_D | L4-r65 | Adeno-associated dependoparvovirus A | Parvoviridae | 98.61 | 6 | 332 | 4615 | 4680 | NC_001401 | Virosaurus | DNA library |
| 21567 | 6/12/2015 | 278_D | L007-r67 | Adeno-associated dependoparvovirus A | Parvoviridae | 100 | 3937 | 189096 | 4680 | 4680 | NC_001401 | Virosaurus | DNA library |
| 21567 | 6/12/2015 | 278_R | L007-r692 | Adeno-associated dependoparvovirus A | Parvoviridae | 39 | 1 | 34 | 1825 | 4680 | NC_001401 | Virosaurus | RNA library |
| 21627 | 6/24/2015 | 602_D | L1-r89 | Adeno-associated dependoparvovirus A | Parvoviridae | 16.24 | 1 | 10 | 760 | 4680 | NC_001401 | Virosaurus | DNA library |
| 21849 | 8/4/2015 | 319_D | L4-r66 | Adeno-associated dependoparvovirus A | Parvoviridae | 19.17 | 1 | 19 | 897 | 4680 | NC_001401 | Virosaurus | DNA library |
| 21851 | 8/4/2015 | 320_D | L5-r66 | Adeno-associated dependoparvovirus A | Parvoviridae | 85.73 | 116 | 4854 | 4012 | 4680 | NC_001401 | Virosaurus | DNA library |
| 21937 | 8/18/2015 | 330_D | L8-r66 | Adeno-associated dependoparvovirus A | Parvoviridae | 100 | 249 | 12232 | 4680 | 4680 | NC_001401 | Virosaurus | DNA library |
| 22041 | 9/1/2015 | 342_D | L6-r66 | Adeno-associated dependoparvovirus A | Parvoviridae | 27.05 | 1 | 23 | 1266 | 4680 | NC_001401 | Virosaurus | DNA library |
| 22107 | 9/9/2015 | 352_D | L1-r66 | Adeno-associated dependoparvovirus A | Parvoviridae | 91.2 | 4 | 203 | 4268 | 4680 | NC_001401 | Virosaurus | DNA library |
| 22110 | 9/9/2015 | 354_D | L3-r66 | Adeno-associated dependoparvovirus A | Parvoviridae | 63.14 | 2 | 58 | 2955 | 4680 | NC_001401 | Virosaurus | DNA library |
| 22203 | 9/21/2015 | 365_D | L001-r73 | Adeno-associated dependoparvovirus A | Parvoviridae | 77.52 | 6 | 271 | 3628 | 4680 | NC_001401 | Virosaurus | DNA library |
| 22276 | 10/9/2015 | 377_D | L003-r71 | Adeno-associated dependoparvovirus A | Parvoviridae | 64.32 | 2 | 68 | 3010 | 4680 | NC_001401 | Virosaurus | DNA library |
| 22349 | 11/2/2015 | 11_D | L004-r44 | Adeno-associated dependoparvovirus A | Parvoviridae | 8.29 | 1 | 4 | 388 | 4680 | NC_001401 | Virosaurus | DNA library |
| 22405 | 10/22/2015 | 393_D | L001-r73 | Adeno-associated dependoparvovirus A | Parvoviridae | 24.55 | 1 | 25 | 1149 | 4680 | NC_001401 | Virosaurus | DNA library |
| 22436 | 11/6/2015 | 13_D | L006-r44 | Adeno-associated dependoparvovirus A | Parvoviridae | 7.33 | 1 | 5 | 343 | 4680 | NC_001401 | Virosaurus | DNA library |
| 22462 | 11/18/2015 | 35_D | L007-r44 | Adeno-associated dependoparvovirus A | Parvoviridae | 10.41 | 1 | 8 | 487 | 4680 | NC_001401 | Virosaurus | DNA library |
| 22488 | 11/26/2015 | 47_D | L002-r46 | Adeno-associated dependoparvovirus A | Parvoviridae | 29.32 | 1 | 23 | 1372 | 4680 | NC_001401 | Virosaurus | DNA library |
| 22663 | 1/27/2016 | 57_D | L005-r46 | Adeno-associated dependoparvovirus A | Parvoviridae | 87.8 | 3 | 134 | 4109 | 4680 | NC_001401 | Virosaurus | DNA library |
| 25029 | 1/21/2015 | 795_D | L6-r111 | Adeno-associated dependoparvovirus A | Parvoviridae | 39.77 | 1 | 28 | 1861 | 4680 | NC_001401 | Virosaurus | DNA library |
| 25033 | 1/22/2015 | 415_D | L001-r75 | Adeno-associated dependoparvovirus A | Parvoviridae | 9.92 | 1 | 6 | 464 | 4680 | NC_001401 | Virosaurus | DNA library |
| 25055 | 1/30/2015 | 418_D | L004-r75 | Adeno-associated dependoparvovirus A | Parvoviridae | 83.25 | 31 | 1290 | 3896 | 4680 | NC_001401 | Virosaurus | DNA library |
| 25099 | 2/17/2015 | 428_D | L005-r73 | Adeno-associated dependoparvovirus A | Parvoviridae | 7.91 | 1 | 5 | 370 | 4680 | NC_001401 | Virosaurus | DNA library |
| 25117 | 2/20/2015 | 435_D | L005-r73 | Adeno-associated dependoparvovirus A | Parvoviridae | 44.98 | 1 | 44 | 2105 | 4680 | NC_001401 | Virosaurus | DNA library |
| 25137 | 2/26/2015 | 437_D | L001-r75 | Adeno-associated dependoparvovirus A | Parvoviridae | 61.24 | 2 | 68 | 2866 | 4680 | NC_001401 | Virosaurus | DNA library |
| 25151 | 3/3/2015 | 442_D | L005-r73 | Adeno-associated dependoparvovirus A | Parvoviridae | 34.21 | 1 | 24 | 1601 | 4680 | NC_001401 | Virosaurus | DNA library |
| 25162 | 3/5/2015 | 445_D | L002-r75 | Adeno-associated dependoparvovirus A | Parvoviridae | 39.53 | 1 | 32 | 1850 | 4680 | NC_001401 | Virosaurus | DNA library |
| 25163 | 3/5/2015 | 446_D | L004-r75 | Adeno-associated dependoparvovirus A | Parvoviridae | 98.18 | 1908 | 88181 | 4595 | 4680 | NC_001401 | Virosaurus | DNA library |
| 25163 | 3/5/2015 | 446_R | L004-r705 | Adeno-associated dependoparvovirus A | Parvoviridae | 7.67 | 2 | 10 | 359 | 4680 | NC_001401 | Virosaurus | RNA library |
| 25179 | 3/9/2015 | 450_D | L006-r73 | Adeno-associated dependoparvovirus A | Parvoviridae | 86.8 | 517 | 21698 | 4062 | 4680 | NC_001401 | Virosaurus | DNA library |
| 25181 | 3/9/2015 | 451_D | L007-r75 | Adeno-associated dependoparvovirus A | Parvoviridae | 50.98 | 2 | 55 | 2386 | 4680 | NC_001401 | Virosaurus | DNA library |
| 25289 | 4/10/2015 | 477_D | L005-r77 | Adeno-associated dependoparvovirus A | Parvoviridae | 34.66 | 1 | 31 | 1622 | 4680 | NC_001401 | Virosaurus | DNA library |
| 25396 | 4/28/2015 | 496_D | L003-r80 | Adeno-associated dependoparvovirus A | Parvoviridae | 6.69 | 1 | 4 | 313 | 4680 | NC_001401 | Virosaurus | DNA library |
| 25472 | 5/11/2015 | 516_D | L005-r84 | Adeno-associated dependoparvovirus A | Parvoviridae | 32.03 | 1 | 29 | 1499 | 4680 | NC_001401 | Virosaurus | DNA library |
| 25535 | 5/21/2015 | 526_D | L004-r80 | Adeno-associated dependoparvovirus A | Parvoviridae | 7.03 | 1 | 4 | 329 | 4680 | NC_001401 | Virosaurus | DNA library |
| 25634 | 6/3/2015 | 560_D | L6-r85 | Adeno-associated dependoparvovirus A | Parvoviridae | 75.47 | 3 | 146 | 3532 | 4680 | NC_001401 | Virosaurus | DNA library |
| 25657 | 6/5/2015 | 568_D | L7-r85 | Adeno-associated dependoparvovirus A | Parvoviridae | 66.5 | 2 | 102 | 3112 | 4680 | NC_001401 | Virosaurus | DNA library |
| 25689 | 6/10/2015 | 578_D | L2-r85 | Adeno-associated dependoparvovirus A | Parvoviridae | 99.66 | 36 | 1832 | 4664 | 4680 | NC_001401 | Virosaurus | DNA library |
| 25761 | 6/23/2015 | 601_D | L7-r88 | Adeno-associated dependoparvovirus A | Parvoviridae | 100 | 3437 | 166600 | 4680 | 4680 | NC_001401 | Virosaurus | DNA library |
| 25761 | 6/23/2015 | 601_R | L003-r723 | Adeno-associated dependoparvovirus A | Parvoviridae | 6.41 | 1 | 3 | 300 | 4680 | NC_001401 | Virosaurus | RNA library |
| 25783 | 6/25/2015 | 607_D | L2-r92 | Adeno-associated dependoparvovirus A | Parvoviridae | 100 | 5454 | 264370 | 4680 | 4680 | NC_001401 | Virosaurus | DNA library |
| 25783 | 6/25/2015 | 607_R | L005-r723 | Adeno-associated dependoparvovirus A | Parvoviridae | 13.12 | 1 | 8 | 614 | 4680 | NC_001401 | Virosaurus | RNA library |
| 25799 | 6/29/2015 | 609_D | L8-r88 | Adeno-associated dependoparvovirus A | Parvoviridae | 20.45 | 1 | 22 | 957 | 4680 | NC_001401 | Virosaurus | DNA library |
| 25886 | 7/10/2015 | 733_D | L5-r96 | Adeno-associated dependoparvovirus A | Parvoviridae | 98.1 | 11 | 579 | 4591 | 4680 | NC_001401 | Virosaurus | DNA library |
| 25923 | 7/16/2015 | 867_D | L3-r132 | Adeno-associated dependoparvovirus A | Parvoviridae | 21.28 | 1 | 13 | 996 | 4680 | NC_001401 | Virosaurus | DNA library |
| 25940 | 7/21/2015 | 741_D | L6-r96 | Adeno-associated dependoparvovirus A | Parvoviridae | 14.36 | 2 | 17 | 672 | 4680 | NC_001401 | Virosaurus | DNA library |
| 26004 | 8/3/2015 | 634_D | L4-r91 | Adeno-associated dependoparvovirus A | Parvoviridae | 100 | 334 | 18710 | 4680 | 4680 | NC_001401 | Virosaurus | DNA library |
| 26040 | 8/6/2015 | 638_D | L8-r91 | Adeno-associated dependoparvovirus A | Parvoviridae | 16.24 | 1 | 11 | 760 | 4680 | NC_001401 | Virosaurus | DNA library |
| 26141 | 8/25/2015 | 646_D | L1-r91 | Adeno-associated dependoparvovirus A | Parvoviridae | 80.92 | 11 | 522 | 3787 | 4680 | NC_001401 | Virosaurus | DNA library |
| 26210 | 9/2/2015 | 833_D | L4-r122 | Adeno-associated dependoparvovirus A | Parvoviridae | 54.25 | 1 | 60 | 2539 | 4680 | NC_001401 | Virosaurus | DNA library |
| 26224 | 9/4/2015 | 839_D | L7-r124 | Adeno-associated dependoparvovirus A | Parvoviridae | 100 | 1401 | 70062 | 4680 | 4680 | NC_001401 | Virosaurus | DNA library |
| 26224 | 9/4/2015 | 839_R | L6-r741 | Adeno-associated dependoparvovirus A | Parvoviridae | 14.49 | 1 | 11 | 678 | 4680 | NC_001401 | Virosaurus | RNA library |
| 26225 | 9/4/2015 | 840_D | L4-r122 | Adeno-associated dependoparvovirus A | Parvoviridae | 12.93 | 1 | 10 | 605 | 4680 | NC_001401 | Virosaurus | DNA library |
| 26229 | 9/4/2015 | 653_D | L1-r91 | Adeno-associated dependoparvovirus A | Parvoviridae | 26.03 | 1 | 24 | 1218 | 4680 | NC_001401 | Virosaurus | DNA library |
| 26254 | 9/8/2015 | 849_D | L6-r122 | Adeno-associated dependoparvovirus A | Parvoviridae | 99.79 | 26 | 1308 | 4670 | 4680 | NC_001401 | Virosaurus | DNA library |
| 26299 | 9/14/2015 | 880_D | L2-r132 | Adeno-associated dependoparvovirus A | Parvoviridae | 32.61 | 1 | 30 | 1526 | 4680 | NC_001401 | Virosaurus | DNA library |
| 26425 | 10/23/2015 | 9_D | L002-r44 | Adeno-associated dependoparvovirus A | Parvoviridae | 11.94 | 1 | 9 | 559 | 4680 | NC_001401 | Virosaurus | DNA library |
| 26479 | 11/10/2015 | 40_D | L004-r44 | Adeno-associated dependoparvovirus A | Parvoviridae | 25.86 | 1 | 18 | 1210 | 4680 | NC_001401 | Virosaurus | DNA library |
| 26487 | 11/11/2015 | 6_D | L006-r44 | Adeno-associated dependoparvovirus A | Parvoviridae | 87.42 | 4 | 209 | 4091 | 4680 | NC_001401 | Virosaurus | DNA library |
| 26558 | 12/4/2015 | 74_D | L008-r46 | Adeno-associated dependoparvovirus A | Parvoviridae | 97.05 | 6 | 357 | 4542 | 4680 | NC_001401 | Virosaurus | DNA library |
| 26140 | 8/25/2015 | 647_D | L3-r91 | Ambidensovirus_1 | Parvoviridae | 9.99 | 22 | 142 | 511 | 5116 | MN687954 | De novo | DNA library |
| 22453 | 11/16/2015 | 400_D | L004-r71 | Ambidensovirus_2 | Parvoviridae | 100 | 1615 | 72802 | 4575 | 4575 | MN765189 | De novo | DNA library |
| 22453 | 11/16/2015 | 400_R | L007-r703 | Ambidensovirus_2 | Parvoviridae | 10.49 | 6 | 27 | 480 | 4575 | MN765189 | De novo | RNA library |
| 21006 | 1/16/2015 | 92_D | L002-r49 | Ambidensovirus_3 | Parvoviridae | 39.88 | 5 | 117 | 1968 | 4935 | MN765190 | De novo | DNA library |
| 21077 | 2/19/2015 | 108_D | L004-r49 | Ambidensovirus_3 | Parvoviridae | 88.63 | 121 | 6024 | 4374 | 4935 | MN765190 | De novo | DNA library |
| 21528 | 6/5/2015 | 271_D | L1-r65 | Ambidensovirus_3 | Parvoviridae | 21.62 | 8 | 88 | 1067 | 4935 | MN765190 | De novo | DNA library |
| 21712 | 7/9/2015 | 301_D | L2-r65 | Ambidensovirus_3 | Parvoviridae | 37.97 | 5 | 93 | 1874 | 4935 | MN765190 | De novo | DNA library |
| 21766 | 7/21/2015 | 621_D | L2-r92 | Ambidensovirus_3 | Parvoviridae | 87.23 | 344 | 14785 | 4305 | 4935 | MN765190 | De novo | DNA library |
| 22049 | 9/2/2015 | 343_D | L7-r66 | Ambidensovirus_3 | Parvoviridae | 23.34 | 7 | 101 | 1152 | 4935 | MN765190 | De novo | DNA library |
| 22062 | 9/3/2015 | 345_D | L1-r66 | Ambidensovirus_3 | Parvoviridae | 6.97 | 6 | 22 | 344 | 4935 | MN765190 | De novo | DNA library |
| 22185 | 9/17/2015 | 359_D | L1-r66 | Ambidensovirus_3 | Parvoviridae | 6.32 | 1 | 15 | 312 | 4935 | MN765190 | De novo | DNA library |
| 22198 | 9/18/2015 | 363_D | L003-r71 | Ambidensovirus_3 | Parvoviridae | 42.57 | 31 | 898 | 2101 | 4935 | MN765190 | De novo | DNA library |
| 22259 | 9/29/2015 | 374_D | L003-r73 | Ambidensovirus_3 | Parvoviridae | 9.61 | 4 | 24 | 474 | 4935 | MN765190 | De novo | DNA library |
| 22401 | 10/21/2015 | 392_D | L003-r71 | Ambidensovirus_3 | Parvoviridae | 7.88 | 8 | 39 | 389 | 4935 | MN765190 | De novo | DNA library |
| 22419 | 10/29/2015 | 785_D | L6-r113 | Ambidensovirus_3 | Parvoviridae | 85.51 | 39 | 1744 | 4220 | 4935 | MN765190 | De novo | DNA library |
| 22432 | 11/4/2015 | 398_D | L002-r71 | Ambidensovirus_3 | Parvoviridae | 31.37 | 46 | 772 | 1548 | 4935 | MN765190 | De novo | DNA library |
| 22442 | 11/11/2015 | 23_D | L001-r44 | Ambidensovirus_3 | Parvoviridae | 9.93 | 7 | 41 | 490 | 4935 | MN765190 | De novo | DNA library |
| 22453 | 11/16/2015 | 400_D | L004-r71 | Ambidensovirus_3 | Parvoviridae | 19.94 | 4 | 42 | 984 | 4935 | MN765190 | De novo | DNA library |
| 25015 | 1/19/2015 | 410_D | L003-r75 | Ambidensovirus_3 | Parvoviridae | 99.27 | 25 | 1328 | 4899 | 4935 | MN765190 | De novo | DNA library |
| 25029 | 1/21/2015 | 795_D | L6-r111 | Ambidensovirus_3 | Parvoviridae | 15.83 | 14 | 160 | 781 | 4935 | MN765190 | De novo | DNA library |
| 25055 | 1/30/2015 | 418_D | L004-r75 | Ambidensovirus_3 | Parvoviridae | 61.05 | 6 | 204 | 3013 | 4935 | MN765190 | De novo | DNA library |
| 25099 | 2/17/2015 | 428_D | L005-r73 | Ambidensovirus_3 | Parvoviridae | 65.21 | 5 | 242 | 3218 | 4935 | MN765190 | De novo | DNA library |
| 25426 | 5/6/2015 | 503_D | L006-r84 | Ambidensovirus_3 | Parvoviridae | 29.24 | 8 | 125 | 1443 | 4935 | MN765190 | De novo | DNA library |
| 25690 | 6/10/2015 | 579_D | L3-r85 | Ambidensovirus_3 | Parvoviridae | 10.94 | 3 | 14 | 540 | 4935 | MN765190 | De novo | DNA library |
| 25761 | 6/23/2015 | 601_D | L7-r88 | Ambidensovirus_3 | Parvoviridae | 99.94 | 325 | 17620 | 4932 | 4935 | MN765190 | De novo | DNA library |
| 25799 | 6/29/2015 | 609_D | L8-r88 | Ambidensovirus_3 | Parvoviridae | 17.83 | 12 | 103 | 880 | 4935 | MN765190 | De novo | DNA library |
| 25837 | 7/2/2015 | 720_D | L4-r92 | Ambidensovirus_3 | Parvoviridae | 68.55 | 54 | 2505 | 3383 | 4935 | MN765190 | De novo | DNA library |
| 25954 | 7/23/2015 | 629_D | L7-r88 | Ambidensovirus_3 | Parvoviridae | 35.44 | 20 | 393 | 1749 | 4935 | MN765190 | De novo | DNA library |
| 25965 | 7/28/2015 | 631_D | L1-r91 | Ambidensovirus_3 | Parvoviridae | 77.81 | 10 | 399 | 3840 | 4935 | MN765190 | De novo | DNA library |
| 25995 | 7/30/2015 | 748_D | L6-r96 | Ambidensovirus_3 | Parvoviridae | 62.92 | 71 | 3865 | 3105 | 4935 | MN765190 | De novo | DNA library |
| 26089 | 8/14/2015 | 760_D | L8-r93 | Ambidensovirus_3 | Parvoviridae | 86.02 | 38 | 1864 | 4245 | 4935 | MN765190 | De novo | DNA library |
| 26108 | 8/18/2015 | 804_D | L8-r111 | Ambidensovirus_3 | Parvoviridae | 13.17 | 5 | 43 | 650 | 4935 | MN765190 | De novo | DNA library |
| 26163 | 8/27/2015 | 817_D | L6-r124 | Ambidensovirus_3 | Parvoviridae | 87.6 | 94 | 4180 | 4323 | 4935 | MN765190 | De novo | DNA library |
| 26174 | 8/27/2015 | 820_D | L6-r122 | Ambidensovirus_3 | Parvoviridae | 7.5 | 32 | 156 | 370 | 4935 | MN765190 | De novo | DNA library |
| 26178 | 8/28/2015 | 822_D | L8-r122 | Ambidensovirus_3 | Parvoviridae | 11.08 | 14 | 122 | 547 | 4935 | MN765190 | De novo | DNA library |
| 26186 | 8/31/2015 | 826_D | L4-r122 | Ambidensovirus_3 | Parvoviridae | 22.7 | 20 | 249 | 1120 | 4935 | MN765190 | De novo | DNA library |
| 26222 | 9/4/2015 | 838_D | L6-r124 | Ambidensovirus_3 | Parvoviridae | 99.98 | 1521 | 68640 | 4934 | 4935 | MN765190 | De novo | DNA library |
| 26224 | 9/4/2015 | 839_D | L7-r124 | Ambidensovirus_3 | Parvoviridae | 9.63 | 11 | 50 | 475 | 4935 | MN765190 | De novo | DNA library |
| 26225 | 9/4/2015 | 840_D | L4-r122 | Ambidensovirus_3 | Parvoviridae | 42.68 | 3 | 91 | 2106 | 4935 | MN765190 | De novo | DNA library |
| 26236 | 9/7/2015 | 842_D | L6-r122 | Ambidensovirus_3 | Parvoviridae | 98.83 | 73 | 3814 | 4877 | 4935 | MN765190 | De novo | DNA library |
| 26361 | 9/22/2015 | 900_D | L8-r127 | Ambidensovirus_3 | Parvoviridae | 56.23 | 6 | 271 | 2775 | 4935 | MN765190 | De novo | DNA library |
| 26436 | 10/27/2015 | 44_D | L001-r44 | Ambidensovirus_3 | Parvoviridae | 72.97 | 22 | 988 | 3601 | 4935 | MN765190 | De novo | DNA library |
| 26439 | 10/28/2015 | 2_D | L002-r44 | Ambidensovirus_3 | Parvoviridae | 10.13 | 10 | 50 | 500 | 4935 | MN765190 | De novo | DNA library |
| 26502 | 11/16/2015 | 673_D | L8-r91 | Ambidensovirus_3 | Parvoviridae | 84.48 | 15 | 785 | 4169 | 4935 | MN765190 | De novo | DNA library |
| 26503 | 11/16/2015 | 41_D | L006-r44 | Ambidensovirus_3 | Parvoviridae | 15.44 | 14 | 102 | 762 | 4935 | MN765190 | De novo | DNA library |
| 22196 | 9/18/2015 | 362_R | L006-r702 | Avian coronavirus | Coronaviridae | 1.7 | 1 | 6 | 471 | 27686 | KP118894 | Virosaurus | RNA library |
| 21058 | 2/10/2015 | 105_R | L004-r676 | Avian gyrovirus 2 | Anelloviridae | 13.76 | 1 | 4 | 328 | 2384 | NC_015396 | Virosaurus | RNA library |
| 22305 | 10/13/2015 | 386_D | L001-r73 | B19 | Parvoviridae | 99.49 | 550 | 32355 | 4908 | 4933 | KR005643 | Virosaurus | DNA library |
| 22319 | 10/20/2015 | 387_D | L002-r73 | B19 | Parvoviridae | 99.78 | 48152 | 2588973 | 4922 | 4933 | KR005643 | Virosaurus | DNA library |
| 22319 | 10/20/2015 | 387_R | L004-r703 | B19 | Parvoviridae | 97.69 | 51 | 2641 | 4819 | 4933 | KR005643 | Virosaurus | RNA library |
| 22436 | 11/6/2015 | 13_D | L006-r44 | B19 | Parvoviridae | 72.82 | 2 | 123 | 3592 | 4933 | KR005643 | Virosaurus | DNA library |
| 25965 | 7/28/2015 | 631_D | L1-r91 | B19 | Parvoviridae | 62.79 | 2 | 70 | 2799 | 4458 | KC013346 | Virosaurus | DNA library |
| 25969 | 7/27/2015 | 743_D | L5-r93 | B19 | Parvoviridae | 100 | 35599 | 2086282 | 5029 | 5029 | NC_004295 | Virosaurus | DNA library |
| 25969 | 7/27/2015 | 743_R | L4-r736 | B19 | Parvoviridae | 97.46 | 112 | 6561 | 4901 | 5029 | NC_004295 | Virosaurus | RNA library |
| 26250 | 9/8/2015 | 676_D | L3-r92 | B19 | Parvoviridae | 99.6 | 92 | 5255 | 4913 | 4933 | KR005643 | Virosaurus | DNA library |
| 26250 | 9/8/2015 | 676_R | L1-r734 | B19 | Parvoviridae | 16.02 | 2 | 14 | 714 | 4458 | KC013346 | Virosaurus | RNA library |
| 26446 | 10/29/2015 | 26_D | L005-r44 | B19 | Parvoviridae | 99.78 | 60230 | 3188152 | 4922 | 4933 | KR005643 | Virosaurus | DNA library |
| 26446 | 10/29/2015 | 26_R | L8-r669 | B19 | Parvoviridae | 96.09 | 64 | 4794 | 4740 | 4933 | KR005643 | Virosaurus | RNA library |
| 22062 | 9/3/2015 | 345_D | L1-r66 | Bat associated cyclovirus 16 | Circoviridae | 20.07 | 1 | 4 | 369 | 1839 | KT732787 | Virosaurus | DNA library |
| 22041 | 9/1/2015 | 342_D | L6-r66 | BK | Polyomaviridae | 48.21 | 1 | 43 | 2430 | 5040 | KP984526 | Virosaurus | DNA library |
| 21277 | 4/20/2015 | 221_R | L007-r690 | Bunya-like_virus_1 | Unknown | 100 | 335 | 21719 | 6226 | Na | MN727194 | De novo | RNA library |
| 21277 | 4/20/2015 | 221_R | L007-r690 | Bunya-like_virus_2 | Unknown | 100 | 478 | 29395 | 6092 | Na | MN727195 | De novo | RNA library |
| 21277 | 4/20/2015 | 221_R | L007-r690 | Bunya-like_virus_3 | Unknown | 100 | 171 | 9172 | 5268 | Na | MN727196 | De novo | RNA library |
| 26222 | 9/4/2015 | 838_R | L5-r741 | Cardiovirus B | Picornaviridae | 9.15 | 1 | 20 | 736 | 8046 | FN999911 | Virosaurus | RNA library |
| 26346 | 9/17/2015 | 895_R | L4-r746 | Cardiovirus B | Picornaviridae | 17.53 | 7 | 226 | 1400 | 7985 | HM181996 | Virosaurus | RNA library |
| 21534 | 6/5/2015 | 273_D | L3-r65 | Chicken stool-associated gemycircularvirus | Genomoviridae | 31.02 | 1 | 11 | 665 | 2144 | NC_033270 | Virosaurus | DNA library |
| 22190 | 9/18/2015 | 781_D | L6-r111 | Chicken stool-associated gemycircularvirus | Genomoviridae | 17.96 | 2 | 9 | 385 | 2144 | NC_033270 | Virosaurus | DNA library |
| 25425 | 5/6/2015 | 502_D | L005-r84 | CRESS_virus | Unknown | 99.05 | 106 | 2899 | 2596 | 2621 | MN765194 | De novo | DNA library |
| 25558 | 5/22/2015 | 529_D | L6-r82 | CRESS_virus | Unknown | 51.28 | 25 | 410 | 1344 | 2621 | MN765194 | De novo | DNA library |
| 25947 | 7/23/2015 | 630_D | L8-r96 | Cyclovirus ZM36a | Circoviridae | 16.08 | 1 | 3 | 300 | 1866 | NC_025476 | Virosaurus | DNA library |
| 26140 | 8/25/2015 | 647_D | L3-r91 | Cyclovirus ZM36a | Circoviridae | 49.41 | 3 | 44 | 922 | 1866 | NC_025476 | Virosaurus | DNA library |
| 21277 | 4/20/2015 | 221_R | L007-r690 | Dengue virus [Serotype 2] | Flaviviridae | 7.03 | 1 | 10 | 769 | 10941 | KX452017 | Virosaurus | RNA library |
| 21461 | 6/2/2015 | 858_R | L3-r745 | Dicistro-like_virus_1 | Unknown | 100 | 517 | 48327 | 9589 | 9589 | MN727201 | De novo | RNA library |
| 21534 | 6/5/2015 | 273_R | L006-r692 | Dicistro-like_virus_1 | Unknown | 6.85 | 3 | 31 | 657 | 9589 | MN727201 | De novo | RNA library |
| 21837 | 8/3/2015 | 315_R | L001-r698 | Dicistro-like_virus_1 | Unknown | 4.44 | 4 | 23 | 426 | 9589 | MN727201 | De novo | RNA library |
| 25639 | 6/3/2015 | 563_R | L7-r717 | Dicistro-like_virus_1 | Unknown | 5.85 | 4 | 26 | 561 | 9589 | MN727201 | De novo | RNA library |
| 25729 | 6/17/2015 | 685_R | L3-r734 | Dicistro-like_virus_1 | Unknown | 81.94 | 6 | 628 | 7857 | 9589 | MN727201 | De novo | RNA library |
| 25731 | 6/17/2015 | 687_R | L3-r734 | Dicistro-like_virus_1 | Unknown | 6.07 | 5 | 29 | 582 | 9589 | MN727201 | De novo | RNA library |
| 26046 | 8/7/2015 | 753_R | L7-r736 | Dicistro-like_virus_1 | Unknown | 11.7 | 7 | 69 | 1122 | 9589 | MN727201 | De novo | RNA library |
| 26182 | 8/28/2015 | 825_R | L2-r741 | Dicistro-like_virus_1 | Unknown | 6.97 | 5 | 34 | 668 | 9589 | MN727201 | De novo | RNA library |
| 26353 | 9/21/2015 | 897_R | L5-r746 | Dicistro-like_virus_1 | Unknown | 10.56 | 7 | 75 | 1013 | 9589 | MN727201 | De novo | RNA library |
| 26513 | 11/19/2015 | 17_R | L5-r669 | Dicistro-like_virus_1 | Unknown | 16.09 | 2 | 36 | 1543 | 9589 | MN727201 | De novo | RNA library |
| 21060 | 2/13/2015 | 106_R | L004-r676 | Dicistro-like_virus_2 | Unknown | 17.73 | 6 | 128 | 1664 | 9388 | MN727202 | De novo | RNA library |
| 25942 | 7/21/2015 | 868_R | L6-r745 | Dicistro-like_virus_2 | Unknown | 100 | 16659 | 1558879 | 9388 | 9388 | MN727202 | De novo | RNA library |
| 21004 | 12/12/2014 | 91_R | L001-r676 | Dicistrovirus | Dicistroviridae | 8.28 | 1 | 13 | 766 | 9251 | MH536111 | De novo | RNA library |
| 21006 | 1/16/2015 | 92_R | L001-r676 | Dicistrovirus | Dicistroviridae | 27.63 | 3 | 97 | 2556 | 9251 | MH536111 | De novo | RNA library |
| 21017 | 1/23/2015 | 766_R | L3-r737 | Dicistrovirus | Dicistroviridae | 9.22 | 4 | 38 | 853 | 9251 | MH536111 | De novo | RNA library |
| 21021 | 1/26/2015 | 96_R | L002-r676 | Dicistrovirus | Dicistroviridae | 99.74 | 32 | 3083 | 9227 | 9251 | MH536111 | De novo | RNA library |
| 21026 | 1/28/2015 | 97_R | L002-r676 | Dicistrovirus | Dicistroviridae | 99.94 | 30 | 3263 | 9245 | 9251 | MH536111 | De novo | RNA library |
| 21028 | 1/28/2015 | 796_R | L2-r740 | Dicistrovirus | Dicistroviridae | 21.67 | 2 | 48 | 2005 | 9251 | MH536111 | De novo | RNA library |
| 21031 | 1/29/2015 | 586_R | L006-r722 | Dicistrovirus | Dicistroviridae | 94.62 | 21 | 2000 | 8753 | 9251 | MH536111 | De novo | RNA library |
| 21039 | 2/2/2015 | 99_R | L003-r676 | Dicistrovirus | Dicistroviridae | 98.58 | 32 | 3223 | 9120 | 9251 | MH536111 | De novo | RNA library |
| 21042 | 2/3/2015 | 101_R | L003-r676 | Dicistrovirus | Dicistroviridae | 99.98 | 750 | 73799 | 9249 | 9251 | MH536111 | De novo | RNA library |
| 21053 | 2/6/2015 | 104_R | L004-r676 | Dicistrovirus | Dicistroviridae | 100 | 1272 | 118892 | 9251 | 9251 | MH536111 | De novo | RNA library |
| 21080 | 2/20/2015 | 109_R | L005-r676 | Dicistrovirus | Dicistroviridae | 55.94 | 5 | 305 | 5175 | 9251 | MH536111 | De novo | RNA library |
| 21092 | 2/27/2015 | 114_R | L006-r676 | Dicistrovirus | Dicistroviridae | 59.65 | 6 | 395 | 5518 | 9251 | MH536111 | De novo | RNA library |
| 21102 | 2/27/2015 | 118_R | L7-r735 | Dicistrovirus | Dicistroviridae | 5.77 | 4 | 22 | 534 | 9251 | MH536111 | De novo | RNA library |
| 21158 | 3/17/2015 | 127_R | L002-r677 | Dicistrovirus | Dicistroviridae | 98.62 | 18 | 1792 | 9123 | 9251 | MH536111 | De novo | RNA library |
| 21220 | 3/30/2015 | 213_R | L005-r690 | Dicistrovirus | Dicistroviridae | 70.41 | 9 | 1164 | 6514 | 9251 | MH536111 | De novo | RNA library |
| 21247 | 4/10/2015 | 215_R | L005-r690 | Dicistrovirus | Dicistroviridae | 96.73 | 34 | 4659 | 8948 | 9251 | MH536111 | De novo | RNA library |
| 21291 | 4/27/2015 | 223_R | L007-r690 | Dicistrovirus | Dicistroviridae | 86.4 | 19 | 2046 | 7993 | 9251 | MH536111 | De novo | RNA library |
| 21332 | 5/11/2015 | 228_R | L002-r691 | Dicistrovirus | Dicistroviridae | 99.94 | 66 | 6093 | 9245 | 9251 | MH536111 | De novo | RNA library |
| 21333 | 5/11/2015 | 229_R | L002-r691 | Dicistrovirus | Dicistroviridae | 87.62 | 50 | 4893 | 8106 | 9251 | MH536111 | De novo | RNA library |
| 21424 | 5/22/2015 | 245_R | L006-r691 | Dicistrovirus | Dicistroviridae | 9.52 | 3 | 37 | 881 | 9251 | MH536111 | De novo | RNA library |
| 21518 | 6/3/2015 | 266_R | L004-r692 | Dicistrovirus | Dicistroviridae | 5.22 | 3 | 18 | 483 | 9251 | MH536111 | De novo | RNA library |
| 21526 | 6/4/2015 | 270_R | L005-r692 | Dicistrovirus | Dicistroviridae | 14.56 | 1 | 23 | 1347 | 9251 | MH536111 | De novo | RNA library |
| 21571 | 6/15/2015 | 280_R | L008-r692 | Dicistrovirus | Dicistroviridae | 32.92 | 6 | 237 | 3045 | 9251 | MH536111 | De novo | RNA library |
| 21573 | 6/15/2015 | 281_R | L008-r692 | Dicistrovirus | Dicistroviridae | 52.99 | 4 | 271 | 4902 | 9251 | MH536111 | De novo | RNA library |
| 21601 | 6/18/2015 | 284_R | L001-r693 | Dicistrovirus | Dicistroviridae | 28.31 | 6 | 198 | 2619 | 9251 | MH536111 | De novo | RNA library |
| 21606 | 6/22/2015 | 598_R | L003-r723 | Dicistrovirus | Dicistroviridae | 9.8 | 24 | 303 | 907 | 9251 | MH536111 | De novo | RNA library |
| 21650 | 6/30/2015 | 293_R | L003-r693 | Dicistrovirus | Dicistroviridae | 4.89 | 5 | 21 | 452 | 9251 | MH536111 | De novo | RNA library |
| 21701 | 7/9/2015 | 298_R | L004-r693 | Dicistrovirus | Dicistroviridae | 41.7 | 5 | 252 | 3858 | 9251 | MH536111 | De novo | RNA library |
| 21938 | 8/18/2015 | 331_R | L005-r698 | Dicistrovirus | Dicistroviridae | 11.88 | 7 | 78 | 1099 | 9251 | MH536111 | De novo | RNA library |
| 22211 | 9/22/2015 | 367_R | L007-r702 | Dicistrovirus | Dicistroviridae | 49.68 | 6 | 338 | 4596 | 9251 | MH536111 | De novo | RNA library |
| 22212 | 9/22/2015 | 368_R | L007-r702 | Dicistrovirus | Dicistroviridae | 98.71 | 43 | 5664 | 9132 | 9251 | MH536111 | De novo | RNA library |
| 22215 | 9/22/2015 | 369_R | L008-r702 | Dicistrovirus | Dicistroviridae | 97.14 | 43 | 5239 | 8986 | 9251 | MH536111 | De novo | RNA library |
| 22276 | 10/9/2015 | 377_R | L002-r703 | Dicistrovirus | Dicistroviridae | 48.84 | 9 | 665 | 4518 | 9251 | MH536111 | De novo | RNA library |
| 22285 | 10/15/2015 | 378_R | L002-r703 | Dicistrovirus | Dicistroviridae | 93.08 | 15 | 1417 | 8611 | 9251 | MH536111 | De novo | RNA library |
| 22292 | 10/19/2015 | 380_R | L002-r703 | Dicistrovirus | Dicistroviridae | 9.05 | 10 | 97 | 837 | 9251 | MH536111 | De novo | RNA library |
| 22295 | 10/20/2015 | 382_R | L003-r703 | Dicistrovirus | Dicistroviridae | 28.89 | 6 | 253 | 2673 | 9251 | MH536111 | De novo | RNA library |
| 22405 | 10/22/2015 | 393_R | L006-r703 | Dicistrovirus | Dicistroviridae | 30.88 | 14 | 427 | 2857 | 9251 | MH536111 | De novo | RNA library |
| 22415 | 10/28/2015 | 10_R | L2-r669 | Dicistrovirus | Dicistroviridae | 82.9 | 4 | 383 | 7669 | 9251 | MH536111 | De novo | RNA library |
| 22418 | 10/29/2015 | 668_R | L5-r725 | Dicistrovirus | Dicistroviridae | 97.96 | 69 | 6832 | 9062 | 9251 | MH536111 | De novo | RNA library |
| 22419 | 10/29/2015 | 785_R | L7-r737 | Dicistrovirus | Dicistroviridae | 99.63 | 54 | 7565 | 9217 | 9251 | MH536111 | De novo | RNA library |
| 22432 | 11/4/2015 | 398_R | L007-r703 | Dicistrovirus | Dicistroviridae | 98.2 | 80 | 13483 | 9084 | 9251 | MH536111 | De novo | RNA library |
| 22442 | 11/11/2015 | 23_R | L6-r669 | Dicistrovirus | Dicistroviridae | 28.79 | 2 | 81 | 2663 | 9251 | MH536111 | De novo | RNA library |
| 22453 | 11/16/2015 | 400_R | L007-r703 | Dicistrovirus | Dicistroviridae | 82.41 | 12 | 1357 | 7624 | 9251 | MH536111 | De novo | RNA library |
| 22460 | 11/17/2015 | 16_R | L4-r669 | Dicistrovirus | Dicistroviridae | 85.32 | 16 | 1663 | 7893 | 9251 | MH536111 | De novo | RNA library |
| 22461 | 11/17/2015 | 24_R | L4-r669 | Dicistrovirus | Dicistroviridae | 99.38 | 42 | 5669 | 9194 | 9251 | MH536111 | De novo | RNA library |
| 22473 | 11/23/2015 | 402_R | L008-r703 | Dicistrovirus | Dicistroviridae | 11.15 | 10 | 122 | 1031 | 9251 | MH536111 | De novo | RNA library |
| 22481 | 11/24/2015 | 662_R | L3-r725 | Dicistrovirus | Dicistroviridae | 32.19 | 14 | 490 | 2978 | 9251 | MH536111 | De novo | RNA library |
| 22485 | 11/26/2015 | 53_R | L006-r674 | Dicistrovirus | Dicistroviridae | 82.23 | 14 | 1204 | 7607 | 9251 | MH536111 | De novo | RNA library |
| 22486 | 8/6/2015 | 59_R | L008-r674 | Dicistrovirus | Dicistroviridae | 16.61 | 4 | 158 | 1537 | 9251 | MH536111 | De novo | RNA library |
| 22495 | 11/24/2015 | 403_R | L008-r703 | Dicistrovirus | Dicistroviridae | 11.86 | 6 | 79 | 1097 | 9251 | MH536111 | De novo | RNA library |
| 22606 | 12/4/2015 | 404_R | L008-r703 | Dicistrovirus | Dicistroviridae | 64.23 | 12 | 1117 | 5942 | 9251 | MH536111 | De novo | RNA library |
| 22624 | 1/7/2016 | 75_R | L004-r675 | Dicistrovirus | Dicistroviridae | 55.78 | 13 | 876 | 5160 | 9251 | MH536111 | De novo | RNA library |
| 22653 | 1/21/2016 | 73_R | L003-r675 | Dicistrovirus | Dicistroviridae | 59.26 | 15 | 1846 | 5482 | 9251 | MH536111 | De novo | RNA library |
| 22671 | 1/29/2016 | 406_R | L002-r704 | Dicistrovirus | Dicistroviridae | 3.33 | 3 | 8 | 308 | 9251 | MH536111 | De novo | RNA library |
| 22672 | 2/1/2016 | 666_R | L4-r725 | Dicistrovirus | Dicistroviridae | 3.91 | 10 | 45 | 362 | 9251 | MH536111 | De novo | RNA library |
| 22673 | 2/1/2016 | 90_R | L008-r675 | Dicistrovirus | Dicistroviridae | 95.33 | 107 | 9604 | 8819 | 9251 | MH536111 | De novo | RNA library |
| 22680 | 2/3/2016 | 76_R | L004-r675 | Dicistrovirus | Dicistroviridae | 12.3 | 11 | 127 | 1138 | 9251 | MH536111 | De novo | RNA library |
| 22686 | 2/9/2016 | 63_R | L001-r675 | Dicistrovirus | Dicistroviridae | 6.29 | 3 | 24 | 582 | 9251 | MH536111 | De novo | RNA library |
| 25008 | 1/15/2015 | 409_R | L002-r704 | Dicistrovirus | Dicistroviridae | 49.77 | 10 | 593 | 4604 | 9251 | MH536111 | De novo | RNA library |
| 25015 | 1/19/2015 | 410_R | L003-r704 | Dicistrovirus | Dicistroviridae | 22.82 | 13 | 298 | 2111 | 9251 | MH536111 | De novo | RNA library |
| 25025 | 1/21/2015 | 413_R | L003-r704 | Dicistrovirus | Dicistroviridae | 91.42 | 7 | 723 | 8457 | 9251 | MH536111 | De novo | RNA library |
| 25026 | 1/21/2015 | 414_R | L004-r704 | Dicistrovirus | Dicistroviridae | 3.99 | 18 | 103 | 369 | 9251 | MH536111 | De novo | RNA library |
| 25029 | 1/21/2015 | 795_R | L2-r740 | Dicistrovirus | Dicistroviridae | 8.77 | 2 | 54 | 811 | 9251 | MH536111 | De novo | RNA library |
| 25034 | 1/22/2015 | 416_R | L004-r704 | Dicistrovirus | Dicistroviridae | 7.26 | 14 | 116 | 672 | 9251 | MH536111 | De novo | RNA library |
| 25055 | 1/30/2015 | 418_R | L005-r704 | Dicistrovirus | Dicistroviridae | 28.74 | 40 | 1079 | 2659 | 9251 | MH536111 | De novo | RNA library |
| 25056 | 1/30/2015 | 419_R | L005-r704 | Dicistrovirus | Dicistroviridae | 84.64 | 44 | 3921 | 7830 | 9251 | MH536111 | De novo | RNA library |
| 25083 | 2/11/2015 | 421_R | L005-r704 | Dicistrovirus | Dicistroviridae | 16.53 | 14 | 275 | 1529 | 9251 | MH536111 | De novo | RNA library |
| 25085 | 2/12/2015 | 422_R | L006-r704 | Dicistrovirus | Dicistroviridae | 4.56 | 13 | 64 | 422 | 9251 | MH536111 | De novo | RNA library |
| 25160 | 3/5/2015 | 444_R | L003-r705 | Dicistrovirus | Dicistroviridae | 18.79 | 14 | 276 | 1738 | 9251 | MH536111 | De novo | RNA library |
| 25161 | 3/5/2015 | 587_R | L006-r722 | Dicistrovirus | Dicistroviridae | 54.52 | 9 | 584 | 5044 | 9251 | MH536111 | De novo | RNA library |
| 25163 | 3/5/2015 | 446_R | L004-r705 | Dicistrovirus | Dicistroviridae | 5.75 | 14 | 76 | 532 | 9251 | MH536111 | De novo | RNA library |
| 25181 | 3/9/2015 | 451_R | L006-r705 | Dicistrovirus | Dicistroviridae | 22.24 | 8 | 286 | 2057 | 9251 | MH536111 | De novo | RNA library |
| 25283 | 4/10/2015 | 475_R | L8-r707 | Dicistrovirus | Dicistroviridae | 99.96 | 237 | 23881 | 9247 | 9251 | MH536111 | De novo | RNA library |
| 25329 | 4/16/2015 | 484_R | L2-r713 | Dicistrovirus | Dicistroviridae | 3.5 | 2 | 8 | 324 | 9251 | MH536111 | De novo | RNA library |
| 25355 | 4/21/2015 | 489_R | L3-r713 | Dicistrovirus | Dicistroviridae | 6.69 | 14 | 72 | 619 | 9251 | MH536111 | De novo | RNA library |
| 25599 | 5/28/2015 | 543_R | L2-r717 | Dicistrovirus | Dicistroviridae | 6.25 | 4 | 46 | 578 | 9251 | MH536111 | De novo | RNA library |
| 25766 | 6/24/2015 | 696_R | L6-r734 | Dicistrovirus | Dicistroviridae | 79.51 | 18 | 1453 | 7355 | 9251 | MH536111 | De novo | RNA library |
| 25772 | 6/24/2015 | 697_R | L6-r734 | Dicistrovirus | Dicistroviridae | 6 | 5 | 29 | 555 | 9251 | MH536111 | De novo | RNA library |
| 25938 | 7/21/2015 | 739_R | L3-r736 | Dicistrovirus | Dicistroviridae | 3.49 | 2 | 6 | 323 | 9251 | MH536111 | De novo | RNA library |
| 26134 | 8/24/2015 | 809_R | L5-r740 | Dicistrovirus | Dicistroviridae | 3.98 | 2 | 9 | 368 | 9251 | MH536111 | De novo | RNA library |
| 26493 | 11/12/2015 | 14_R | L5-r669 | Dicistrovirus | Dicistroviridae | 28.34 | 1 | 38 | 2622 | 9251 | MH536111 | De novo | RNA library |
| 26502 | 11/16/2015 | 673_R | L6-r725 | Dicistrovirus | Dicistroviridae | 95.96 | 41 | 4547 | 8877 | 9251 | MH536111 | De novo | RNA library |
| 26503 | 11/16/2015 | 41_R | L004-r674 | Dicistrovirus | Dicistroviridae | 90.91 | 8 | 1089 | 8410 | 9251 | MH536111 | De novo | RNA library |
| 26513 | 11/19/2015 | 17_R | L5-r669 | Dicistrovirus | Dicistroviridae | 3.98 | 2 | 6 | 368 | 9251 | MH536111 | De novo | RNA library |
| 21077 | 2/19/2015 | 108_R | L005-r676 | Enterovirus A | Picornaviridae | 10.27 | 14 | 468 | 762 | 7418 | KX372322 | Virosaurus | RNA library |
| 21105 | 3/2/2015 | 120_R | L008-r676 | Enterovirus A | Picornaviridae | 15.97 | 5 | 79 | 1178 | 7376 | MF422542 | Virosaurus | RNA library |
| 21175 | 3/16/2015 | 130_R | L002-r677 | Enterovirus A | Picornaviridae | 5.97 | 3 | 14 | 446 | 7469 | KP676985 | Virosaurus | RNA library |
| 21294 | 5/5/2015 | 224_R | L007-r690 | Enterovirus A | Picornaviridae | 5.42 | 1 | 4 | 400 | 7379 | KY271949 | Virosaurus | RNA library |
| 21493 | 5/29/2015 | 261_R | L002-r692 | Enterovirus A | Picornaviridae | 74.65 | 33 | 2441 | 5529 | 7407 | HQ728261 | Virosaurus | RNA library |
| 21533 | 6/5/2015 | 272_R | L006-r692 | Enterovirus A | Picornaviridae | 45.2 | 13 | 781 | 3335 | 7379 | KY271949 | Virosaurus | RNA library |
| 22001 | 8/26/2015 | 339_R | L007-r698 | Enterovirus A | Picornaviridae | 43.08 | 113 | 5316 | 3191 | 7407 | HQ728261 | Virosaurus | RNA library |
| 22107 | 9/9/2015 | 352_R | L003-r702 | Enterovirus A | Picornaviridae | 44.04 | 19 | 1456 | 3250 | 7379 | KY271949 | Virosaurus | RNA library |
| 22108 | 9/9/2015 | 353_R | L003-r702 | Enterovirus A | Picornaviridae | 44.74 | 13 | 895 | 3301 | 7379 | KY271949 | Virosaurus | RNA library |
| 22163 | 9/15/2015 | 357_R | L004-r702 | Enterovirus A | Picornaviridae | 9.45 | 1 | 7 | 700 | 7411 | DQ452074 | Virosaurus | RNA library |
| 22185 | 9/17/2015 | 359_R | L004-r702 | Enterovirus A | Picornaviridae | 10.36 | 12 | 106 | 764 | 7376 | MF422542 | Virosaurus | RNA library |
| 22652 | 1/20/2016 | 66_R | L002-r675 | Enterovirus A | Picornaviridae | 4.43 | 1 | 4 | 328 | 7411 | DQ452074 | Virosaurus | RNA library |
| 22663 | 1/27/2016 | 57_R | L007-r674 | Enterovirus A | Picornaviridae | 8.77 | 1 | 19 | 652 | 7435 | AB779617 | Virosaurus | RNA library |
| 25037 | 1/23/2015 | 417_R | L004-r704 | Enterovirus A | Picornaviridae | 8.54 | 1 | 12 | 630 | 7376 | MF422542 | Virosaurus | RNA library |
| 25216 | 3/19/2015 | 461_R | L008-r705 | Enterovirus A | Picornaviridae | 5.9 | 1 | 6 | 437 | 7411 | DQ452074 | Virosaurus | RNA library |
| 25222 | 3/20/2015 | 464_R | L5-r707 | Enterovirus A | Picornaviridae | 41.52 | 9 | 325 | 3075 | 7407 | HQ728261 | Virosaurus | RNA library |
| 25353 | 4/21/2015 | 488_R | L3-r713 | Enterovirus A | Picornaviridae | 12.87 | 13 | 535 | 955 | 7418 | KX372322 | Virosaurus | RNA library |
| 25361 | 4/22/2015 | 490_R | L3-r713 | Enterovirus A | Picornaviridae | 11.08 | 30 | 910 | 822 | 7418 | KX372322 | Virosaurus | RNA library |
| 25396 | 4/28/2015 | 496_R | L6-r713 | Enterovirus A | Picornaviridae | 51.57 | 49 | 5449 | 3805 | 7379 | KY271949 | Virosaurus | RNA library |
| 25426 | 5/6/2015 | 503_R | L7-r713 | Enterovirus A | Picornaviridae | 9.74 | 3 | 36 | 717 | 7364 | KX810065 | Virosaurus | RNA library |
| 25432 | 5/6/2015 | 504_R | L8-r713 | Enterovirus A | Picornaviridae | 40.43 | 14 | 668 | 2983 | 7379 | KY271949 | Virosaurus | RNA library |
| 25446 | 5/8/2015 | 508_R | L1-r714 | Enterovirus A | Picornaviridae | 28.36 | 14 | 644 | 2113 | 7450 | KP289442 | Virosaurus | RNA library |
| 25467 | 5/12/2015 | 513_R | L2-r714 | Enterovirus A | Picornaviridae | 4.89 | 2 | 7 | 361 | 7376 | MF422542 | Virosaurus | RNA library |
| 25676 | 6/9/2015 | 572_R | L001-r722 | Enterovirus A | Picornaviridae | 13.81 | 8 | 1300 | 1019 | 7379 | KY271949 | Virosaurus | RNA library |
| 25772 | 6/24/2015 | 697_R | L6-r734 | Enterovirus A | Picornaviridae | 48.19 | 26 | 3358 | 3556 | 7379 | KY271949 | Virosaurus | RNA library |
| 25784 | 6/25/2015 | 701_R | L7-r734 | Enterovirus A | Picornaviridae | 16.5 | 10 | 201 | 1217 | 7376 | MF422542 | Virosaurus | RNA library |
| 25826 | 7/1/2015 | 716_R | L3-r735 | Enterovirus A | Picornaviridae | 44.14 | 22 | 1798 | 3257 | 7379 | KY271949 | Virosaurus | RNA library |
| 25923 | 7/16/2015 | 867_R | L5-r745 | Enterovirus A | Picornaviridae | 94.03 | 207 | 18073 | 7167 | 7622 | KR815992 | Virosaurus | RNA library |
| 26074 | 8/10/2015 | 780_R | L6-r737 | Enterovirus A | Picornaviridae | 8.18 | 7 | 62 | 603 | 7376 | MF422542 | Virosaurus | RNA library |
| 26089 | 8/14/2015 | 760_R | L8-r736 | Enterovirus A | Picornaviridae | 10.34 | 8 | 107 | 767 | 7418 | KX372322 | Virosaurus | RNA library |
| 26102 | 8/18/2015 | 764_R | L1-r737 | Enterovirus A | Picornaviridae | 43.35 | 12 | 735 | 3199 | 7379 | KY271949 | Virosaurus | RNA library |
| 26104 | 8/18/2015 | 765_R | L2-r737 | Enterovirus A | Picornaviridae | 4.42 | 1 | 5 | 326 | 7376 | MF422542 | Virosaurus | RNA library |
| 26165 | 8/26/2015 | 818_R | L8-r740 | Enterovirus A | Picornaviridae | 95.47 | 1411 | 122018 | 7277 | 7622 | KR815992 | Virosaurus | RNA library |
| 26181 | 8/28/2015 | 824_R | L2-r741 | Enterovirus A | Picornaviridae | 8.87 | 1 | 11 | 654 | 7376 | MF422542 | Virosaurus | RNA library |
| 26208 | 9/2/2015 | 652_R | L1-r725 | Enterovirus A | Picornaviridae | 23.5 | 2 | 53 | 1734 | 7379 | KY271949 | Virosaurus | RNA library |
| 26251 | 9/8/2015 | 847_R | L8-r741 | Enterovirus A | Picornaviridae | 70.87 | 20 | 1573 | 5249 | 7407 | HQ728261 | Virosaurus | RNA library |
| 26340 | 9/17/2015 | 892_R | L4-r746 | Enterovirus A | Picornaviridae | 11.15 | 3 | 24 | 823 | 7379 | KY271949 | Virosaurus | RNA library |
| 26462 | 11/3/2015 | 12_R | L1-r669 | Enterovirus A | Picornaviridae | 36.54 | 7 | 285 | 2696 | 7379 | KY271949 | Virosaurus | RNA library |
| 26483 | 11/10/2015 | 21_R | L6-r669 | Enterovirus A | Picornaviridae | 9.28 | 4 | 36 | 685 | 7379 | KY271949 | Virosaurus | RNA library |
| 21183 | 3/13/2015 | 134_R | L003-r677 | Enterovirus B | Picornaviridae | 6.02 | 1 | 5 | 420 | 6975 | KJ701248 | Virosaurus | RNA library |
| 21184 | 3/13/2015 | 135_R | L004-r677 | Enterovirus B | Picornaviridae | 7.53 | 1 | 10 | 545 | 7241 | MF678296 | Virosaurus | RNA library |
| 21609 | 6/22/2015 | 287_R | L002-r693 | Enterovirus B | Picornaviridae | 6.75 | 1 | 6 | 456 | 6759 | MG451803 | Virosaurus | RNA library |
| 21610 | 6/22/2015 | 288_R | L002-r693 | Enterovirus B | Picornaviridae | 5.4 | 1 | 5 | 365 | 6759 | MG451803 | Virosaurus | RNA library |
| 22293 | 10/19/2015 | 381_R | L003-r703 | Enterovirus B | Picornaviridae | 5.92 | 1 | 4 | 400 | 6759 | MG451803 | Virosaurus | RNA library |
| 22295 | 10/20/2015 | 382_R | L003-r703 | Enterovirus B | Picornaviridae | 30.43 | 1 | 28 | 2057 | 6759 | MG451803 | Virosaurus | RNA library |
| 22359 | 11/6/2015 | 390_R | L005-r703 | Enterovirus B | Picornaviridae | 5.64 | 1 | 5 | 381 | 6759 | MG451803 | Virosaurus | RNA library |
| 22435 | 11/6/2015 | 39_R | L002-r674 | Enterovirus B | Picornaviridae | 45.16 | 2 | 62 | 3052 | 6759 | MG451803 | Virosaurus | RNA library |
| 22442 | 11/11/2015 | 23_R | L6-r669 | Enterovirus B | Picornaviridae | 33.75 | 1 | 32 | 2281 | 6759 | MG451803 | Virosaurus | RNA library |
| 22444 | 11/11/2015 | 29_R | L8-r669 | Enterovirus B | Picornaviridae | 87.97 | 30 | 2066 | 5946 | 6759 | MG451803 | Virosaurus | RNA library |
| 22606 | 12/4/2015 | 404_R | L008-r703 | Enterovirus B | Picornaviridae | 6.87 | 1 | 5 | 464 | 6759 | MG451803 | Virosaurus | RNA library |
| 22623 | 1/7/2016 | 79_R | L005-r675 | Enterovirus B | Picornaviridae | 8.95 | 1 | 7 | 605 | 6759 | MG451803 | Virosaurus | RNA library |
| 22667 | 3/20/2015 | 62_R | L001-r675 | Enterovirus B | Picornaviridae | 8.26 | 1 | 7 | 558 | 6759 | MG451803 | Virosaurus | RNA library |
| 25117 | 2/20/2015 | 435_R | L001-r705 | Enterovirus B | Picornaviridae | 10.28 | 1 | 11 | 695 | 6759 | MG451803 | Virosaurus | RNA library |
| 25598 | 5/28/2015 | 542_R | L2-r717 | Enterovirus B | Picornaviridae | 19.25 | 1 | 25 | 1418 | 7365 | KX810066 | Virosaurus | RNA library |
| 25654 | 6/5/2015 | 566_R | L8-r717 | Enterovirus B | Picornaviridae | 4.68 | 1 | 5 | 316 | 6759 | MG451803 | Virosaurus | RNA library |
| 25721 | 6/16/2015 | 682_R | L2-r734 | Enterovirus B | Picornaviridae | 17.29 | 2 | 38 | 1252 | 7241 | MF678296 | Virosaurus | RNA library |
| 25722 | 6/16/2015 | 683_R | L2-r734 | Enterovirus B | Picornaviridae | 4.95 | 1 | 5 | 345 | 6975 | KJ701248 | Virosaurus | RNA library |
| 25729 | 6/17/2015 | 685_R | L3-r734 | Enterovirus B | Picornaviridae | 4.52 | 1 | 5 | 327 | 7241 | MF678296 | Virosaurus | RNA library |
| 25801 | 6/29/2015 | 608_R | L005-r723 | Enterovirus B | Picornaviridae | 18.56 | 4 | 186 | 1344 | 7241 | MF678296 | Virosaurus | RNA library |
| 25895 | 7/13/2015 | 735_R | L2-r736 | Enterovirus B | Picornaviridae | 13.24 | 4 | 52 | 959 | 7241 | MF678296 | Virosaurus | RNA library |
| 26046 | 8/7/2015 | 753_R | L7-r736 | Enterovirus B | Picornaviridae | 12.97 | 5 | 87 | 947 | 7304 | MG451804 | Virosaurus | RNA library |
| 26130 | 8/24/2015 | 808_R | L5-r740 | Enterovirus B | Picornaviridae | 19.65 | 8 | 124 | 1328 | 6759 | MG451803 | Virosaurus | RNA library |
| 26222 | 9/4/2015 | 838_R | L5-r741 | Enterovirus B | Picornaviridae | 17.06 | 1 | 19 | 1153 | 6759 | MG451803 | Virosaurus | RNA library |
| 26333 | 9/17/2015 | 670_R | L5-r725 | Enterovirus B | Picornaviridae | 9.6 | 1 | 14 | 701 | 7304 | MG451804 | Virosaurus | RNA library |
| 26346 | 9/17/2015 | 895_R | L4-r746 | Enterovirus B | Picornaviridae | 4.85 | 2 | 6 | 328 | 6759 | MG451803 | Virosaurus | RNA library |
| 26351 | 9/21/2015 | 896_R | L5-r746 | Enterovirus B | Picornaviridae | 8.51 | 1 | 12 | 575 | 6759 | MG451803 | Virosaurus | RNA library |
| 26498 | 11/13/2015 | 34_R | L8-r669 | Enterovirus B | Picornaviridae | 51.55 | 2 | 80 | 3484 | 6759 | MG451803 | Virosaurus | RNA library |
| 26503 | 11/16/2015 | 41_R | L004-r674 | Enterovirus B | Picornaviridae | 7.13 | 1 | 6 | 492 | 6898 | MF678340 | Virosaurus | RNA library |
| 26513 | 11/19/2015 | 17_R | L5-r669 | Enterovirus B | Picornaviridae | 85.71 | 4 | 359 | 5793 | 6759 | MG451803 | Virosaurus | RNA library |
| 26557 | 12/3/2015 | 54_R | L007-r674 | Enterovirus B | Picornaviridae | 6.11 | 1 | 7 | 413 | 6759 | MG451803 | Virosaurus | RNA library |
| 25146 | 3/2/2015 | 439_R | L002-r705 | Enterovirus C [Non-polio] | Picornaviridae | 13.83 | 1 | 13 | 1032 | 7463 | AB769152 | Virosaurus | RNA library |
| 21333 | 5/11/2015 | 229_D | L004-r61 | Geminivirus | Genomoviridae | 27.54 | 19 | 121 | 600 | 2179 | MN765192 | De novo | DNA library |
| 21573 | 6/15/2015 | 281_D | L4-r65 | Geminivirus | Genomoviridae | 37.08 | 4 | 24 | 808 | 2179 | MN765192 | De novo | DNA library |
| 21712 | 7/9/2015 | 301_D | L2-r65 | Geminivirus | Genomoviridae | 21.11 | 7 | 55 | 460 | 2179 | MN765192 | De novo | DNA library |
| 22212 | 9/22/2015 | 368_D | L004-r73 | Geminivirus | Genomoviridae | 60.53 | 6 | 93 | 1319 | 2179 | MN765192 | De novo | DNA library |
| 22415 | 10/28/2015 | 10_D | L003-r44 | Geminivirus | Genomoviridae | 21.94 | 12 | 63 | 478 | 2179 | MN765192 | De novo | DNA library |
| 22419 | 10/29/2015 | 785_D | L6-r113 | Geminivirus | Genomoviridae | 68.89 | 16 | 323 | 1501 | 2179 | MN765192 | De novo | DNA library |
| 22460 | 11/17/2015 | 16_D | L001-r44 | Geminivirus | Genomoviridae | 99.82 | 117 | 2750 | 2175 | 2179 | MN765192 | De novo | DNA library |
| 25156 | 3/4/2015 | 443_D | L006-r73 | Geminivirus | Genomoviridae | 17.07 | 1 | 12 | 372 | 2179 | MN765192 | De novo | DNA library |
| 25233 | 3/24/2015 | 465_D | L002-r80 | Geminivirus | Genomoviridae | 99.95 | 147 | 3125 | 2178 | 2179 | MN765192 | De novo | DNA library |
| 26502 | 11/16/2015 | 673_D | L8-r91 | Geminivirus | Genomoviridae | 26.53 | 12 | 79 | 578 | 2179 | MN765192 | De novo | DNA library |
| 26546 | 11/30/2015 | 70_D | L004-r46 | Geminivirus | Genomoviridae | 33 | 17 | 137 | 719 | 2179 | MN765192 | De novo | DNA library |
| 21988 | 8/25/2015 | 338_R | L007-r698 | Hepatovirus A | Picornaviridae | 97.28 | 29 | 2506 | 7317 | 7522 | KX035096 | Virosaurus | RNA library |
| 22001 | 8/26/2015 | 339_R | L007-r698 | Hepatovirus A | Picornaviridae | 43.42 | 1 | 64 | 3266 | 7522 | KX035096 | Virosaurus | RNA library |
| 22107 | 9/9/2015 | 352_R | L003-r702 | Hepatovirus A | Picornaviridae | 22.89 | 1 | 28 | 1722 | 7522 | KX035096 | Virosaurus | RNA library |
| 22359 | 11/6/2015 | 390_R | L005-r703 | Hepatovirus A | Picornaviridae | 5.4 | 1 | 6 | 406 | 7522 | KX035096 | Virosaurus | RNA library |
| 22408 | 10/23/2015 | 395_R | L006-r703 | Hepatovirus A | Picornaviridae | 99.08 | 767 | 63168 | 7453 | 7522 | KX035096 | Virosaurus | RNA library |
| 22495 | 11/24/2015 | 403_R | L008-r703 | Hepatovirus A | Picornaviridae | 6.97 | 1 | 6 | 524 | 7522 | KX035096 | Virosaurus | RNA library |
| 22601 | 12/4/2015 | 664_R | L4-r725 | Hepatovirus A | Picornaviridae | 43.89 | 1 | 63 | 3301 | 7522 | KX035096 | Virosaurus | RNA library |
| 25114 | 2/20/2015 | 434_R | L001-r705 | Hepatovirus A | Picornaviridae | 11.37 | 1 | 15 | 855 | 7522 | KX035096 | Virosaurus | RNA library |
| 26111 | 8/18/2015 | 805_R | L4-r740 | Hepatovirus A | Picornaviridae | 95.53 | 4 | 418 | 7186 | 7522 | KX035096 | Virosaurus | RNA library |
| 26149 | 8/24/2015 | 648_R | L8-r724 | Hepatovirus A | Picornaviridae | 93.69 | 4 | 327 | 7047 | 7522 | KX035096 | Virosaurus | RNA library |
| 21083 | 2/20/2015 | 111_R | L006-r676 | HPgV-1 | Flaviviridae | 82.31 | 48 | 20710 | 7620 | 9258 | KP710602 | Virosaurus | RNA library |
| 21085 | 2/23/2015 | 112_R | L006-r676 | HPgV-1 | Flaviviridae | 70.17 | 28 | 8116 | 6496 | 9258 | KP710602 | Virosaurus | RNA library |
| 21148 | 3/13/2015 | 126_R | L001-r677 | HPgV-1 | Flaviviridae | 87.44 | 73 | 15270 | 8095 | 9258 | KP710602 | Virosaurus | RNA library |
| 21220 | 3/30/2015 | 213_R | L005-r690 | HPgV-1 | Flaviviridae | 71.97 | 44 | 8980 | 6662 | 9257 | KP710606 | Virosaurus | RNA library |
| 21542 | 6/8/2015 | 274_R | L006-r692 | HPgV-1 | Flaviviridae | 90.98 | 96 | 15379 | 8423 | 9258 | KP710602 | Virosaurus | RNA library |
| 21648 | 6/29/2015 | 291_R | L003-r693 | HPgV-1 | Flaviviridae | 85.44 | 16 | 1956 | 7827 | 9161 | AY949771 | Virosaurus | RNA library |
| 21737 | 7/14/2015 | 307_R | L007-r693 | HPgV-1 | Flaviviridae | 76.09 | 9 | 1148 | 7044 | 9258 | KP710602 | Virosaurus | RNA library |
| 21766 | 7/21/2015 | 621_R | L008-r723 | HPgV-1 | Flaviviridae | 17.62 | 1 | 25 | 1632 | 9260 | KC618401 | Virosaurus | RNA library |
| 21843 | 8/3/2015 | 318_R | L002-r698 | HPgV-1 | Flaviviridae | 32.71 | 1 | 59 | 3028 | 9258 | KP710602 | Virosaurus | RNA library |
| 21849 | 8/4/2015 | 319_R | L002-r698 | HPgV-1 | Flaviviridae | 72.8 | 8 | 831 | 6740 | 9258 | KP710602 | Virosaurus | RNA library |
| 21871 | 8/6/2015 | 323_R | L003-r698 | HPgV-1 | Flaviviridae | 88.39 | 81 | 13076 | 8183 | 9258 | KP710602 | Virosaurus | RNA library |
| 21911 | 8/13/2015 | 322_R | L003-r698 | HPgV-1 | Flaviviridae | 68.89 | 6 | 707 | 6378 | 9258 | KP710602 | Virosaurus | RNA library |
| 22250 | 9/28/2015 | 372_R | L008-r702 | HPgV-1 | Flaviviridae | 86.5 | 29 | 4097 | 8008 | 9258 | KP710602 | Virosaurus | RNA library |
| 22623 | 1/7/2016 | 79_R | L005-r675 | HPgV-1 | Flaviviridae | 89.65 | 115 | 22334 | 8300 | 9258 | KP710602 | Virosaurus | RNA library |
| 25029 | 1/21/2015 | 795_R | L2-r740 | HPgV-1 | Flaviviridae | 92.64 | 67 | 9043 | 8577 | 9258 | KC618399 | Virosaurus | RNA library |
| 25093 | 2/16/2015 | 426_R | L007-r704 | HPgV-1 | Flaviviridae | 56.31 | 9 | 871 | 5291 | 9396 | AF121950 | Virosaurus | RNA library |
| 25117 | 2/20/2015 | 435_R | L001-r705 | HPgV-1 | Flaviviridae | 90.62 | 259 | 44213 | 8390 | 9258 | KP710602 | Virosaurus | RNA library |
| 25161 | 3/5/2015 | 587_R | L006-r722 | HPgV-1 | Flaviviridae | 89.25 | 15 | 1786 | 8263 | 9258 | KC618399 | Virosaurus | RNA library |
| 25196 | 3/13/2015 | 454_R | L006-r705 | HPgV-1 | Flaviviridae | 81.83 | 17 | 1994 | 7576 | 9258 | KP710602 | Virosaurus | RNA library |
| 25222 | 3/20/2015 | 464_R | L5-r707 | HPgV-1 | Flaviviridae | 76.9 | 66 | 17169 | 7119 | 9257 | KP710606 | Virosaurus | RNA library |
| 25247 | 3/26/2015 | 468_R | L6-r707 | HPgV-1 | Flaviviridae | 97.82 | 814 | 121150 | 9056 | 9258 | KC618399 | Virosaurus | RNA library |
| 25286 | 4/10/2015 | 476_R | L8-r707 | HPgV-1 | Flaviviridae | 71.13 | 71 | 21598 | 6584 | 9257 | KP710606 | Virosaurus | RNA library |
| 25353 | 4/21/2015 | 488_R | L3-r713 | HPgV-1 | Flaviviridae | 85.87 | 126 | 25834 | 7950 | 9258 | KP710602 | Virosaurus | RNA library |
| 25408 | 4/30/2015 | 497_R | L6-r713 | HPgV-1 | Flaviviridae | 46.57 | 6 | 677 | 4312 | 9260 | KC618401 | Virosaurus | RNA library |
| 25441 | 5/7/2015 | 589_R | L006-r722 | HPgV-1 | Flaviviridae | 64.46 | 30 | 7246 | 5967 | 9257 | KP710606 | Virosaurus | RNA library |
| 25517 | 5/19/2015 | 523_R | L4-r714 | HPgV-1 | Flaviviridae | 88.17 | 35 | 6422 | 8163 | 9258 | KP710602 | Virosaurus | RNA library |
| 25644 | 6/4/2015 | 564_R | L7-r717 | HPgV-1 | Flaviviridae | 88.15 | 12 | 1699 | 8161 | 9258 | KP710602 | Virosaurus | RNA library |
| 25683 | 6/9/2015 | 576_R | L002-r722 | HPgV-1 | Flaviviridae | 92.56 | 56 | 5643 | 8569 | 9258 | KC618399 | Virosaurus | RNA library |
| 25783 | 6/25/2015 | 607_R | L005-r723 | HPgV-1 | Flaviviridae | 87.99 | 346 | 53279 | 8146 | 9258 | KP710602 | Virosaurus | RNA library |
| 25850 | 7/3/2015 | 726_R | L6-r735 | HPgV-1 | Flaviviridae | 90.34 | 190 | 37483 | 8364 | 9258 | KP710602 | Virosaurus | RNA library |
| 25851 | 7/6/2015 | 727_R | L6-r735 | HPgV-1 | Flaviviridae | 60.93 | 12 | 3379 | 5640 | 9257 | KP710606 | Virosaurus | RNA library |
| 25898 | 7/13/2015 | 737_R | L3-r736 | HPgV-1 | Flaviviridae | 68.76 | 11 | 1276 | 6366 | 9258 | KP710602 | Virosaurus | RNA library |
| 26028 | 8/5/2015 | 751_R | L6-r736 | HPgV-1 | Flaviviridae | 92 | 99 | 20712 | 8519 | 9260 | KC618401 | Virosaurus | RNA library |
| 26141 | 8/25/2015 | 646_R | L7-r724 | HPgV-1 | Flaviviridae | 92.34 | 515 | 75907 | 8549 | 9258 | KP710602 | Virosaurus | RNA library |
| 26153 | 8/26/2015 | 814_R | L7-r740 | HPgV-1 | Flaviviridae | 94.12 | 342 | 53064 | 8714 | 9258 | KP710602 | Virosaurus | RNA library |
| 26177 | 8/28/2015 | 821_R | L1-r741 | HPgV-1 | Flaviviridae | 88.1 | 162 | 23758 | 8156 | 9258 | KP710602 | Virosaurus | RNA library |
| 26225 | 9/4/2015 | 840_R | L6-r741 | HPgV-1 | Flaviviridae | 90.25 | 107 | 13808 | 8357 | 9260 | KC618401 | Virosaurus | RNA library |
| 26247 | 9/8/2015 | 846_R | L7-r741 | HPgV-1 | Flaviviridae | 95.6 | 1251 | 252361 | 8851 | 9258 | KP710602 | Virosaurus | RNA library |
| 26309 | 9/15/2015 | 882_R | L1-r746 | HPgV-1 | Flaviviridae | 98.22 | 718 | 126637 | 9093 | 9258 | KC618399 | Virosaurus | RNA library |
| 26312 | 9/15/2015 | 884_R | L2-r746 | HPgV-1 | Flaviviridae | 85.26 | 48 | 7705 | 7893 | 9258 | KP710602 | Virosaurus | RNA library |
| 26318 | 9/16/2015 | 885_R | L2-r746 | HPgV-1 | Flaviviridae | 90.16 | 700 | 102586 | 8347 | 9258 | KP710602 | Virosaurus | RNA library |
| 26343 | 9/17/2015 | 894_R | L4-r746 | HPgV-1 | Flaviviridae | 82.83 | 29 | 3392 | 7668 | 9258 | KP710602 | Virosaurus | RNA library |
| 21090 | 2/24/2015 | 113_R | L006-r676 | HPIV-4a | Paramyxoviridae | 1.85 | 1 | 4 | 317 | 17099 | KF483663 | Virosaurus | RNA library |
| 21722 | 7/10/2015 | 306_D | L007-r67 | Human alphaherpesvirus 1 | Herpesviridae | 0.44 | Na | 6 | 518 | Na | HM585502 | Virosaurus | DNA library |
| 21737 | 7/14/2015 | 307_D | L1-r65 | Human alphaherpesvirus 3 | Herpesviridae | 9.37 | Na | 141 | 9529 | Na | DQ008354:DQ479957:JN704702:KM355709:NC_001348 | Virosaurus | DNA library |
| 21737 | 7/14/2015 | 307_R | L007-r693 | Human alphaherpesvirus 3 | Herpesviridae | 1.6 | Na | 24 | 1575 | Na | DQ452050:NC_001348:S56048 | Virosaurus | RNA library |
| 21911 | 8/13/2015 | 322_D | L7-r66 | Human alphaherpesvirus 3 | Herpesviridae | 0.42 | Na | 7 | 431 | Na | DQ479957:NC_001348 | Virosaurus | DNA library |
| 21911 | 8/13/2015 | 322_R | L003-r698 | Human alphaherpesvirus 3 | Herpesviridae | 1.3 | Na | 24 | 1310 | Na | JN704702:NC_001348 | Virosaurus | RNA library |
| 22285 | 10/15/2015 | 378_D | L004-r71 | Human alphaherpesvirus 3 | Herpesviridae | 17.06 | Na | 263 | 17425 | Na | DQ008354:DQ452050:DQ479957:JN704702:NC_001348 | Virosaurus | DNA library |
| 22285 | 10/15/2015 | 378_R | L002-r703 | Human alphaherpesvirus 3 | Herpesviridae | 1.56 | Na | 27 | 1586 | Na | DQ008354:DQ479957:NC_001348 | Virosaurus | RNA library |
| 26147 | 8/25/2015 | 813_D | L6-r122 | Human alphaherpesvirus 3 | Herpesviridae | 5.07 | Na | 112 | 5238 | Na | DQ008354:DQ452050:DQ479957:JN704702:KM355709:NC_001348 | Virosaurus | DNA library |
| 26147 | 8/25/2015 | 813_R | L7-r740 | Human alphaherpesvirus 3 | Herpesviridae | 2.72 | Na | 39 | 2773 | Na | DQ008354:DQ452050:DQ479957:JN704702:NC_001348 | Virosaurus | RNA library |
| 25837 | 7/2/2015 | 720_D | L4-r92 | Human associated cyclovirus 12 | Circoviridae | 100 | 2543 | 47437 | 1724 | 1724 | NC_032682 | Virosaurus | DNA library |
| 25837 | 7/2/2015 | 720_R | L4-r735 | Human associated cyclovirus 12 | Circoviridae | 89.5 | 4 | 92 | 1543 | 1724 | NC_032682 | Virosaurus | RNA library |
| 26140 | 8/25/2015 | 647_D | L3-r91 | Human associated cyclovirus 8 | Circoviridae | 33.87 | 4 | 44 | 629 | 1857 | AB937984 | Virosaurus | DNA library |
| 22062 | 9/3/2015 | 345_D | L1-r66 | Human associated cyclovirus 9 | Circoviridae | 16.37 | 1 | 4 | 300 | 1833 | NC_021568 | Virosaurus | DNA library |
| 22259 | 9/29/2015 | 374_D | L003-r73 | Human associated cyclovirus 9 | Circoviridae | 19.42 | 3 | 10 | 356 | 1833 | NC_021568 | Virosaurus | DNA library |
| 22435 | 11/6/2015 | 39_D | L003-r44 | Human associated cyclovirus 9 | Circoviridae | 18.22 | 1 | 6 | 334 | 1833 | NC_021568 | Virosaurus | DNA library |
| 21006 | 1/16/2015 | 92_D | L002-r49 | Human associated gemykibivirus 2 | Genomoviridae | 95 | 330 | 7646 | 2090 | 2200 | NC_026818 | Virosaurus | DNA library |
| 21044 | 2/3/2015 | 102_D | L005-r49 | Human associated gemykibivirus 2 | Genomoviridae | 14.5 | 3 | 18 | 319 | 2200 | NC_026818 | Virosaurus | DNA library |
| 21191 | 3/20/2015 | 50_D | L005-r46 | Human associated gemykibivirus 2 | Genomoviridae | 16.41 | 1 | 5 | 361 | 2200 | NC_026818 | Virosaurus | DNA library |
| 21247 | 4/10/2015 | 215_D | L004-r57 | Human associated gemykibivirus 2 | Genomoviridae | 14.64 | 1 | 4 | 322 | 2200 | NC_026818 | Virosaurus | DNA library |
| 21332 | 5/11/2015 | 228_D | L003-r61 | Human associated gemykibivirus 2 | Genomoviridae | 33.96 | 2 | 26 | 747 | 2200 | NC_026818 | Virosaurus | DNA library |
| 21333 | 5/11/2015 | 229_D | L004-r61 | Human associated gemykibivirus 2 | Genomoviridae | 13.73 | 1 | 4 | 302 | 2200 | NC_026818 | Virosaurus | DNA library |
| 21449 | 5/25/2015 | 250_D | L004-r61 | Human associated gemykibivirus 2 | Genomoviridae | 14.18 | 1 | 4 | 312 | 2200 | NC_026818 | Virosaurus | DNA library |
| 21701 | 7/9/2015 | 298_D | L7-r65 | Human associated gemykibivirus 2 | Genomoviridae | 68.73 | 1 | 30 | 1512 | 2200 | NC_026818 | Virosaurus | DNA library |
| 21789 | 7/23/2015 | 627_D | L1-r92 | Human associated gemykibivirus 2 | Genomoviridae | 13.64 | 1 | 4 | 300 | 2200 | NC_026818 | Virosaurus | DNA library |
| 22212 | 9/22/2015 | 368_D | L004-r73 | Human associated gemykibivirus 2 | Genomoviridae | 41.36 | 1 | 14 | 910 | 2200 | NC_026818 | Virosaurus | DNA library |
| 22215 | 9/22/2015 | 369_D | L002-r71 | Human associated gemykibivirus 2 | Genomoviridae | 13.64 | 1 | 3 | 300 | 2200 | NC_026818 | Virosaurus | DNA library |
| 22259 | 9/29/2015 | 374_D | L003-r73 | Human associated gemykibivirus 2 | Genomoviridae | 15.5 | 2 | 8 | 341 | 2200 | NC_026818 | Virosaurus | DNA library |
| 22277 | 10/12/2015 | 782_D | L8-r111 | Human associated gemykibivirus 2 | Genomoviridae | 27.55 | 1 | 9 | 606 | 2200 | NC_026818 | Virosaurus | DNA library |
| 22285 | 10/15/2015 | 378_D | L004-r71 | Human associated gemykibivirus 2 | Genomoviridae | 94.18 | 36 | 970 | 2072 | 2200 | NC_026818 | Virosaurus | DNA library |
| 22293 | 10/19/2015 | 381_D | L003-r73 | Human associated gemykibivirus 2 | Genomoviridae | 47.64 | 1 | 18 | 1048 | 2200 | NC_026818 | Virosaurus | DNA library |
| 22405 | 10/22/2015 | 393_D | L001-r73 | Human associated gemykibivirus 2 | Genomoviridae | 67.41 | 2 | 41 | 1483 | 2200 | NC_026818 | Virosaurus | DNA library |
| 22415 | 10/28/2015 | 10_D | L003-r44 | Human associated gemykibivirus 2 | Genomoviridae | 35.5 | 1 | 9 | 781 | 2200 | NC_026818 | Virosaurus | DNA library |
| 22418 | 10/29/2015 | 668_D | L2-r91 | Human associated gemykibivirus 2 | Genomoviridae | 28.14 | 1 | 10 | 619 | 2200 | NC_026818 | Virosaurus | DNA library |
| 22419 | 10/29/2015 | 785_D | L6-r113 | Human associated gemykibivirus 2 | Genomoviridae | 93.23 | 5 | 125 | 2051 | 2200 | NC_026818 | Virosaurus | DNA library |
| 22432 | 11/4/2015 | 398_D | L002-r71 | Human associated gemykibivirus 2 | Genomoviridae | 52.82 | 1 | 30 | 1162 | 2200 | NC_026818 | Virosaurus | DNA library |
| 22453 | 11/16/2015 | 400_D | L004-r71 | Human associated gemykibivirus 2 | Genomoviridae | 49.36 | 2 | 30 | 1086 | 2200 | NC_026818 | Virosaurus | DNA library |
| 22460 | 11/17/2015 | 16_D | L001-r44 | Human associated gemykibivirus 2 | Genomoviridae | 52.23 | 2 | 24 | 1149 | 2200 | NC_026818 | Virosaurus | DNA library |
| 22461 | 11/17/2015 | 24_D | L002-r44 | Human associated gemykibivirus 2 | Genomoviridae | 39.73 | 1 | 13 | 874 | 2200 | NC_026818 | Virosaurus | DNA library |
| 22624 | 1/7/2016 | 75_D | L001-r46 | Human associated gemykibivirus 2 | Genomoviridae | 59.05 | 2 | 32 | 1299 | 2200 | NC_026818 | Virosaurus | DNA library |
| 25174 | 3/6/2015 | 449_D | L005-r73 | Human associated gemykibivirus 2 | Genomoviridae | 94.18 | 23 | 580 | 2072 | 2200 | NC_026818 | Virosaurus | DNA library |
| 25213 | 3/19/2015 | 460_D | L002-r77 | Human associated gemykibivirus 2 | Genomoviridae | 16.77 | 1 | 5 | 369 | 2200 | NC_026818 | Virosaurus | DNA library |
| 25329 | 4/16/2015 | 484_D | L005-r77 | Human associated gemykibivirus 2 | Genomoviridae | 13.82 | 1 | 4 | 304 | 2200 | NC_026818 | Virosaurus | DNA library |
| 26502 | 11/16/2015 | 673_D | L8-r91 | Human associated gemykibivirus 2 | Genomoviridae | 62.77 | 2 | 42 | 1381 | 2200 | NC_026818 | Virosaurus | DNA library |
| 26503 | 11/16/2015 | 41_D | L006-r44 | Human associated gemykibivirus 2 | Genomoviridae | 59.59 | 2 | 28 | 1311 | 2200 | NC_026818 | Virosaurus | DNA library |
| 21148 | 3/13/2015 | 126_R | L001-r677 | Human associated gemyvongvirus 1 | Genomoviridae | 30.14 | 1 | 12 | 683 | 2266 | KP974694 | Virosaurus | RNA library |
| 21021 | 1/26/2015 | 96_R | L002-r676 | Human betaherpesvirus 5 | Herpesviridae | 0.39 | Na | 8 | 582 | Na | KJ361952:KX544841 | Virosaurus | RNA library |
| 21195 | 12/11/2015 | 60_D | L008-r46 | Human betaherpesvirus 5 | Herpesviridae | 0.36 | Na | 9 | 539 | Na | JX512198:KP745642:NC_006273 | Virosaurus | DNA library |
| 21819 | 7/30/2015 | 314_D | L1-r65 | Human betaherpesvirus 5 | Herpesviridae | 0.99 | Na | 21 | 1486 | Na | LT907985:NC_006273 | Virosaurus | DNA library |
| 21819 | 7/30/2015 | 314_R | L008-r693 | Human betaherpesvirus 5 | Herpesviridae | 0.68 | Na | 12 | 999 | Na | AY436380:FJ527563:KJ361949:KJ361957:LT907985:NC_006273 | Virosaurus | RNA library |
| 21877 | 8/7/2015 | 640_D | L3-r91 | Human betaherpesvirus 5 | Herpesviridae | 0.37 | Na | 8 | 546 | Na | GENE_6759-13155NC_006273:KY490076:NC_006273 | Virosaurus | DNA library |
| 21877 | 8/7/2015 | 640_R | L6-r724 | Human betaherpesvirus 5 | Herpesviridae | 1.16 | Na | 28 | 1738 | Na | JX512207:KJ361952:KP745645:KX101021:M81432:NC_006273 | Virosaurus | RNA library |
| 22321 | 10/20/2015 | 388_R | L004-r703 | Human betaherpesvirus 5 | Herpesviridae | 0.8 | Na | 20 | 1197 | Na | KJ361947:KX544841:M81432:NC_006273 | Virosaurus | RNA library |
| 22409 | 10/23/2015 | 396_D | L004-r73 | Human betaherpesvirus 5 | Herpesviridae | 1.41 | Na | 31 | 1956 | Na | GU179288:JX512202:JX512207:KC519319:KJ361949:NC_006273 | Virosaurus | DNA library |
| 22601 | 12/4/2015 | 664_D | L6-r91 | Human betaherpesvirus 5 | Herpesviridae | 0.3 | Na | 14 | 447 | Na | LT907985 | Virosaurus | DNA library |
| 25194 | 3/12/2015 | 452_D | L001-r77 | Human betaherpesvirus 5 | Herpesviridae | 2.33 | Na | 60 | 3474 | Na | GQ221974:GQ466044:JX512199:JX512207:KJ361952:KJ361957:KP745654:KX101021:KY490084:LT907985:NC_006273 | Virosaurus | DNA library |
| 25194 | 3/12/2015 | 452_R | L006-r705 | Human betaherpesvirus 5 | Herpesviridae | 0.14 | Na | 3 | 204 | Na | KJ361952 | Virosaurus | RNA library |
| 25329 | 4/16/2015 | 484_R | L2-r713 | Human betaherpesvirus 5 | Herpesviridae | 1.36 | Na | 27 | 2028 | Na | FJ527563:GENE_6759-13155LT907985:NC_006273 | Virosaurus | RNA library |
| 25365 | 4/22/2015 | 491_R | L4-r713 | Human betaherpesvirus 5 | Herpesviridae | 0.2 | Na | 4 | 301 | Na | NC_006273 | Virosaurus | RNA library |
| 25600 | 5/28/2015 | 544_D | L4-r85 | Human betaherpesvirus 5 | Herpesviridae | 0.34 | Na | 6 | 499 | Na | LT907985:NC_006273 | Virosaurus | DNA library |
| 25600 | 5/28/2015 | 544_R | L2-r717 | Human betaherpesvirus 5 | Herpesviridae | 0.36 | Na | 6 | 516 | Na | DQ485984:KJ361947:NC_006273 | Virosaurus | RNA library |
| 25604 | 5/29/2015 | 548_D | L8-r85 | Human betaherpesvirus 5 | Herpesviridae | 0.75 | Na | 17 | 1118 | Na | LT907985:NC_006273 | Virosaurus | DNA library |
| 25604 | 5/29/2015 | 548_R | L3-r717 | Human betaherpesvirus 5 | Herpesviridae | 1.28 | Na | 28 | 1907 | Na | FJ527563:GU179290:KJ176662:KJ361952:NC_006273 | Virosaurus | RNA library |
| 25692 | 6/10/2015 | 581_D | L6-r85 | Human betaherpesvirus 5 | Herpesviridae | 1.01 | Na | 18 | 1495 | Na | LT907985 | Virosaurus | DNA library |
| 25874 | 7/9/2015 | 776_D | L4-r113 | Human betaherpesvirus 5 | Herpesviridae | 0.27 | Na | 5 | 407 | Na | KR534205 | Virosaurus | DNA library |
| 26224 | 9/4/2015 | 839_R | L6-r741 | Human betaherpesvirus 5 | Herpesviridae | 0.23 | Na | 4 | 340 | Na | LT907985 | Virosaurus | RNA library |
| 21129 | 3/10/2015 | 125_R | L001-r677 | Human betaherpesvirus 6 | Herpesviridae | 0.55 | Na | 8 | 676 | Na | NC_000898 | Virosaurus | RNA library |
| 21519 | 6/3/2015 | 267_D | L007-r61 | Human betaherpesvirus 6 | Herpesviridae | 0.66 | Na | 13 | 835 | Na | NC_000898 | Virosaurus | DNA library |
| 21519 | 6/3/2015 | 267_R | L004-r692 | Human betaherpesvirus 6 | Herpesviridae | 1.43 | Na | 27 | 1773 | Na | NC_000898 | Virosaurus | RNA library |
| 21528 | 6/5/2015 | 271_D | L1-r65 | Human betaherpesvirus 6 | Herpesviridae | 4.97 | Na | 89 | 6324 | Na | KC465951:KY316036:NC_000898 | Virosaurus | DNA library |
| 21528 | 6/5/2015 | 271_R | L006-r692 | Human betaherpesvirus 6 | Herpesviridae | 3.26 | Na | 62 | 4239 | Na | KC465951:NC_000898 | Virosaurus | RNA library |
| 21571 | 6/15/2015 | 280_D | L3-r65 | Human betaherpesvirus 6 | Herpesviridae | 0.36 | Na | 13 | 459 | Na | NC_000898 | Virosaurus | DNA library |
| 21571 | 6/15/2015 | 280_R | L008-r692 | Human betaherpesvirus 6 | Herpesviridae | 0.39 | Na | 7 | 478 | Na | KC465951:NC_000898 | Virosaurus | RNA library |
| 21715 | 7/10/2015 | 303_D | L5-r65 | Human betaherpesvirus 6 | Herpesviridae | 2.01 | Na | 40 | 2612 | Na | NC_000898 | Virosaurus | DNA library |
| 21715 | 7/10/2015 | 303_R | L006-r693 | Human betaherpesvirus 6 | Herpesviridae | 0.63 | Na | 10 | 783 | Na | KY316042:NC_000898:NC_000898U13194 | Virosaurus | RNA library |
| 21879 | 8/7/2015 | 324_D | L2-r66 | Human betaherpesvirus 6 | Herpesviridae | 1.55 | Na | 30 | 2019 | Na | NC_000898 | Virosaurus | DNA library |
| 21879 | 8/7/2015 | 324_R | L004-r698 | Human betaherpesvirus 6 | Herpesviridae | 0.65 | Na | 12 | 828 | Na | KC465951:NC_000898 | Virosaurus | RNA library |
| 21988 | 8/25/2015 | 338_D | L1-r66 | Human betaherpesvirus 6 | Herpesviridae | 1.14 | Na | 21 | 1411 | Na | NC_000898 | Virosaurus | DNA library |
| 22196 | 9/18/2015 | 362_D | L002-r71 | Human betaherpesvirus 6 | Herpesviridae | 0.69 | Na | 11 | 861 | Na | AB075776:NC_000898 | Virosaurus | DNA library |
| 22196 | 9/18/2015 | 362_R | L006-r702 | Human betaherpesvirus 6 | Herpesviridae | 2.51 | Na | 47 | 3268 | Na | KC465951:NC_000898 | Virosaurus | RNA library |
| 22198 | 9/18/2015 | 363_D | L003-r71 | Human betaherpesvirus 6 | Herpesviridae | 2.06 | Na | 60 | 2554 | Na | KC465951:KY316036:NC_000898 | Virosaurus | DNA library |
| 22198 | 9/18/2015 | 363_R | L006-r702 | Human betaherpesvirus 6 | Herpesviridae | 3.47 | Na | 66 | 4526 | Na | AB075775:KC465951:KY316037:NC_000898:NC_000898KC465951 | Virosaurus | RNA library |
| 25029 | 1/21/2015 | 795_D | L6-r111 | Human betaherpesvirus 6 | Herpesviridae | 0.78 | Na | 13 | 963 | Na | KC465951:NC_000898 | Virosaurus | DNA library |
| 25029 | 1/21/2015 | 795_R | L2-r740 | Human betaherpesvirus 6 | Herpesviridae | 1.12 | Na | 23 | 1422 | Na | AB075775:KC465951:NC_000898 | Virosaurus | RNA library |
| 25085 | 2/12/2015 | 422_D | L001-r75 | Human betaherpesvirus 6 | Herpesviridae | 0.32 | Na | 5 | 400 | Na | KC465951:NC_000898 | Virosaurus | DNA library |
| 25085 | 2/12/2015 | 422_R | L006-r704 | Human betaherpesvirus 6 | Herpesviridae | 1.62 | Na | 28 | 2057 | Na | KC465951:NC_000898 | Virosaurus | RNA library |
| 25111 | 2/19/2015 | 432_D | L004-r75 | Human betaherpesvirus 6 | Herpesviridae | 1.36 | Na | 26 | 1687 | Na | KC465951:NC_000898 | Virosaurus | DNA library |
| 25111 | 2/19/2015 | 432_R | L008-r704 | Human betaherpesvirus 6 | Herpesviridae | 2.13 | Na | 39 | 2573 | Na | AB443480:KC465951:NC_000898 | Virosaurus | RNA library |
| 25276 | 4/9/2015 | 473_R | L7-r707 | Human betaherpesvirus 6 | Herpesviridae | 0.72 | Na | 11 | 892 | Na | NC_000898 | Virosaurus | RNA library |
| 25323 | 4/15/2015 | 482_R | L1-r713 | Human betaherpesvirus 6 | Herpesviridae | 1.87 | Na | 37 | 2376 | Na | NC_000898 | Virosaurus | RNA library |
| 25572 | 5/26/2015 | 532_D | L003-r80 | Human betaherpesvirus 6 | Herpesviridae | 0.6 | Na | 14 | 740 | Na | NC_000898 | Virosaurus | DNA library |
| 25715 | 6/15/2015 | 585_D | L2-r85 | Human betaherpesvirus 6 | Herpesviridae | 4.6 | Na | 99 | 5980 | Na | KC465951:KY316037:NC_000898 | Virosaurus | DNA library |
| 25715 | 6/15/2015 | 585_R | L005-r722 | Human betaherpesvirus 6 | Herpesviridae | 3.78 | Na | 73 | 4931 | Na | KC465951:NC_000898 | Virosaurus | RNA library |
| 25790 | 6/26/2015 | 706_D | L4-r92 | Human betaherpesvirus 6 | Herpesviridae | 0.39 | Na | 5 | 500 | Na | NC_000898 | Virosaurus | DNA library |
| 25790 | 6/26/2015 | 706_R | L8-r734 | Human betaherpesvirus 6 | Herpesviridae | 0.27 | Na | 4 | 339 | Na | NC_000898 | Virosaurus | RNA library |
| 25795 | 6/26/2015 | 708_D | L1-r93 | Human betaherpesvirus 6 | Herpesviridae | 0.6 | Na | 11 | 744 | Na | KC465951:NC_000898 | Virosaurus | DNA library |
| 25795 | 6/26/2015 | 708_R | L1-r735 | Human betaherpesvirus 6 | Herpesviridae | 1.82 | Na | 28 | 2254 | Na | KC465951:NC_000898 | Virosaurus | RNA library |
| 25831 | 7/1/2015 | 719_D | L3-r92 | Human betaherpesvirus 6 | Herpesviridae | 0.4 | Na | 7 | 500 | Na | NC_000898 | Virosaurus | DNA library |
| 25831 | 7/1/2015 | 719_R | L3-r735 | Human betaherpesvirus 6 | Herpesviridae | 0.88 | Na | 13 | 1094 | Na | NC_000898 | Virosaurus | RNA library |
| 26091 | 8/14/2015 | 761_D | L5-r96 | Human betaherpesvirus 6 | Herpesviridae | 0.27 | Na | 5 | 339 | Na | NC_000898 | Virosaurus | DNA library |
| 26091 | 8/14/2015 | 761_R | L1-r737 | Human betaherpesvirus 6 | Herpesviridae | 3.03 | Na | 68 | 3949 | Na | AB075775:KC465951:NC_000898 | Virosaurus | RNA library |
| 26203 | 9/1/2015 | 831_R | L4-r741 | Human betaherpesvirus 6 | Herpesviridae | 1.39 | Na | 25 | 1759 | Na | KC465951:NC_000898 | Virosaurus | RNA library |
| 26237 | 9/7/2015 | 843_D | L8-r122 | Human betaherpesvirus 6 | Herpesviridae | 0.51 | Na | 12 | 652 | Na | AB075776:NC_000898 | Virosaurus | DNA library |
| 26237 | 9/7/2015 | 843_R | L7-r741 | Human betaherpesvirus 6 | Herpesviridae | 1.25 | Na | 24 | 1579 | Na | KC465951:NC_000898 | Virosaurus | RNA library |
| 26259 | 9/9/2015 | 852_D | L6-r124 | Human betaherpesvirus 6 | Herpesviridae | 0.29 | Na | 8 | 356 | Na | NC_000898 | Virosaurus | DNA library |
| 26259 | 9/9/2015 | 852_R | L1-r745 | Human betaherpesvirus 6 | Herpesviridae | 0.87 | Na | 22 | 1071 | Na | NC_000898 | Virosaurus | RNA library |
| 26281 | 9/11/2015 | 658_D | L7-r91 | Human betaherpesvirus 6 | Herpesviridae | 1.03 | Na | 28 | 1338 | Na | KC465951:NC_000898 | Virosaurus | DNA library |
| 26424 | 10/23/2015 | 1_D | L008-r54 | Human betaherpesvirus 6 | Herpesviridae | 0.8 | Na | 14 | 988 | Na | NC_000898 | Virosaurus | DNA library |
| 26424 | 10/23/2015 | 1_R | L2-r669 | Human betaherpesvirus 6 | Herpesviridae | 3.69 | Na | 71 | 4697 | Na | AB075775:KC465951:NC_000898:NC_000898KC465951:U13194 | Virosaurus | RNA library |
| 26439 | 10/28/2015 | 2_D | L002-r44 | Human betaherpesvirus 6 | Herpesviridae | 1.38 | Na | 29 | 1790 | Na | KC465951:NC_000898 | Virosaurus | DNA library |
| 26439 | 10/28/2015 | 2_R | L2-r669 | Human betaherpesvirus 6 | Herpesviridae | 1.63 | Na | 27 | 2071 | Na | KC465951:KY316042:NC_000898 | Virosaurus | RNA library |
| 26531 | 11/24/2015 | 77_D | L003-r46 | Human betaherpesvirus 6 | Herpesviridae | 0.86 | Na | 18 | 1096 | Na | KC465951:NC_000898:NC_000898KC465951 | Virosaurus | DNA library |
| 21116 | 3/4/2015 | 123_D | L005-r49 | Human betaherpesvirus 6B | Herpesviridae | 1.92 | Na | 34 | 2439 | Na | KC465951:KY316042:NC_000898:NC_000898KC465951 | Virosaurus | DNA library |
| 21122 | 3/5/2015 | 124_D | L006-r49 | Human betaherpesvirus 6B | Herpesviridae | 2.41 | Na | 44 | 3151 | Na | AB075775:AB075776:KC465951:KY316036:KY316042:NC_000898 | Virosaurus | DNA library |
| 21176 | 3/16/2015 | 131_D | L006-r49 | Human betaherpesvirus 6B | Herpesviridae | 0.87 | Na | 16 | 1108 | Na | KC465951:NC_000898 | Virosaurus | DNA library |
| 21176 | 3/16/2015 | 131_R | L003-r677 | Human betaherpesvirus 6B | Herpesviridae | 2.23 | Na | 44 | 2827 | Na | AB075775:KC465951:NC_000898 | Virosaurus | RNA library |
| 21262 | 4/15/2015 | 219_D | L008-r55 | Human betaherpesvirus 6B | Herpesviridae | 3.35 | Na | 64 | 4142 | Na | NC_000898:NC_000898KY316037 | Virosaurus | DNA library |
| 21262 | 4/15/2015 | 219_R | L006-r690 | Human betaherpesvirus 6B | Herpesviridae | 10.83 | Na | 234 | 14267 | Na | AB075775:AB075776:KC465951:NC_000898:U13194 | Virosaurus | RNA library |
| 21282 | 4/21/2015 | 797_D | L8-r111 | Human betaherpesvirus 6B | Herpesviridae | 5.2 | Na | 148 | 6611 | Na | KC465951:NC_000898 | Virosaurus | DNA library |
| 21362 | 5/14/2015 | 236_R | L004-r691 | Human betaherpesvirus 6B | Herpesviridae | 2.58 | Na | 46 | 3196 | Na | KC465951:NC_000898 | Virosaurus | RNA library |
| 21419 | 5/21/2015 | 244_D | L005-r61 | Human betaherpesvirus 6B | Herpesviridae | 1.74 | Na | 31 | 2211 | Na | AB075775:NC_000898:NC_000898KY316035 | Virosaurus | DNA library |
| 21419 | 5/21/2015 | 244_R | L006-r691 | Human betaherpesvirus 6B | Herpesviridae | 4.32 | Na | 87 | 5651 | Na | AB075775:KC465951:NC_000898:NC_000898KC465951 | Virosaurus | RNA library |
| 21441 | 5/25/2015 | 248_D | L001-r61 | Human betaherpesvirus 6B | Herpesviridae | 5.12 | Na | 152 | 6675 | Na | KC465951:KY316037:NC_000898 | Virosaurus | DNA library |
| 21441 | 5/25/2015 | 248_R | L007-r691 | Human betaherpesvirus 6B | Herpesviridae | 9.75 | Na | 219 | 12762 | Na | AB075775:KC465951:NC_000898:U13194 | Virosaurus | RNA library |
| 21476 | 5/28/2015 | 255_D | L001-r61 | Human betaherpesvirus 6B | Herpesviridae | 5.79 | Na | 109 | 7175 | Na | KC465951:NC_000898 | Virosaurus | DNA library |
| 21476 | 5/28/2015 | 255_R | L001-r692 | Human betaherpesvirus 6B | Herpesviridae | 4.53 | Na | 80 | 5901 | Na | KC465951:NC_000898 | Virosaurus | RNA library |
| 21491 | 5/29/2015 | 260_D | L007-r61 | Human betaherpesvirus 6B | Herpesviridae | 0.95 | Na | 18 | 1209 | Na | NC_000898:NC_000898KC465951:U13194 | Virosaurus | DNA library |
| 21491 | 5/29/2015 | 260_R | L002-r692 | Human betaherpesvirus 6B | Herpesviridae | 0.92 | Na | 15 | 1137 | Na | KC465951:NC_000898 | Virosaurus | RNA library |
| 21518 | 6/3/2015 | 266_D | L006-r61 | Human betaherpesvirus 6B | Herpesviridae | 6.37 | Na | 152 | 8073 | Na | KY316042:NC_000898 | Virosaurus | DNA library |
| 21518 | 6/3/2015 | 266_R | L004-r692 | Human betaherpesvirus 6B | Herpesviridae | 0.29 | Na | 7 | 353 | Na | KC465951:NC_000898 | Virosaurus | RNA library |
| 21574 | 6/15/2015 | 282_D | L5-r65 | Human betaherpesvirus 6B | Herpesviridae | 6.96 | Na | 150 | 8941 | Na | AB075776:KC465951:NC_000898 | Virosaurus | DNA library |
| 21574 | 6/15/2015 | 282_R | L008-r692 | Human betaherpesvirus 6B | Herpesviridae | 11.56 | Na | 243 | 15224 | Na | AB075775:AB075776:KC465951:NC_000898 | Virosaurus | RNA library |
| 21584 | 6/17/2015 | 283_D | L6-r65 | Human betaherpesvirus 6B | Herpesviridae | 0.56 | Na | 12 | 690 | Na | NC_000898 | Virosaurus | DNA library |
| 21607 | 6/22/2015 | 286_D | L1-r65 | Human betaherpesvirus 6B | Herpesviridae | 7.34 | Na | 154 | 9548 | Na | AB075775:KC465951:KY316042:NC_000898 | Virosaurus | DNA library |
| 21701 | 7/9/2015 | 298_D | L7-r65 | Human betaherpesvirus 6B | Herpesviridae | 2.07 | Na | 36 | 2626 | Na | KC465951:NC_000898 | Virosaurus | DNA library |
| 21701 | 7/9/2015 | 298_R | L004-r693 | Human betaherpesvirus 6B | Herpesviridae | 2.31 | Na | 46 | 2938 | Na | KC465951:NC_000898 | Virosaurus | RNA library |
| 21762 | 7/21/2015 | 310_D | L4-r65 | Human betaherpesvirus 6B | Herpesviridae | 4.13 | Na | 64 | 5366 | Na | KC465951:KY316036:KY316037:NC_000898:U13194 | Virosaurus | DNA library |
| 21762 | 7/21/2015 | 310_R | L007-r693 | Human betaherpesvirus 6B | Herpesviridae | 7.27 | Na | 138 | 9483 | Na | AB075775:KC465951:KY316037:NC_000898:U13194 | Virosaurus | RNA library |
| 21812 | 7/28/2015 | 313_D | L007-r67 | Human betaherpesvirus 6B | Herpesviridae | 4 | Na | 60 | 5073 | Na | KC465951:NC_000898 | Virosaurus | DNA library |
| 21812 | 7/28/2015 | 313_R | L008-r693 | Human betaherpesvirus 6B | Herpesviridae | 1.53 | Na | 27 | 1896 | Na | KC465951:NC_000898:NC_000898KC465951 | Virosaurus | RNA library |
| 21839 | 8/3/2015 | 317_D | L2-r66 | Human betaherpesvirus 6B | Herpesviridae | 4.79 | Na | 82 | 6096 | Na | AB075775:NC_000898 | Virosaurus | DNA library |
| 21839 | 8/3/2015 | 317_R | L002-r698 | Human betaherpesvirus 6B | Herpesviridae | 0.71 | Na | 13 | 897 | Na | NC_000898 | Virosaurus | RNA library |
| 21903 | 8/10/2015 | 327_D | L5-r66 | Human betaherpesvirus 6B | Herpesviridae | 5.9 | Na | 112 | 7493 | Na | AB075775:KC465951:KY316037:KY316042:NC_000898 | Virosaurus | DNA library |
| 21903 | 8/10/2015 | 327_R | L004-r698 | Human betaherpesvirus 6B | Herpesviridae | 0.86 | Na | 16 | 1062 | Na | KY316036:NC_000898:NC_000898KY316037 | Virosaurus | RNA library |
| 21920 | 8/14/2015 | 328_D | L6-r66 | Human betaherpesvirus 6B | Herpesviridae | 2.27 | Na | 68 | 2954 | Na | KC465951:NC_000898 | Virosaurus | DNA library |
| 21920 | 8/14/2015 | 328_R | L005-r698 | Human betaherpesvirus 6B | Herpesviridae | 9.35 | Na | 206 | 12212 | Na | AB075775:KC465951:KY316036:KY316037:NC_000898 | Virosaurus | RNA library |
| 22277 | 10/12/2015 | 782_D | L8-r111 | Human betaherpesvirus 6B | Herpesviridae | 2.24 | Na | 47 | 2776 | Na | KC465951:KY316042:NC_000898:NC_000898KY316037 | Virosaurus | DNA library |
| 22461 | 11/17/2015 | 24_D | L002-r44 | Human betaherpesvirus 6B | Herpesviridae | 6.99 | Na | 119 | 9167 | Na | AB075775:AB075776:KC465951:NC_000898 | Virosaurus | DNA library |
| 22461 | 11/17/2015 | 24_R | L4-r669 | Human betaherpesvirus 6B | Herpesviridae | 3.03 | Na | 57 | 3953 | Na | AB075775:KC465951:NC_000898 | Virosaurus | RNA library |
| 22484 | 11/26/2015 | 82_D | L001-r46 | Human betaherpesvirus 6B | Herpesviridae | 0.95 | Na | 23 | 1239 | Na | NC_000898 | Virosaurus | DNA library |
| 22484 | 11/26/2015 | 82_R | L006-r675 | Human betaherpesvirus 6B | Herpesviridae | 3.58 | Na | 61 | 4706 | Na | AB075776:KC465951:NC_000898 | Virosaurus | RNA library |
| 22668 | 1/28/2016 | 85_D | L004-r46 | Human betaherpesvirus 6B | Herpesviridae | 3.49 | Na | 65 | 4430 | Na | NC_000898 | Virosaurus | DNA library |
| 22668 | 1/28/2016 | 85_R | L006-r675 | Human betaherpesvirus 6B | Herpesviridae | 3.9 | Na | 65 | 5074 | Na | KC465951:NC_000898 | Virosaurus | RNA library |
| 22686 | 2/9/2016 | 63_D | L004-r46 | Human betaherpesvirus 6B | Herpesviridae | 48.94 | Na | 1708 | 64098 | Na | AB075775:KC465951:NC_000898:U13194 | Virosaurus | DNA library |
| 22686 | 2/9/2016 | 63_R | L001-r675 | Human betaherpesvirus 6B | Herpesviridae | 10.14 | Na | 226 | 13320 | Na | AB075776:KC465951:KY316042:NC_000898 | Virosaurus | RNA library |
| 25033 | 1/22/2015 | 415_D | L001-r75 | Human betaherpesvirus 6B | Herpesviridae | 7.25 | Na | 141 | 9425 | Na | AB075775:KY316036:KY316042:NC_000898 | Virosaurus | DNA library |
| 25093 | 2/16/2015 | 426_D | L005-r75 | Human betaherpesvirus 6B | Herpesviridae | 5.5 | Na | 93 | 7183 | Na | AB075775:KC465951:NC_000898:NC_000898KY316037 | Virosaurus | DNA library |
| 25093 | 2/16/2015 | 426_R | L007-r704 | Human betaherpesvirus 6B | Herpesviridae | 1.3 | Na | 19 | 1605 | Na | NC_000898 | Virosaurus | RNA library |
| 25160 | 3/5/2015 | 444_D | L001-r75 | Human betaherpesvirus 6B | Herpesviridae | 18.73 | Na | 351 | 24480 | Na | AB075775:KC465951:KY316037:KY316042:NC_000898 | Virosaurus | DNA library |
| 25160 | 3/5/2015 | 444_R | L003-r705 | Human betaherpesvirus 6B | Herpesviridae | 0.39 | Na | 7 | 500 | Na | NC_000898 | Virosaurus | RNA library |
| 25302 | 4/16/2015 | 480_D | L007-r75 | Human betaherpesvirus 6B | Herpesviridae | 39.35 | Na | 844 | 51468 | Na | AB075775:KC465951:KY316036:KY316042:NC_000898:NC_000898KC465951:U13194 | Virosaurus | DNA library |
| 25302 | 4/16/2015 | 480_R | L1-r713 | Human betaherpesvirus 6B | Herpesviridae | 1.06 | Na | 20 | 1382 | Na | AB075775:KC465951:NC_000898:NC_000898U13194 | Virosaurus | RNA library |
| 25491 | 5/15/2015 | 520_D | L006-r80 | Human betaherpesvirus 6B | Herpesviridae | 1.66 | Na | 36 | 2157 | Na | AB075775:NC_000898 | Virosaurus | DNA library |
| 25532 | 5/20/2015 | 525_D | L003-r80 | Human betaherpesvirus 6B | Herpesviridae | 1.9 | Na | 57 | 2409 | Na | NC_000898 | Virosaurus | DNA library |
| 25532 | 5/20/2015 | 525_R | L5-r714 | Human betaherpesvirus 6B | Herpesviridae | 1.05 | Na | 22 | 1328 | Na | KY316042:NC_000898:NC_000898KC465951 | Virosaurus | RNA library |
| 25551 | 5/22/2015 | 528_D | L5-r82 | Human betaherpesvirus 6B | Herpesviridae | 48.31 | Na | 1950 | 63179 | Na | AB075775:KC465951:KY316036:KY316042:NC_000898:U13194 | Virosaurus | DNA library |
| 25551 | 5/22/2015 | 528_R | L6-r714 | Human betaherpesvirus 6B | Herpesviridae | 6.78 | Na | 143 | 8868 | Na | AB075775:KC465951:NC_000898 | Virosaurus | RNA library |
| 25558 | 5/22/2015 | 529_D | L6-r82 | Human betaherpesvirus 6B | Herpesviridae | 1.35 | Na | 23 | 1664 | Na | NC_000898 | Virosaurus | DNA library |
| 25558 | 5/22/2015 | 529_R | L6-r714 | Human betaherpesvirus 6B | Herpesviridae | 1.74 | Na | 38 | 2265 | Na | AB075775:KC465951:NC_000898 | Virosaurus | RNA library |
| 25596 | 5/28/2015 | 541_D | L1-r85 | Human betaherpesvirus 6B | Herpesviridae | 1.18 | Na | 21 | 1501 | Na | NC_000898 | Virosaurus | DNA library |
| 25596 | 5/28/2015 | 541_R | L2-r717 | Human betaherpesvirus 6B | Herpesviridae | 3.59 | Na | 62 | 4477 | Na | AB075775:KC465951:NC_000898 | Virosaurus | RNA library |
| 25634 | 6/3/2015 | 560_D | L6-r85 | Human betaherpesvirus 6B | Herpesviridae | 29.8 | Na | 760 | 39030 | Na | AB075775:KC465951:KY316037:NC_000898:U13194 | Virosaurus | DNA library |
| 25634 | 6/3/2015 | 560_R | L6-r717 | Human betaherpesvirus 6B | Herpesviridae | 3.32 | Na | 63 | 4219 | Na | KC465951:KY316037:NC_000898 | Virosaurus | RNA library |
| 25635 | 6/3/2015 | 561_D | L7-r85 | Human betaherpesvirus 6B | Herpesviridae | 0.51 | Na | 8 | 648 | Na | KC465951:NC_000898 | Virosaurus | DNA library |
| 25635 | 6/3/2015 | 561_R | L7-r717 | Human betaherpesvirus 6B | Herpesviridae | 3.68 | Na | 96 | 4682 | Na | KC465951:NC_000898 | Virosaurus | RNA library |
| 25644 | 6/4/2015 | 564_D | L2-r85 | Human betaherpesvirus 6B | Herpesviridae | 8.49 | Na | 224 | 10822 | Na | KC465951:NC_000898 | Virosaurus | DNA library |
| 25679 | 6/9/2015 | 574_D | L6-r85 | Human betaherpesvirus 6B | Herpesviridae | 8.91 | Na | 214 | 11318 | Na | KC465951:NC_000898 | Virosaurus | DNA library |
| 25730 | 6/17/2015 | 686_D | L6-r92 | Human betaherpesvirus 6B | Herpesviridae | 38.94 | Na | 1115 | 51199 | Na | AB075776:KC465951:KY316036:NC_000898 | Virosaurus | DNA library |
| 25730 | 6/17/2015 | 686_R | L3-r734 | Human betaherpesvirus 6B | Herpesviridae | 16.36 | Na | 484 | 21335 | Na | AB075775:KC465951:KY316039:NC_000898 | Virosaurus | RNA library |
| 25747 | 6/22/2015 | 692_D | L5-r92 | Human betaherpesvirus 6B | Herpesviridae | 0.66 | Na | 11 | 834 | Na | NC_000898 | Virosaurus | DNA library |
| 25747 | 6/22/2015 | 692_R | L5-r734 | Human betaherpesvirus 6B | Herpesviridae | 2.91 | Na | 52 | 3613 | Na | AB075775:KC465951:NC_000898:NC_000898KC465951 | Virosaurus | RNA library |
| 25756 | 6/22/2015 | 599_D | L1-r92 | Human betaherpesvirus 6B | Herpesviridae | 43.25 | Na | 942 | 56982 | Na | AB021506:AB075775:AB075776:KC465951:KY316036:KY316042:NC_000898:U13194 | Virosaurus | DNA library |
| 25777 | 6/24/2015 | 604_D | L3-r89 | Human betaherpesvirus 6B | Herpesviridae | 0.97 | Na | 17 | 1267 | Na | AB075776:KC465951:KY316042:NC_000898 | Virosaurus | DNA library |
| 25777 | 6/24/2015 | 604_R | L004-r723 | Human betaherpesvirus 6B | Herpesviridae | 5.84 | Na | 138 | 7490 | Na | AB075775:AB075776:KC465951:NC_000898 | Virosaurus | RNA library |
| 25813 | 6/30/2015 | 612_D | L4-r89 | Human betaherpesvirus 6B | Herpesviridae | 8.03 | Na | 167 | 10191 | Na | KC465951:KY316042:NC_000898 | Virosaurus | DNA library |
| 25813 | 6/30/2015 | 612_R | L006-r723 | Human betaherpesvirus 6B | Herpesviridae | 0.38 | Na | 6 | 465 | Na | NC_000898 | Virosaurus | RNA library |
| 25818 | 6/30/2015 | 713_D | L4-r92 | Human betaherpesvirus 6B | Herpesviridae | 5.59 | Na | 146 | 7269 | Na | KC465951:NC_000898 | Virosaurus | DNA library |
| 25818 | 6/30/2015 | 713_R | L2-r735 | Human betaherpesvirus 6B | Herpesviridae | 5.53 | Na | 101 | 7039 | Na | AB075775:KC465951:NC_000898 | Virosaurus | RNA library |
| 25837 | 7/2/2015 | 720_D | L4-r92 | Human betaherpesvirus 6B | Herpesviridae | 1.07 | Na | 24 | 1359 | Na | NC_000898 | Virosaurus | DNA library |
| 25837 | 7/2/2015 | 720_R | L4-r735 | Human betaherpesvirus 6B | Herpesviridae | 0.55 | Na | 10 | 704 | Na | NC_000898 | Virosaurus | RNA library |
| 25851 | 7/6/2015 | 727_D | L6-r96 | Human betaherpesvirus 6B | Herpesviridae | 0.91 | Na | 16 | 1161 | Na | KC465951:KY316036:NC_000898 | Virosaurus | DNA library |
| 25851 | 7/6/2015 | 727_R | L6-r735 | Human betaherpesvirus 6B | Herpesviridae | 3.55 | Na | 61 | 4500 | Na | KC465951:KY316042:NC_000898 | Virosaurus | RNA library |
| 25865 | 7/8/2015 | 728_R | L6-r735 | Human betaherpesvirus 6B | Herpesviridae | 2.36 | Na | 39 | 2995 | Na | KC465951:NC_000898 | Virosaurus | RNA library |
| 25874 | 7/9/2015 | 776_D | L4-r113 | Human betaherpesvirus 6B | Herpesviridae | 1.53 | Na | 53 | 1945 | Na | KC465951:NC_000898:NC_000898KY316037 | Virosaurus | DNA library |
| 25874 | 7/9/2015 | 776_R | L5-r737 | Human betaherpesvirus 6B | Herpesviridae | 2.31 | Na | 43 | 2935 | Na | AB075775:NC_000898:NC_000898KC465951 | Virosaurus | RNA library |
| 25886 | 7/10/2015 | 733_D | L5-r96 | Human betaherpesvirus 6B | Herpesviridae | 1.01 | Na | 25 | 1250 | Na | NC_000898 | Virosaurus | DNA library |
| 25886 | 7/10/2015 | 733_R | L2-r736 | Human betaherpesvirus 6B | Herpesviridae | 3.35 | Na | 66 | 4358 | Na | KC465951:KY316036:KY316037:NC_000898 | Virosaurus | RNA library |
| 25900 | 7/13/2015 | 738_D | L8-r93 | Human betaherpesvirus 6B | Herpesviridae | 0.7 | Na | 10 | 865 | Na | NC_000898 | Virosaurus | DNA library |
| 25900 | 7/13/2015 | 738_R | L3-r736 | Human betaherpesvirus 6B | Herpesviridae | 1.42 | Na | 23 | 1840 | Na | NC_000898 | Virosaurus | RNA library |
| 26015 | 8/4/2015 | 749_D | L7-r96 | Human betaherpesvirus 6B | Herpesviridae | 0.67 | Na | 20 | 832 | Na | KY316036:NC_000898 | Virosaurus | DNA library |
| 26015 | 8/4/2015 | 749_R | L6-r736 | Human betaherpesvirus 6B | Herpesviridae | 2.74 | Na | 47 | 3493 | Na | KC465951:NC_000898 | Virosaurus | RNA library |
| 26049 | 8/10/2015 | 754_D | L5-r96 | Human betaherpesvirus 6B | Herpesviridae | 4.16 | Na | 101 | 5273 | Na | KC465951:KY316032:NC_000898:NC_000898KC465951 | Virosaurus | DNA library |
| 26086 | 8/13/2015 | 759_R | L8-r736 | Human betaherpesvirus 6B | Herpesviridae | 18.28 | Na | 459 | 23888 | Na | AB075775:KC465951:KY316042:NC_000898 | Virosaurus | RNA library |
| 26124 | 8/21/2015 | 806_D | L6-r113 | Human betaherpesvirus 6B | Herpesviridae | 2.28 | Na | 47 | 2822 | Na | AB075775:KY316036:NC_000898 | Virosaurus | DNA library |
| 26140 | 8/25/2015 | 647_D | L3-r91 | Human betaherpesvirus 6B | Herpesviridae | 24.09 | Na | 771 | 31395 | Na | AB021506:AB075775:KC465951:KY316036:KY316042:NC_000898:U13194 | Virosaurus | DNA library |
| 26140 | 8/25/2015 | 647_R | L8-r724 | Human betaherpesvirus 6B | Herpesviridae | 1.09 | Na | 18 | 1384 | Na | NC_000898 | Virosaurus | RNA library |
| 26174 | 8/27/2015 | 820_D | L6-r122 | Human betaherpesvirus 6B | Herpesviridae | 4.32 | Na | 90 | 5474 | Na | KC465951:NC_000898:NC_000898KY316037 | Virosaurus | DNA library |
| 26174 | 8/27/2015 | 820_R | L1-r741 | Human betaherpesvirus 6B | Herpesviridae | 1.67 | Na | 30 | 2070 | Na | KC465951:NC_000898:NC_000898KC465951 | Virosaurus | RNA library |
| 26213 | 9/2/2015 | 834_D | L5-r122 | Human betaherpesvirus 6B | Herpesviridae | 0.26 | Na | 4 | 316 | Na | NC_000898 | Virosaurus | DNA library |
| 26213 | 9/2/2015 | 834_R | L4-r741 | Human betaherpesvirus 6B | Herpesviridae | 3.74 | Na | 65 | 4860 | Na | KC465951:NC_000898 | Virosaurus | RNA library |
| 26312 | 9/15/2015 | 884_D | L6-r132 | Human betaherpesvirus 6B | Herpesviridae | 0.34 | Na | 5 | 421 | Na | KC465951:NC_000898:NC_000898KY316037 | Virosaurus | DNA library |
| 26312 | 9/15/2015 | 884_R | L2-r746 | Human betaherpesvirus 6B | Herpesviridae | 10.85 | Na | 256 | 14229 | Na | AB075776:KC465951:NC_000898 | Virosaurus | RNA library |
| 26331 | 9/17/2015 | 888_D | L3-r132 | Human betaherpesvirus 6B | Herpesviridae | 3.51 | Na | 68 | 4456 | Na | KC465951:KY316036:NC_000898 | Virosaurus | DNA library |
| 26331 | 9/17/2015 | 888_R | L3-r746 | Human betaherpesvirus 6B | Herpesviridae | 1.46 | Na | 24 | 1810 | Na | NC_000898:NC_000898KC465951 | Virosaurus | RNA library |
| 26343 | 9/17/2015 | 894_D | L1-r132 | Human betaherpesvirus 6B | Herpesviridae | 3.93 | Na | 129 | 5128 | Na | KC465951:NC_000898 | Virosaurus | DNA library |
| 26343 | 9/17/2015 | 894_R | L4-r746 | Human betaherpesvirus 6B | Herpesviridae | 3.48 | Na | 69 | 4535 | Na | KC465951:KY316036:NC_000898 | Virosaurus | RNA library |
| 26455 | 11/2/2015 | 27_D | L006-r44 | Human betaherpesvirus 6B | Herpesviridae | 3.55 | Na | 82 | 4557 | Na | AB075775:AB075776:KC465951:NC_000898 | Virosaurus | DNA library |
| 26455 | 11/2/2015 | 27_R | L7-r669 | Human betaherpesvirus 6B | Herpesviridae | 3.31 | Na | 52 | 4188 | Na | KC465951:NC_000898 | Virosaurus | RNA library |
| 26493 | 11/12/2015 | 14_D | L004-r55 | Human betaherpesvirus 6B | Herpesviridae | 1.8 | Na | 47 | 2284 | Na | AB075775:NC_000898 | Virosaurus | DNA library |
| 26493 | 11/12/2015 | 14_R | L5-r669 | Human betaherpesvirus 6B | Herpesviridae | 0.83 | Na | 12 | 1058 | Na | KC465951:NC_000898 | Virosaurus | RNA library |
| 26507 | 11/17/2015 | 30_D | L001-r44 | Human betaherpesvirus 6B | Herpesviridae | 10.35 | Na | 238 | 13244 | Na | AB075775:AB075776:KC465951:NC_000898 | Virosaurus | DNA library |
| 26507 | 11/17/2015 | 30_R | L7-r669 | Human betaherpesvirus 6B | Herpesviridae | 2.32 | Na | 43 | 3022 | Na | KC465951:NC_000898 | Virosaurus | RNA library |
| 21053 | 2/6/2015 | 104_D | L007-r49 | Human betaherpesvirus 7 | Herpesviridae | 12.88 | Na | 316 | 12088 | Na | NC_001716 | Virosaurus | DNA library |
| 21053 | 2/6/2015 | 104_R | L004-r676 | Human betaherpesvirus 7 | Herpesviridae | 12.57 | Na | 200 | 11800 | Na | NC_001716 | Virosaurus | RNA library |
| 21105 | 3/2/2015 | 120_D | L001-r49 | Human betaherpesvirus 7 | Herpesviridae | 1.82 | Na | 21 | 1670 | Na | NC_001716 | Virosaurus | DNA library |
| 21105 | 3/2/2015 | 120_R | L008-r676 | Human betaherpesvirus 7 | Herpesviridae | 16.74 | Na | 260 | 16028 | Na | NC_001716 | Virosaurus | RNA library |
| 21122 | 3/5/2015 | 124_D | L006-r49 | Human betaherpesvirus 7 | Herpesviridae | 17.51 | Na | 286 | 16763 | Na | NC_001716 | Virosaurus | DNA library |
| 21122 | 3/5/2015 | 124_R | L001-r677 | Human betaherpesvirus 7 | Herpesviridae | 2.78 | Na | 31 | 2606 | Na | NC_001716 | Virosaurus | RNA library |
| 21158 | 3/17/2015 | 127_D | L001-r49 | Human betaherpesvirus 7 | Herpesviridae | 1.79 | Na | 34 | 1682 | Na | NC_001716 | Virosaurus | DNA library |
| 21195 | 12/11/2015 | 60_D | L008-r46 | Human betaherpesvirus 7 | Herpesviridae | 6.42 | Na | 103 | 6027 | Na | NC_001716 | Virosaurus | DNA library |
| 21201 | 3/23/2015 | 67_D | L008-r46 | Human betaherpesvirus 7 | Herpesviridae | 47.26 | Na | 1269 | 45253 | Na | NC_001716 | Virosaurus | DNA library |
| 21201 | 3/23/2015 | 67_R | L002-r675 | Human betaherpesvirus 7 | Herpesviridae | 18.67 | Na | 289 | 17871 | Na | NC_001716 | Virosaurus | RNA library |
| 21437 | 5/22/2015 | 774_D | L7-r111 | Human betaherpesvirus 7 | Herpesviridae | 4.32 | Na | 66 | 3976 | Na | NC_001716 | Virosaurus | DNA library |
| 21437 | 5/22/2015 | 774_R | L5-r737 | Human betaherpesvirus 7 | Herpesviridae | 5.16 | Na | 70 | 4745 | Na | NC_001716 | Virosaurus | RNA library |
| 21449 | 5/25/2015 | 250_D | L004-r61 | Human betaherpesvirus 7 | Herpesviridae | 2.5 | Na | 31 | 2301 | Na | NC_001716 | Virosaurus | DNA library |
| 21449 | 5/25/2015 | 250_R | L008-r691 | Human betaherpesvirus 7 | Herpesviridae | 0.81 | Na | 10 | 741 | Na | NC_001716 | Virosaurus | RNA library |
| 21521 | 6/4/2015 | 268_D | L008-r61 | Human betaherpesvirus 7 | Herpesviridae | 10.21 | Na | 147 | 9773 | Na | NC_001716 | Virosaurus | DNA library |
| 21521 | 6/4/2015 | 268_R | L004-r692 | Human betaherpesvirus 7 | Herpesviridae | 7.38 | Na | 97 | 7067 | Na | NC_001716 | Virosaurus | RNA library |
| 21556 | 6/10/2015 | 276_D | L6-r65 | Human betaherpesvirus 7 | Herpesviridae | 55.55 | Na | 1342 | 53192 | Na | NC_001716 | Virosaurus | DNA library |
| 21556 | 6/10/2015 | 276_R | L007-r692 | Human betaherpesvirus 7 | Herpesviridae | 21.14 | Na | 322 | 20242 | Na | NC_001716 | Virosaurus | RNA library |
| 21812 | 7/28/2015 | 313_D | L007-r67 | Human betaherpesvirus 7 | Herpesviridae | 0.38 | Na | 4 | 347 | Na | NC_001716 | Virosaurus | DNA library |
| 21838 | 8/3/2015 | 316_D | L1-r66 | Human betaherpesvirus 7 | Herpesviridae | 5.17 | Na | 70 | 4853 | Na | NC_001716 | Virosaurus | DNA library |
| 21838 | 8/3/2015 | 316_R | L002-r698 | Human betaherpesvirus 7 | Herpesviridae | 22.65 | Na | 392 | 21691 | Na | NC_001716 | Virosaurus | RNA library |
| 21937 | 8/18/2015 | 330_D | L8-r66 | Human betaherpesvirus 7 | Herpesviridae | 0.67 | Na | 7 | 612 | Na | NC_001716 | Virosaurus | DNA library |
| 21937 | 8/18/2015 | 330_R | L005-r698 | Human betaherpesvirus 7 | Herpesviridae | 1.6 | Na | 20 | 1500 | Na | NC_001716 | Virosaurus | RNA library |
| 21938 | 8/18/2015 | 331_D | L1-r66 | Human betaherpesvirus 7 | Herpesviridae | 3.01 | Na | 35 | 2769 | Na | NC_001716 | Virosaurus | DNA library |
| 21938 | 8/18/2015 | 331_R | L005-r698 | Human betaherpesvirus 7 | Herpesviridae | 12.55 | Na | 195 | 11782 | Na | NC_001716 | Virosaurus | RNA library |
| 21982 | 8/24/2015 | 336_D | L7-r66 | Human betaherpesvirus 7 | Herpesviridae | 3.44 | Na | 56 | 3226 | Na | NC_001716 | Virosaurus | DNA library |
| 21982 | 8/24/2015 | 336_R | L007-r698 | Human betaherpesvirus 7 | Herpesviridae | 2.14 | Na | 25 | 2007 | Na | NC_001716 | Virosaurus | RNA library |
| 21987 | 8/25/2015 | 337_D | L8-r66 | Human betaherpesvirus 7 | Herpesviridae | 17 | Na | 257 | 15959 | Na | NC_001716 | Virosaurus | DNA library |
| 21987 | 8/25/2015 | 337_R | L007-r698 | Human betaherpesvirus 7 | Herpesviridae | 34.24 | Na | 630 | 32790 | Na | NC_001716 | Virosaurus | RNA library |
| 21988 | 8/25/2015 | 338_R | L007-r698 | Human betaherpesvirus 7 | Herpesviridae | 1.62 | Na | 19 | 1485 | Na | NC_001716 | Virosaurus | RNA library |
| 22408 | 10/23/2015 | 395_D | L003-r73 | Human betaherpesvirus 7 | Herpesviridae | 0.36 | Na | 6 | 331 | Na | NC_001716 | Virosaurus | DNA library |
| 22423 | 11/2/2015 | 38_D | L002-r44 | Human betaherpesvirus 7 | Herpesviridae | 16.71 | Na | 283 | 15996 | Na | NC_001716 | Virosaurus | DNA library |
| 22423 | 11/2/2015 | 38_R | L004-r674 | Human betaherpesvirus 7 | Herpesviridae | 3.68 | Na | 43 | 3386 | Na | NC_001716 | Virosaurus | RNA library |
| 22603 | 12/3/2015 | 71_D | L005-r46 | Human betaherpesvirus 7 | Herpesviridae | 4.08 | Na | 56 | 3827 | Na | NC_001716 | Virosaurus | DNA library |
| 22603 | 12/3/2015 | 71_R | L003-r675 | Human betaherpesvirus 7 | Herpesviridae | 0.47 | Na | 6 | 430 | Na | NC_001716 | Virosaurus | RNA library |
| 25194 | 3/12/2015 | 452_D | L001-r77 | Human betaherpesvirus 7 | Herpesviridae | 52.12 | Na | 970 | 49911 | Na | NC_001716 | Virosaurus | DNA library |
| 25194 | 3/12/2015 | 452_R | L006-r705 | Human betaherpesvirus 7 | Herpesviridae | 21.4 | Na | 343 | 20496 | Na | NC_001716 | Virosaurus | RNA library |
| 25212 | 3/18/2015 | 459_D | L001-r77 | Human betaherpesvirus 7 | Herpesviridae | 8.27 | Na | 149 | 7606 | Na | NC_001716 | Virosaurus | DNA library |
| 25212 | 3/18/2015 | 459_R | L008-r705 | Human betaherpesvirus 7 | Herpesviridae | 5.71 | Na | 75 | 5363 | Na | NC_001716 | Virosaurus | RNA library |
| 25425 | 5/6/2015 | 502_D | L005-r84 | Human betaherpesvirus 7 | Herpesviridae | 16.92 | Na | 279 | 15879 | Na | NC_001716 | Virosaurus | DNA library |
| 25425 | 5/6/2015 | 502_R | L7-r713 | Human betaherpesvirus 7 | Herpesviridae | 1.15 | Na | 13 | 1061 | Na | NC_001716 | Virosaurus | RNA library |
| 25438 | 5/7/2015 | 507_D | L5-r82 | Human betaherpesvirus 7 | Herpesviridae | 62.87 | Na | 1458 | 60200 | Na | NC_001716 | Virosaurus | DNA library |
| 25438 | 5/7/2015 | 507_R | L8-r713 | Human betaherpesvirus 7 | Herpesviridae | 3.08 | Na | 43 | 2890 | Na | NC_001716 | Virosaurus | RNA library |
| 25606 | 5/29/2015 | 549_D | L2-r85 | Human betaherpesvirus 7 | Herpesviridae | 7.82 | Na | 183 | 7338 | Na | NC_001716 | Virosaurus | DNA library |
| 25606 | 5/29/2015 | 549_R | L4-r717 | Human betaherpesvirus 7 | Herpesviridae | 11.06 | Na | 246 | 10591 | Na | NC_001716 | Virosaurus | RNA library |
| 25779 | 6/25/2015 | 700_R | L7-r734 | Human betaherpesvirus 7 | Herpesviridae | 4.55 | Na | 61 | 4182 | Na | NC_001716 | Virosaurus | RNA library |
| 25784 | 6/25/2015 | 701_D | L1-r93 | Human betaherpesvirus 7 | Herpesviridae | 0.89 | Na | 10 | 819 | Na | NC_001716 | Virosaurus | DNA library |
| 25786 | 6/24/2015 | 702_D | L2-r93 | Human betaherpesvirus 7 | Herpesviridae | 8.26 | Na | 119 | 7753 | Na | NC_001716 | Virosaurus | DNA library |
| 25786 | 6/24/2015 | 702_R | L7-r734 | Human betaherpesvirus 7 | Herpesviridae | 23.16 | Na | 385 | 22180 | Na | NC_001716 | Virosaurus | RNA library |
| 25807 | 6/29/2015 | 711_D | L4-r93 | Human betaherpesvirus 7 | Herpesviridae | 9.87 | Na | 160 | 9258 | Na | NC_001716 | Virosaurus | DNA library |
| 25807 | 6/29/2015 | 711_R | L1-r735 | Human betaherpesvirus 7 | Herpesviridae | 20.78 | Na | 327 | 19894 | Na | NC_001716 | Virosaurus | RNA library |
| 25843 | 7/3/2015 | 725_D | L4-r96 | Human betaherpesvirus 7 | Herpesviridae | 47.9 | Na | 1016 | 45868 | Na | NC_001716 | Virosaurus | DNA library |
| 25843 | 7/3/2015 | 725_R | L6-r735 | Human betaherpesvirus 7 | Herpesviridae | 47.78 | Na | 1109 | 45758 | Na | NC_001716 | Virosaurus | RNA library |
| 25876 | 7/9/2015 | 729_D | L6-r93 | Human betaherpesvirus 7 | Herpesviridae | 14.17 | Na | 323 | 13299 | Na | NC_001716 | Virosaurus | DNA library |
| 25980 | 7/28/2015 | 744_D | L6-r93 | Human betaherpesvirus 7 | Herpesviridae | 8.37 | Na | 155 | 7852 | Na | NC_001716 | Virosaurus | DNA library |
| 25980 | 7/28/2015 | 744_R | L4-r736 | Human betaherpesvirus 7 | Herpesviridae | 8.78 | Na | 127 | 8238 | Na | NC_001716 | Virosaurus | RNA library |
| 26145 | 8/25/2015 | 812_D | L5-r122 | Human betaherpesvirus 7 | Herpesviridae | 52.51 | Na | 1045 | 50286 | Na | NC_001716 | Virosaurus | DNA library |
| 26145 | 8/25/2015 | 812_R | L7-r740 | Human betaherpesvirus 7 | Herpesviridae | 1.58 | Na | 19 | 1450 | Na | NC_001716 | Virosaurus | RNA library |
| 26340 | 9/17/2015 | 892_D | L8-r124 | Human betaherpesvirus 7 | Herpesviridae | 6.83 | Na | 94 | 6409 | Na | NC_001716 | Virosaurus | DNA library |
| 26340 | 9/17/2015 | 892_R | L4-r746 | Human betaherpesvirus 7 | Herpesviridae | 15.06 | Na | 229 | 14141 | Na | NC_001716 | Virosaurus | RNA library |
| 26342 | 9/17/2015 | 893_D | L8-r127 | Human betaherpesvirus 7 | Herpesviridae | 20.54 | Na | 371 | 19672 | Na | NC_001716 | Virosaurus | DNA library |
| 26342 | 9/17/2015 | 893_R | L4-r746 | Human betaherpesvirus 7 | Herpesviridae | 16.97 | Na | 307 | 16245 | Na | NC_001716 | Virosaurus | RNA library |
| 26558 | 12/4/2015 | 74_D | L008-r46 | Human betaherpesvirus 7 | Herpesviridae | 15.45 | Na | 249 | 14792 | Na | NC_001716 | Virosaurus | DNA library |
| 26558 | 12/4/2015 | 74_R | L004-r675 | Human betaherpesvirus 7 | Herpesviridae | 1.83 | Na | 25 | 1687 | Na | NC_001716 | Virosaurus | RNA library |
| 22172 | 9/16/2015 | 358_D | L8-r66 | Human bocavirus 2 | Parvoviridae | 27.07 | 2 | 33 | 1437 | 5308 | JX257046 | Virosaurus | DNA library |
| 22277 | 10/12/2015 | 782_D | L8-r111 | Human bocavirus 2 | Parvoviridae | 28.64 | 1 | 35 | 1520 | 5308 | JX257046 | Virosaurus | DNA library |
| 22409 | 10/23/2015 | 396_D | L004-r73 | Human bocavirus 2 | Parvoviridae | 8.74 | 1 | 7 | 464 | 5308 | JX257046 | Virosaurus | DNA library |
| 25923 | 7/16/2015 | 867_D | L3-r132 | Human bocavirus 2 | Parvoviridae | 40.28 | 2 | 61 | 2138 | 5308 | JX257046 | Virosaurus | DNA library |
| 26293 | 9/14/2015 | 877_D | L6-r132 | Human bocavirus 2 | Parvoviridae | 18.61 | 1 | 16 | 988 | 5308 | JX257046 | Virosaurus | DNA library |
| 22095 | 9/8/2015 | 860_D | L3-r132 | Human bocavirus1 or 3 | Parvoviridae | 97.67 | 19 | 1263 | 5415 | 5544 | JQ923422 | Virosaurus | DNA library |
| 25613 | 6/1/2015 | 554_D | L7-r85 | Human bocavirus1 or 3 | Parvoviridae | 77.11 | 2 | 130 | 4275 | 5544 | JQ923422 | Virosaurus | DNA library |
| 25675 | 6/9/2015 | 571_D | L2-r85 | Human bocavirus1 or 3 | Parvoviridae | 6.5 | 1 | 7 | 346 | 5320 | JN086998 | Virosaurus | DNA library |
| 25851 | 7/6/2015 | 727_D | L6-r96 | Human bocavirus1 or 3 | Parvoviridae | 18.15 | 1 | 17 | 1006 | 5544 | JQ923422 | Virosaurus | DNA library |
| 26086 | 8/13/2015 | 759_R | L8-r736 | Human bocavirus1 or 3 | Parvoviridae | 13.51 | 1 | 16 | 749 | 5544 | JQ923422 | Virosaurus | RNA library |
| 22671 | 1/29/2016 | 406_R | L002-r704 | Human coronavirus OC43 | Coronaviridae | 2.04 | 1 | 10 | 628 | 30726 | KY369907 | Virosaurus | RNA library |
| 25026 | 1/21/2015 | 414_R | L004-r704 | Human coronavirus OC43 | Coronaviridae | 1.73 | 1 | 7 | 530 | 30726 | KY369907 | Virosaurus | RNA library |
| 25787 | 6/25/2015 | 703_D | L3-r93 | Human CSF-associated densovirus | Parvoviridae | 12.76 | 1 | 7 | 597 | 4679 | KX035107 | Virosaurus | DNA library |
| 22462 | 11/18/2015 | 35_D | L007-r44 | Human gammaherpesvirus 8 | Herpesviridae | 9.3 | Na | 138 | 9119 | Na | AF091347:AF117253:JX228174:NC_009333 | Virosaurus | DNA library |
| 22462 | 11/18/2015 | 35_R | L001-r674 | Human gammaherpesvirus 8 | Herpesviridae | 1.56 | Na | 20 | 1495 | Na | AF091349:AF117253:JX228174:NC_009333 | Virosaurus | RNA library |
| 21568 | 6/12/2015 | 279_R | L008-r692 | Human immunodeficiency virus 1 | Retroviridae | 25.3 | 2 | 64 | 2539 | 10036 | AF133821 | Virosaurus | RNA library |
| 21622 | 6/24/2015 | 289_R | L002-r693 | Human immunodeficiency virus 1 | Retroviridae | 33.09 | 3 | 142 | 3206 | 9689 | AB485650 | Virosaurus | RNA library |
| 21819 | 7/30/2015 | 314_R | L008-r693 | Human immunodeficiency virus 1 | Retroviridae | 7.18 | 1 | 12 | 658 | 9165 | AY322190 | Virosaurus | RNA library |
| 22321 | 10/20/2015 | 388_R | L004-r703 | Human immunodeficiency virus 1 | Retroviridae | 61.34 | 26 | 13134 | 5472 | 8921 | EU110086 | Virosaurus | RNA library |
| 22462 | 11/18/2015 | 35_R | L001-r674 | Human immunodeficiency virus 1 | Retroviridae | 49.73 | 7 | 1350 | 4482 | 9013 | AF286235 | Virosaurus | RNA library |
| 25838 | 7/2/2015 | 721_R | L5-r735 | Human immunodeficiency virus 1 | Retroviridae | 48.7 | 4 | 315 | 4887 | 10036 | AF133821 | Virosaurus | RNA library |
| 26546 | 11/30/2015 | 70_R | L003-r675 | Human immunodeficiency virus 1 | Retroviridae | 17.7 | 2 | 42 | 1573 | 8889 | EU110093 | Virosaurus | RNA library |
| 22488 | 11/26/2015 | 47_R | L005-r674 | Human mastadenovirus A | Adenoviridae | 1.66 | 2 | 10 | 566 | 34126 | NC_001460 | Virosaurus | RNA library |
| 25783 | 6/25/2015 | 607_D | L2-r92 | Human mastadenovirus A | Adenoviridae | 64.74 | 3 | 931 | 22128 | 34178 | GU191019 | Virosaurus | DNA library |
| 25783 | 6/25/2015 | 607_R | L005-r723 | Human mastadenovirus A | Adenoviridae | 0.88 | 1 | 3 | 300 | 34178 | GU191019 | Virosaurus | RNA library |
| 21339 | 5/12/2015 | 230_D | L005-r61 | Human mastadenovirus C | Adenoviridae | 8.75 | 1 | 83 | 3145 | 35937 | JX173080 | Virosaurus | DNA library |
| 21567 | 6/12/2015 | 278_D | L007-r67 | Human mastadenovirus C | Adenoviridae | 22.4 | 2 | 195 | 8050 | 35936 | M73260 | Virosaurus | DNA library |
| 21567 | 6/12/2015 | 278_R | L007-r692 | Human mastadenovirus C | Adenoviridae | 1.94 | 1 | 11 | 697 | 36007 | JX173085 | Virosaurus | RNA library |
| 21858 | 8/5/2015 | 321_D | L6-r66 | Human mastadenovirus C | Adenoviridae | 1.45 | 1 | 8 | 521 | 35959 | MF315029 | Virosaurus | DNA library |
| 21884 | 8/7/2015 | 325_D | L3-r66 | Human mastadenovirus C | Adenoviridae | 2.13 | 1 | 8 | 762 | 35819 | HQ003817 | Virosaurus | DNA library |
| 22196 | 9/18/2015 | 362_D | L002-r71 | Human mastadenovirus C | Adenoviridae | 2.44 | 1 | 12 | 877 | 36007 | JX173085 | Virosaurus | DNA library |
| 22246 | 9/28/2015 | 371_D | L004-r71 | Human mastadenovirus C | Adenoviridae | 1.44 | 1 | 8 | 515 | 35760 | HQ413315 | Virosaurus | DNA library |
| 22382 | 11/18/2015 | 391_D | L002-r71 | Human mastadenovirus C | Adenoviridae | 2.51 | 1 | 12 | 905 | 36007 | JX173085 | Virosaurus | DNA library |
| 25055 | 1/30/2015 | 418_D | L004-r75 | Human mastadenovirus C | Adenoviridae | 2.5 | 1 | 10 | 895 | 35760 | HQ413315 | Virosaurus | DNA library |
| 25163 | 3/5/2015 | 446_D | L004-r75 | Human mastadenovirus C | Adenoviridae | 66.61 | 3 | 867 | 23936 | 35936 | M73260 | Virosaurus | DNA library |
| 25329 | 4/16/2015 | 484_D | L005-r77 | Human mastadenovirus C | Adenoviridae | 16.47 | 1 | 141 | 5924 | 35959 | MF315029 | Virosaurus | DNA library |
| 26078 | 8/13/2015 | 642_D | L5-r91 | Human mastadenovirus C | Adenoviridae | 1.36 | 1 | 9 | 489 | 36007 | JX173085 | Virosaurus | DNA library |
| 26173 | 8/27/2015 | 819_R | L1-r741 | Human mastadenovirus C | Adenoviridae | 1.21 | 1 | 8 | 434 | 35760 | HQ413315 | Virosaurus | RNA library |
| 26224 | 9/4/2015 | 839_D | L7-r124 | Human mastadenovirus C | Adenoviridae | 5.66 | 1 | 33 | 2024 | 35760 | HQ413315 | Virosaurus | DNA library |
| 26299 | 9/14/2015 | 880_D | L2-r132 | Human mastadenovirus C | Adenoviridae | 1.5 | 1 | 11 | 540 | 36007 | JX173085 | Virosaurus | DNA library |
| 26309 | 9/15/2015 | 882_R | L1-r746 | Human mastadenovirus C | Adenoviridae | 1.87 | 1 | 14 | 671 | 35937 | JX173080 | Virosaurus | RNA library |
| 26241 | 9/7/2015 | 675_R | L7-r725 | Human mastadenovirus E | Adenoviridae | 0.83 | 1 | 3 | 300 | 35968 | KX384951 | Virosaurus | RNA library |
| 21851 | 8/4/2015 | 320_D | L5-r66 | Human mastadenovirus F | Adenoviridae | 1.46 | 3 | 12 | 499 | 34199 | KY316161 | Virosaurus | DNA library |
| 22107 | 9/9/2015 | 352_D | L1-r66 | Human mastadenovirus F | Adenoviridae | 1.6 | 1 | 7 | 547 | 34166 | KY316162 | Virosaurus | DNA library |
| 22195 | 9/18/2015 | 361_D | L001-r71 | Human mastadenovirus F | Adenoviridae | 1.66 | 1 | 8 | 569 | 34215 | NC_001454 | Virosaurus | DNA library |
| 22195 | 9/18/2015 | 361_R | L006-r702 | Human mastadenovirus F | Adenoviridae | 1.01 | 1 | 6 | 344 | 34166 | KY316162 | Virosaurus | RNA library |
| 25179 | 3/9/2015 | 450_D | L006-r73 | Human mastadenovirus F | Adenoviridae | 1.87 | 1 | 10 | 641 | 34215 | NC_001454 | Virosaurus | DNA library |
| 25289 | 4/10/2015 | 477_D | L005-r77 | Human mastadenovirus F | Adenoviridae | 0.93 | 1 | 8 | 318 | 34166 | KY316162 | Virosaurus | DNA library |
| 25761 | 6/23/2015 | 601_D | L7-r88 | Human mastadenovirus F | Adenoviridae | 58.92 | 2 | 543 | 20158 | 34215 | NC_001454 | Virosaurus | DNA library |
| 25761 | 6/23/2015 | 601_R | L003-r723 | Human mastadenovirus F | Adenoviridae | 2.12 | 1 | 10 | 725 | 34215 | NC_001454 | Virosaurus | RNA library |
| 26004 | 8/3/2015 | 634_D | L4-r91 | Human mastadenovirus F | Adenoviridae | 11.94 | 1 | 84 | 4085 | 34215 | NC_001454 | Virosaurus | DNA library |
| 26263 | 9/9/2015 | 854_R | L1-r745 | Human mastadenovirus F | Adenoviridae | 1.9 | 1 | 13 | 650 | 34166 | KY316162 | Virosaurus | RNA library |
| 26299 | 9/14/2015 | 880_D | L2-r132 | Human mastadenovirus F | Adenoviridae | 0.9 | 1 | 4 | 307 | 34199 | KY316161 | Virosaurus | DNA library |
| 26558 | 12/4/2015 | 74_D | L008-r46 | Human mastadenovirus F | Adenoviridae | 1.44 | 1 | 7 | 494 | 34215 | NC_001454 | Virosaurus | DNA library |
| 25807 | 6/29/2015 | 711_D | L4-r93 | Human polyomavirus 10 | Polyomaviridae | 8.12 | 1 | 6 | 401 | 4940 | JQ898292 | Virosaurus | DNA library |
| 22628 | 1/11/2016 | 793_R | L1-r740 | Iflavirus | Iflaviridae | 100 | 539 | 53038 | 10003 | 10003 | MN727203 | De novo | RNA library |
| 21042 | 2/3/2015 | 101_R | L003-r676 | Mamastrovirus 1 [Classical] | Astroviridae | 30.84 | 1 | 30 | 2058 | 6674 | NC_030922 | Virosaurus | RNA library |
| 21102 | 2/27/2015 | 118_R | L7-r735 | Mamastrovirus 1 [Classical] | Astroviridae | 29.49 | 1 | 27 | 1968 | 6674 | NC_030922 | Virosaurus | RNA library |
| 21780 | 7/24/2015 | 626_R | L2-r724 | Mamastrovirus 1 [Classical] | Astroviridae | 8.03 | 1 | 6 | 541 | 6734 | KF039912 | Virosaurus | RNA library |
| 25671 | 6/8/2015 | 569_R | L001-r722 | Mamastrovirus 1 [Classical] | Astroviridae | 95.47 | 51 | 3987 | 6470 | 6777 | KP862744 | Virosaurus | RNA library |
| 25762 | 6/23/2015 | 600_R | L003-r723 | Mamastrovirus 1 [Classical] | Astroviridae | 5.89 | 1 | 6 | 392 | 6654 | KY271946 | Virosaurus | RNA library |
| 25792 | 6/26/2015 | 707_R | L8-r734 | Mamastrovirus 1 [Classical] | Astroviridae | 5.59 | 1 | 6 | 379 | 6779 | KF039911 | Virosaurus | RNA library |
| 25883 | 7/10/2015 | 731_R | L1-r736 | Mamastrovirus 1 [Classical] | Astroviridae | 80.93 | 2 | 175 | 5486 | 6779 | KF039911 | Virosaurus | RNA library |
| 21039 | 2/2/2015 | 99_R | L003-r676 | Mamastrovirus 6 [Novel] | Astroviridae | 30.49 | 1 | 29 | 1891 | 6202 | AB823731 | Virosaurus | RNA library |
| 21718 | 7/10/2015 | 304_R | L006-r693 | Mamastrovirus 6 [Novel] | Astroviridae | 4.89 | 1 | 4 | 303 | 6202 | AB823731 | Virosaurus | RNA library |
| 21871 | 8/6/2015 | 323_R | L003-r698 | Mamastrovirus 6 [Novel] | Astroviridae | 20.91 | 1 | 20 | 1297 | 6202 | AB823731 | Virosaurus | RNA library |
| 22462 | 11/18/2015 | 35_R | L001-r674 | Mamastrovirus 6 [Novel] | Astroviridae | 95.25 | 4 | 316 | 5857 | 6149 | AB829252 | Virosaurus | RNA library |
| 25145 | 3/2/2015 | 438_R | L002-r705 | Mamastrovirus 6 [Novel] | Astroviridae | 9.12 | 1 | 8 | 561 | 6149 | AB829252 | Virosaurus | RNA library |
| 26254 | 9/8/2015 | 849_R | L8-r741 | Mamastrovirus 6 [Novel] | Astroviridae | 11.75 | 1 | 18 | 729 | 6202 | AB823731 | Virosaurus | RNA library |
| 21294 | 5/5/2015 | 224_R | L007-r690 | Mamastrovirus 9 [Novel] | Astroviridae | 7.28 | 1 | 6 | 479 | 6582 | NC_019026 | Virosaurus | RNA library |
| 21526 | 6/4/2015 | 270_R | L005-r692 | Mamastrovirus 9 [Novel] | Astroviridae | 50.9 | 2 | 73 | 3353 | 6587 | NC_013060 | Virosaurus | RNA library |
| 21721 | 7/10/2015 | 305_R | L006-r693 | Mamastrovirus 9 [Novel] | Astroviridae | 68.35 | 1 | 98 | 4502 | 6587 | NC_013060 | Virosaurus | RNA library |
| 22038 | 9/1/2015 | 341_R | L008-r698 | Mamastrovirus 9 [Novel] | Astroviridae | 29.22 | 1 | 28 | 1925 | 6587 | NC_013060 | Virosaurus | RNA library |
| 25273 | 4/9/2015 | 472_R | L7-r707 | Mamastrovirus 9 [Novel] | Astroviridae | 93.11 | 3 | 262 | 6133 | 6587 | NC_013060 | Virosaurus | RNA library |
| 25282 | 4/10/2015 | 474_R | L7-r707 | Mamastrovirus 9 [Novel] | Astroviridae | 37.31 | 1 | 36 | 2456 | 6582 | NC_019026 | Virosaurus | RNA library |
| 25729 | 6/17/2015 | 685_R | L3-r734 | Mamastrovirus 9 [Novel] | Astroviridae | 21.85 | 1 | 23 | 1438 | 6582 | NC_019026 | Virosaurus | RNA library |
| 26083 | 8/13/2015 | 802_R | L4-r740 | Mamastrovirus 9 [Novel] | Astroviridae | 98.59 | 7 | 592 | 6494 | 6587 | NC_013060 | Virosaurus | RNA library |
| 26141 | 8/25/2015 | 646_R | L7-r724 | Mamastrovirus 9 [Novel] | Astroviridae | 19.78 | 1 | 19 | 1302 | 6582 | NC_019026 | Virosaurus | RNA library |
| 26145 | 8/25/2015 | 812_R | L7-r740 | Mamastrovirus 9 [Novel] | Astroviridae | 6.06 | 1 | 6 | 399 | 6582 | NC_019026 | Virosaurus | RNA library |
| 26219 | 9/3/2015 | 836_R | L5-r741 | Mamastrovirus 9 [Novel] | Astroviridae | 10.97 | 1 | 9 | 722 | 6582 | NC_019026 | Virosaurus | RNA library |
| 25569 | 5/26/2015 | 531_R | L6-r714 | Mammalian orthoreovirus | Reoviridae | 5.76 | Na | 23 | 1125 | 19526 | DQ885990:JN799423:JX486057:LC121914 | Virosaurus | RNA library |
| 21017 | 1/23/2015 | 766_R | L3-r737 | Norwalk virus [Genogroupe II] | Caliciviridae | 59.93 | 3 | 148 | 4544 | 7582 | KU935739 | Virosaurus | RNA library |
| 21110 | 3/3/2015 | 121_R | L008-r676 | Norwalk virus [Genogroupe II] | Caliciviridae | 5.79 | 1 | 7 | 439 | 7582 | KU935739 | Virosaurus | RNA library |
| 21253 | 4/13/2015 | 216_R | L005-r690 | Norwalk virus [Genogroupe II] | Caliciviridae | 83.53 | 318 | 21460 | 6248 | 7480 | JN400618 | Virosaurus | RNA library |
| 21291 | 4/27/2015 | 223_R | L007-r690 | Norwalk virus [Genogroupe II] | Caliciviridae | 5.09 | 2 | 10 | 387 | 7606 | KF306214 | Virosaurus | RNA library |
| 21350 | 5/13/2015 | 233_R | L003-r691 | Norwalk virus [Genogroupe II] | Caliciviridae | 5.07 | 2 | 24 | 382 | 7537 | LC209439 | Virosaurus | RNA library |
| 21949 | 8/19/2015 | 333_R | L006-r698 | Norwalk virus [Genogroupe II] | Caliciviridae | 19.47 | 1 | 20 | 1456 | 7480 | JN400618 | Virosaurus | RNA library |
| 22290 | 10/16/2015 | 379_R | L002-r703 | Norwalk virus [Genogroupe II] | Caliciviridae | 10.51 | 1 | 16 | 783 | 7453 | AB972472 | Virosaurus | RNA library |
| 22292 | 10/19/2015 | 380_R | L002-r703 | Norwalk virus [Genogroupe II] | Caliciviridae | 4.33 | 2 | 5 | 323 | 7453 | AB972472 | Virosaurus | RNA library |
| 25220 | 3/19/2015 | 463_R | L5-r707 | Norwalk virus [Genogroupe II] | Caliciviridae | 9.88 | 1 | 10 | 745 | 7544 | KJ196295 | Virosaurus | RNA library |
| 25766 | 6/24/2015 | 696_R | L6-r734 | Norwalk virus [Genogroupe II] | Caliciviridae | 47.66 | 2 | 106 | 3565 | 7480 | JN400618 | Virosaurus | RNA library |
| 26261 | 9/9/2015 | 654_R | L1-r725 | Norwalk virus [Genogroupe II] | Caliciviridae | 4.22 | 1 | 4 | 318 | 7537 | LC209439 | Virosaurus | RNA library |
| 26524 | 11/23/2015 | 43_R | L003-r674 | Norwalk virus [Genogroupe II] | Caliciviridae | 5.28 | 1 | 4 | 395 | 7480 | JN400618 | Virosaurus | RNA library |
| 21332 | 5/11/2015 | 228_R | L002-r691 | Parechovirus A | Picornaviridae | 13.86 | 1 | 16 | 1012 | 7303 | GQ183034 | Virosaurus | RNA library |
| 22179 | 9/17/2015 | 667_R | L5-r725 | Parechovirus A | Picornaviridae | 4.39 | 1 | 6 | 320 | 7295 | GQ183029 | Virosaurus | RNA library |
| 25004 | 1/15/2015 | 408_R | L002-r704 | Parechovirus A | Picornaviridae | 4.09 | 1 | 3 | 300 | 7330 | KC769584 | Virosaurus | RNA library |
| 25107 | 2/18/2015 | 430_R | L008-r704 | Parechovirus A | Picornaviridae | 10.11 | 2 | 22 | 746 | 7381 | FM178558 | Virosaurus | RNA library |
| 25267 | 4/9/2015 | 471_R | L7-r707 | Parechovirus A | Picornaviridae | 8.94 | 1 | 13 | 648 | 7250 | HM996978 | Virosaurus | RNA library |
| 25604 | 5/29/2015 | 548_R | L3-r717 | Parechovirus A | Picornaviridae | 4.23 | 1 | 5 | 312 | 7381 | FM178558 | Virosaurus | RNA library |
| 25788 | 6/25/2015 | 704_R | L8-r734 | Parechovirus A | Picornaviridae | 12.67 | 1 | 14 | 931 | 7348 | AB252582 | Virosaurus | RNA library |
| 26004 | 8/3/2015 | 634_R | L4-r724 | Parechovirus A | Picornaviridae | 4.68 | 1 | 5 | 339 | 7250 | HM996978 | Virosaurus | RNA library |
| 26330 | 9/17/2015 | 887_R | L2-r746 | Parechovirus A | Picornaviridae | 10.45 | 1 | 8 | 763 | 7303 | GQ183034 | Virosaurus | RNA library |
| 21234 | 4/8/2015 | 214_R | L005-r690 | Phlebovirus-like_virus | Phenuiviridae | 10.33 | Na | 22 | 1194 | 11559 | MN062090:MN062091 | De novo | RNA library |
| 21082 | 2/20/2015 | 110_R | L005-r676 | Picorna-like | Unknown | 100 | 376 | 42384 | 11235 | 11235 | MN727199 | De novo | RNA library |
| 21085 | 2/23/2015 | 112_R | L006-r676 | Picorna-like | Unknown | 13.96 | 3 | 65 | 1568 | 11235 | MN727199 | De novo | RNA library |
| 22259 | 9/29/2015 | 374_R | L001-r703 | Picorna-like | Unknown | 86.77 | 40 | 4067 | 9748 | 11235 | MN727199 | De novo | RNA library |
| 22432 | 11/4/2015 | 398_R | L007-r703 | Picorna-like | Unknown | 23.85 | 9 | 314 | 2679 | 11235 | MN727199 | De novo | RNA library |
| 22453 | 11/16/2015 | 400_R | L007-r703 | Picorna-like | Unknown | 30.9 | 5 | 222 | 3472 | 11235 | MN727199 | De novo | RNA library |
| 25003 | 12/11/2014 | 407_R | L002-r704 | Picorna-like | Unknown | 22.07 | 14 | 425 | 2480 | 11235 | MN727199 | De novo | RNA library |
| 25008 | 1/15/2015 | 409_R | L002-r704 | Picorna-like | Unknown | 3.6 | 5 | 35 | 404 | 11235 | MN727199 | De novo | RNA library |
| 25026 | 1/21/2015 | 414_R | L004-r704 | Picorna-like | Unknown | 19.22 | 18 | 479 | 2159 | 11235 | MN727199 | De novo | RNA library |
| 25033 | 1/22/2015 | 415_R | L004-r704 | Picorna-like | Unknown | 37.81 | 27 | 1530 | 4248 | 11235 | MN727199 | De novo | RNA library |
| 25034 | 1/22/2015 | 416_R | L004-r704 | Picorna-like | Unknown | 4.17 | 8 | 43 | 469 | 11235 | MN727199 | De novo | RNA library |
| 25103 | 2/18/2015 | 429_R | L007-r704 | Picorna-like | Unknown | 5.43 | 5 | 43 | 610 | 11235 | MN727199 | De novo | RNA library |
| 25644 | 6/4/2015 | 564_R | L7-r717 | Picorna-like | Unknown | 32.47 | 8 | 395 | 3648 | 11235 | MN727199 | De novo | RNA library |
| 25671 | 6/8/2015 | 569_R | L001-r722 | Picorna-like | Unknown | 74.5 | 107 | 9051 | 8370 | 11235 | MN727199 | De novo | RNA library |
| 25694 | 6/11/2015 | 582_R | L004-r722 | Picorna-like | Unknown | 5.96 | 4 | 34 | 669 | 11235 | MN727199 | De novo | RNA library |
| 25700 | 6/11/2015 | 583_R | L004-r722 | Picorna-like | Unknown | 17.13 | 7 | 152 | 1924 | 11235 | MN727199 | De novo | RNA library |
| 25736 | 6/18/2015 | 866_R | L5-r745 | Picorna-like | Unknown | 7.84 | 5 | 44 | 881 | 11235 | MN727199 | De novo | RNA library |
| 25761 | 6/23/2015 | 601_R | L003-r723 | Picorna-like | Unknown | 32.11 | 4 | 207 | 3607 | 11235 | MN727199 | De novo | RNA library |
| 25789 | 6/25/2015 | 705_R | L8-r734 | Picorna-like | Unknown | 6.66 | 2 | 27 | 748 | 11235 | MN727199 | De novo | RNA library |
| 25807 | 6/29/2015 | 711_R | L1-r735 | Picorna-like | Unknown | 3.51 | 4 | 29 | 394 | 11235 | MN727199 | De novo | RNA library |
| 25837 | 7/2/2015 | 720_R | L4-r735 | Picorna-like | Unknown | 51.94 | 218 | 17363 | 5835 | 11235 | MN727199 | De novo | RNA library |
| 25838 | 7/2/2015 | 721_R | L5-r735 | Picorna-like | Unknown | 13.48 | 4 | 95 | 1514 | 11235 | MN727199 | De novo | RNA library |
| 25874 | 7/9/2015 | 776_R | L5-r737 | Picorna-like | Unknown | 21.26 | 3 | 99 | 2389 | 11235 | MN727199 | De novo | RNA library |
| 25993 | 7/30/2015 | 747_R | L5-r736 | Picorna-like | Unknown | 3.71 | 2 | 15 | 417 | 11235 | MN727199 | De novo | RNA library |
| 26130 | 8/24/2015 | 808_R | L5-r740 | Picorna-like | Unknown | 9.61 | 3 | 46 | 1080 | 11235 | MN727199 | De novo | RNA library |
| 26294 | 9/14/2015 | 878_R | L8-r745 | Picorna-like | Unknown | 4.85 | 7 | 39 | 545 | 11235 | MN727199 | De novo | RNA library |
| 26349 | 9/21/2015 | 3_R | L1-r669 | Picorna-like | Unknown | 4.76 | 1 | 10 | 535 | 11235 | MN727199 | De novo | RNA library |
| 26513 | 11/19/2015 | 17_R | L5-r669 | Picorna-like | Unknown | 4.44 | 1 | 6 | 499 | 11235 | MN727199 | De novo | RNA library |
| 21148 | 3/13/2015 | 126_D | L008-r49 | Porcine parvovirus 4 | Parvoviridae | 5.62 | 2 | 10 | 332 | 5906 | NC_014665 | Virosaurus | DNA library |
| 21195 | 12/11/2015 | 60_D | L008-r46 | Porcine parvovirus 4 | Parvoviridae | 5.18 | 1 | 4 | 306 | 5906 | NC_014665 | Virosaurus | DNA library |
| 21247 | 4/10/2015 | 215_D | L008-r67 | Porcine parvovirus 4 | Parvoviridae | 13.11 | 1 | 12 | 774 | 5906 | NC_014665 | Virosaurus | DNA library |
| 21253 | 4/13/2015 | 216_D | L005-r57 | Porcine parvovirus 4 | Parvoviridae | 7.09 | 4 | 27 | 419 | 5906 | NC_014665 | Virosaurus | DNA library |
| 21259 | 4/15/2015 | 218_D | L007-r55 | Porcine parvovirus 4 | Parvoviridae | 11.07 | 1 | 8 | 654 | 5906 | NC_014665 | Virosaurus | DNA library |
| 21282 | 4/21/2015 | 797_D | L8-r111 | Porcine parvovirus 4 | Parvoviridae | 6.33 | 1 | 5 | 374 | 5906 | NC_014665 | Virosaurus | DNA library |
| 21294 | 5/5/2015 | 224_D | L006-r55 | Porcine parvovirus 4 | Parvoviridae | 8.55 | 2 | 24 | 505 | 5906 | NC_014665 | Virosaurus | DNA library |
| 21303 | 5/6/2015 | 226_D | L001-r61 | Porcine parvovirus 4 | Parvoviridae | 16.04 | 2 | 29 | 947 | 5906 | NC_014665 | Virosaurus | DNA library |
| 21332 | 5/11/2015 | 228_D | L003-r61 | Porcine parvovirus 4 | Parvoviridae | 7.67 | 1 | 8 | 453 | 5906 | NC_014665 | Virosaurus | DNA library |
| 21360 | 5/13/2015 | 235_D | L003-r61 | Porcine parvovirus 4 | Parvoviridae | 6.77 | 1 | 4 | 400 | 5906 | NC_014665 | Virosaurus | DNA library |
| 25150 | 3/3/2015 | 441_D | L006-r75 | Porcine parvovirus 4 | Parvoviridae | 7.6 | 1 | 7 | 449 | 5906 | NC_014665 | Virosaurus | DNA library |
| 25160 | 3/5/2015 | 444_D | L001-r75 | Porcine parvovirus 4 | Parvoviridae | 20.67 | 2 | 27 | 1221 | 5906 | NC_014665 | Virosaurus | DNA library |
| 25162 | 3/5/2015 | 445_D | L002-r75 | Porcine parvovirus 4 | Parvoviridae | 8.62 | 2 | 13 | 509 | 5906 | NC_014665 | Virosaurus | DNA library |
| 25181 | 3/9/2015 | 451_D | L007-r75 | Porcine parvovirus 4 | Parvoviridae | 5.99 | 1 | 6 | 354 | 5906 | NC_014665 | Virosaurus | DNA library |
| 25195 | 3/12/2015 | 453_D | L002-r77 | Porcine parvovirus 4 | Parvoviridae | 7.33 | 1 | 7 | 433 | 5906 | NC_014665 | Virosaurus | DNA library |
| 25199 | 3/12/2015 | 455_D | L004-r77 | Porcine parvovirus 4 | Parvoviridae | 13.8 | 1 | 11 | 815 | 5906 | NC_014665 | Virosaurus | DNA library |
| 25217 | 3/19/2015 | 462_D | L004-r77 | Porcine parvovirus 4 | Parvoviridae | 87.83 | 5 | 361 | 5187 | 5906 | NC_014665 | Virosaurus | DNA library |
| 25220 | 3/19/2015 | 463_D | L005-r77 | Porcine parvovirus 4 | Parvoviridae | 13.95 | 1 | 12 | 824 | 5906 | NC_014665 | Virosaurus | DNA library |
| 25282 | 4/10/2015 | 474_D | L001-r77 | Porcine parvovirus 4 | Parvoviridae | 5.6 | 1 | 5 | 331 | 5906 | NC_014665 | Virosaurus | DNA library |
| 25286 | 4/10/2015 | 476_D | L004-r77 | Porcine parvovirus 4 | Parvoviridae | 16.04 | 1 | 14 | 947 | 5906 | NC_014665 | Virosaurus | DNA library |
| 25299 | 4/13/2015 | 478_D | L001-r80 | Porcine parvovirus 4 | Parvoviridae | 18.27 | 1 | 20 | 1079 | 5906 | NC_014665 | Virosaurus | DNA library |
| 25323 | 4/15/2015 | 482_D | L002-r77 | Porcine parvovirus 4 | Parvoviridae | 12.09 | 1 | 14 | 714 | 5906 | NC_014665 | Virosaurus | DNA library |
| 25325 | 4/15/2015 | 483_D | L004-r77 | Porcine parvovirus 4 | Parvoviridae | 5.86 | 1 | 4 | 346 | 5906 | NC_014665 | Virosaurus | DNA library |
| 25329 | 4/16/2015 | 484_D | L005-r77 | Porcine parvovirus 4 | Parvoviridae | 14.46 | 1 | 18 | 854 | 5906 | NC_014665 | Virosaurus | DNA library |
| 25353 | 4/21/2015 | 488_D | L001-r77 | Porcine parvovirus 4 | Parvoviridae | 19.49 | 1 | 19 | 1151 | 5906 | NC_014665 | Virosaurus | DNA library |
| 25374 | 4/23/2015 | 493_D | L002-r80 | Porcine parvovirus 4 | Parvoviridae | 9.58 | 1 | 7 | 566 | 5906 | NC_014665 | Virosaurus | DNA library |
| 25424 | 5/6/2015 | 501_D | L6-r82 | Porcine parvovirus 4 | Parvoviridae | 10.08 | 1 | 9 | 595 | 5906 | NC_014665 | Virosaurus | DNA library |
| 25425 | 5/6/2015 | 502_D | L005-r84 | Porcine parvovirus 4 | Parvoviridae | 10.8 | 1 | 12 | 638 | 5906 | NC_014665 | Virosaurus | DNA library |
| 25441 | 5/7/2015 | 589_D | L2-r89 | Porcine parvovirus 4 | Parvoviridae | 10.02 | 1 | 15 | 592 | 5906 | NC_014665 | Virosaurus | DNA library |
| 25463 | 5/12/2015 | 512_D | L005-r80 | Porcine parvovirus 4 | Parvoviridae | 5.99 | 1 | 5 | 354 | 5906 | NC_014665 | Virosaurus | DNA library |
| 25467 | 5/12/2015 | 513_D | L006-r80 | Porcine parvovirus 4 | Parvoviridae | 7.45 | 1 | 9 | 440 | 5906 | NC_014665 | Virosaurus | DNA library |
| 25472 | 5/11/2015 | 516_D | L005-r84 | Porcine parvovirus 4 | Parvoviridae | 5.74 | 1 | 6 | 339 | 5906 | NC_014665 | Virosaurus | DNA library |
| 25488 | 5/15/2015 | 519_D | L004-r80 | Porcine parvovirus 4 | Parvoviridae | 17.15 | 2 | 25 | 1013 | 5906 | NC_014665 | Virosaurus | DNA library |
| 21026 | 1/28/2015 | 97_D | L007-r49 | Porcine parvovirus 5 | Parvoviridae | 7.3 | 1 | 7 | 424 | 5806 | NC_023020 | Virosaurus | DNA library |
| 21042 | 2/3/2015 | 101_D | L002-r55 | Porcine parvovirus 5 | Parvoviridae | 6.89 | 1 | 7 | 400 | 5806 | NC_023020 | Virosaurus | DNA library |
| 21195 | 12/11/2015 | 60_D | L008-r46 | Porcine parvovirus 5 | Parvoviridae | 12.01 | 1 | 15 | 697 | 5806 | NC_023020 | Virosaurus | DNA library |
| 21291 | 4/27/2015 | 223_D | L005-r57 | Porcine parvovirus 5 | Parvoviridae | 6.29 | 1 | 6 | 365 | 5806 | NC_023020 | Virosaurus | DNA library |
| 21344 | 5/12/2015 | 231_D | L006-r61 | Porcine parvovirus 5 | Parvoviridae | 6.13 | 1 | 4 | 356 | 5806 | NC_023020 | Virosaurus | DNA library |
| 25160 | 3/5/2015 | 444_D | L001-r75 | Porcine parvovirus 5 | Parvoviridae | 6.15 | 1 | 7 | 357 | 5806 | NC_023020 | Virosaurus | DNA library |
| 25162 | 3/5/2015 | 445_D | L002-r75 | Porcine parvovirus 5 | Parvoviridae | 5.17 | 1 | 3 | 300 | 5806 | NC_023020 | Virosaurus | DNA library |
| 25212 | 3/18/2015 | 459_D | L001-r77 | Porcine parvovirus 5 | Parvoviridae | 5.77 | 1 | 6 | 335 | 5806 | NC_023020 | Virosaurus | DNA library |
| 25217 | 3/19/2015 | 462_D | L004-r77 | Porcine parvovirus 5 | Parvoviridae | 85.6 | 2 | 184 | 4970 | 5806 | NC_023020 | Virosaurus | DNA library |
| 25283 | 4/10/2015 | 475_D | L003-r77 | Porcine parvovirus 5 | Parvoviridae | 6.99 | 1 | 5 | 406 | 5806 | NC_023020 | Virosaurus | DNA library |
| 25329 | 4/16/2015 | 484_D | L005-r77 | Porcine parvovirus 5 | Parvoviridae | 17.76 | 2 | 32 | 1031 | 5806 | NC_023020 | Virosaurus | DNA library |
| 25353 | 4/21/2015 | 488_D | L001-r77 | Porcine parvovirus 5 | Parvoviridae | 8.7 | 1 | 7 | 505 | 5806 | NC_023020 | Virosaurus | DNA library |
| 25355 | 4/21/2015 | 489_D | L002-r77 | Porcine parvovirus 5 | Parvoviridae | 5.17 | 1 | 4 | 300 | 5806 | NC_023020 | Virosaurus | DNA library |
| 25441 | 5/7/2015 | 589_D | L2-r89 | Porcine parvovirus 5 | Parvoviridae | 5.17 | 1 | 4 | 300 | 5806 | NC_023020 | Virosaurus | DNA library |
| 21026 | 1/28/2015 | 97_D | L007-r49 | Porcine parvovirus 6 | Parvoviridae | 13.79 | 1 | 9 | 855 | 6200 | KY094494 | Virosaurus | DNA library |
| 25217 | 3/19/2015 | 462_D | L004-r77 | Porcine parvovirus 6 | Parvoviridae | 54.53 | 1 | 62 | 3381 | 6200 | KY094494 | Virosaurus | DNA library |
| 25376 | 4/24/2015 | 798_D | L5-r113 | Porcine parvovirus 6 | Parvoviridae | 4.84 | 1 | 3 | 300 | 6200 | KY094494 | Virosaurus | DNA library |
| 21158 | 3/17/2015 | 127_R | L002-r677 | Providence-like_virus_1 | Unknown | 100 | 43 | 2335 | 5325 | 5325 | MN727248 | De novo | RNA library |
| 25003 | 12/11/2014 | 407_R | L002-r704 | Providence-like_virus_1 | Unknown | 97.88 | 43 | 2593 | 5212 | 5325 | MN727248 | De novo | RNA library |
| 25097 | 2/17/2015 | 427_R | L007-r704 | Providence-like_virus_1 | Unknown | 19.25 | 11 | 143 | 1025 | 5325 | MN727248 | De novo | RNA library |
| 25761 | 6/23/2015 | 601_R | L003-r723 | Providence-like_virus_1 | Unknown | 35.51 | 3 | 80 | 1891 | 5325 | MN727248 | De novo | RNA library |
| 25799 | 6/29/2015 | 609_R | L005-r723 | Providence-like_virus_1 | Unknown | 13.95 | 5 | 43 | 743 | 5325 | MN727248 | De novo | RNA library |
| 25837 | 7/2/2015 | 720_R | L4-r735 | Providence-like_virus_1 | Unknown | 58.37 | 7 | 284 | 3108 | 5325 | MN727248 | De novo | RNA library |
| 26089 | 8/14/2015 | 760_R | L8-r736 | Providence-like_virus_1 | Unknown | 11.96 | 6 | 40 | 637 | 5325 | MN727248 | De novo | RNA library |
| 21006 | 1/16/2015 | 92_R | L001-r676 | Providence-like_virus_2 | Unknown | 99.63 | 543 | 28724 | 5575 | 5596 | MN727249 | De novo | RNA library |
| 21602 | 6/19/2015 | 285_R | L001-r693 | Providence-like_virus_2 | Unknown | 6.9 | 15 | 47 | 386 | 5596 | MN727249 | De novo | RNA library |
| 21627 | 6/24/2015 | 602_R | L004-r723 | Providence-like_virus_2 | Unknown | 24.39 | 6 | 103 | 1365 | 5596 | MN727249 | De novo | RNA library |
| 21703 | 7/9/2015 | 617_R | L007-r723 | Providence-like_virus_2 | Unknown | 33.27 | 8 | 187 | 1862 | 5596 | MN727249 | De novo | RNA library |
| 21712 | 7/9/2015 | 301_R | L005-r693 | Providence-like_virus_2 | Unknown | 97.71 | 17 | 1023 | 5468 | 5596 | MN727249 | De novo | RNA library |
| 21766 | 7/21/2015 | 621_R | L008-r723 | Providence-like_virus_2 | Unknown | 32.68 | 9 | 228 | 1829 | 5596 | MN727249 | De novo | RNA library |
| 21770 | 7/21/2015 | 625_R | L1-r724 | Providence-like_virus_2 | Unknown | 97.73 | 25 | 1381 | 5469 | 5596 | MN727249 | De novo | RNA library |
| 21789 | 7/23/2015 | 627_R | L2-r724 | Providence-like_virus_2 | Unknown | 98.98 | 18 | 1159 | 5539 | 5596 | MN727249 | De novo | RNA library |
| 22067 | 9/3/2015 | 346_R | L001-r702 | Providence-like_virus_2 | Unknown | 50.48 | 2 | 132 | 2825 | 5596 | MN727249 | De novo | RNA library |
| 22079 | 9/7/2015 | 349_R | L002-r702 | Providence-like_virus_2 | Unknown | 99.66 | 2338 | 128127 | 5577 | 5596 | MN727249 | De novo | RNA library |
| 22095 | 9/8/2015 | 860_R | L4-r745 | Providence-like_virus_2 | Unknown | 80.08 | 13 | 843 | 4481 | 5596 | MN727249 | De novo | RNA library |
| 22108 | 9/9/2015 | 353_R | L003-r702 | Providence-like_virus_2 | Unknown | 100 | 572 | 32112 | 5596 | 5596 | MN727249 | De novo | RNA library |
| 22419 | 10/29/2015 | 785_R | L7-r737 | Providence-like_virus_2 | Unknown | 20.96 | 3 | 50 | 1173 | 5596 | MN727249 | De novo | RNA library |
| 25026 | 1/21/2015 | 414_R | L004-r704 | Providence-like_virus_2 | Unknown | 96.44 | 81 | 4787 | 5397 | 5596 | MN727249 | De novo | RNA library |
| 25033 | 1/22/2015 | 415_R | L004-r704 | Providence-like_virus_2 | Unknown | 57.38 | 17 | 646 | 3211 | 5596 | MN727249 | De novo | RNA library |
| 25805 | 6/29/2015 | 610_R | L006-r723 | Providence-like_virus_2 | Unknown | 80.31 | 56 | 2917 | 4494 | 5596 | MN727249 | De novo | RNA library |
| 25807 | 6/29/2015 | 711_R | L1-r735 | Providence-like_virus_2 | Unknown | 36.26 | 10 | 250 | 2029 | 5596 | MN727249 | De novo | RNA library |
| 22485 | 11/26/2015 | 53_R | L006-r674 | Rhinovirus A | Picornaviridae | 7.72 | 1 | 6 | 551 | 7137 | FJ445154 | Virosaurus | RNA library |
| 22653 | 1/21/2016 | 73_R | L003-r675 | Rhinovirus A | Picornaviridae | 6.1 | 1 | 5 | 435 | 7131 | GQ415051 | Virosaurus | RNA library |
| 22665 | 1/27/2016 | 86_R | L007-r675 | Rhinovirus A | Picornaviridae | 96.73 | 18 | 1488 | 6898 | 7131 | GQ415051 | Virosaurus | RNA library |
| 21018 | 1/26/2015 | 94_R | L001-r676 | Rhinovirus C | Picornaviridae | 87.87 | 20 | 1428 | 6228 | 7088 | JN837686 | Virosaurus | RNA library |
| 21027 | 1/28/2015 | 98_R | L002-r676 | Rhinovirus C | Picornaviridae | 68.88 | 2 | 118 | 4901 | 7115 | EF582386 | Virosaurus | RNA library |
| 21060 | 2/13/2015 | 106_R | L004-r676 | Rhinovirus C | Picornaviridae | 99.42 | 47 | 3961 | 7068 | 7109 | EU840952 | Virosaurus | RNA library |
| 21060 | 2/13/2015 | 106_R | L004-r676 | Rhinovirus C | Picornaviridae | 99.19 | 25 | 2113 | 7057 | 7115 | EF582386 | Virosaurus | RNA library |
| 21104 | 2/27/2015 | 119_R | L008-r676 | Rhinovirus C | Picornaviridae | 65.36 | 2 | 99 | 4539 | 6945 | EF077279 | Virosaurus | RNA library |
| 21148 | 3/13/2015 | 126_R | L001-r677 | Rhinovirus C | Picornaviridae | 4.8 | 8 | 37 | 332 | 6913 | KX348031 | Virosaurus | RNA library |
| 21154 | 3/16/2015 | 770_R | L4-r737 | Rhinovirus C | Picornaviridae | 95.91 | 4 | 321 | 6855 | 7147 | MF775367 | Virosaurus | RNA library |
| 21180 | 3/13/2015 | 133_R | L003-r677 | Rhinovirus C | Picornaviridae | 4.95 | 14 | 81 | 342 | 6913 | KX348031 | Virosaurus | RNA library |
| 21333 | 5/11/2015 | 229_R | L002-r691 | Rhinovirus C | Picornaviridae | 4.22 | 1 | 3 | 300 | 7116 | KF958311 | Virosaurus | RNA library |
| 21377 | 5/18/2015 | 238_R | L005-r691 | Rhinovirus C | Picornaviridae | 5.09 | 2 | 10 | 361 | 7091 | MN727255 | De novo | RNA library |
| 21424 | 5/22/2015 | 245_R | L006-r691 | Rhinovirus C | Picornaviridae | 78.22 | 2 | 148 | 5581 | 7135 | EF186077 | Virosaurus | RNA library |
| 21508 | 6/1/2015 | 263_R | L003-r692 | Rhinovirus C | Picornaviridae | 82.59 | 2 | 165 | 5876 | 7115 | EF582386 | Virosaurus | RNA library |
| 21609 | 6/22/2015 | 287_R | L002-r693 | Rhinovirus C | Picornaviridae | 100 | 917 | 67173 | 7091 | 7091 | MN727255 | De novo | RNA library |
| 21610 | 6/22/2015 | 288_R | L002-r693 | Rhinovirus C | Picornaviridae | 99.92 | 759 | 55269 | 7085 | 7091 | MN727255 | De novo | RNA library |
| 21627 | 6/24/2015 | 602_R | L004-r723 | Rhinovirus C | Picornaviridae | 71.17 | 3 | 231 | 5062 | 7113 | KJ675506 | Virosaurus | RNA library |
| 21648 | 6/29/2015 | 291_R | L003-r693 | Rhinovirus C | Picornaviridae | 8.12 | 1 | 8 | 575 | 7081 | KY189320 | Virosaurus | RNA library |
| 21858 | 8/5/2015 | 321_R | L003-r698 | Rhinovirus C | Picornaviridae | 53.41 | 3 | 179 | 3787 | 7091 | MN727255 | De novo | RNA library |
| 21902 | 8/10/2015 | 326_R | L004-r698 | Rhinovirus C | Picornaviridae | 87.76 | 3 | 243 | 6260 | 7133 | JF317016 | Virosaurus | RNA library |
| 21911 | 8/13/2015 | 322_R | L003-r698 | Rhinovirus C | Picornaviridae | 99.1 | 31 | 2151 | 7027 | 7091 | MN727255 | De novo | RNA library |
| 21973 | 8/21/2015 | 334_R | L006-r698 | Rhinovirus C | Picornaviridae | 99.26 | 29 | 2483 | 7094 | 7147 | MF775367 | Virosaurus | RNA library |
| 22041 | 9/1/2015 | 342_R | L008-r698 | Rhinovirus C | Picornaviridae | 13.29 | 3 | 65 | 940 | 7073 | DQ875932 | Virosaurus | RNA library |
| 22326 | 10/22/2015 | 389_R | L005-r703 | Rhinovirus C | Picornaviridae | 99.44 | 616 | 51312 | 7075 | 7115 | EF582386 | Virosaurus | RNA library |
| 22326 | 10/22/2015 | 389_R | L005-r703 | Rhinovirus C | Picornaviridae | 7.88 | 5 | 132 | 560 | 7109 | EU840952 | Virosaurus | RNA library |
| 22437 | 11/6/2015 | 399_R | L007-r703 | Rhinovirus C | Picornaviridae | 99.31 | 17 | 1503 | 7098 | 7147 | MF775367 | Virosaurus | RNA library |
| 22453 | 11/16/2015 | 400_R | L007-r703 | Rhinovirus C | Picornaviridae | 82.24 | 4 | 285 | 5859 | 7124 | KJ675505 | Virosaurus | RNA library |
| 22457 | 11/16/2015 | 401_R | L008-r703 | Rhinovirus C | Picornaviridae | 99.21 | 8 | 704 | 7028 | 7084 | KY369877 | Virosaurus | RNA library |
| 22476 | 11/24/2015 | 46_R | L005-r674 | Rhinovirus C | Picornaviridae | 12.21 | 1 | 11 | 870 | 7124 | KJ675505 | Virosaurus | RNA library |
| 25160 | 3/5/2015 | 444_R | L003-r705 | Rhinovirus C | Picornaviridae | 76.12 | 16 | 1111 | 5417 | 7116 | KF958311 | Virosaurus | RNA library |
| 25163 | 3/5/2015 | 446_R | L004-r705 | Rhinovirus C | Picornaviridae | 98.82 | 15 | 1288 | 7031 | 7115 | EF582386 | Virosaurus | RNA library |
| 25179 | 3/9/2015 | 450_R | L005-r705 | Rhinovirus C | Picornaviridae | 6.54 | 1 | 11 | 465 | 7115 | EF582386 | Virosaurus | RNA library |
| 25598 | 5/28/2015 | 542_R | L2-r717 | Rhinovirus C | Picornaviridae | 77.49 | 16 | 1229 | 5514 | 7116 | KF958311 | Virosaurus | RNA library |
| 25604 | 5/29/2015 | 548_R | L3-r717 | Rhinovirus C | Picornaviridae | 18.71 | 6 | 118 | 1327 | 7091 | MN727255 | De novo | RNA library |
| 25653 | 6/4/2015 | 565_R | L8-r717 | Rhinovirus C | Picornaviridae | 99.87 | 111 | 7876 | 7082 | 7091 | MN727255 | De novo | RNA library |
| 25681 | 6/9/2015 | 864_R | L5-r745 | Rhinovirus C | Picornaviridae | 18.08 | 1 | 19 | 1250 | 6913 | KX348031 | Virosaurus | RNA library |
| 25691 | 6/10/2015 | 580_R | L003-r722 | Rhinovirus C | Picornaviridae | 13.46 | 1 | 19 | 960 | 7133 | JF317014 | Virosaurus | RNA library |
| 25779 | 6/25/2015 | 700_R | L7-r734 | Rhinovirus C | Picornaviridae | 15.6 | 9 | 120 | 1106 | 7091 | MN727255 | De novo | RNA library |
| 25788 | 6/25/2015 | 704_R | L8-r734 | Rhinovirus C | Picornaviridae | 12.58 | 1 | 13 | 899 | 7147 | MF775367 | Virosaurus | RNA library |
| 25807 | 6/29/2015 | 711_R | L1-r735 | Rhinovirus C | Picornaviridae | 56.65 | 1 | 79 | 4042 | 7135 | EF186077 | Virosaurus | RNA library |
| 26042 | 8/6/2015 | 637_R | L5-r724 | Rhinovirus C | Picornaviridae | 14.89 | 4 | 63 | 1056 | 7091 | MN727255 | De novo | RNA library |
| 21333 | 5/11/2015 | 229_R | L002-r691 | Rotavirus A | Reoviridae | 84.97 | Na | 1688 | 16344 | 19235 | AF190172:DQ146672:EF560707:HQ657145:JF831954:JN232047:KC769481:KJ094892:KU248438:KX655530:MG181560 | Virosaurus | RNA library |
| 21344 | 5/12/2015 | 231_R | L003-r691 | Rotavirus A | Reoviridae | 13.46 | Na | 32 | 2463 | 18299 | DQ146672:GQ477091:HQ657145:KC769481:KJ094892:KX655530:KX778615:KY634824:MG181661 | Virosaurus | RNA library |
| 21349 | 5/13/2015 | 232_R | L003-r691 | Rotavirus A | Reoviridae | 4.14 | Na | 10 | 766 | 18500 | JN129069:KC769481:KJ094892:MG181725 | Virosaurus | RNA library |
| 21350 | 5/13/2015 | 233_R | L003-r691 | Rotavirus A | Reoviridae | 15.42 | Na | 53 | 2961 | 19198 | AF190171:DQ146672:GQ477091:HQ657145:KC442953:KC769481:KU248438:KY634534:MG181725 | Virosaurus | RNA library |
| 21353 | 5/13/2015 | 234_R | L004-r691 | Rotavirus A | Reoviridae | 11.08 | Na | 28 | 2116 | 19091 | AF190172:DQ146672:KC769481:KM008637:MG181560 | Virosaurus | RNA library |
| 21368 | 5/14/2015 | 237_R | L004-r691 | Rotavirus A | Reoviridae | 5.71 | Na | 12 | 1062 | 18607 | DQ146672:GQ477091:JN232047:KU248438:MG181560 | Virosaurus | RNA library |
| 21384 | 5/18/2015 | 240_R | L005-r691 | Rotavirus A | Reoviridae | 1.72 | Na | 4 | 320 | 18623 | DQ146672 | Virosaurus | RNA library |
| 21453 | 5/26/2015 | 251_R | L008-r691 | Rotavirus A | Reoviridae | 14.03 | Na | 33 | 2582 | 18397 | DQ146672:GQ477091:HQ657145:JF831954:JN232047:KC769481:KJ094892:KX655530:KX778615 | Virosaurus | RNA library |
| 21468 | 5/27/2015 | 252_R | L008-r691 | Rotavirus A | Reoviridae | 83.93 | Na | 2038 | 15706 | 18713 | AY787647:EF554085:GU390434:KP752498:KU925784:KX655439:KX655449:KX655450:LC066658:MG181625:MG181773 | Virosaurus | RNA library |
| 21528 | 6/5/2015 | 271_R | L006-r692 | Rotavirus A | Reoviridae | 35.68 | Na | 132 | 6591 | 18472 | DQ146672:GQ477091:HQ657145:JN013983:KC769481:KJ094892:KU714449:KX632352:KX655530:KY634824:MG181725 | Virosaurus | RNA library |
| 21556 | 6/10/2015 | 276_R | L007-r692 | Rotavirus A | Reoviridae | 41.77 | Na | 147 | 7634 | 18275 | DQ146672:HQ657145:JF831954:JN232047:KC769481:KJ094892:KX655530:KY634824:MG181560 | Virosaurus | RNA library |
| 21606 | 6/22/2015 | 598_R | L003-r723 | Rotavirus A | Reoviridae | 87.05 | Na | 1498 | 16289 | 18713 | AY787647:EF554085:GU390434:KP752498:KU925784:KX655439:KX655449:KX655450:LC066658:MG181625:MG181773 | Virosaurus | RNA library |
| 21627 | 6/24/2015 | 602_R | L004-r723 | Rotavirus A | Reoviridae | 35.97 | Na | 124 | 6647 | 18479 | DQ146672:EF560707:HQ657145:JF831954:KC769481:KJ094892:KP752498:KU248438:KX655530:KY634824:MG181725 | Virosaurus | RNA library |
| 21644 | 6/29/2015 | 611_R | L006-r723 | Rotavirus A | Reoviridae | 6.74 | Na | 18 | 1235 | 18325 | KC769481:KU714449:KX655530:KY634824:MG181560 | Virosaurus | RNA library |
| 21650 | 6/30/2015 | 293_R | L003-r693 | Rotavirus A | Reoviridae | 72.28 | Na | 615 | 13383 | 18516 | DQ146672:EF560707:HQ657145:JN013983:JN232047:KC769481:KJ094892:KU248438:KX655530:KY634824:MG181560 | Virosaurus | RNA library |
| 21656 | 7/1/2015 | 294_R | L003-r693 | Rotavirus A | Reoviridae | 40.92 | Na | 143 | 7472 | 18258 | DQ146672:GQ477091:HQ657145:JF831954:KC769481:KJ094892:KX655530:KY634824:MG181661:MG181725 | Virosaurus | RNA library |
| 21664 | 7/1/2015 | 615_R | L007-r723 | Rotavirus A | Reoviridae | 35.05 | Na | 129 | 6472 | 18466 | DQ146672:EF560707:HQ657145:KC769481:KJ094892:KP752498:KX632352:KX655530:KY634824:MG181560 | Virosaurus | RNA library |
| 21701 | 7/9/2015 | 298_R | L004-r693 | Rotavirus A | Reoviridae | 19.67 | Na | 61 | 3625 | 18430 | DQ146672:EF560707:HQ657145:KC769481:KU714449:KX632352:KX655530:KY634534:KY634824:MG181560 | Virosaurus | RNA library |
| 21707 | 7/9/2015 | 299_R | L005-r693 | Rotavirus A | Reoviridae | 36.68 | Na | 149 | 6695 | 18252 | DQ146672:EF560707:HQ657145:JN013983:KC769481:KJ094892:KP752498:KX655530:KY634824:KY658044:MG181560 | Virosaurus | RNA library |
| 21712 | 7/9/2015 | 301_R | L005-r693 | Rotavirus A | Reoviridae | 28.87 | Na | 92 | 5261 | 18222 | GQ477091:HQ657145:JN013983:KC769481:KJ094892:KU714445:KX655530:KY634824:KY658044:MG181661:MG181725 | Virosaurus | RNA library |
| 21721 | 7/10/2015 | 305_R | L006-r693 | Rotavirus A | Reoviridae | 5.99 | Na | 15 | 1098 | 18344 | KC769481:KU714445:KY634824:MG181560 | Virosaurus | RNA library |
| 21762 | 7/21/2015 | 310_R | L007-r693 | Rotavirus A | Reoviridae | 17.43 | Na | 41 | 3213 | 18435 | GQ477091:HQ657145:JN013983:KC769481:KJ094892:KU714445:KX632352:KX655530:KY634824:MG181725 | Virosaurus | RNA library |
| 21766 | 7/21/2015 | 621_R | L008-r723 | Rotavirus A | Reoviridae | 11.46 | Na | 28 | 2107 | 18390 | DQ146672:HQ657145:JN013983:KC769481:KJ094892:KU714449:KX655530:KX778615:KY634824 | Virosaurus | RNA library |
| 21780 | 7/24/2015 | 626_R | L2-r724 | Rotavirus A | Reoviridae | 14.81 | Na | 41 | 2712 | 18316 | DQ146672:HQ657145:JF831954:KC769481:KU714449:KX655530:KY634824:MG181560 | Virosaurus | RNA library |
| 21801 | 7/24/2015 | 312_R | L008-r693 | Rotavirus A | Reoviridae | 85.28 | Na | 1926 | 15636 | 18334 | DQ146672:GQ477091:HQ657145:JF831954:JN232047:KC769481:KJ094892:KU248438:KX655530:KY634824:MG181560 | Virosaurus | RNA library |
| 21837 | 8/3/2015 | 315_R | L001-r698 | Rotavirus A | Reoviridae | 61.93 | Na | 307 | 11399 | 18406 | DQ146672:GQ477091:HQ657145:JN013983:JN232047:KC769481:KJ094892:KU248438:KX655530:KY634824:MG181560 | Virosaurus | RNA library |
| 21843 | 8/3/2015 | 318_R | L002-r698 | Rotavirus A | Reoviridae | 33.36 | Na | 98 | 6054 | 18150 | GQ477091:HQ657145:JF831954:KC769481:KJ094892:KU714445:KX655530:KY634824:KY658039:MG181661:MG181725 | Virosaurus | RNA library |
| 21849 | 8/4/2015 | 319_R | L002-r698 | Rotavirus A | Reoviridae | 38.34 | Na | 131 | 7094 | 18501 | AB326292:DQ146672:EF560707:HQ657145:KC769481:KJ094892:KU714449:KX632352:KX655530:KY634824:MG181725 | Virosaurus | RNA library |
| 21937 | 8/18/2015 | 330_R | L005-r698 | Rotavirus A | Reoviridae | 24.4 | Na | 66 | 4454 | 18252 | DQ146672:EF560707:HQ657145:JF831954:KC769481:KJ094892:KP752498:KX655530:KY634824:MG181560 | Virosaurus | RNA library |
| 21938 | 8/18/2015 | 331_R | L005-r698 | Rotavirus A | Reoviridae | 26.29 | Na | 75 | 4792 | 18225 | DQ146672:GQ477091:HQ657145:JF831954:KC769481:KU714449:KX655530:KY634824:MG181560 | Virosaurus | RNA library |
| 21973 | 8/21/2015 | 334_R | L006-r698 | Rotavirus A | Reoviridae | 3.95 | Na | 8 | 734 | 18603 | HQ657145:KC769481:KX655439:KX655530 | Virosaurus | RNA library |
| 22013 | 8/27/2015 | 340_R | L008-r698 | Rotavirus A | Reoviridae | 2.51 | Na | 6 | 464 | 18476 | GQ477091:KX655530 | Virosaurus | RNA library |
| 22107 | 9/9/2015 | 352_R | L003-r702 | Rotavirus A | Reoviridae | 4.54 | Na | 11 | 862 | 19000 | AF190172:GQ477091:JN129069:KC769481:MG181560 | Virosaurus | RNA library |
| 22108 | 9/9/2015 | 353_R | L003-r702 | Rotavirus A | Reoviridae | 10.67 | Na | 29 | 2041 | 19133 | AF190172:D86274:DQ146672:JF831954:KC769481:KJ094892:MG181725 | Virosaurus | RNA library |
| 22110 | 9/9/2015 | 354_R | L003-r702 | Rotavirus A | Reoviridae | 6.16 | Na | 16 | 1140 | 18505 | AB326292:DQ146672:HQ657145:KC769481:KJ094892:MG181625 | Virosaurus | RNA library |
| 22117 | 9/9/2015 | 655_R | L2-r725 | Rotavirus A | Reoviridae | 7.94 | Na | 25 | 1469 | 18511 | EF560707:KC769481:KX655530:MG181560:MG181661 | Virosaurus | RNA library |
| 22152 | 9/14/2015 | 660_R | L3-r725 | Rotavirus A | Reoviridae | 18.8 | Na | 62 | 3466 | 18440 | DQ146672:GQ477091:HQ657145:KC769481:KJ094892:KX632352:KX655530:KX778615:KY634824 | Virosaurus | RNA library |
| 22206 | 9/21/2015 | 366_R | L007-r702 | Rotavirus A | Reoviridae | 89.42 | Na | 3975 | 16734 | 18713 | AY787647:EF554085:GU390434:KP752498:KU925784:KX655439:KX655449:KX655450:LC066658:MG181625:MG181773 | Virosaurus | RNA library |
| 22259 | 9/29/2015 | 374_R | L001-r703 | Rotavirus A | Reoviridae | 17.65 | Na | 47 | 3263 | 18484 | AB008288:DQ146672:FJ169862:GU199492:HQ657145:KC443316:KJ094892:KX655530:LC066658 | Virosaurus | RNA library |
| 22262 | 9/29/2015 | 375_R | L001-r703 | Rotavirus A | Reoviridae | 11.21 | Na | 31 | 2047 | 18255 | DQ146672:GQ477091:GU199492:HQ657145:JF831954:KJ094892:KX655530:KY634824:MG181725 | Virosaurus | RNA library |
| 22283 | 10/19/2015 | 784_R | L7-r737 | Rotavirus A | Reoviridae | 21.87 | Na | 58 | 3976 | 18180 | DQ146672:GQ477091:HQ657145:JN013983:KC769481:KJ094892:KX655530:KY634824:KY658044:MG181560 | Virosaurus | RNA library |
| 22319 | 10/20/2015 | 387_R | L004-r703 | Rotavirus A | Reoviridae | 61.89 | Na | 359 | 11505 | 18590 | DQ146672:EF560707:HQ657145:JN013983:KC769481:KJ094892:KU248438:KX655530:KY634824:MG181661:MG181725 | Virosaurus | RNA library |
| 22435 | 11/6/2015 | 39_R | L002-r674 | Rotavirus A | Reoviridae | 54.43 | Na | 310 | 9973 | 18323 | DQ146672:GQ477091:HQ657145:JF831954:KC769481:KJ094892:KX632352:KX655530:KY634824:MG181560:MG181661 | Virosaurus | RNA library |
| 25560 | 5/25/2015 | 861_R | L4-r745 | Rotavirus A | Reoviridae | 5.54 | Na | 16 | 1038 | 18745 | GU390434:KC442953:KP752498:KU925784:KX655439:KX655450 | Virosaurus | RNA library |
| 25563 | 5/25/2015 | 530_R | L6-r714 | Rotavirus A | Reoviridae | 5.37 | Na | 14 | 998 | 18588 | EF554085:KU925784:KX655439:MG181625 | Virosaurus | RNA library |
| 25574 | 5/26/2015 | 533_R | L7-r714 | Rotavirus A | Reoviridae | 90.8 | Na | 11621 | 16980 | 18701 | AY787647:EF554085:GU390434:KU925784:KX655439:KX655445:KX655449:LC066658:LC169961:MG181625:MG181773 | Virosaurus | RNA library |
| 25594 | 5/28/2015 | 540_R | L1-r717 | Rotavirus A | Reoviridae | 86.87 | Na | 9758 | 16290 | 18752 | AY787647:EF554085:GU390434:KU925784:KX655439:KX655445:KX655449:KX655450:LC066658:MG181625:MG181773 | Virosaurus | RNA library |
| 25598 | 5/28/2015 | 542_R | L2-r717 | Rotavirus A | Reoviridae | 5.42 | Na | 14 | 1004 | 18540 | DQ146685:KU925784:KX655445:LC066658 | Virosaurus | RNA library |
| 25619 | 6/2/2015 | 556_R | L5-r717 | Rotavirus A | Reoviridae | 89.88 | Na | 4317 | 16820 | 18713 | AY787647:EF554085:GU390434:KP752498:KU925784:KX655439:KX655449:KX655450:LC066658:MG181625:MG181773 | Virosaurus | RNA library |
| 25621 | 6/2/2015 | 863_R | L4-r745 | Rotavirus A | Reoviridae | 10.69 | Na | 30 | 1983 | 18553 | AY787645:EF554085:KU925784:KX655439:KX655445:LC066658:MG181625 | Virosaurus | RNA library |
| 25626 | 6/2/2015 | 594_R | L002-r723 | Rotavirus A | Reoviridae | 4.87 | Na | 11 | 901 | 18520 | EF554085:KU925784:LC066658:MG181773 | Virosaurus | RNA library |
| 25643 | 6/4/2015 | 799_R | L3-r740 | Rotavirus A | Reoviridae | 9.64 | Na | 23 | 1782 | 18482 | EF554085:KC443316:KU925784:LC066658:MG181625 | Virosaurus | RNA library |
| 25652 | 6/4/2015 | 801_R | L3-r740 | Rotavirus A | Reoviridae | 2.5 | Na | 6 | 463 | 18555 | KP752498:KX655439 | Virosaurus | RNA library |
| 25675 | 6/9/2015 | 571_R | L001-r722 | Rotavirus A | Reoviridae | 13.14 | Na | 39 | 2434 | 18529 | AY787647:DQ146685:KU925784:KX655439:KX655445:LC066658:MG181625:MG181773 | Virosaurus | RNA library |
| 25690 | 6/10/2015 | 579_R | L003-r722 | Rotavirus A | Reoviridae | 5.92 | Na | 17 | 1095 | 18488 | EF554085:KC442953:KP752498:KU925784:KX655439 | Virosaurus | RNA library |
| 25718 | 6/15/2015 | 681_R | L2-r734 | Rotavirus A | Reoviridae | 73.84 | Na | 626 | 13374 | 18112 | DQ146672:GQ477091:HQ657145:JF831954:JN232047:KC769481:KJ094892:KX655530:KY634824:KY658044:MG181560 | Virosaurus | RNA library |
| 25736 | 6/18/2015 | 866_R | L5-r745 | Rotavirus A | Reoviridae | 60.09 | Na | 328 | 11180 | 18606 | AY787647:EF554085:GU390434:KC870025:KP752498:KU925784:KX655439:LC066658:LC169961:MG181625:MG181773 | Virosaurus | RNA library |
| 25758 | 6/23/2015 | 694_R | L5-r734 | Rotavirus A | Reoviridae | 59.28 | Na | 273 | 10926 | 18432 | AB326292:DQ146672:EF560707:HQ657145:KC769481:KJ094892:KU248438:KU714449:KX655530:KY634824:MG181560 | Virosaurus | RNA library |
| 25766 | 6/24/2015 | 696_R | L6-r734 | Rotavirus A | Reoviridae | 11.45 | Na | 27 | 2099 | 18328 | DQ146672:GQ477091:KC769481:KJ094892:KX655530:KY634824:MG181661:MG181725 | Virosaurus | RNA library |
| 25768 | 6/24/2015 | 605_R | L004-r723 | Rotavirus A | Reoviridae | 15.6 | Na | 48 | 3008 | 19279 | AF190171:DQ146672:JF766596:KC769481:KJ094892:KU714449:KX655530:MG181725 | Virosaurus | RNA library |
| 25773 | 6/24/2015 | 698_R | L6-r734 | Rotavirus A | Reoviridae | 4.68 | Na | 14 | 900 | 19225 | AF190172:DQ146672:EF560707:HQ657145:KJ094892:KU714449 | Virosaurus | RNA library |
| 25805 | 6/29/2015 | 610_R | L006-r723 | Rotavirus A | Reoviridae | 20.35 | Na | 56 | 3756 | 18461 | EF560707:HQ657145:JN013983:KC769481:KJ094892:KX632343:KX655530:MG181560:MG181661 | Virosaurus | RNA library |
| 25807 | 6/29/2015 | 711_R | L1-r735 | Rotavirus A | Reoviridae | 18.25 | Na | 58 | 3332 | 18254 | EF560707:JN013978:JN013983:KC769481:KJ094892:KP752498:KX632343:KX655530:KY634824:MG181560 | Virosaurus | RNA library |
| 25843 | 7/3/2015 | 725_R | L6-r735 | Rotavirus A | Reoviridae | 9.44 | Na | 25 | 1738 | 18414 | JF831954:KC769481:KJ094892:KX632343:KX655530:KX778615:MG181661 | Virosaurus | RNA library |
| 25893 | 7/10/2015 | 734_R | L2-r736 | Rotavirus A | Reoviridae | 15.94 | Na | 43 | 2927 | 18359 | AB326292:DQ146672:EF560707:HQ657145:KC769481:KJ094892:KU714449:KX655530:KY634824:MG181725 | Virosaurus | RNA library |
| 25930 | 7/21/2015 | 622_R | L1-r724 | Rotavirus A | Reoviridae | 64.92 | Na | 388 | 11969 | 18436 | DQ146672:EF560707:HQ657145:JF831954:JN232047:KC769481:KJ094892:KX632352:KX655530:KY634824:MG181560 | Virosaurus | RNA library |
| 25955 | 7/23/2015 | 742_R | L4-r736 | Rotavirus A | Reoviridae | 20.32 | Na | 68 | 3733 | 18370 | DQ146672:GQ477091:HQ657145:JN013983:KC442953:KC769481:KM008637:KX632352:KY634824:MG181725 | Virosaurus | RNA library |
| 25969 | 7/27/2015 | 743_R | L4-r736 | Rotavirus A | Reoviridae | 4.94 | Na | 10 | 921 | 18635 | DQ146672:JN232047:KC769481:KX655530 | Virosaurus | RNA library |
| 25987 | 7/30/2015 | 746_R | L5-r736 | Rotavirus A | Reoviridae | 4.86 | Na | 13 | 902 | 18562 | HQ657145:KC769481:KX632343:KX655530:MG181725 | Virosaurus | RNA library |
| 26042 | 8/6/2015 | 637_R | L5-r724 | Rotavirus A | Reoviridae | 40.52 | Na | 165 | 7455 | 18400 | DQ146672:GQ477091:HQ657145:JF831954:KC769481:KJ094892:KX632352:KX655530:KY634824:MG181661:MG181725 | Virosaurus | RNA library |
| 26046 | 8/7/2015 | 753_R | L7-r736 | Rotavirus A | Reoviridae | 20.08 | Na | 55 | 3716 | 18509 | DQ146672:EF560707:HQ657145:JF831954:KC769481:KJ094892:KX632352:KX655530:KY634824:MG181725 | Virosaurus | RNA library |
| 26052 | 8/10/2015 | 755_R | L7-r736 | Rotavirus A | Reoviridae | 14.19 | Na | 45 | 2712 | 19118 | AF190172:DQ146672:GQ477091:HQ657145:KC769481:KP752498:KX632352:KX655530:KX778615:KY634534 | Virosaurus | RNA library |
| 26079 | 8/13/2015 | 674_R | L6-r725 | Rotavirus A | Reoviridae | 65.4 | Na | 391 | 12106 | 18510 | DQ146672:EF560707:HQ657145:JF831954:KC769481:KJ094892:KX632352:KX655530:KY634824:MG181661:MG181725 | Virosaurus | RNA library |
| 26101 | 9/9/2015 | 763_R | L1-r737 | Rotavirus A | Reoviridae | 6.58 | Na | 17 | 1206 | 18340 | GQ477091:HQ657145:KC769481:KU714445:KX655530:KY634824:MG181661 | Virosaurus | RNA library |
| 26108 | 8/18/2015 | 804_R | L4-r740 | Rotavirus A | Reoviridae | 40.53 | Na | 211 | 7777 | 19186 | AF190172:DQ146672:GQ477091:HQ657145:JN013983:KC769481:KJ094892:KX632352:KX655530:MG181560:MG181661 | Virosaurus | RNA library |
| 26129 | 8/21/2015 | 807_R | L5-r740 | Rotavirus A | Reoviridae | 6.43 | Na | 14 | 1306 | 20305 | AF190169:AF190172:JN232047:JN651766:JQ309138:KT694975 | Virosaurus | RNA library |
| 26163 | 8/27/2015 | 817_R | L8-r740 | Rotavirus A | Reoviridae | 8.49 | Na | 24 | 1620 | 19092 | AF190172:DQ146672:EF560707:JN974818:KC769481:KT694975:KX655530:KY634534:KY658034 | Virosaurus | RNA library |
| 26165 | 8/26/2015 | 818_R | L8-r740 | Rotavirus A | Reoviridae | 27.62 | Na | 74 | 5012 | 18148 | GQ477091:HQ657145:JF831954:JN232047:KC769481:KJ094892:KU714445:KX655530:KY634824:MG181560 | Virosaurus | RNA library |
| 26178 | 8/28/2015 | 822_R | L1-r741 | Rotavirus A | Reoviridae | 57.34 | Na | 256 | 10488 | 18292 | DQ146672:GQ477091:HQ657145:JF831954:KC769481:KJ094892:KP752498:KU248438:KX655530:KY634824:MG181560 | Virosaurus | RNA library |
| 26182 | 8/28/2015 | 825_R | L2-r741 | Rotavirus A | Reoviridae | 5.66 | Na | 15 | 1054 | 18629 | DQ146672:JN013983:KC769481:KX655530:MG181725 | Virosaurus | RNA library |
| 26194 | 8/31/2015 | 828_R | L3-r741 | Rotavirus A | Reoviridae | 90.81 | Na | 6424 | 17029 | 18752 | AY787647:EF554085:GU390434:KU925784:KX655439:KX655445:KX655449:KX655450:LC066658:MG181625:MG181773 | Virosaurus | RNA library |
| 26198 | 9/1/2015 | 829_R | L3-r741 | Rotavirus A | Reoviridae | 26.6 | Na | 82 | 4865 | 18291 | DQ146672:EF560707:HQ657145:JF831954:KC769481:KJ094892:KU714449:KX655530:KY634824:MG181560 | Virosaurus | RNA library |
| 26203 | 9/1/2015 | 831_R | L4-r741 | Rotavirus A | Reoviridae | 15.92 | Na | 40 | 2947 | 18514 | D86274:DQ146672:GU390434:HQ657145:KC769481:KJ094892:KX655530:KY634824:MG181725 | Virosaurus | RNA library |
| 26234 | 9/7/2015 | 841_R | L6-r741 | Rotavirus A | Reoviridae | 70.14 | Na | 457 | 12701 | 18109 | DQ146672:GQ477091:HQ657145:JF831954:KC769481:KJ094892:KU714449:KX655530:KY634824:KY658039:MG181560 | Virosaurus | RNA library |
| 26263 | 9/9/2015 | 854_R | L1-r745 | Rotavirus A | Reoviridae | 86.24 | Na | 2730 | 15966 | 18513 | DQ146672:EF560707:HQ657145:JF831954:JN232047:KC769481:KJ094892:KX632352:KX655530:KY634824:MG181725 | Virosaurus | RNA library |
| 26284 | 9/11/2015 | 874_R | L7-r745 | Rotavirus A | Reoviridae | 39.42 | Na | 162 | 7251 | 18395 | DQ146672:GQ477091:HQ657145:JN013983:KC769481:KJ094892:KU714449:KX632352:KX655530:KY634824:MG181560 | Virosaurus | RNA library |
| 26287 | 9/11/2015 | 876_R | L8-r745 | Rotavirus A | Reoviridae | 6.5 | Na | 15 | 1206 | 18562 | HQ657145:KC769481:KX632343:KX655530:MG181725 | Virosaurus | RNA library |
| 26294 | 9/14/2015 | 878_R | L8-r745 | Rotavirus A | Reoviridae | 4.06 | Na | 10 | 750 | 18474 | DQ146672:JF831954:KJ094892:KX778615 | Virosaurus | RNA library |
| 26295 | 9/14/2015 | 879_R | L8-r745 | Rotavirus A | Reoviridae | 12.84 | Na | 34 | 2385 | 18574 | D86274:DQ146672:KC769481:KX632352:KX655530:KX778615:KY634824 | Virosaurus | RNA library |
| 26299 | 9/14/2015 | 880_R | L1-r746 | Rotavirus A | Reoviridae | 7.16 | Na | 18 | 1377 | 19233 | AF190171:DQ146672:EF560707:HQ657145:KC442953:KC769481:KJ094892:MG181725 | Virosaurus | RNA library |
| 26301 | 9/15/2015 | 881_R | L1-r746 | Rotavirus A | Reoviridae | 5.98 | Na | 16 | 1101 | 18422 | EF560707:KC769481:KX655530:KY634824 | Virosaurus | RNA library |
| 26317 | 9/16/2015 | 678_R | L1-r734 | Rotavirus A | Reoviridae | 13.39 | Na | 42 | 2576 | 19242 | AB534534:AF190172:EF560707:HQ657145:JF831954:JN129069:KC769481:KU248438:KX655530:KX778615 | Virosaurus | RNA library |
| 26342 | 9/17/2015 | 893_R | L4-r746 | Rotavirus A | Reoviridae | 20.76 | Na | 63 | 3849 | 18543 | AB008288:DQ146672:EF560707:HQ657145:KC769481:KJ094892:KP752498:KX632352:KX655530:KY634824 | Virosaurus | RNA library |
| 26345 | 9/17/2015 | 679_R | L1-r734 | Rotavirus A | Reoviridae | 80.31 | Na | 975 | 14987 | 18662 | AY787647:EF554085:GU390434:KP752498:KU925784:KX655439:KX655449:LC066658:LC169961:MG181625:MG181773 | Virosaurus | RNA library |
| 26360 | 9/22/2015 | 899_R | L5-r746 | Rotavirus A | Reoviridae | 21.68 | Na | 68 | 3943 | 18188 | DQ146672:GQ477091:HQ657145:JF831954:KC769481:KJ094892:KP752498:KX655530:KX778615:KY634824 | Virosaurus | RNA library |
| 26361 | 9/22/2015 | 900_R | L6-r746 | Rotavirus A | Reoviridae | 14.41 | Na | 37 | 2626 | 18225 | DQ146672:GQ477091:KC769481:KJ094892:KU714449:KX655530:KX778615:KY634824:KY658039 | Virosaurus | RNA library |
| 26488 | 11/11/2015 | 15_R | L4-r669 | Rotavirus A | Reoviridae | 18.23 | Na | 49 | 3336 | 18298 | DQ146672:EF560707:HQ657145:JF831954:KC769481:KJ094892:KP752498:KX655530:KX778615:KY634824 | Virosaurus | RNA library |
| 26533 | 11/24/2015 | 663_R | L4-r725 | Rotavirus A | Reoviridae | 1.76 | Na | 4 | 327 | 18569 | KU925784 | Virosaurus | RNA library |
| 21701 | 7/9/2015 | 298_R | L004-r693 | Rotavirus D | Reoviridae | 6.09 | Na | 18 | 1127 | 18501 | JN034679:NC_014512:NC_014515:NC_014517:NC_014518 | Virosaurus | RNA library |
| 25423 | 5/6/2015 | 500_R | L7-r713 | Rotavirus D | Reoviridae | 1.86 | Na | 5 | 345 | 18501 | NC_014515 | Virosaurus | RNA library |
| 26278 | 9/10/2015 | 657_R | L2-r725 | Rubella virus | Togaviridae | 16.59 | 1 | 27 | 1622 | 9779 | AY258322 | Virosaurus | RNA library |
| 21437 | 5/22/2015 | 774_R | L5-r737 | Sapporo virus | Caliciviridae | 5.05 | 1 | 4 | 376 | 7442 | KT327081 | Virosaurus | RNA library |
| 25107 | 2/18/2015 | 430_R | L008-r704 | Sapporo virus | Caliciviridae | 8.05 | 1 | 6 | 600 | 7451 | KX274477 | Virosaurus | RNA library |
| 25491 | 5/15/2015 | 520_R | L4-r714 | Sapporo virus | Caliciviridae | 9.18 | 1 | 9 | 683 | 7438 | DQ125333 | Virosaurus | RNA library |
| 25885 | 7/13/2015 | 732_R | L1-r736 | Sapporo virus | Caliciviridae | 51.72 | 2 | 115 | 3849 | 7442 | KT327081 | Virosaurus | RNA library |
| 26287 | 9/11/2015 | 876_D | L5-r132 | Taupapillomavirus 3 | Papillomaviridae | 4.27 | 1 | 6 | 324 | 7584 | NC_021472 | Virosaurus | DNA library |
| 21027 | 1/28/2015 | 98_D | L008-r49 | Trichodysplasia spinulosa-associated polyomavirus | Polyomaviridae | 37.7 | 1 | 28 | 1974 | 5236 | KM007161 | Virosaurus | DNA library |
| 21138 | 3/11/2015 | 769_D | L4-r113 | Trichodysplasia spinulosa-associated polyomavirus | Polyomaviridae | 25.12 | 1 | 26 | 1315 | 5236 | KM007161 | Virosaurus | DNA library |
| 21004 | 12/12/2014 | 91_D | L001-r49 | TTMDV | Anelloviridae | 19.09 | 4 | 47 | 611 | 3201 | AB303563 | Virosaurus | DNA library |
| 21006 | 1/16/2015 | 92_D | L002-r49 | TTMDV | Anelloviridae | 12.19 | 4 | 22 | 390 | 3199 | AB303553 | Virosaurus | DNA library |
| 21007 | 1/19/2015 | 794_D | L8-r113 | TTMDV | Anelloviridae | 99.96 | 58 | 1485 | 2604 | 2605 | MN778014 | De novo | DNA library |
| 21014 | 1/23/2015 | 93_D | L003-r49 | TTMDV | Anelloviridae | 17.17 | 17 | 133 | 549 | 3198 | AB303554 | Virosaurus | DNA library |
| 21017 | 1/23/2015 | 766_D | L6-r111 | TTMDV | Anelloviridae | 28.68 | 5 | 54 | 728 | 2538 | KP343822 | Virosaurus | DNA library |
| 21018 | 1/26/2015 | 94_D | L004-r49 | TTMDV | Anelloviridae | 96.28 | 43 | 1246 | 2587 | 2687 | KP343821 | Virosaurus | DNA library |
| 21019 | 1/26/2015 | 95_D | L005-r49 | TTMDV | Anelloviridae | 18.51 | 30 | 337 | 592 | 3198 | AB303554 | Virosaurus | DNA library |
| 21021 | 1/26/2015 | 96_R | L002-r676 | TTMDV | Anelloviridae | 14.89 | 6 | 25 | 394 | 2646 | MN775645 | De novo | RNA library |
| 21021 | 1/26/2015 | 96_D | L006-r49 | TTMDV | Anelloviridae | 85.63 | 14 | 740 | 2730 | 3188 | KF545587 | Virosaurus | DNA library |
| 21026 | 1/28/2015 | 97_D | L007-r49 | TTMDV | Anelloviridae | 96.22 | 33 | 1078 | 2778 | 2887 | MN776313 | De novo | DNA library |
| 21027 | 1/28/2015 | 98_D | L008-r49 | TTMDV | Anelloviridae | 18.54 | 15 | 157 | 593 | 3198 | AB303554 | Virosaurus | DNA library |
| 21028 | 1/28/2015 | 796_D | L7-r111 | TTMDV | Anelloviridae | 100 | 15 | 508 | 2687 | 2687 | KP343821 | Virosaurus | DNA library |
| 21031 | 1/29/2015 | 586_D | L7-r88 | TTMDV | Anelloviridae | 18.95 | 9 | 73 | 481 | 2538 | KP343822 | Virosaurus | DNA library |
| 21039 | 2/2/2015 | 99_D | L002-r49 | TTMDV | Anelloviridae | 19.48 | 7 | 1074 | 623 | 3199 | AB303553 | Virosaurus | DNA library |
| 21041 | 2/2/2015 | 100_D | L003-r55 | TTMDV | Anelloviridae | 28.62 | 1 | 237 | 769 | 2687 | KP343821 | Virosaurus | DNA library |
| 21042 | 2/3/2015 | 101_D | L002-r55 | TTMDV | Anelloviridae | 85.9 | 6 | 233 | 2766 | 3220 | MN774941 | De novo | DNA library |
| 21044 | 2/3/2015 | 102_D | L005-r49 | TTMDV | Anelloviridae | 29.76 | 40 | 2128 | 952 | 3199 | AB303553 | Virosaurus | DNA library |
| 21048 | 2/4/2015 | 103_D | L001-r55 | TTMDV | Anelloviridae | 13.92 | 3 | 16 | 445 | 3198 | AB303554 | Virosaurus | DNA library |
| 21053 | 2/6/2015 | 104_D | L007-r49 | TTMDV | Anelloviridae | 23.8 | 3 | 48 | 761 | 3198 | AB303554 | Virosaurus | DNA library |
| 21060 | 2/13/2015 | 106_D | L001-r49 | TTMDV | Anelloviridae | 23.09 | 2 | 23 | 739 | 3201 | AB303556 | Virosaurus | DNA library |
| 21074 | 2/19/2015 | 107_D | L003-r49 | TTMDV | Anelloviridae | 39.8 | 11 | 171 | 1264 | 3176 | AB303560 | Virosaurus | DNA library |
| 21077 | 2/19/2015 | 108_D | L004-r49 | TTMDV | Anelloviridae | 16.6 | 21 | 177 | 531 | 3198 | AB303554 | Virosaurus | DNA library |
| 21080 | 2/20/2015 | 109_D | L005-r49 | TTMDV | Anelloviridae | 88.24 | 24 | 808 | 2813 | 3188 | KF545587 | Virosaurus | DNA library |
| 21082 | 2/20/2015 | 110_D | L006-r49 | TTMDV | Anelloviridae | 99.47 | 15 | 438 | 2619 | 2633 | MN776965 | De novo | DNA library |
| 21083 | 2/20/2015 | 111_D | L007-r49 | TTMDV | Anelloviridae | 16.7 | 34 | 351 | 534 | 3198 | AB303554 | Virosaurus | DNA library |
| 21085 | 2/23/2015 | 112_D | L008-r49 | TTMDV | Anelloviridae | 50.25 | 3 | 91 | 1620 | 3224 | AB303566 | Virosaurus | DNA library |
| 21090 | 2/24/2015 | 113_D | L001-r49 | TTMDV | Anelloviridae | 19.01 | 7 | 72 | 613 | 3224 | AB303558 | Virosaurus | DNA library |
| 21092 | 2/27/2015 | 114_D | L002-r49 | TTMDV | Anelloviridae | 13.19 | 4 | 30 | 422 | 3199 | AB303553 | Virosaurus | DNA library |
| 21099 | 2/26/2015 | 116_D | L005-r49 | TTMDV | Anelloviridae | 46.12 | 8 | 447 | 1475 | 3198 | AB303554 | Virosaurus | DNA library |
| 21102 | 2/27/2015 | 118_D | L007-r49 | TTMDV | Anelloviridae | 23.57 | 3 | 208 | 760 | 3224 | AB303558 | Virosaurus | DNA library |
| 21104 | 2/27/2015 | 119_D | L008-r49 | TTMDV | Anelloviridae | 59.96 | 1 | 35 | 1680 | 2802 | KF545588 | Virosaurus | DNA library |
| 21105 | 3/2/2015 | 120_D | L001-r49 | TTMDV | Anelloviridae | 46.93 | 4 | 78 | 1513 | 3224 | AB303566 | Virosaurus | DNA library |
| 21109 | 3/11/2015 | 767_D | L7-r111 | TTMDV | Anelloviridae | 95.08 | 5 | 143 | 2839 | 2986 | MN778956 | De novo | DNA library |
| 21110 | 3/3/2015 | 121_D | L002-r49 | TTMDV | Anelloviridae | 20.21 | 2 | 21 | 656 | 3246 | NC_009225 | Virosaurus | DNA library |
| 21115 | 3/4/2015 | 122_D | L003-r49 | TTMDV | Anelloviridae | 68.99 | 3 | 77 | 1927 | 2793 | KT163882 | Virosaurus | DNA library |
| 21116 | 3/4/2015 | 123_D | L005-r49 | TTMDV | Anelloviridae | 29.95 | 4 | 857 | 958 | 3199 | AB303553 | Virosaurus | DNA library |
| 21122 | 3/5/2015 | 124_D | L006-r49 | TTMDV | Anelloviridae | 99.89 | 5 | 272 | 2684 | 2687 | KP343821 | Virosaurus | DNA library |
| 21124 | 3/16/2015 | 768_D | L8-r111 | TTMDV | Anelloviridae | 20.94 | 3 | 65 | 665 | 3176 | AB303560 | Virosaurus | DNA library |
| 21129 | 3/10/2015 | 125_D | L007-r49 | TTMDV | Anelloviridae | 73.23 | 11 | 429 | 2342 | 3198 | AB303554 | Virosaurus | DNA library |
| 21138 | 3/11/2015 | 769_D | L4-r113 | TTMDV | Anelloviridae | 15.21 | 1 | 8 | 483 | 3176 | AB303560 | Virosaurus | DNA library |
| 21148 | 3/13/2015 | 126_D | L008-r49 | TTMDV | Anelloviridae | 16.92 | 5 | 39 | 543 | 3209 | AB303552 | Virosaurus | DNA library |
| 21154 | 3/16/2015 | 770_R | L4-r737 | TTMDV | Anelloviridae | 11.83 | 1 | 4 | 354 | 2993 | MN774964 | De novo | RNA library |
| 21154 | 3/16/2015 | 770_D | L5-r113 | TTMDV | Anelloviridae | 85.51 | 4 | 136 | 2396 | 2802 | KF545588 | Virosaurus | DNA library |
| 21158 | 3/17/2015 | 127_D | L001-r49 | TTMDV | Anelloviridae | 40.52 | 10 | 151 | 1287 | 3176 | AB303560 | Virosaurus | DNA library |
| 21159 | 3/17/2015 | 128_D | L002-r49 | TTMDV | Anelloviridae | 18.33 | 20 | 183 | 591 | 3224 | AB303558 | Virosaurus | DNA library |
| 21169 | 3/19/2015 | 129_D | L003-r49 | TTMDV | Anelloviridae | 26.57 | 2 | 23 | 844 | 3176 | AB303560 | Virosaurus | DNA library |
| 21175 | 3/16/2015 | 130_D | L004-r49 | TTMDV | Anelloviridae | 73.71 | 4 | 167 | 2350 | 3188 | KF545587 | Virosaurus | DNA library |
| 21176 | 3/16/2015 | 131_D | L006-r49 | TTMDV | Anelloviridae | 18.17 | 23 | 255 | 581 | 3198 | AB303554 | Virosaurus | DNA library |
| 21177 | 3/17/2015 | 132_D | L007-r49 | TTMDV | Anelloviridae | 17.32 | 4 | 33 | 554 | 3198 | AB303554 | Virosaurus | DNA library |
| 21180 | 3/13/2015 | 133_D | L008-r49 | TTMDV | Anelloviridae | 24.45 | 3 | 134 | 782 | 3199 | AB303553 | Virosaurus | DNA library |
| 21183 | 3/13/2015 | 134_D | L001-r49 | TTMDV | Anelloviridae | 99.04 | 160 | 4233 | 2586 | 2611 | MN775504 | De novo | DNA library |
| 21191 | 3/20/2015 | 50_D | L005-r46 | TTMDV | Anelloviridae | 51.51 | 69 | 7653 | 1676 | 3254 | NC_014093 | Virosaurus | DNA library |
| 21195 | 12/11/2015 | 60_D | L008-r46 | TTMDV | Anelloviridae | 21.39 | 34 | 655 | 684 | 3198 | AB303554 | Virosaurus | DNA library |
| 21200 | 3/20/2015 | 61_D | L001-r46 | TTMDV | Anelloviridae | 92.09 | 12 | 1363 | 2072 | 2250 | NC_007013 | Virosaurus | DNA library |
| 21201 | 3/23/2015 | 67_D | L008-r46 | TTMDV | Anelloviridae | 94.4 | 27 | 922 | 2124 | 2250 | NC_007013 | Virosaurus | DNA library |
| 21220 | 3/30/2015 | 213_D | L002-r57 | TTMDV | Anelloviridae | 100 | 38 | 1273 | 2687 | 2687 | KP343821 | Virosaurus | DNA library |
| 21234 | 4/8/2015 | 214_D | L003-r57 | TTMDV | Anelloviridae | 22.91 | 4 | 770 | 733 | 3199 | AB303553 | Virosaurus | DNA library |
| 21247 | 4/10/2015 | 215_D | L004-r57 | TTMDV | Anelloviridae | 17.45 | 9 | 60 | 558 | 3198 | AB303554 | Virosaurus | DNA library |
| 21253 | 4/13/2015 | 216_D | L005-r57 | TTMDV | Anelloviridae | 22.67 | 2 | 78 | 731 | 3224 | AB303566 | Virosaurus | DNA library |
| 21258 | 4/15/2015 | 217_D | L006-r55 | TTMDV | Anelloviridae | 15.85 | 19 | 229 | 507 | 3199 | AB303553 | Virosaurus | DNA library |
| 21259 | 4/15/2015 | 218_D | L007-r55 | TTMDV | Anelloviridae | 98.51 | 143 | 4507 | 2647 | 2687 | KP343821 | Virosaurus | DNA library |
| 21272 | 4/20/2015 | 220_D | L001-r57 | TTMDV | Anelloviridae | 17.1 | 10 | 81 | 547 | 3198 | AB303554 | Virosaurus | DNA library |
| 21277 | 4/20/2015 | 221_D | L003-r57 | TTMDV | Anelloviridae | 22.95 | 2 | 20 | 740 | 3224 | AB303566 | Virosaurus | DNA library |
| 21280 | 4/21/2015 | 222_D | L004-r57 | TTMDV | Anelloviridae | 41.63 | 24 | 410 | 1322 | 3176 | AB303560 | Virosaurus | DNA library |
| 21282 | 4/21/2015 | 797_D | L8-r111 | TTMDV | Anelloviridae | 18.11 | 13 | 174 | 579 | 3198 | AB303554 | Virosaurus | DNA library |
| 21294 | 5/5/2015 | 224_D | L006-r55 | TTMDV | Anelloviridae | 18.73 | 12 | 150 | 604 | 3224 | AB303558 | Virosaurus | DNA library |
| 21297 | 5/5/2015 | 225_D | L007-r55 | TTMDV | Anelloviridae | 89.57 | 125 | 3818 | 2705 | 3020 | MN780374 | De novo | DNA library |
| 21303 | 5/6/2015 | 226_D | L001-r61 | TTMDV | Anelloviridae | 16.39 | 4 | 37 | 524 | 3198 | AB303554 | Virosaurus | DNA library |
| 21323 | 5/7/2015 | 227_D | L002-r61 | TTMDV | Anelloviridae | 13.3 | 2 | 8 | 424 | 3188 | AB303562 | Virosaurus | DNA library |
| 21333 | 5/11/2015 | 229_D | L004-r61 | TTMDV | Anelloviridae | 25.03 | 5 | 69 | 795 | 3176 | AB303560 | Virosaurus | DNA library |
| 21344 | 5/12/2015 | 231_D | L006-r61 | TTMDV | Anelloviridae | 94.93 | 21 | 692 | 2136 | 2250 | NC_007013 | Virosaurus | DNA library |
| 21346 | 5/13/2015 | 856_D | L8-r124 | TTMDV | Anelloviridae | 39.4 | 3 | 308 | 1260 | 3198 | AB303554 | Virosaurus | DNA library |
| 21349 | 5/13/2015 | 232_D | L007-r61 | TTMDV | Anelloviridae | 31.14 | 10 | 468 | 996 | 3198 | AB303554 | Virosaurus | DNA library |
| 21350 | 5/13/2015 | 233_D | L008-r61 | TTMDV | Anelloviridae | 24.15 | 4 | 74 | 613 | 2538 | KP343822 | Virosaurus | DNA library |
| 21353 | 5/13/2015 | 234_D | L002-r61 | TTMDV | Anelloviridae | 52.42 | 4 | 371 | 1690 | 3224 | AB303566 | Virosaurus | DNA library |
| 21360 | 5/13/2015 | 235_D | L003-r61 | TTMDV | Anelloviridae | 46.47 | 6 | 3633 | 1486 | 3198 | AB303554 | Virosaurus | DNA library |
| 21362 | 5/14/2015 | 236_D | L004-r61 | TTMDV | Anelloviridae | 35.8 | 41 | 474 | 1113 | 3109 | MN780399 | De novo | DNA library |
| 21368 | 5/14/2015 | 237_D | L005-r61 | TTMDV | Anelloviridae | 33 | 1 | 34 | 1052 | 3188 | AB303559 | Virosaurus | DNA library |
| 21370 | 5/20/2015 | 590_R | L001-r723 | TTMDV | Anelloviridae | 14.81 | 3 | 13 | 411 | 2775 | MN775355 | De novo | RNA library |
| 21370 | 5/20/2015 | 590_D | L3-r89 | TTMDV | Anelloviridae | 96.09 | 17 | 1466 | 2582 | 2687 | KP343821 | Virosaurus | DNA library |
| 21377 | 5/18/2015 | 238_D | L006-r61 | TTMDV | Anelloviridae | 34.96 | 3 | 94 | 1118 | 3198 | AB303554 | Virosaurus | DNA library |
| 21382 | 5/18/2015 | 239_D | L007-r61 | TTMDV | Anelloviridae | 61.01 | 7 | 223 | 1951 | 3198 | AB303554 | Virosaurus | DNA library |
| 21384 | 5/18/2015 | 240_R | L005-r691 | TTMDV | Anelloviridae | 19.02 | 2 | 14 | 553 | 2907 | MN775975 | De novo | RNA library |
| 21384 | 5/18/2015 | 240_D | L008-r61 | TTMDV | Anelloviridae | 54.06 | 5 | 147 | 1743 | 3224 | AB303566 | Virosaurus | DNA library |
| 21404 | 5/20/2015 | 241_D | L001-r61 | TTMDV | Anelloviridae | 43.18 | 9 | 265 | 1381 | 3198 | AB303554 | Virosaurus | DNA library |
| 21405 | 5/20/2015 | 242_D | L003-r61 | TTMDV | Anelloviridae | 39.3 | 12 | 204 | 1248 | 3176 | AB303560 | Virosaurus | DNA library |
| 21419 | 5/21/2015 | 244_D | L005-r61 | TTMDV | Anelloviridae | 99.97 | 637 | 18816 | 3080 | 3081 | MN780420 | De novo | DNA library |
| 21420 | 5/21/2015 | 857_D | L8-r127 | TTMDV | Anelloviridae | 18.79 | 16 | 363 | 599 | 3188 | AB303562 | Virosaurus | DNA library |
| 21424 | 5/22/2015 | 245_D | L006-r61 | TTMDV | Anelloviridae | 82.08 | 11 | 301 | 2300 | 2802 | KF545588 | Virosaurus | DNA library |
| 21430 | 5/22/2015 | 246_D | L007-r61 | TTMDV | Anelloviridae | 16.68 | 5 | 23 | 409 | 2452 | KP343823 | Virosaurus | DNA library |
| 21433 | 5/22/2015 | 247_D | L008-r61 | TTMDV | Anelloviridae | 39.93 | 6 | 1062 | 1273 | 3188 | AB303562 | Virosaurus | DNA library |
| 21437 | 5/22/2015 | 774_D | L7-r111 | TTMDV | Anelloviridae | 38.51 | 5 | 145 | 1226 | 3184 | AB303565 | Virosaurus | DNA library |
| 21441 | 5/25/2015 | 248_D | L001-r61 | TTMDV | Anelloviridae | 35.16 | 2 | 63 | 1121 | 3188 | AB303562 | Virosaurus | DNA library |
| 21447 | 5/25/2015 | 249_D | L002-r61 | TTMDV | Anelloviridae | 23.52 | 7 | 170 | 752 | 3198 | AB303554 | Virosaurus | DNA library |
| 21449 | 5/25/2015 | 250_D | L004-r61 | TTMDV | Anelloviridae | 47.36 | 2 | 33 | 1327 | 2802 | KF545588 | Virosaurus | DNA library |
| 21453 | 5/26/2015 | 251_D | L005-r61 | TTMDV | Anelloviridae | 14.36 | 2 | 10 | 463 | 3224 | AB303558 | Virosaurus | DNA library |
| 21461 | 6/2/2015 | 858_D | L1-r132 | TTMDV | Anelloviridae | 40.34 | 4 | 1013 | 1290 | 3198 | AB303554 | Virosaurus | DNA library |
| 21468 | 5/27/2015 | 252_R | L008-r691 | TTMDV | Anelloviridae | 42.88 | 3 | 63 | 1375 | 3207 | MN774951 | De novo | RNA library |
| 21468 | 5/27/2015 | 252_D | L006-r61 | TTMDV | Anelloviridae | 18.42 | 11 | 211 | 598 | 3246 | NC_009225 | Virosaurus | DNA library |
| 21469 | 5/27/2015 | 593_R | L001-r723 | TTMDV | Anelloviridae | 10.99 | 4 | 12 | 338 | 3076 | MN780395 | De novo | RNA library |
| 21469 | 5/27/2015 | 593_D | L2-r92 | TTMDV | Anelloviridae | 86.54 | 54 | 2452 | 2759 | 3188 | KF545587 | Virosaurus | DNA library |
| 21471 | 5/28/2015 | 253_D | L007-r61 | TTMDV | Anelloviridae | 29.99 | 6 | 141 | 959 | 3198 | AB303554 | Virosaurus | DNA library |
| 21472 | 5/28/2015 | 254_R | L001-r692 | TTMDV | Anelloviridae | 29.24 | 8 | 62 | 784 | 2681 | MN777049 | De novo | RNA library |
| 21472 | 5/28/2015 | 254_D | L008-r61 | TTMDV | Anelloviridae | 57.1 | 3 | 887 | 1826 | 3198 | AB303554 | Virosaurus | DNA library |
| 21476 | 5/28/2015 | 255_D | L001-r61 | TTMDV | Anelloviridae | 17.03 | 1 | 8 | 541 | 3176 | AB303560 | Virosaurus | DNA library |
| 21477 | 5/28/2015 | 256_D | L002-r61 | TTMDV | Anelloviridae | 14.38 | 6 | 39 | 458 | 3184 | AB303565 | Virosaurus | DNA library |
| 21478 | 5/28/2015 | 257_D | L003-r61 | TTMDV | Anelloviridae | 99.61 | 396 | 12387 | 3078 | 3090 | MN778907 | De novo | DNA library |
| 21482 | 5/28/2015 | 258_D | L005-r61 | TTMDV | Anelloviridae | 43.29 | 32 | 499 | 1155 | 2668 | MN778687 | De novo | DNA library |
| 21488 | 5/29/2015 | 259_D | L006-r61 | TTMDV | Anelloviridae | 36.36 | 1 | 113 | 818 | 2250 | NC_007013 | Virosaurus | DNA library |
| 21491 | 5/29/2015 | 260_D | L007-r61 | TTMDV | Anelloviridae | 37.48 | 2 | 145 | 1195 | 3188 | KF545587 | Virosaurus | DNA library |
| 21493 | 5/29/2015 | 261_D | L008-r61 | TTMDV | Anelloviridae | 45.31 | 4 | 131 | 1449 | 3198 | AB303554 | Virosaurus | DNA library |
| 21504 | 6/1/2015 | 262_D | L001-r61 | TTMDV | Anelloviridae | 48.87 | 2 | 320 | 1558 | 3188 | KF545587 | Virosaurus | DNA library |
| 21508 | 6/1/2015 | 263_D | L002-r61 | TTMDV | Anelloviridae | 87.9 | 16 | 436 | 2463 | 2802 | KF545588 | Virosaurus | DNA library |
| 21513 | 6/1/2015 | 264_D | L003-r61 | TTMDV | Anelloviridae | 92.43 | 113 | 2837 | 2478 | 2681 | MN777049 | De novo | DNA library |
| 21514 | 6/1/2015 | 265_D | L004-r61 | TTMDV | Anelloviridae | 47.15 | 2 | 46 | 1520 | 3224 | AB303566 | Virosaurus | DNA library |
| 21518 | 6/3/2015 | 266_D | L006-r61 | TTMDV | Anelloviridae | 22.4 | 7 | 200 | 717 | 3201 | AB303556 | Virosaurus | DNA library |
| 21519 | 6/3/2015 | 267_D | L007-r61 | TTMDV | Anelloviridae | 21.54 | 2 | 66 | 684 | 3176 | AB303560 | Virosaurus | DNA library |
| 21521 | 6/4/2015 | 268_D | L008-r61 | TTMDV | Anelloviridae | 53.48 | 4 | 110 | 1705 | 3188 | AB303559 | Virosaurus | DNA library |
| 21523 | 6/4/2015 | 269_D | L001-r61 | TTMDV | Anelloviridae | 25.09 | 2 | 24 | 797 | 3176 | AB303560 | Virosaurus | DNA library |
| 21526 | 6/4/2015 | 270_D | L002-r61 | TTMDV | Anelloviridae | 30.38 | 2 | 38 | 809 | 2663 | KP343827 | Virosaurus | DNA library |
| 21528 | 6/5/2015 | 271_D | L1-r65 | TTMDV | Anelloviridae | 56.1 | 1 | 32 | 1572 | 2802 | KF545588 | Virosaurus | DNA library |
| 21533 | 6/5/2015 | 272_D | L2-r65 | TTMDV | Anelloviridae | 28.48 | 8 | 121 | 914 | 3209 | AB303552 | Virosaurus | DNA library |
| 21534 | 6/5/2015 | 273_D | L3-r65 | TTMDV | Anelloviridae | 13.26 | 3 | 17 | 424 | 3198 | AB303554 | Virosaurus | DNA library |
| 21542 | 6/8/2015 | 274_D | L4-r65 | TTMDV | Anelloviridae | 90.47 | 28 | 745 | 2296 | 2538 | KP343822 | Virosaurus | DNA library |
| 21548 | 6/9/2015 | 275_D | L5-r65 | TTMDV | Anelloviridae | 44.14 | 35 | 749 | 1402 | 3176 | AB303560 | Virosaurus | DNA library |
| 21556 | 6/10/2015 | 276_D | L6-r65 | TTMDV | Anelloviridae | 42.19 | 20 | 519 | 1340 | 3176 | AB303560 | Virosaurus | DNA library |
| 21557 | 6/11/2015 | 595_D | L1-r89 | TTMDV | Anelloviridae | 18.9 | 1 | 111 | 605 | 3201 | AB303556 | Virosaurus | DNA library |
| 21562 | 6/11/2015 | 277_D | L7-r65 | TTMDV | Anelloviridae | 19.81 | 2 | 17 | 643 | 3246 | NC_009225 | Virosaurus | DNA library |
| 21567 | 6/12/2015 | 278_D | L007-r67 | TTMDV | Anelloviridae | 15.73 | 5 | 34 | 503 | 3198 | AB303554 | Virosaurus | DNA library |
| 21568 | 6/12/2015 | 279_D | L2-r65 | TTMDV | Anelloviridae | 99.96 | 470 | 13523 | 2677 | 2678 | MN776612 | De novo | DNA library |
| 21571 | 6/15/2015 | 280_D | L3-r65 | TTMDV | Anelloviridae | 25.6 | 3 | 302 | 819 | 3199 | AB303553 | Virosaurus | DNA library |
| 21573 | 6/15/2015 | 281_D | L4-r65 | TTMDV | Anelloviridae | 97.76 | 22 | 1477 | 2972 | 3040 | MN775977 | De novo | DNA library |
| 21574 | 6/15/2015 | 282_D | L5-r65 | TTMDV | Anelloviridae | 68.58 | 32 | 790 | 1761 | 2568 | MN775653 | De novo | DNA library |
| 21584 | 6/17/2015 | 283_D | L6-r65 | TTMDV | Anelloviridae | 97.47 | 51 | 1336 | 2656 | 2725 | MN778879 | De novo | DNA library |
| 21591 | 6/17/2015 | 596_D | L2-r89 | TTMDV | Anelloviridae | 99.69 | 778 | 27343 | 3219 | 3229 | MN774952 | De novo | DNA library |
| 21601 | 6/18/2015 | 284_D | L7-r65 | TTMDV | Anelloviridae | 9.63 | 5 | 28 | 307 | 3188 | AB303562 | Virosaurus | DNA library |
| 21602 | 6/19/2015 | 285_D | L007-r67 | TTMDV | Anelloviridae | 31.21 | 2 | 36 | 995 | 3188 | AB303562 | Virosaurus | DNA library |
| 21606 | 6/22/2015 | 598_D | L4-r89 | TTMDV | Anelloviridae | 100 | 61 | 1842 | 2687 | 2687 | KP343821 | Virosaurus | DNA library |
| 21607 | 6/22/2015 | 286_D | L1-r65 | TTMDV | Anelloviridae | 17.17 | 4 | 87 | 549 | 3198 | AB303554 | Virosaurus | DNA library |
| 21609 | 6/22/2015 | 287_D | L3-r65 | TTMDV | Anelloviridae | 28.68 | 2 | 22 | 918 | 3201 | AB303556 | Virosaurus | DNA library |
| 21610 | 6/22/2015 | 288_D | L4-r65 | TTMDV | Anelloviridae | 29.62 | 1 | 226 | 796 | 2687 | KP343821 | Virosaurus | DNA library |
| 21622 | 6/24/2015 | 289_D | L5-r65 | TTMDV | Anelloviridae | 34.82 | 11 | 206 | 1106 | 3176 | AB303560 | Virosaurus | DNA library |
| 21627 | 6/24/2015 | 602_D | L1-r89 | TTMDV | Anelloviridae | 95.37 | 15 | 447 | 2881 | 3021 | MN778449 | De novo | DNA library |
| 21644 | 6/29/2015 | 611_R | L006-r723 | TTMDV | Anelloviridae | 58.04 | 14 | 228 | 1585 | 2731 | MN778180 | De novo | RNA library |
| 21644 | 6/29/2015 | 611_D | L3-r89 | TTMDV | Anelloviridae | 53.5 | 53 | 4520 | 1699 | 3176 | AB303560 | Virosaurus | DNA library |
| 21648 | 6/29/2015 | 291_D | L7-r65 | TTMDV | Anelloviridae | 15.42 | 4 | 30 | 493 | 3198 | AB303554 | Virosaurus | DNA library |
| 21649 | 6/29/2015 | 292_D | L8-r65 | TTMDV | Anelloviridae | 10.35 | 2 | 6 | 331 | 3198 | AB303554 | Virosaurus | DNA library |
| 21650 | 6/30/2015 | 293_D | L1-r65 | TTMDV | Anelloviridae | 86.38 | 5 | 168 | 2284 | 2644 | MN775455 | De novo | DNA library |
| 21656 | 7/1/2015 | 294_D | L2-r65 | TTMDV | Anelloviridae | 53.51 | 24 | 1983 | 1729 | 3231 | AB303564 | Virosaurus | DNA library |
| 21661 | 7/1/2015 | 614_D | L2-r92 | TTMDV | Anelloviridae | 34.97 | 6 | 110 | 1138 | 3254 | NC_014093 | Virosaurus | DNA library |
| 21664 | 7/1/2015 | 615_R | L007-r723 | TTMDV | Anelloviridae | 23.68 | 5 | 42 | 643 | 2716 | MN777952 | De novo | RNA library |
| 21664 | 7/1/2015 | 615_D | L7-r88 | TTMDV | Anelloviridae | 86.95 | 22 | 1372 | 2772 | 3188 | KF545587 | Virosaurus | DNA library |
| 21668 | 7/3/2015 | 616_D | L8-r88 | TTMDV | Anelloviridae | 51.97 | 3 | 504 | 1662 | 3198 | AB303554 | Virosaurus | DNA library |
| 21674 | 7/3/2015 | 290_D | L6-r65 | TTMDV | Anelloviridae | 16.98 | 16 | 96 | 543 | 3198 | AB303554 | Virosaurus | DNA library |
| 21675 | 7/3/2015 | 295_D | L4-r65 | TTMDV | Anelloviridae | 100 | 504 | 16585 | 2687 | 2687 | KP343821 | Virosaurus | DNA library |
| 21676 | 7/3/2015 | 296_D | L5-r65 | TTMDV | Anelloviridae | 35.95 | 1 | 41 | 966 | 2687 | KP343821 | Virosaurus | DNA library |
| 21691 | 7/8/2015 | 297_D | L6-r65 | TTMDV | Anelloviridae | 46.24 | 15 | 740 | 1474 | 3188 | AB303562 | Virosaurus | DNA library |
| 21701 | 7/9/2015 | 298_D | L7-r65 | TTMDV | Anelloviridae | 20.23 | 1 | 277 | 645 | 3188 | KF545587 | Virosaurus | DNA library |
| 21703 | 7/9/2015 | 617_D | L1-r89 | TTMDV | Anelloviridae | 53.67 | 2 | 715 | 1711 | 3188 | KF545587 | Virosaurus | DNA library |
| 21707 | 7/9/2015 | 299_D | L007-r67 | TTMDV | Anelloviridae | 23.05 | 1 | 25 | 737 | 3198 | AB303554 | Virosaurus | DNA library |
| 21710 | 7/9/2015 | 300_D | L1-r65 | TTMDV | Anelloviridae | 37.17 | 1 | 32 | 1185 | 3188 | AB303559 | Virosaurus | DNA library |
| 21712 | 7/9/2015 | 301_D | L2-r65 | TTMDV | Anelloviridae | 93.77 | 14 | 382 | 2619 | 2793 | KT163882 | Virosaurus | DNA library |
| 21713 | 7/9/2015 | 302_D | L3-r65 | TTMDV | Anelloviridae | 28.55 | 17 | 622 | 913 | 3198 | AB303554 | Virosaurus | DNA library |
| 21718 | 7/10/2015 | 304_D | L6-r65 | TTMDV | Anelloviridae | 85.35 | 17 | 678 | 2721 | 3188 | KF545587 | Virosaurus | DNA library |
| 21721 | 7/10/2015 | 305_D | L7-r65 | TTMDV | Anelloviridae | 12.29 | 1 | 22 | 393 | 3198 | AB303554 | Virosaurus | DNA library |
| 21722 | 7/10/2015 | 306_D | L007-r67 | TTMDV | Anelloviridae | 43.53 | 4 | 660 | 1392 | 3198 | AB303554 | Virosaurus | DNA library |
| 21737 | 7/14/2015 | 307_D | L1-r65 | TTMDV | Anelloviridae | 16.01 | 3 | 50 | 516 | 3224 | AB303558 | Virosaurus | DNA library |
| 21751 | 7/16/2015 | 308_D | L2-r65 | TTMDV | Anelloviridae | 14.73 | 9 | 63 | 471 | 3198 | AB303554 | Virosaurus | DNA library |
| 21755 | 7/16/2015 | 619_D | L4-r89 | TTMDV | Anelloviridae | 27.01 | 2 | 23 | 755 | 2795 | KP343824 | Virosaurus | DNA library |
| 21758 | 7/21/2015 | 624_D | L1-r89 | TTMDV | Anelloviridae | 86.22 | 8 | 236 | 2416 | 2802 | KF545588 | Virosaurus | DNA library |
| 21759 | 7/21/2015 | 309_D | L3-r65 | TTMDV | Anelloviridae | 80.26 | 4 | 170 | 2037 | 2538 | KP343822 | Virosaurus | DNA library |
| 21761 | 7/21/2015 | 623_D | L8-r88 | TTMDV | Anelloviridae | 99.61 | 1236 | 34563 | 3042 | 3054 | MN776487 | De novo | DNA library |
| 21766 | 7/21/2015 | 621_R | L008-r723 | TTMDV | Anelloviridae | 16.79 | 4 | 35 | 440 | 2621 | MN779380 | De novo | RNA library |
| 21766 | 7/21/2015 | 621_D | L2-r92 | TTMDV | Anelloviridae | 50.91 | 70 | 2798 | 1632 | 3206 | AB303555 | Virosaurus | DNA library |
| 21770 | 7/21/2015 | 625_D | L2-r89 | TTMDV | Anelloviridae | 90.58 | 18 | 533 | 2538 | 2802 | KF545588 | Virosaurus | DNA library |
| 21771 | 7/21/2015 | 311_D | L6-r65 | TTMDV | Anelloviridae | 78.14 | 3 | 171 | 2491 | 3188 | KF545587 | Virosaurus | DNA library |
| 21780 | 7/24/2015 | 626_R | L2-r724 | TTMDV | Anelloviridae | 14.66 | 4 | 12 | 344 | 2347 | MN775866 | De novo | RNA library |
| 21780 | 7/24/2015 | 626_D | L4-r89 | TTMDV | Anelloviridae | 89.4 | 4 | 159 | 2505 | 2802 | KF545588 | Virosaurus | DNA library |
| 21789 | 7/23/2015 | 627_R | L2-r724 | TTMDV | Anelloviridae | 24.57 | 3 | 24 | 750 | 3052 | MN780401 | De novo | RNA library |
| 21789 | 7/23/2015 | 627_D | L1-r92 | TTMDV | Anelloviridae | 65.76 | 7 | 362 | 2103 | 3198 | AB303554 | Virosaurus | DNA library |
| 21801 | 7/24/2015 | 312_D | L7-r65 | TTMDV | Anelloviridae | 20.19 | 2 | 54 | 646 | 3199 | AB303553 | Virosaurus | DNA library |
| 21812 | 7/28/2015 | 313_D | L007-r67 | TTMDV | Anelloviridae | 47.61 | 3 | 119 | 1535 | 3224 | AB303566 | Virosaurus | DNA library |
| 21816 | 7/28/2015 | 632_D | L2-r91 | TTMDV | Anelloviridae | 32.07 | 3 | 46 | 1021 | 3184 | AB303565 | Virosaurus | DNA library |
| 21818 | 7/30/2015 | 633_D | L3-r91 | TTMDV | Anelloviridae | 16.84 | 5 | 52 | 543 | 3224 | AB303558 | Virosaurus | DNA library |
| 21819 | 7/30/2015 | 314_R | L008-r693 | TTMDV | Anelloviridae | 12.71 | 3 | 10 | 327 | 2573 | MN780431 | De novo | RNA library |
| 21819 | 7/30/2015 | 314_D | L1-r65 | TTMDV | Anelloviridae | 32.02 | 19 | 1317 | 1024 | 3198 | AB303554 | Virosaurus | DNA library |
| 21837 | 8/3/2015 | 315_D | L2-r65 | TTMDV | Anelloviridae | 40.78 | 2 | 89 | 1300 | 3188 | AB303559 | Virosaurus | DNA library |
| 21838 | 8/3/2015 | 316_D | L1-r66 | TTMDV | Anelloviridae | 14.88 | 4 | 41 | 476 | 3198 | AB303554 | Virosaurus | DNA library |
| 21839 | 8/3/2015 | 317_D | L2-r66 | TTMDV | Anelloviridae | 19.68 | 2 | 26 | 636 | 3231 | AB303564 | Virosaurus | DNA library |
| 21843 | 8/3/2015 | 318_D | L3-r66 | TTMDV | Anelloviridae | 99.55 | 10 | 305 | 2675 | 2687 | KP343821 | Virosaurus | DNA library |
| 21849 | 8/4/2015 | 319_R | L002-r698 | TTMDV | Anelloviridae | 13.49 | 6 | 30 | 365 | 2705 | MN779647 | De novo | RNA library |
| 21849 | 8/4/2015 | 319_D | L4-r66 | TTMDV | Anelloviridae | 98.22 | 201 | 8995 | 2210 | 2250 | NC_007013 | Virosaurus | DNA library |
| 21851 | 8/4/2015 | 320_D | L5-r66 | TTMDV | Anelloviridae | 91.33 | 15 | 390 | 2318 | 2538 | KP343822 | Virosaurus | DNA library |
| 21858 | 8/5/2015 | 321_D | L6-r66 | TTMDV | Anelloviridae | 18.6 | 4 | 73 | 595 | 3199 | AB303553 | Virosaurus | DNA library |
| 21871 | 8/6/2015 | 323_D | L8-r66 | TTMDV | Anelloviridae | 20.3 | 2 | 32 | 647 | 3188 | AB303562 | Virosaurus | DNA library |
| 21875 | 8/6/2015 | 639_D | L2-r91 | TTMDV | Anelloviridae | 36.21 | 7 | 89 | 1150 | 3176 | AB303560 | Virosaurus | DNA library |
| 21877 | 8/7/2015 | 640_R | L6-r724 | TTMDV | Anelloviridae | 25.66 | 10 | 82 | 668 | 2603 | MN780312 | De novo | RNA library |
| 21877 | 8/7/2015 | 640_D | L3-r91 | TTMDV | Anelloviridae | 81.5 | 1 | 414 | 2190 | 2687 | KP343821 | Virosaurus | DNA library |
| 21879 | 8/7/2015 | 324_D | L2-r66 | TTMDV | Anelloviridae | 15.79 | 3 | 34 | 509 | 3224 | AB303558 | Virosaurus | DNA library |
| 21884 | 8/7/2015 | 325_D | L3-r66 | TTMDV | Anelloviridae | 100 | 17 | 601 | 2687 | 2687 | KP343821 | Virosaurus | DNA library |
| 21894 | 8/14/2015 | 779_D | L7-r113 | TTMDV | Anelloviridae | 68.44 | 6 | 519 | 2182 | 3188 | AB303559 | Virosaurus | DNA library |
| 21902 | 8/10/2015 | 326_D | L4-r66 | TTMDV | Anelloviridae | 74.95 | 64 | 2367 | 2014 | 2687 | KP343821 | Virosaurus | DNA library |
| 21903 | 8/10/2015 | 327_D | L5-r66 | TTMDV | Anelloviridae | 17.04 | 22 | 179 | 545 | 3198 | AB303554 | Virosaurus | DNA library |
| 21911 | 8/13/2015 | 322_D | L7-r66 | TTMDV | Anelloviridae | 15.23 | 2 | 10 | 487 | 3198 | AB303554 | Virosaurus | DNA library |
| 21920 | 8/14/2015 | 328_D | L6-r66 | TTMDV | Anelloviridae | 92.89 | 4 | 328 | 2863 | 3082 | MN780418 | De novo | DNA library |
| 21926 | 8/14/2015 | 329_D | L7-r66 | TTMDV | Anelloviridae | 14.42 | 5 | 29 | 461 | 3198 | AB303554 | Virosaurus | DNA library |
| 21937 | 8/18/2015 | 330_D | L8-r66 | TTMDV | Anelloviridae | 30.29 | 23 | 218 | 962 | 3176 | AB303560 | Virosaurus | DNA library |
| 21938 | 8/18/2015 | 331_D | L1-r66 | TTMDV | Anelloviridae | 37.85 | 46 | 671 | 1202 | 3176 | AB303560 | Virosaurus | DNA library |
| 21948 | 8/19/2015 | 332_D | L3-r66 | TTMDV | Anelloviridae | 31.33 | 4 | 73 | 995 | 3176 | AB303560 | Virosaurus | DNA library |
| 21949 | 8/19/2015 | 333_D | L4-r66 | TTMDV | Anelloviridae | 99.47 | 16909 | 507381 | 3212 | 3229 | MN774952 | De novo | DNA library |
| 21949 | 8/19/2015 | 333_R | L006-r698 | TTMDV | Anelloviridae | 11.89 | 1 | 7 | 384 | 3229 | MN774952 | De novo | RNA library |
| 21973 | 8/21/2015 | 334_D | L5-r66 | TTMDV | Anelloviridae | 63.1 | 26 | 1553 | 2018 | 3198 | AB303554 | Virosaurus | DNA library |
| 21975 | 8/24/2015 | 335_D | L6-r66 | TTMDV | Anelloviridae | 18.41 | 11 | 397 | 589 | 3199 | AB303553 | Virosaurus | DNA library |
| 21982 | 8/24/2015 | 336_D | L7-r66 | TTMDV | Anelloviridae | 15.25 | 13 | 104 | 486 | 3188 | AB303562 | Virosaurus | DNA library |
| 21987 | 8/25/2015 | 337_D | L8-r66 | TTMDV | Anelloviridae | 17.2 | 6 | 43 | 550 | 3198 | AB303554 | Virosaurus | DNA library |
| 21988 | 8/25/2015 | 338_D | L1-r66 | TTMDV | Anelloviridae | 18.29 | 8 | 131 | 585 | 3199 | AB303553 | Virosaurus | DNA library |
| 22001 | 8/26/2015 | 339_D | L2-r66 | TTMDV | Anelloviridae | 29.83 | 3 | 30 | 757 | 2538 | KP343822 | Virosaurus | DNA library |
| 22013 | 8/27/2015 | 340_D | L4-r66 | TTMDV | Anelloviridae | 100 | 102 | 3141 | 2687 | 2687 | KP343821 | Virosaurus | DNA library |
| 22038 | 9/1/2015 | 341_R | L008-r698 | TTMDV | Anelloviridae | 17.77 | 8 | 38 | 476 | 2678 | MN776933 | De novo | RNA library |
| 22038 | 9/1/2015 | 341_D | L5-r66 | TTMDV | Anelloviridae | 93.36 | 23 | 752 | 2616 | 2802 | KF545588 | Virosaurus | DNA library |
| 22041 | 9/1/2015 | 342_R | L008-r698 | TTMDV | Anelloviridae | 12.11 | 4 | 20 | 352 | 2907 | MN775975 | De novo | RNA library |
| 22041 | 9/1/2015 | 342_D | L6-r66 | TTMDV | Anelloviridae | 29.59 | 5 | 386 | 954 | 3224 | AB303566 | Virosaurus | DNA library |
| 22042 | 9/1/2015 | 650_R | L8-r724 | TTMDV | Anelloviridae | 9.98 | 5 | 32 | 316 | 3165 | MN780460 | De novo | RNA library |
| 22042 | 9/1/2015 | 650_D | L6-r91 | TTMDV | Anelloviridae | 19.98 | 2 | 80 | 639 | 3199 | AB303553 | Virosaurus | DNA library |
| 22049 | 9/2/2015 | 343_D | L7-r66 | TTMDV | Anelloviridae | 14.42 | 2 | 15 | 461 | 3198 | AB303554 | Virosaurus | DNA library |
| 22055 | 9/2/2015 | 344_D | L8-r66 | TTMDV | Anelloviridae | 9.57 | 2 | 5 | 306 | 3199 | AB303553 | Virosaurus | DNA library |
| 22062 | 9/3/2015 | 345_D | L1-r66 | TTMDV | Anelloviridae | 17.95 | 3 | 28 | 574 | 3198 | AB303554 | Virosaurus | DNA library |
| 22067 | 9/3/2015 | 346_D | L2-r66 | TTMDV | Anelloviridae | 95.11 | 49 | 1655 | 2665 | 2802 | KF545588 | Virosaurus | DNA library |
| 22068 | 9/3/2015 | 347_D | L3-r66 | TTMDV | Anelloviridae | 16.88 | 2 | 10 | 536 | 3176 | AB303560 | Virosaurus | DNA library |
| 22071 | 9/3/2015 | 348_D | L5-r66 | TTMDV | Anelloviridae | 27.61 | 3 | 93 | 883 | 3198 | AB303554 | Virosaurus | DNA library |
| 22079 | 9/7/2015 | 349_D | L6-r66 | TTMDV | Anelloviridae | 23.75 | 5 | 140 | 771 | 3246 | NC_009225 | Virosaurus | DNA library |
| 22084 | 9/7/2015 | 350_D | L7-r66 | TTMDV | Anelloviridae | 22.95 | 5 | 169 | 740 | 3224 | AB303558 | Virosaurus | DNA library |
| 22093 | 9/8/2015 | 859_D | L2-r132 | TTMDV | Anelloviridae | 100 | 1342 | 43914 | 2687 | 2687 | KP343821 | Virosaurus | DNA library |
| 22095 | 9/8/2015 | 860_D | L3-r132 | TTMDV | Anelloviridae | 34.41 | 5 | 91 | 1097 | 3188 | AB303562 | Virosaurus | DNA library |
| 22107 | 9/9/2015 | 352_D | L1-r66 | TTMDV | Anelloviridae | 42.47 | 2 | 2744 | 1354 | 3188 | KF545587 | Virosaurus | DNA library |
| 22108 | 9/9/2015 | 353_D | L2-r66 | TTMDV | Anelloviridae | 83.09 | 10 | 1373 | 2649 | 3188 | KF545587 | Virosaurus | DNA library |
| 22110 | 9/9/2015 | 354_D | L3-r66 | TTMDV | Anelloviridae | 38.09 | 1 | 29 | 1218 | 3198 | AB303554 | Virosaurus | DNA library |
| 22111 | 9/9/2015 | 355_D | L4-r66 | TTMDV | Anelloviridae | 40.43 | 4 | 91 | 1289 | 3188 | AB303562 | Virosaurus | DNA library |
| 22117 | 9/9/2015 | 655_D | L4-r91 | TTMDV | Anelloviridae | 68.13 | 2 | 416 | 2200 | 3229 | MN774952 | De novo | DNA library |
| 22126 | 9/10/2015 | 356_D | L6-r66 | TTMDV | Anelloviridae | 96.23 | 51 | 1350 | 2581 | 2682 | MN780031 | De novo | DNA library |
| 22152 | 9/14/2015 | 660_D | L1-r91 | TTMDV | Anelloviridae | 19.16 | 8 | 258 | 613 | 3199 | AB303553 | Virosaurus | DNA library |
| 22163 | 9/15/2015 | 357_D | L7-r66 | TTMDV | Anelloviridae | 50.02 | 2 | 166 | 1344 | 2687 | KP343821 | Virosaurus | DNA library |
| 22172 | 9/16/2015 | 358_D | L8-r66 | TTMDV | Anelloviridae | 19.79 | 7 | 284 | 638 | 3224 | AB303558 | Virosaurus | DNA library |
| 22179 | 9/17/2015 | 667_D | L1-r91 | TTMDV | Anelloviridae | 70.33 | 2 | 485 | 2242 | 3188 | KF545587 | Virosaurus | DNA library |
| 22185 | 9/17/2015 | 359_D | L1-r66 | TTMDV | Anelloviridae | 42.73 | 22 | 544 | 1357 | 3176 | AB303560 | Virosaurus | DNA library |
| 22190 | 9/18/2015 | 781_D | L6-r111 | TTMDV | Anelloviridae | 27.62 | 3 | 45 | 701 | 2538 | KP343822 | Virosaurus | DNA library |
| 22191 | 9/18/2015 | 360_D | L2-r66 | TTMDV | Anelloviridae | 31.09 | 5 | 99 | 789 | 2538 | KP343822 | Virosaurus | DNA library |
| 22195 | 9/18/2015 | 361_D | L001-r71 | TTMDV | Anelloviridae | 87.15 | 13 | 367 | 2442 | 2802 | KF545588 | Virosaurus | DNA library |
| 22196 | 9/18/2015 | 362_D | L002-r71 | TTMDV | Anelloviridae | 85.86 | 9 | 238 | 2179 | 2538 | KP343822 | Virosaurus | DNA library |
| 22198 | 9/18/2015 | 363_D | L003-r71 | TTMDV | Anelloviridae | 98.08 | 54 | 1985 | 3015 | 3074 | MN780383 | De novo | DNA library |
| 22201 | 9/21/2015 | 364_D | L004-r71 | TTMDV | Anelloviridae | 13.13 | 11 | 138 | 420 | 3198 | AB303554 | Virosaurus | DNA library |
| 22203 | 9/21/2015 | 365_D | L001-r73 | TTMDV | Anelloviridae | 47.59 | 3 | 195 | 1517 | 3188 | AB303559 | Virosaurus | DNA library |
| 22206 | 9/21/2015 | 366_D | L002-r73 | TTMDV | Anelloviridae | 95.42 | 1693 | 49129 | 3081 | 3229 | MN774940 | De novo | DNA library |
| 22211 | 9/22/2015 | 367_R | L007-r702 | TTMDV | Anelloviridae | 75.01 | 12 | 294 | 2332 | 3109 | MN780399 | De novo | RNA library |
| 22211 | 9/22/2015 | 367_D | L003-r73 | TTMDV | Anelloviridae | 41.03 | 56 | 1013 | 1303 | 3176 | AB303560 | Virosaurus | DNA library |
| 22212 | 9/22/2015 | 368_D | L004-r73 | TTMDV | Anelloviridae | 99.28 | 34 | 2088 | 3011 | 3033 | MN774984 | De novo | DNA library |
| 22212 | 9/22/2015 | 368_R | L007-r702 | TTMDV | Anelloviridae | 12.79 | 2 | 8 | 306 | 2392 | MN775775 | De novo | RNA library |
| 22215 | 9/22/2015 | 369_D | L002-r71 | TTMDV | Anelloviridae | 47.79 | 2 | 164 | 1213 | 2538 | KP343822 | Virosaurus | DNA library |
| 22232 | 9/22/2015 | 370_D | L003-r71 | TTMDV | Anelloviridae | 100 | 4632 | 133549 | 2687 | 2687 | KP343821 | Virosaurus | DNA library |
| 22250 | 9/28/2015 | 372_D | L001-r73 | TTMDV | Anelloviridae | 87.61 | 24 | 821 | 2793 | 3188 | KF545587 | Virosaurus | DNA library |
| 22251 | 9/28/2015 | 373_D | L002-r73 | TTMDV | Anelloviridae | 98.87 | 151 | 5444 | 3159 | 3195 | MN774944 | De novo | DNA library |
| 22259 | 9/29/2015 | 374_D | L003-r73 | TTMDV | Anelloviridae | 91.91 | 23 | 645 | 2748 | 2990 | MN778866 | De novo | DNA library |
| 22274 | 10/9/2015 | 376_D | L001-r71 | TTMDV | Anelloviridae | 93.46 | 379 | 10765 | 2372 | 2538 | KP343822 | Virosaurus | DNA library |
| 22276 | 10/9/2015 | 377_D | L003-r71 | TTMDV | Anelloviridae | 91.3 | 28 | 1146 | 2844 | 3115 | MN778884 | De novo | DNA library |
| 22277 | 10/12/2015 | 782_R | L7-r737 | TTMDV | Anelloviridae | 88.68 | 14 | 471 | 2451 | 2764 | MN777134 | De novo | RNA library |
| 22277 | 10/12/2015 | 782_D | L8-r111 | TTMDV | Anelloviridae | 61.29 | 3 | 590 | 1960 | 3198 | AB303554 | Virosaurus | DNA library |
| 22278 | 10/15/2015 | 783_D | L4-r113 | TTMDV | Anelloviridae | 25.4 | 3 | 67 | 819 | 3224 | AB303558 | Virosaurus | DNA library |
| 22283 | 10/19/2015 | 784_D | L5-r113 | TTMDV | Anelloviridae | 71.15 | 24 | 759 | 2195 | 3085 | MN776314 | De novo | DNA library |
| 22285 | 10/15/2015 | 378_D | L004-r71 | TTMDV | Anelloviridae | 24.04 | 2 | 19 | 610 | 2538 | KP343822 | Virosaurus | DNA library |
| 22292 | 10/19/2015 | 380_D | L002-r73 | TTMDV | Anelloviridae | 93.85 | 930 | 20925 | 2382 | 2538 | KP343822 | Virosaurus | DNA library |
| 22293 | 10/19/2015 | 381_D | L003-r73 | TTMDV | Anelloviridae | 99.97 | 122 | 3415 | 3048 | 3049 | MN778394 | De novo | DNA library |
| 22295 | 10/20/2015 | 382_D | L004-r73 | TTMDV | Anelloviridae | 98.67 | 425 | 11925 | 2964 | 3004 | MN775492 | De novo | DNA library |
| 22297 | 10/20/2015 | 384_D | L002-r71 | TTMDV | Anelloviridae | 91.75 | 13 | 347 | 2779 | 3029 | MN776513 | De novo | DNA library |
| 22301 | 10/12/2015 | 385_D | L004-r71 | TTMDV | Anelloviridae | 48.15 | 2 | 115 | 1535 | 3188 | KF545587 | Virosaurus | DNA library |
| 22305 | 10/13/2015 | 386_D | L001-r73 | TTMDV | Anelloviridae | 17.07 | 10 | 77 | 546 | 3198 | AB303554 | Virosaurus | DNA library |
| 22319 | 10/20/2015 | 387_D | L002-r73 | TTMDV | Anelloviridae | 92.53 | 6 | 185 | 2812 | 3039 | MN780423 | De novo | DNA library |
| 22321 | 10/20/2015 | 388_D | L003-r73 | TTMDV | Anelloviridae | 96.4 | 82 | 12208 | 2169 | 2250 | NC_007013 | Virosaurus | DNA library |
| 22321 | 10/20/2015 | 388_R | L004-r703 | TTMDV | Anelloviridae | 23.87 | 1 | 14 | 537 | 2250 | NC_007013 | Virosaurus | RNA library |
| 22326 | 10/22/2015 | 389_D | L004-r73 | TTMDV | Anelloviridae | 99.25 | 2214 | 60418 | 3161 | 3185 | MN774949 | De novo | DNA library |
| 22346 | 11/2/2015 | 32_D | L003-r44 | TTMDV | Anelloviridae | 82.36 | 21 | 638 | 2245 | 2726 | MN775032 | De novo | DNA library |
| 22349 | 11/2/2015 | 11_D | L004-r44 | TTMDV | Anelloviridae | 43.93 | 5 | 2620 | 1405 | 3198 | AB303554 | Virosaurus | DNA library |
| 22359 | 11/6/2015 | 390_D | L001-r71 | TTMDV | Anelloviridae | 99.18 | 8190 | 221989 | 3035 | 3060 | MN774955 | De novo | DNA library |
| 22382 | 11/18/2015 | 391_D | L002-r71 | TTMDV | Anelloviridae | 99.78 | 16601 | 458220 | 3174 | 3181 | MN774943 | De novo | DNA library |
| 22383 | 11/20/2015 | 789_D | L7-r111 | TTMDV | Anelloviridae | 42.09 | 3 | 212 | 1346 | 3198 | AB303554 | Virosaurus | DNA library |
| 22401 | 10/21/2015 | 392_D | L003-r71 | TTMDV | Anelloviridae | 98.92 | 3910 | 111502 | 3034 | 3067 | MN780419 | De novo | DNA library |
| 22405 | 10/22/2015 | 393_D | L001-r73 | TTMDV | Anelloviridae | 40.27 | 11 | 241 | 1279 | 3176 | AB303560 | Virosaurus | DNA library |
| 22406 | 10/22/2015 | 394_D | L002-r73 | TTMDV | Anelloviridae | 97.19 | 31 | 1828 | 2909 | 2993 | MN776993 | De novo | DNA library |
| 22409 | 10/23/2015 | 396_D | L004-r73 | TTMDV | Anelloviridae | 93.02 | 1108 | 26802 | 2452 | 2636 | NC_007014 | Virosaurus | DNA library |
| 22411 | 10/27/2015 | 31_R | L001-r674 | TTMDV | Anelloviridae | 30.41 | 2 | 24 | 937 | 3081 | MN780420 | De novo | RNA library |
| 22411 | 10/27/2015 | 31_D | L002-r44 | TTMDV | Anelloviridae | 74.2 | 20 | 1754 | 2373 | 3198 | AB303554 | Virosaurus | DNA library |
| 22413 | 10/27/2015 | 37_D | L001-r44 | TTMDV | Anelloviridae | 98 | 241 | 17667 | 3034 | 3096 | MN775978 | De novo | DNA library |
| 22414 | 10/30/2015 | 45_D | L002-r44 | TTMDV | Anelloviridae | 32.08 | 3 | 37 | 1019 | 3176 | AB303560 | Virosaurus | DNA library |
| 22415 | 10/28/2015 | 10_D | L003-r44 | TTMDV | Anelloviridae | 82.93 | 16 | 1436 | 1866 | 2250 | NC_007013 | Virosaurus | DNA library |
| 22418 | 10/29/2015 | 668_D | L2-r91 | TTMDV | Anelloviridae | 10.67 | 3 | 21 | 340 | 3188 | AB303562 | Virosaurus | DNA library |
| 22419 | 10/29/2015 | 785_D | L6-r113 | TTMDV | Anelloviridae | 50.63 | 3 | 683 | 1619 | 3198 | AB303554 | Virosaurus | DNA library |
| 22423 | 11/2/2015 | 38_D | L002-r44 | TTMDV | Anelloviridae | 16.73 | 31 | 258 | 535 | 3198 | AB303554 | Virosaurus | DNA library |
| 22424 | 11/2/2015 | 19_R | L6-r669 | TTMDV | Anelloviridae | 44.63 | 4 | 63 | 1368 | 3065 | MN774936 | De novo | RNA library |
| 22424 | 11/2/2015 | 19_D | L005-r44 | TTMDV | Anelloviridae | 20.78 | 29 | 409 | 665 | 3201 | AB303556 | Virosaurus | DNA library |
| 22431 | 11/4/2015 | 397_D | L001-r71 | TTMDV | Anelloviridae | 92.26 | 128 | 3248 | 2823 | 3060 | MN778838 | De novo | DNA library |
| 22432 | 11/4/2015 | 398_R | L007-r703 | TTMDV | Anelloviridae | 17.53 | 16 | 88 | 531 | 3029 | MN776513 | De novo | RNA library |
| 22432 | 11/4/2015 | 398_D | L002-r71 | TTMDV | Anelloviridae | 93.85 | 604 | 15008 | 2382 | 2538 | KP343822 | Virosaurus | DNA library |
| 22435 | 11/6/2015 | 39_D | L003-r44 | TTMDV | Anelloviridae | 80.27 | 3 | 656 | 1806 | 2250 | NC_007013 | Virosaurus | DNA library |
| 22436 | 11/6/2015 | 13_D | L006-r44 | TTMDV | Anelloviridae | 18.26 | 4 | 267 | 584 | 3199 | AB303553 | Virosaurus | DNA library |
| 22437 | 11/6/2015 | 399_D | L003-r71 | TTMDV | Anelloviridae | 99.79 | 4295 | 110066 | 2861 | 2867 | MN780003 | De novo | DNA library |
| 22442 | 11/11/2015 | 23_D | L001-r44 | TTMDV | Anelloviridae | 91.39 | 70 | 3345 | 2792 | 3055 | MN775188 | De novo | DNA library |
| 22444 | 11/11/2015 | 29_D | L008-r44 | TTMDV | Anelloviridae | 20.51 | 32 | 387 | 656 | 3198 | AB303554 | Virosaurus | DNA library |
| 22448 | 11/12/2015 | 788_D | L6-r111 | TTMDV | Anelloviridae | 21.83 | 1 | 7 | 554 | 2538 | KP343822 | Virosaurus | DNA library |
| 22453 | 11/16/2015 | 400_D | L004-r71 | TTMDV | Anelloviridae | 16.89 | 4 | 27 | 540 | 3198 | AB303554 | Virosaurus | DNA library |
| 22457 | 11/16/2015 | 401_D | L002-r73 | TTMDV | Anelloviridae | 99.12 | 275 | 9334 | 3137 | 3165 | MN780460 | De novo | DNA library |
| 22460 | 11/17/2015 | 16_D | L001-r44 | TTMDV | Anelloviridae | 90.67 | 22 | 679 | 2807 | 3096 | MN775978 | De novo | DNA library |
| 22461 | 11/17/2015 | 24_D | L002-r44 | TTMDV | Anelloviridae | 97.41 | 141 | 3651 | 2590 | 2659 | MN779670 | De novo | DNA library |
| 22462 | 11/18/2015 | 35_R | L001-r674 | TTMDV | Anelloviridae | 16.38 | 2 | 16 | 493 | 3010 | MN774977 | De novo | RNA library |
| 22462 | 11/18/2015 | 35_D | L007-r44 | TTMDV | Anelloviridae | 95.92 | 61 | 1696 | 2679 | 2793 | KT163882 | Virosaurus | DNA library |
| 22463 | 11/18/2015 | 42_D | L007-r44 | TTMDV | Anelloviridae | 18.24 | 5 | 46 | 592 | 3246 | NC_009225 | Virosaurus | DNA library |
| 22473 | 11/23/2015 | 402_D | L003-r73 | TTMDV | Anelloviridae | 100 | 62 | 15624 | 3056 | 3056 | MN780387 | De novo | DNA library |
| 22476 | 11/24/2015 | 46_D | L001-r46 | TTMDV | Anelloviridae | 95.38 | 43 | 1107 | 2499 | 2620 | MN778135 | De novo | DNA library |
| 22481 | 11/24/2015 | 662_D | L3-r91 | TTMDV | Anelloviridae | 38.46 | 2 | 102 | 1230 | 3198 | AB303554 | Virosaurus | DNA library |
| 22482 | 11/27/2015 | 791_D | L5-r113 | TTMDV | Anelloviridae | 66.64 | 8 | 287 | 2131 | 3198 | AB303554 | Virosaurus | DNA library |
| 22484 | 11/26/2015 | 82_D | L001-r46 | TTMDV | Anelloviridae | 97.82 | 4158 | 121035 | 3047 | 3115 | MN778884 | De novo | DNA library |
| 22485 | 11/26/2015 | 53_D | L008-r46 | TTMDV | Anelloviridae | 16.64 | 40 | 257 | 532 | 3198 | AB303554 | Virosaurus | DNA library |
| 22486 | 8/6/2015 | 59_D | L007-r46 | TTMDV | Anelloviridae | 99.61 | 100 | 3250 | 3075 | 3087 | MN776317 | De novo | DNA library |
| 22488 | 11/26/2015 | 47_D | L002-r46 | TTMDV | Anelloviridae | 66.67 | 4 | 180 | 2132 | 3198 | AB303554 | Virosaurus | DNA library |
| 22494 | 12/1/2015 | 78_D | L005-r46 | TTMDV | Anelloviridae | 96.9 | 66 | 2192 | 2972 | 3067 | MN780375 | De novo | DNA library |
| 22495 | 11/24/2015 | 403_D | L004-r73 | TTMDV | Anelloviridae | 45.81 | 2 | 600 | 1477 | 3224 | AB303566 | Virosaurus | DNA library |
| 22499 | 12/2/2015 | 83_D | L002-r46 | TTMDV | Anelloviridae | 44.57 | 13 | 827 | 1440 | 3231 | AB303564 | Virosaurus | DNA library |
| 22516 | 12/2/2015 | 48_D | L003-r46 | TTMDV | Anelloviridae | 16.23 | 14 | 124 | 519 | 3198 | AB303554 | Virosaurus | DNA library |
| 22518 | 12/3/2015 | 792_D | L6-r113 | TTMDV | Anelloviridae | 95.34 | 35 | 1221 | 2963 | 3108 | MN780389 | De novo | DNA library |
| 22531 | 12/11/2015 | 65_D | L006-r46 | TTMDV | Anelloviridae | 32.79 | 2 | 24 | 1057 | 3224 | AB303566 | Virosaurus | DNA library |
| 22603 | 12/3/2015 | 71_D | L005-r46 | TTMDV | Anelloviridae | 98.66 | 1718 | 50550 | 2933 | 2973 | MN776384 | De novo | DNA library |
| 22605 | 12/3/2015 | 64_D | L005-r46 | TTMDV | Anelloviridae | 93.3 | 344 | 10125 | 2368 | 2538 | KP343822 | Virosaurus | DNA library |
| 22606 | 12/4/2015 | 404_D | L001-r71 | TTMDV | Anelloviridae | 89.14 | 19 | 1104 | 2593 | 2909 | MN779905 | De novo | DNA library |
| 22610 | 12/7/2015 | 89_D | L001-r46 | TTMDV | Anelloviridae | 70.01 | 17 | 437 | 2239 | 3198 | AB303554 | Virosaurus | DNA library |
| 22618 | 12/10/2015 | 49_D | L004-r46 | TTMDV | Anelloviridae | 18.61 | 5 | 54 | 604 | 3246 | NC_009225 | Virosaurus | DNA library |
| 22622 | 1/8/2016 | 72_D | L006-r46 | TTMDV | Anelloviridae | 17.07 | 16 | 119 | 546 | 3198 | AB303554 | Virosaurus | DNA library |
| 22623 | 1/7/2016 | 79_D | L006-r46 | TTMDV | Anelloviridae | 45.88 | 4 | 69 | 1479 | 3224 | AB303566 | Virosaurus | DNA library |
| 22624 | 1/7/2016 | 75_D | L001-r46 | TTMDV | Anelloviridae | 91.7 | 15 | 396 | 2331 | 2542 | KP343826 | Virosaurus | DNA library |
| 22628 | 1/11/2016 | 793_D | L7-r113 | TTMDV | Anelloviridae | 16.94 | 5 | 57 | 550 | 3246 | NC_009225 | Virosaurus | DNA library |
| 22639 | 1/18/2016 | 51_D | L006-r46 | TTMDV | Anelloviridae | 20.69 | 24 | 872 | 662 | 3199 | AB303553 | Virosaurus | DNA library |
| 22644 | 1/18/2016 | 56_D | L004-r46 | TTMDV | Anelloviridae | 20.38 | 3 | 21 | 657 | 3224 | AB303566 | Virosaurus | DNA library |
| 22648 | 1/19/2016 | 80_D | L007-r46 | TTMDV | Anelloviridae | 34.48 | 10 | 131 | 1095 | 3176 | AB303560 | Virosaurus | DNA library |
| 22652 | 1/20/2016 | 66_D | L007-r46 | TTMDV | Anelloviridae | 37.15 | 2 | 135 | 1188 | 3198 | AB303554 | Virosaurus | DNA library |
| 22653 | 1/21/2016 | 73_D | L007-r46 | TTMDV | Anelloviridae | 16.26 | 6 | 46 | 520 | 3198 | AB303554 | Virosaurus | DNA library |
| 22654 | 1/21/2016 | 665_D | L7-r91 | TTMDV | Anelloviridae | 20.35 | 8 | 188 | 651 | 3199 | AB303553 | Virosaurus | DNA library |
| 22663 | 1/27/2016 | 57_D | L005-r46 | TTMDV | Anelloviridae | 33.18 | 7 | 682 | 842 | 2538 | KP343822 | Virosaurus | DNA library |
| 22665 | 1/27/2016 | 86_D | L006-r46 | TTMDV | Anelloviridae | 38.14 | 35 | 796 | 1241 | 3254 | NC_014093 | Virosaurus | DNA library |
| 22667 | 3/20/2015 | 62_D | L003-r46 | TTMDV | Anelloviridae | 17.57 | 27 | 189 | 562 | 3198 | AB303554 | Virosaurus | DNA library |
| 22668 | 1/28/2016 | 85_D | L004-r46 | TTMDV | Anelloviridae | 16.7 | 9 | 80 | 534 | 3198 | AB303554 | Virosaurus | DNA library |
| 22672 | 2/1/2016 | 666_D | L8-r91 | TTMDV | Anelloviridae | 75.05 | 17 | 816 | 2400 | 3198 | AB303554 | Virosaurus | DNA library |
| 22675 | 2/2/2016 | 81_D | L008-r46 | TTMDV | Anelloviridae | 82.87 | 17 | 872 | 2642 | 3188 | KF545587 | Virosaurus | DNA library |
| 22680 | 2/3/2016 | 76_D | L002-r46 | TTMDV | Anelloviridae | 67.85 | 3 | 149 | 1823 | 2687 | KP343821 | Virosaurus | DNA library |
| 22685 | 2/5/2016 | 58_D | L006-r46 | TTMDV | Anelloviridae | 60.79 | 6 | 176 | 1938 | 3188 | AB303559 | Virosaurus | DNA library |
| 22686 | 2/9/2016 | 63_D | L004-r46 | TTMDV | Anelloviridae | 11.17 | 1 | 5 | 360 | 3224 | AB303558 | Virosaurus | DNA library |
| 22689 | 2/9/2016 | 68_D | L001-r46 | TTMDV | Anelloviridae | 93.85 | 438 | 15544 | 2382 | 2538 | KP343822 | Virosaurus | DNA library |
| 25003 | 12/11/2014 | 407_D | L006-r73 | TTMDV | Anelloviridae | 61.17 | 14 | 290 | 1651 | 2699 | MN775076 | De novo | DNA library |
| 25004 | 1/15/2015 | 408_D | L001-r75 | TTMDV | Anelloviridae | 18.32 | 15 | 120 | 586 | 3198 | AB303554 | Virosaurus | DNA library |
| 25008 | 1/15/2015 | 409_D | L002-r75 | TTMDV | Anelloviridae | 34.45 | 4 | 61 | 1094 | 3176 | AB303560 | Virosaurus | DNA library |
| 25015 | 1/19/2015 | 410_D | L003-r75 | TTMDV | Anelloviridae | 34.73 | 11 | 115 | 1103 | 3176 | AB303560 | Virosaurus | DNA library |
| 25016 | 1/19/2015 | 411_D | L004-r75 | TTMDV | Anelloviridae | 31.83 | 3 | 42 | 1011 | 3176 | AB303560 | Virosaurus | DNA library |
| 25023 | 1/20/2015 | 412_D | L005-r75 | TTMDV | Anelloviridae | 40.33 | 1 | 19 | 1130 | 2802 | KF545588 | Virosaurus | DNA library |
| 25025 | 1/21/2015 | 413_D | L006-r75 | TTMDV | Anelloviridae | 79.64 | 2 | 814 | 1792 | 2250 | NC_007013 | Virosaurus | DNA library |
| 25026 | 1/21/2015 | 414_D | L006-r73 | TTMDV | Anelloviridae | 45.42 | 2 | 358 | 1448 | 3188 | KF545587 | Virosaurus | DNA library |
| 25029 | 1/21/2015 | 795_D | L6-r111 | TTMDV | Anelloviridae | 9.98 | 4 | 18 | 319 | 3198 | AB303554 | Virosaurus | DNA library |
| 25033 | 1/22/2015 | 415_D | L001-r75 | TTMDV | Anelloviridae | 37.68 | 3 | 51 | 1206 | 3201 | AB303556 | Virosaurus | DNA library |
| 25034 | 1/22/2015 | 416_D | L002-r75 | TTMDV | Anelloviridae | 25.06 | 1 | 16 | 808 | 3224 | AB303566 | Virosaurus | DNA library |
| 25037 | 1/23/2015 | 417_D | L003-r75 | TTMDV | Anelloviridae | 82.12 | 7 | 225 | 2535 | 3087 | MN778834 | De novo | DNA library |
| 25055 | 1/30/2015 | 418_D | L004-r75 | TTMDV | Anelloviridae | 89.51 | 3 | 103 | 2405 | 2687 | KP343821 | Virosaurus | DNA library |
| 25056 | 1/30/2015 | 419_D | L005-r75 | TTMDV | Anelloviridae | 50.84 | 3 | 61 | 1144 | 2250 | NC_007013 | Virosaurus | DNA library |
| 25081 | 2/10/2015 | 420_D | L006-r75 | TTMDV | Anelloviridae | 18.27 | 30 | 211 | 589 | 3224 | AB303566 | Virosaurus | DNA library |
| 25083 | 2/11/2015 | 421_D | L005-r73 | TTMDV | Anelloviridae | 17.67 | 57 | 464 | 565 | 3198 | AB303554 | Virosaurus | DNA library |
| 25085 | 2/12/2015 | 422_D | L001-r75 | TTMDV | Anelloviridae | 14.27 | 7 | 35 | 455 | 3188 | KF545587 | Virosaurus | DNA library |
| 25086 | 2/12/2015 | 423_D | L002-r75 | TTMDV | Anelloviridae | 21.62 | 8 | 171 | 697 | 3224 | AB303558 | Virosaurus | DNA library |
| 25089 | 2/16/2015 | 424_D | L003-r75 | TTMDV | Anelloviridae | 18.36 | 4 | 26 | 583 | 3176 | AB303560 | Virosaurus | DNA library |
| 25090 | 2/16/2015 | 425_D | L004-r75 | TTMDV | Anelloviridae | 96.93 | 86 | 2329 | 2561 | 2642 | MN778616 | De novo | DNA library |
| 25093 | 2/16/2015 | 426_D | L005-r75 | TTMDV | Anelloviridae | 19.39 | 7 | 153 | 618 | 3188 | AB303562 | Virosaurus | DNA library |
| 25097 | 2/17/2015 | 427_D | L006-r75 | TTMDV | Anelloviridae | 15.97 | 2 | 34 | 515 | 3224 | AB303558 | Virosaurus | DNA library |
| 25103 | 2/18/2015 | 429_D | L006-r73 | TTMDV | Anelloviridae | 20.54 | 28 | 208 | 657 | 3198 | AB303554 | Virosaurus | DNA library |
| 25107 | 2/18/2015 | 430_D | L002-r75 | TTMDV | Anelloviridae | 13.32 | 1 | 30 | 426 | 3199 | AB303553 | Virosaurus | DNA library |
| 25110 | 2/19/2015 | 431_D | L003-r75 | TTMDV | Anelloviridae | 17.57 | 46 | 297 | 562 | 3198 | AB303554 | Virosaurus | DNA library |
| 25111 | 2/19/2015 | 432_D | L004-r75 | TTMDV | Anelloviridae | 15.09 | 8 | 68 | 481 | 3188 | AB303562 | Virosaurus | DNA library |
| 25114 | 2/20/2015 | 434_D | L006-r75 | TTMDV | Anelloviridae | 44.92 | 4 | 241 | 1438 | 3201 | AB303556 | Virosaurus | DNA library |
| 25117 | 2/20/2015 | 435_D | L005-r73 | TTMDV | Anelloviridae | 30.54 | 3 | 40 | 970 | 3176 | AB303560 | Virosaurus | DNA library |
| 25121 | 2/23/2015 | 436_D | L006-r73 | TTMDV | Anelloviridae | 24.31 | 2 | 19 | 617 | 2538 | KP343822 | Virosaurus | DNA library |
| 25137 | 2/26/2015 | 437_D | L001-r75 | TTMDV | Anelloviridae | 17.39 | 11 | 1129 | 556 | 3198 | AB303554 | Virosaurus | DNA library |
| 25147 | 3/2/2015 | 440_D | L005-r75 | TTMDV | Anelloviridae | 18.04 | 35 | 274 | 577 | 3198 | AB303554 | Virosaurus | DNA library |
| 25150 | 3/3/2015 | 441_D | L006-r75 | TTMDV | Anelloviridae | 18.79 | 48 | 495 | 601 | 3198 | AB303554 | Virosaurus | DNA library |
| 25151 | 3/3/2015 | 442_D | L005-r73 | TTMDV | Anelloviridae | 61.38 | 7 | 662 | 1963 | 3198 | AB303554 | Virosaurus | DNA library |
| 25156 | 3/4/2015 | 443_D | L006-r73 | TTMDV | Anelloviridae | 9.48 | 3 | 13 | 303 | 3198 | AB303554 | Virosaurus | DNA library |
| 25160 | 3/5/2015 | 444_D | L001-r75 | TTMDV | Anelloviridae | 82.21 | 2 | 54 | 2209 | 2687 | KP343821 | Virosaurus | DNA library |
| 25161 | 3/5/2015 | 587_D | L8-r88 | TTMDV | Anelloviridae | 97.96 | 598 | 28161 | 2204 | 2250 | NC_007013 | Virosaurus | DNA library |
| 25161 | 3/5/2015 | 587_R | L006-r722 | TTMDV | Anelloviridae | 25.2 | 1 | 8 | 567 | 2250 | NC_007013 | Virosaurus | RNA library |
| 25162 | 3/5/2015 | 445_D | L002-r75 | TTMDV | Anelloviridae | 59.55 | 8 | 199 | 1920 | 3224 | AB303566 | Virosaurus | DNA library |
| 25163 | 3/5/2015 | 446_D | L004-r75 | TTMDV | Anelloviridae | 81.62 | 5 | 343 | 2602 | 3188 | KF545587 | Virosaurus | DNA library |
| 25164 | 3/5/2015 | 447_D | L005-r75 | TTMDV | Anelloviridae | 80.14 | 2 | 60 | 2034 | 2538 | KP343822 | Virosaurus | DNA library |
| 25169 | 3/6/2015 | 448_D | L006-r75 | TTMDV | Anelloviridae | 26.64 | 5 | 197 | 859 | 3224 | AB303558 | Virosaurus | DNA library |
| 25174 | 3/6/2015 | 449_D | L005-r73 | TTMDV | Anelloviridae | 33.91 | 3 | 54 | 903 | 2663 | KP343827 | Virosaurus | DNA library |
| 25179 | 3/9/2015 | 450_D | L006-r73 | TTMDV | Anelloviridae | 91.02 | 14 | 432 | 2048 | 2250 | NC_007013 | Virosaurus | DNA library |
| 25181 | 3/9/2015 | 451_D | L007-r75 | TTMDV | Anelloviridae | 31.39 | 5 | 74 | 836 | 2663 | KP343827 | Virosaurus | DNA library |
| 25194 | 3/12/2015 | 452_D | L001-r77 | TTMDV | Anelloviridae | 79.93 | 5 | 213 | 2548 | 3188 | KF545587 | Virosaurus | DNA library |
| 25195 | 3/12/2015 | 453_D | L002-r77 | TTMDV | Anelloviridae | 18.07 | 35 | 473 | 578 | 3198 | AB303554 | Virosaurus | DNA library |
| 25196 | 3/13/2015 | 454_D | L003-r77 | TTMDV | Anelloviridae | 44.15 | 29 | 597 | 1412 | 3198 | AB303554 | Virosaurus | DNA library |
| 25199 | 3/12/2015 | 455_D | L004-r77 | TTMDV | Anelloviridae | 37.08 | 2 | 19 | 941 | 2538 | KP343822 | Virosaurus | DNA library |
| 25203 | 3/17/2015 | 456_D | L005-r77 | TTMDV | Anelloviridae | 99.19 | 47 | 1467 | 3050 | 3075 | MN776349 | De novo | DNA library |
| 25204 | 3/17/2015 | 457_D | L001-r80 | TTMDV | Anelloviridae | 79.71 | 5 | 210 | 2541 | 3188 | KF545587 | Virosaurus | DNA library |
| 25207 | 3/17/2015 | 458_D | L002-r80 | TTMDV | Anelloviridae | 36.72 | 3 | 157 | 1169 | 3184 | AB303565 | Virosaurus | DNA library |
| 25212 | 3/18/2015 | 459_D | L001-r77 | TTMDV | Anelloviridae | 19.78 | 3 | 51 | 633 | 3201 | AB303556 | Virosaurus | DNA library |
| 25213 | 3/19/2015 | 460_D | L002-r77 | TTMDV | Anelloviridae | 34.54 | 41 | 787 | 1124 | 3254 | NC_014093 | Virosaurus | DNA library |
| 25216 | 3/19/2015 | 461_D | L003-r77 | TTMDV | Anelloviridae | 98.62 | 355 | 26510 | 2992 | 3034 | MN778311 | De novo | DNA library |
| 25217 | 3/19/2015 | 462_D | L004-r77 | TTMDV | Anelloviridae | 17.1 | 8 | 91 | 547 | 3198 | AB303554 | Virosaurus | DNA library |
| 25222 | 3/20/2015 | 464_D | L001-r80 | TTMDV | Anelloviridae | 95.46 | 21 | 708 | 2984 | 3126 | MN776320 | De novo | DNA library |
| 25223 | 3/23/2015 | 771_D | L6-r113 | TTMDV | Anelloviridae | 30.18 | 2 | 1116 | 962 | 3188 | KF545587 | Virosaurus | DNA library |
| 25233 | 3/24/2015 | 465_D | L002-r80 | TTMDV | Anelloviridae | 30.62 | 1 | 21 | 976 | 3188 | KF545587 | Virosaurus | DNA library |
| 25247 | 3/26/2015 | 468_D | L003-r77 | TTMDV | Anelloviridae | 17.7 | 41 | 392 | 566 | 3198 | AB303554 | Virosaurus | DNA library |
| 25253 | 3/27/2015 | 469_D | L004-r77 | TTMDV | Anelloviridae | 90.74 | 31 | 807 | 2303 | 2538 | KP343822 | Virosaurus | DNA library |
| 25258 | 3/30/2015 | 470_D | L005-r77 | TTMDV | Anelloviridae | 94.05 | 1295 | 30160 | 2387 | 2538 | KP343822 | Virosaurus | DNA library |
| 25267 | 4/9/2015 | 471_D | L001-r80 | TTMDV | Anelloviridae | 97.75 | 63 | 1942 | 2996 | 3065 | MN774936 | De novo | DNA library |
| 25273 | 4/9/2015 | 472_D | L002-r80 | TTMDV | Anelloviridae | 46.44 | 3 | 91 | 1485 | 3198 | AB303554 | Virosaurus | DNA library |
| 25276 | 4/9/2015 | 473_D | L007-r75 | TTMDV | Anelloviridae | 39.8 | 11 | 257 | 1292 | 3246 | NC_009225 | Virosaurus | DNA library |
| 25282 | 4/10/2015 | 474_D | L001-r77 | TTMDV | Anelloviridae | 86.13 | 26 | 639 | 2236 | 2596 | MN780159 | De novo | DNA library |
| 25283 | 4/10/2015 | 475_D | L003-r77 | TTMDV | Anelloviridae | 100 | 577 | 17618 | 2687 | 2687 | KP343821 | Virosaurus | DNA library |
| 25286 | 4/10/2015 | 476_D | L004-r77 | TTMDV | Anelloviridae | 64.01 | 9 | 829 | 2047 | 3198 | AB303554 | Virosaurus | DNA library |
| 25289 | 4/10/2015 | 477_D | L005-r77 | TTMDV | Anelloviridae | 99.15 | 13 | 2972 | 3017 | 3043 | MN780398 | De novo | DNA library |
| 25299 | 4/13/2015 | 478_D | L001-r80 | TTMDV | Anelloviridae | 97.96 | 166 | 6922 | 2204 | 2250 | NC_007013 | Virosaurus | DNA library |
| 25300 | 4/13/2015 | 479_D | L002-r80 | TTMDV | Anelloviridae | 72.84 | 4 | 381 | 2322 | 3188 | KF545587 | Virosaurus | DNA library |
| 25302 | 4/16/2015 | 480_D | L007-r75 | TTMDV | Anelloviridae | 33.08 | 4 | 324 | 1058 | 3198 | AB303554 | Virosaurus | DNA library |
| 25317 | 4/15/2015 | 481_D | L001-r77 | TTMDV | Anelloviridae | 34.48 | 13 | 405 | 1122 | 3254 | NC_014093 | Virosaurus | DNA library |
| 25323 | 4/15/2015 | 482_D | L002-r77 | TTMDV | Anelloviridae | 17.48 | 11 | 110 | 559 | 3198 | AB303554 | Virosaurus | DNA library |
| 25325 | 4/15/2015 | 483_D | L004-r77 | TTMDV | Anelloviridae | 15.31 | 21 | 152 | 488 | 3188 | AB303562 | Virosaurus | DNA library |
| 25329 | 4/16/2015 | 484_D | L005-r77 | TTMDV | Anelloviridae | 89.95 | 4 | 1338 | 2283 | 2538 | KP343822 | Virosaurus | DNA library |
| 25334 | 4/16/2015 | 485_D | L001-r80 | TTMDV | Anelloviridae | 100 | 8 | 298 | 2687 | 2687 | KP343821 | Virosaurus | DNA library |
| 25342 | 4/16/2015 | 772_D | L7-r113 | TTMDV | Anelloviridae | 20.2 | 8 | 137 | 646 | 3198 | AB303554 | Virosaurus | DNA library |
| 25343 | 4/17/2015 | 486_D | L002-r80 | TTMDV | Anelloviridae | 49.57 | 2 | 32 | 1389 | 2802 | KF545588 | Virosaurus | DNA library |
| 25345 | 4/17/2015 | 487_D | L007-r75 | TTMDV | Anelloviridae | 13.73 | 4 | 23 | 439 | 3198 | AB303554 | Virosaurus | DNA library |
| 25353 | 4/21/2015 | 488_D | L001-r77 | TTMDV | Anelloviridae | 27.45 | 16 | 356 | 885 | 3224 | AB303558 | Virosaurus | DNA library |
| 25355 | 4/21/2015 | 489_D | L002-r77 | TTMDV | Anelloviridae | 14.17 | 3 | 24 | 453 | 3198 | AB303554 | Virosaurus | DNA library |
| 25361 | 4/22/2015 | 490_D | L003-r77 | TTMDV | Anelloviridae | 93.6 | 9 | 262 | 2106 | 2250 | NC_007013 | Virosaurus | DNA library |
| 25365 | 4/22/2015 | 491_D | L005-r77 | TTMDV | Anelloviridae | 99.11 | 24 | 52299 | 3022 | 3049 | MN778394 | De novo | DNA library |
| 25365 | 4/22/2015 | 491_R | L4-r713 | TTMDV | Anelloviridae | 28.53 | 9 | 114 | 765 | 2681 | MN777049 | De novo | RNA library |
| 25368 | 4/22/2015 | 492_D | L001-r80 | TTMDV | Anelloviridae | 52.43 | 2 | 71 | 1620 | 3090 | MN778050 | De novo | DNA library |
| 25374 | 4/23/2015 | 493_D | L002-r80 | TTMDV | Anelloviridae | 15.04 | 4 | 24 | 481 | 3198 | AB303554 | Virosaurus | DNA library |
| 25376 | 4/24/2015 | 798_D | L5-r113 | TTMDV | Anelloviridae | 49.5 | 1 | 27 | 1387 | 2802 | KF545588 | Virosaurus | DNA library |
| 25380 | 4/27/2015 | 494_D | L007-r75 | TTMDV | Anelloviridae | 16.84 | 3 | 43 | 543 | 3224 | AB303558 | Virosaurus | DNA library |
| 25385 | 4/28/2015 | 495_D | L001-r77 | TTMDV | Anelloviridae | 26.13 | 2 | 51 | 833 | 3188 | AB303559 | Virosaurus | DNA library |
| 25396 | 4/28/2015 | 496_D | L003-r80 | TTMDV | Anelloviridae | 17.32 | 19 | 281 | 554 | 3198 | AB303554 | Virosaurus | DNA library |
| 25409 | 4/30/2015 | 498_D | L005-r80 | TTMDV | Anelloviridae | 30.52 | 8 | 679 | 973 | 3188 | KF545587 | Virosaurus | DNA library |
| 25413 | 5/5/2015 | 499_D | L006-r80 | TTMDV | Anelloviridae | 25.35 | 1 | 44 | 808 | 3188 | AB303559 | Virosaurus | DNA library |
| 25423 | 5/6/2015 | 500_R | L7-r713 | TTMDV | Anelloviridae | 16.26 | 4 | 18 | 436 | 2681 | MN777049 | De novo | RNA library |
| 25423 | 5/6/2015 | 500_D | L5-r82 | TTMDV | Anelloviridae | 94.29 | 1837 | 56062 | 2393 | 2538 | KP343822 | Virosaurus | DNA library |
| 25424 | 5/6/2015 | 501_D | L6-r82 | TTMDV | Anelloviridae | 21.65 | 1 | 11 | 693 | 3201 | AB303556 | Virosaurus | DNA library |
| 25425 | 5/6/2015 | 502_D | L005-r84 | TTMDV | Anelloviridae | 51.53 | 4 | 384 | 1648 | 3198 | AB303554 | Virosaurus | DNA library |
| 25426 | 5/6/2015 | 503_D | L006-r84 | TTMDV | Anelloviridae | 51.07 | 1 | 138 | 1149 | 2250 | NC_007013 | Virosaurus | DNA library |
| 25432 | 5/6/2015 | 504_D | L004-r80 | TTMDV | Anelloviridae | 99.81 | 14 | 448 | 2682 | 2687 | KP343821 | Virosaurus | DNA library |
| 25434 | 5/7/2015 | 505_D | L005-r80 | TTMDV | Anelloviridae | 39.84 | 2 | 159 | 1274 | 3198 | AB303554 | Virosaurus | DNA library |
| 25435 | 5/7/2015 | 506_D | L006-r80 | TTMDV | Anelloviridae | 15.48 | 7 | 48 | 495 | 3198 | AB303554 | Virosaurus | DNA library |
| 25438 | 5/7/2015 | 507_D | L5-r82 | TTMDV | Anelloviridae | 98.4 | 541 | 15706 | 3016 | 3065 | MN774936 | De novo | DNA library |
| 25441 | 5/7/2015 | 589_D | L2-r89 | TTMDV | Anelloviridae | 33.37 | 4 | 118 | 847 | 2538 | KP343822 | Virosaurus | DNA library |
| 25446 | 5/8/2015 | 508_D | L6-r82 | TTMDV | Anelloviridae | 92.07 | 54 | 1751 | 2715 | 2949 | MN778074 | De novo | DNA library |
| 25449 | 5/8/2015 | 509_D | L005-r84 | TTMDV | Anelloviridae | 53.42 | 2 | 62 | 1703 | 3188 | AB303559 | Virosaurus | DNA library |
| 25459 | 5/12/2015 | 511_D | L003-r80 | TTMDV | Anelloviridae | 53.03 | 3 | 145 | 1696 | 3198 | AB303554 | Virosaurus | DNA library |
| 25463 | 5/12/2015 | 512_D | L005-r80 | TTMDV | Anelloviridae | 14.95 | 12 | 152 | 478 | 3198 | AB303554 | Virosaurus | DNA library |
| 25467 | 5/12/2015 | 513_D | L006-r80 | TTMDV | Anelloviridae | 39.52 | 10 | 215 | 1255 | 3176 | AB303560 | Virosaurus | DNA library |
| 25468 | 5/12/2015 | 514_D | L5-r82 | TTMDV | Anelloviridae | 89.01 | 22 | 673 | 2259 | 2538 | KP343822 | Virosaurus | DNA library |
| 25470 | 5/12/2015 | 515_D | L6-r82 | TTMDV | Anelloviridae | 95.55 | 328 | 9356 | 2898 | 3033 | MN775002 | De novo | DNA library |
| 25472 | 5/11/2015 | 516_D | L005-r84 | TTMDV | Anelloviridae | 96.77 | 87 | 2859 | 3082 | 3185 | MN774949 | De novo | DNA library |
| 25482 | 5/14/2015 | 517_D | L006-r84 | TTMDV | Anelloviridae | 48.71 | 10 | 749 | 1553 | 3188 | AB303562 | Virosaurus | DNA library |
| 25488 | 5/15/2015 | 519_D | L004-r80 | TTMDV | Anelloviridae | 16.2 | 9 | 55 | 518 | 3198 | AB303554 | Virosaurus | DNA library |
| 25491 | 5/15/2015 | 520_D | L006-r80 | TTMDV | Anelloviridae | 34.8 | 4 | 200 | 1122 | 3224 | AB303558 | Virosaurus | DNA library |
| 25492 | 5/15/2015 | 521_D | L5-r82 | TTMDV | Anelloviridae | 100 | 363 | 12078 | 3033 | 3033 | MN774934 | De novo | DNA library |
| 25512 | 5/19/2015 | 591_D | L4-r89 | TTMDV | Anelloviridae | 21.05 | 3 | 91 | 671 | 3188 | AB303562 | Virosaurus | DNA library |
| 25515 | 5/19/2015 | 522_D | L6-r82 | TTMDV | Anelloviridae | 15.7 | 11 | 75 | 502 | 3198 | AB303554 | Virosaurus | DNA library |
| 25517 | 5/19/2015 | 523_D | L005-r84 | TTMDV | Anelloviridae | 66.69 | 3 | 686 | 2126 | 3188 | KF545587 | Virosaurus | DNA library |
| 25528 | 5/20/2015 | 524_D | L006-r84 | TTMDV | Anelloviridae | 10.04 | 2 | 14 | 321 | 3198 | AB303554 | Virosaurus | DNA library |
| 25532 | 5/20/2015 | 525_D | L003-r80 | TTMDV | Anelloviridae | 35.07 | 14 | 260 | 1118 | 3188 | AB303562 | Virosaurus | DNA library |
| 25535 | 5/21/2015 | 526_D | L004-r80 | TTMDV | Anelloviridae | 40.62 | 14 | 193 | 1290 | 3176 | AB303560 | Virosaurus | DNA library |
| 25545 | 5/21/2015 | 773_D | L8-r113 | TTMDV | Anelloviridae | 16.23 | 29 | 180 | 519 | 3198 | AB303554 | Virosaurus | DNA library |
| 25550 | 5/21/2015 | 527_D | L005-r80 | TTMDV | Anelloviridae | 16.02 | 1 | 9 | 510 | 3184 | AB303565 | Virosaurus | DNA library |
| 25551 | 5/22/2015 | 528_D | L5-r82 | TTMDV | Anelloviridae | 99.81 | 1721 | 65083 | 3117 | 3123 | MN780421 | De novo | DNA library |
| 25556 | 5/22/2015 | 592_D | L1-r92 | TTMDV | Anelloviridae | 95.99 | 10 | 409 | 2944 | 3067 | MN780419 | De novo | DNA library |
| 25558 | 5/22/2015 | 529_D | L6-r82 | TTMDV | Anelloviridae | 15.42 | 9 | 44 | 493 | 3198 | AB303554 | Virosaurus | DNA library |
| 25560 | 5/25/2015 | 861_D | L4-r132 | TTMDV | Anelloviridae | 100 | 427 | 14027 | 2687 | 2687 | KP343821 | Virosaurus | DNA library |
| 25563 | 5/25/2015 | 530_D | L005-r84 | TTMDV | Anelloviridae | 14.14 | 1 | 26 | 456 | 3224 | AB303558 | Virosaurus | DNA library |
| 25569 | 5/26/2015 | 531_D | L006-r84 | TTMDV | Anelloviridae | 27.3 | 2 | 47 | 867 | 3176 | AB303560 | Virosaurus | DNA library |
| 25572 | 5/26/2015 | 532_D | L003-r80 | TTMDV | Anelloviridae | 14.98 | 2 | 14 | 479 | 3198 | AB303554 | Virosaurus | DNA library |
| 25574 | 5/26/2015 | 533_D | L004-r80 | TTMDV | Anelloviridae | 36.3 | 33 | 351 | 917 | 2526 | MN777833 | De novo | DNA library |
| 25575 | 5/26/2015 | 534_D | L005-r80 | TTMDV | Anelloviridae | 21.21 | 8 | 653 | 679 | 3201 | AB303556 | Virosaurus | DNA library |
| 25581 | 5/27/2015 | 535_D | L006-r80 | TTMDV | Anelloviridae | 31.36 | 4 | 58 | 996 | 3176 | AB303560 | Virosaurus | DNA library |
| 25583 | 5/27/2015 | 536_D | L6-r82 | TTMDV | Anelloviridae | 33.09 | 11 | 159 | 1051 | 3176 | AB303560 | Virosaurus | DNA library |
| 25584 | 5/27/2015 | 537_D | L005-r84 | TTMDV | Anelloviridae | 27.58 | 8 | 81 | 876 | 3176 | AB303560 | Virosaurus | DNA library |
| 25586 | 5/27/2015 | 862_D | L5-r132 | TTMDV | Anelloviridae | 14.54 | 25 | 231 | 465 | 3199 | AB303553 | Virosaurus | DNA library |
| 25589 | 5/27/2015 | 538_D | L006-r84 | TTMDV | Anelloviridae | 28.43 | 3 | 31 | 903 | 3176 | AB303560 | Virosaurus | DNA library |
| 25592 | 5/27/2015 | 539_D | L003-r80 | TTMDV | Anelloviridae | 17.32 | 6 | 142 | 554 | 3198 | AB303554 | Virosaurus | DNA library |
| 25594 | 5/28/2015 | 540_D | L004-r80 | TTMDV | Anelloviridae | 17.01 | 2 | 16 | 544 | 3198 | AB303554 | Virosaurus | DNA library |
| 25596 | 5/28/2015 | 541_D | L1-r85 | TTMDV | Anelloviridae | 99.4 | 3745 | 114095 | 3160 | 3179 | MN774942 | De novo | DNA library |
| 25598 | 5/28/2015 | 542_D | L2-r85 | TTMDV | Anelloviridae | 16.7 | 17 | 111 | 534 | 3198 | AB303554 | Virosaurus | DNA library |
| 25599 | 5/28/2015 | 543_D | L3-r85 | TTMDV | Anelloviridae | 59.67 | 1 | 25 | 1672 | 2802 | KF545588 | Virosaurus | DNA library |
| 25600 | 5/28/2015 | 544_D | L4-r85 | TTMDV | Anelloviridae | 11.01 | 2 | 10 | 352 | 3198 | AB303554 | Virosaurus | DNA library |
| 25601 | 5/28/2015 | 545_D | L5-r85 | TTMDV | Anelloviridae | 99.44 | 2758 | 75178 | 2851 | 2867 | MN780003 | De novo | DNA library |
| 25602 | 5/28/2015 | 546_D | L6-r85 | TTMDV | Anelloviridae | 14.63 | 9 | 52 | 468 | 3198 | AB303554 | Virosaurus | DNA library |
| 25603 | 5/29/2015 | 547_D | L7-r85 | TTMDV | Anelloviridae | 13.1 | 1 | 31 | 419 | 3198 | AB303554 | Virosaurus | DNA library |
| 25604 | 5/29/2015 | 548_D | L8-r85 | TTMDV | Anelloviridae | 42 | 14 | 214 | 1334 | 3176 | AB303560 | Virosaurus | DNA library |
| 25606 | 5/29/2015 | 549_D | L2-r85 | TTMDV | Anelloviridae | 17.32 | 42 | 343 | 554 | 3198 | AB303554 | Virosaurus | DNA library |
| 25607 | 5/29/2015 | 550_D | L3-r85 | TTMDV | Anelloviridae | 47.2 | 2 | 60 | 1062 | 2250 | NC_007013 | Virosaurus | DNA library |
| 25608 | 5/29/2015 | 551_D | L4-r85 | TTMDV | Anelloviridae | 85.34 | 3 | 196 | 2585 | 3029 | MN776513 | De novo | DNA library |
| 25610 | 6/1/2015 | 552_R | L4-r717 | TTMDV | Anelloviridae | 10.85 | 7 | 18 | 334 | 3078 | MN779750 | De novo | RNA library |
| 25610 | 6/1/2015 | 552_D | L5-r85 | TTMDV | Anelloviridae | 94.25 | 2837 | 63442 | 2392 | 2538 | KP343822 | Virosaurus | DNA library |
| 25611 | 6/1/2015 | 553_D | L6-r85 | TTMDV | Anelloviridae | 10.94 | 2 | 38 | 350 | 3199 | AB303553 | Virosaurus | DNA library |
| 25613 | 6/1/2015 | 554_R | L5-r717 | TTMDV | Anelloviridae | 16.49 | 2 | 14 | 439 | 2663 | MN777060 | De novo | RNA library |
| 25613 | 6/1/2015 | 554_D | L7-r85 | TTMDV | Anelloviridae | 96.27 | 21 | 1538 | 2166 | 2250 | NC_007013 | Virosaurus | DNA library |
| 25617 | 6/2/2015 | 555_D | L8-r85 | TTMDV | Anelloviridae | 20.29 | 6 | 167 | 654 | 3224 | AB303558 | Virosaurus | DNA library |
| 25619 | 6/2/2015 | 556_D | L1-r85 | TTMDV | Anelloviridae | 94.29 | 2235 | 56336 | 2393 | 2538 | KP343822 | Virosaurus | DNA library |
| 25621 | 6/2/2015 | 863_D | L6-r132 | TTMDV | Anelloviridae | 52.75 | 21 | 1074 | 1687 | 3198 | AB303554 | Virosaurus | DNA library |
| 25625 | 6/2/2015 | 557_D | L3-r85 | TTMDV | Anelloviridae | 17.9 | 16 | 205 | 577 | 3224 | AB303558 | Virosaurus | DNA library |
| 25626 | 6/2/2015 | 594_D | L8-r88 | TTMDV | Anelloviridae | 98.64 | 766 | 23554 | 3048 | 3090 | MN780388 | De novo | DNA library |
| 25630 | 6/2/2015 | 558_D | L4-r85 | TTMDV | Anelloviridae | 18.19 | 24 | 601 | 582 | 3199 | AB303553 | Virosaurus | DNA library |
| 25632 | 6/3/2015 | 559_D | L5-r85 | TTMDV | Anelloviridae | 97.74 | 84 | 2524 | 3026 | 3096 | MN775978 | De novo | DNA library |
| 25634 | 6/3/2015 | 560_D | L6-r85 | TTMDV | Anelloviridae | 14.42 | 3 | 43 | 461 | 3198 | AB303554 | Virosaurus | DNA library |
| 25635 | 6/3/2015 | 561_D | L7-r85 | TTMDV | Anelloviridae | 35.07 | 4 | 75 | 1141 | 3254 | NC_014093 | Virosaurus | DNA library |
| 25636 | 6/3/2015 | 562_R | L7-r717 | TTMDV | Anelloviridae | 32.93 | 8 | 74 | 975 | 2961 | MN774973 | De novo | RNA library |
| 25636 | 6/3/2015 | 562_D | L8-r85 | TTMDV | Anelloviridae | 18.73 | 10 | 172 | 608 | 3246 | NC_009225 | Virosaurus | DNA library |
| 25639 | 6/3/2015 | 563_D | L1-r85 | TTMDV | Anelloviridae | 97.55 | 14 | 5588 | 2951 | 3025 | MN779910 | De novo | DNA library |
| 25643 | 6/4/2015 | 799_D | L6-r113 | TTMDV | Anelloviridae | 61.54 | 4 | 801 | 1968 | 3198 | AB303554 | Virosaurus | DNA library |
| 25644 | 6/4/2015 | 564_D | L2-r85 | TTMDV | Anelloviridae | 12.13 | 1 | 8 | 388 | 3198 | AB303554 | Virosaurus | DNA library |
| 25650 | 6/4/2015 | 800_D | L7-r113 | TTMDV | Anelloviridae | 60.19 | 2 | 75 | 1919 | 3188 | KF545587 | Virosaurus | DNA library |
| 25652 | 6/4/2015 | 801_D | L8-r113 | TTMDV | Anelloviridae | 17.03 | 1 | 21 | 549 | 3224 | AB303558 | Virosaurus | DNA library |
| 25653 | 6/4/2015 | 565_D | L4-r85 | TTMDV | Anelloviridae | 23.02 | 11 | 113 | 738 | 3206 | AB303555 | Virosaurus | DNA library |
| 25654 | 6/5/2015 | 566_R | L8-r717 | TTMDV | Anelloviridae | 11.42 | 1 | 5 | 300 | 2627 | MN776101 | De novo | RNA library |
| 25654 | 6/5/2015 | 566_D | L5-r85 | TTMDV | Anelloviridae | 84.51 | 35 | 1413 | 2368 | 2802 | KF545588 | Virosaurus | DNA library |
| 25656 | 6/5/2015 | 567_D | L6-r85 | TTMDV | Anelloviridae | 24.43 | 3 | 33 | 776 | 3176 | AB303560 | Virosaurus | DNA library |
| 25657 | 6/5/2015 | 568_D | L7-r85 | TTMDV | Anelloviridae | 90 | 203 | 5424 | 2501 | 2779 | MN779698 | De novo | DNA library |
| 25671 | 6/8/2015 | 569_D | L8-r85 | TTMDV | Anelloviridae | 31.33 | 3 | 176 | 1010 | 3224 | AB303566 | Virosaurus | DNA library |
| 25672 | 6/8/2015 | 570_D | L1-r85 | TTMDV | Anelloviridae | 99.15 | 359 | 11004 | 3039 | 3065 | MN776459 | De novo | DNA library |
| 25675 | 6/9/2015 | 571_D | L2-r85 | TTMDV | Anelloviridae | 18.18 | 2 | 15 | 582 | 3201 | AB303556 | Virosaurus | DNA library |
| 25676 | 6/9/2015 | 572_D | L3-r85 | TTMDV | Anelloviridae | 35.36 | 4 | 78 | 1123 | 3176 | AB303560 | Virosaurus | DNA library |
| 25678 | 6/9/2015 | 573_D | L5-r85 | TTMDV | Anelloviridae | 100 | 45988 | 1382138 | 3248 | 3248 | MN774939 | De novo | DNA library |
| 25679 | 6/9/2015 | 574_D | L6-r85 | TTMDV | Anelloviridae | 9.66 | 4 | 18 | 309 | 3198 | AB303554 | Virosaurus | DNA library |
| 25680 | 6/9/2015 | 575_D | L7-r85 | TTMDV | Anelloviridae | 76.13 | 56 | 1764 | 2213 | 2907 | MN774998 | De novo | DNA library |
| 25681 | 6/9/2015 | 864_D | L8-r127 | TTMDV | Anelloviridae | 10.32 | 4 | 14 | 330 | 3198 | AB303554 | Virosaurus | DNA library |
| 25685 | 6/9/2015 | 577_D | L1-r85 | TTMDV | Anelloviridae | 99.29 | 524 | 41284 | 3197 | 3220 | MN774941 | De novo | DNA library |
| 25689 | 6/10/2015 | 578_D | L2-r85 | TTMDV | Anelloviridae | 39.89 | 12 | 303 | 1270 | 3184 | AB303565 | Virosaurus | DNA library |
| 25690 | 6/10/2015 | 579_D | L3-r85 | TTMDV | Anelloviridae | 12.04 | 1 | 4 | 306 | 2542 | KP343826 | Virosaurus | DNA library |
| 25691 | 6/10/2015 | 580_D | L4-r85 | TTMDV | Anelloviridae | 30.54 | 2 | 30 | 970 | 3176 | AB303560 | Virosaurus | DNA library |
| 25692 | 6/10/2015 | 581_R | L004-r722 | TTMDV | Anelloviridae | 23.06 | 2 | 16 | 596 | 2585 | MN775865 | De novo | RNA library |
| 25692 | 6/10/2015 | 581_D | L6-r85 | TTMDV | Anelloviridae | 23.52 | 4 | 57 | 753 | 3201 | AB303556 | Virosaurus | DNA library |
| 25694 | 6/11/2015 | 582_D | L7-r85 | TTMDV | Anelloviridae | 34.95 | 10 | 151 | 1110 | 3176 | AB303560 | Virosaurus | DNA library |
| 25700 | 6/11/2015 | 583_D | L8-r85 | TTMDV | Anelloviridae | 35.33 | 2 | 39 | 1139 | 3224 | AB303566 | Virosaurus | DNA library |
| 25710 | 6/15/2015 | 584_R | L004-r722 | TTMDV | Anelloviridae | 50.65 | 8 | 182 | 1518 | 2997 | MN774970 | De novo | RNA library |
| 25710 | 6/15/2015 | 584_D | L1-r85 | TTMDV | Anelloviridae | 40.3 | 23 | 642 | 1280 | 3176 | AB303560 | Virosaurus | DNA library |
| 25715 | 6/15/2015 | 585_D | L2-r85 | TTMDV | Anelloviridae | 41.66 | 3 | 53 | 1343 | 3224 | AB303566 | Virosaurus | DNA library |
| 25718 | 6/15/2015 | 681_D | L2-r93 | TTMDV | Anelloviridae | 89.32 | 35 | 962 | 2267 | 2538 | KP343822 | Virosaurus | DNA library |
| 25721 | 6/16/2015 | 682_D | L3-r93 | TTMDV | Anelloviridae | 17.14 | 29 | 355 | 548 | 3198 | AB303554 | Virosaurus | DNA library |
| 25722 | 6/16/2015 | 683_D | L4-r93 | TTMDV | Anelloviridae | 9.69 | 3 | 14 | 310 | 3198 | AB303554 | Virosaurus | DNA library |
| 25723 | 6/16/2015 | 684_D | L4-r92 | TTMDV | Anelloviridae | 57.1 | 8 | 978 | 1826 | 3198 | AB303554 | Virosaurus | DNA library |
| 25729 | 6/17/2015 | 685_D | L5-r92 | TTMDV | Anelloviridae | 12.73 | 2 | 13 | 407 | 3198 | AB303554 | Virosaurus | DNA library |
| 25730 | 6/17/2015 | 686_R | L3-r734 | TTMDV | Anelloviridae | 22.69 | 3 | 25 | 606 | 2671 | MN779999 | De novo | RNA library |
| 25730 | 6/17/2015 | 686_D | L6-r92 | TTMDV | Anelloviridae | 39.1 | 12 | 714 | 1245 | 3184 | AB303565 | Virosaurus | DNA library |
| 25731 | 6/17/2015 | 687_R | L3-r734 | TTMDV | Anelloviridae | 12.04 | 2 | 6 | 376 | 3123 | MN780421 | De novo | RNA library |
| 25731 | 6/17/2015 | 687_D | L1-r93 | TTMDV | Anelloviridae | 81.02 | 5 | 470 | 2583 | 3188 | KF545587 | Virosaurus | DNA library |
| 25733 | 6/18/2015 | 865_D | L1-r132 | TTMDV | Anelloviridae | 72.2 | 19 | 1676 | 2309 | 3198 | AB303554 | Virosaurus | DNA library |
| 25734 | 6/18/2015 | 688_D | L2-r93 | TTMDV | Anelloviridae | 91.15 | 31 | 759 | 2492 | 2734 | MN777626 | De novo | DNA library |
| 25736 | 6/18/2015 | 866_D | L2-r132 | TTMDV | Anelloviridae | 25.03 | 13 | 637 | 807 | 3224 | AB303558 | Virosaurus | DNA library |
| 25740 | 6/18/2015 | 689_D | L3-r93 | TTMDV | Anelloviridae | 87.95 | 109 | 3201 | 2642 | 3004 | MN779243 | De novo | DNA library |
| 25742 | 6/19/2015 | 690_D | L4-r93 | TTMDV | Anelloviridae | 37.06 | 8 | 183 | 1180 | 3184 | AB303565 | Virosaurus | DNA library |
| 25746 | 6/19/2015 | 691_D | L3-r92 | TTMDV | Anelloviridae | 21.02 | 3 | 62 | 670 | 3188 | AB303562 | Virosaurus | DNA library |
| 25747 | 6/22/2015 | 692_D | L5-r92 | TTMDV | Anelloviridae | 11.32 | 1 | 5 | 362 | 3198 | AB303554 | Virosaurus | DNA library |
| 25756 | 6/22/2015 | 599_D | L1-r92 | TTMDV | Anelloviridae | 72.34 | 3 | 74 | 2027 | 2802 | KF545588 | Virosaurus | DNA library |
| 25757 | 6/22/2015 | 597_D | L3-r89 | TTMDV | Anelloviridae | 99.71 | 70 | 2003 | 3045 | 3054 | MN780380 | De novo | DNA library |
| 25758 | 6/23/2015 | 694_D | L1-r93 | TTMDV | Anelloviridae | 34.3 | 36 | 585 | 1116 | 3254 | NC_014093 | Virosaurus | DNA library |
| 25761 | 6/23/2015 | 601_D | L7-r88 | TTMDV | Anelloviridae | 16.79 | 14 | 154 | 537 | 3198 | AB303554 | Virosaurus | DNA library |
| 25762 | 6/23/2015 | 600_D | L2-r92 | TTMDV | Anelloviridae | 94.17 | 438 | 13018 | 2876 | 3054 | MN776246 | De novo | DNA library |
| 25764 | 6/23/2015 | 695_D | L2-r93 | TTMDV | Anelloviridae | 23.92 | 2 | 28 | 765 | 3198 | AB303554 | Virosaurus | DNA library |
| 25766 | 6/24/2015 | 696_D | L3-r93 | TTMDV | Anelloviridae | 17.32 | 39 | 331 | 554 | 3198 | AB303554 | Virosaurus | DNA library |
| 25767 | 6/24/2015 | 603_D | L2-r89 | TTMDV | Anelloviridae | 25.79 | 2 | 60 | 693 | 2687 | KP343821 | Virosaurus | DNA library |
| 25768 | 6/24/2015 | 605_D | L4-r89 | TTMDV | Anelloviridae | 38.18 | 3 | 61 | 1224 | 3206 | AB303555 | Virosaurus | DNA library |
| 25772 | 6/24/2015 | 697_D | L4-r93 | TTMDV | Anelloviridae | 24.25 | 2 | 30 | 773 | 3188 | AB303562 | Virosaurus | DNA library |
| 25773 | 6/24/2015 | 698_D | L3-r92 | TTMDV | Anelloviridae | 39.52 | 3 | 74 | 1260 | 3188 | AB303562 | Virosaurus | DNA library |
| 25774 | 6/24/2015 | 606_D | L1-r92 | TTMDV | Anelloviridae | 45.98 | 3 | 99 | 1167 | 2538 | KP343822 | Virosaurus | DNA library |
| 25777 | 6/24/2015 | 604_R | L004-r723 | TTMDV | Anelloviridae | 18.56 | 3 | 26 | 563 | 3033 | MN774984 | De novo | RNA library |
| 25777 | 6/24/2015 | 604_D | L3-r89 | TTMDV | Anelloviridae | 40.08 | 10 | 1179 | 1276 | 3184 | AB303565 | Virosaurus | DNA library |
| 25778 | 6/25/2015 | 699_D | L4-r92 | TTMDV | Anelloviridae | 32.62 | 4 | 56 | 1036 | 3176 | AB303560 | Virosaurus | DNA library |
| 25779 | 6/25/2015 | 700_D | L6-r92 | TTMDV | Anelloviridae | 96.91 | 31 | 1313 | 2604 | 2687 | KP343821 | Virosaurus | DNA library |
| 25783 | 6/25/2015 | 607_D | L2-r92 | TTMDV | Anelloviridae | 98.58 | 7 | 2583 | 2990 | 3033 | MN774984 | De novo | DNA library |
| 25784 | 6/25/2015 | 701_D | L1-r93 | TTMDV | Anelloviridae | 18.39 | 30 | 378 | 588 | 3198 | AB303554 | Virosaurus | DNA library |
| 25786 | 6/24/2015 | 702_D | L2-r93 | TTMDV | Anelloviridae | 22.81 | 5 | 49 | 579 | 2538 | KP343822 | Virosaurus | DNA library |
| 25787 | 6/25/2015 | 703_D | L3-r93 | TTMDV | Anelloviridae | 35.95 | 1 | 19 | 966 | 2687 | KP343821 | Virosaurus | DNA library |
| 25788 | 6/25/2015 | 704_D | L4-r93 | TTMDV | Anelloviridae | 77.6 | 4 | 271 | 2474 | 3188 | KF545587 | Virosaurus | DNA library |
| 25789 | 6/25/2015 | 705_D | L3-r92 | TTMDV | Anelloviridae | 82.26 | 4 | 142 | 2305 | 2802 | KF545588 | Virosaurus | DNA library |
| 25790 | 6/26/2015 | 706_D | L4-r92 | TTMDV | Anelloviridae | 44.47 | 2 | 45 | 1246 | 2802 | KF545588 | Virosaurus | DNA library |
| 25792 | 6/26/2015 | 707_D | L5-r92 | TTMDV | Anelloviridae | 44.82 | 3 | 75 | 1445 | 3224 | AB303566 | Virosaurus | DNA library |
| 25798 | 6/26/2015 | 709_D | L2-r93 | TTMDV | Anelloviridae | 28.71 | 4 | 93 | 919 | 3201 | AB303563 | Virosaurus | DNA library |
| 25799 | 6/29/2015 | 609_D | L8-r88 | TTMDV | Anelloviridae | 98.96 | 102 | 12175 | 3042 | 3074 | MN780383 | De novo | DNA library |
| 25801 | 6/29/2015 | 608_D | L7-r88 | TTMDV | Anelloviridae | 29.78 | 2 | 27 | 960 | 3224 | AB303566 | Virosaurus | DNA library |
| 25803 | 6/29/2015 | 710_D | L3-r93 | TTMDV | Anelloviridae | 91.69 | 21 | 672 | 2569 | 2802 | KF545588 | Virosaurus | DNA library |
| 25805 | 6/29/2015 | 610_D | L2-r89 | TTMDV | Anelloviridae | 21.95 | 408 | 3348 | 702 | 3198 | AB303554 | Virosaurus | DNA library |
| 25807 | 6/29/2015 | 711_D | L4-r93 | TTMDV | Anelloviridae | 17.29 | 6 | 38 | 553 | 3198 | AB303554 | Virosaurus | DNA library |
| 25812 | 6/30/2015 | 613_R | L006-r723 | TTMDV | Anelloviridae | 26.25 | 15 | 122 | 805 | 3067 | MN780419 | De novo | RNA library |
| 25812 | 6/30/2015 | 613_D | L1-r92 | TTMDV | Anelloviridae | 19.48 | 12 | 6631 | 623 | 3199 | AB303561 | Virosaurus | DNA library |
| 25813 | 6/30/2015 | 612_D | L4-r89 | TTMDV | Anelloviridae | 83.37 | 81 | 2066 | 2090 | 2507 | MN775040 | De novo | DNA library |
| 25814 | 6/30/2015 | 712_D | L3-r92 | TTMDV | Anelloviridae | 73.95 | 27 | 1643 | 2365 | 3198 | AB303554 | Virosaurus | DNA library |
| 25818 | 6/30/2015 | 713_D | L4-r92 | TTMDV | Anelloviridae | 33.44 | 7 | 104 | 1072 | 3206 | AB303555 | Virosaurus | DNA library |
| 25819 | 6/30/2015 | 714_D | L5-r92 | TTMDV | Anelloviridae | 13.37 | 7 | 59 | 431 | 3224 | AB303558 | Virosaurus | DNA library |
| 25823 | 7/1/2015 | 715_R | L2-r735 | TTMDV | Anelloviridae | 23.2 | 2 | 36 | 697 | 3004 | MN775492 | De novo | RNA library |
| 25823 | 7/1/2015 | 715_D | L6-r92 | TTMDV | Anelloviridae | 21.66 | 2 | 78 | 688 | 3176 | AB303560 | Virosaurus | DNA library |
| 25826 | 7/1/2015 | 716_D | L2-r93 | TTMDV | Anelloviridae | 100 | 22 | 823 | 2687 | 2687 | KP343821 | Virosaurus | DNA library |
| 25829 | 7/1/2015 | 717_D | L3-r93 | TTMDV | Anelloviridae | 21.41 | 3 | 24 | 680 | 3176 | AB303560 | Virosaurus | DNA library |
| 25830 | 7/1/2015 | 718_D | L4-r93 | TTMDV | Anelloviridae | 20.73 | 14 | 223 | 663 | 3198 | AB303554 | Virosaurus | DNA library |
| 25831 | 7/1/2015 | 719_D | L3-r92 | TTMDV | Anelloviridae | 20.04 | 1 | 18 | 641 | 3198 | AB303554 | Virosaurus | DNA library |
| 25837 | 7/2/2015 | 720_D | L4-r92 | TTMDV | Anelloviridae | 14.88 | 25 | 225 | 476 | 3199 | AB303553 | Virosaurus | DNA library |
| 25838 | 7/2/2015 | 721_R | L5-r735 | TTMDV | Anelloviridae | 49.47 | 5 | 131 | 1302 | 2632 | MN779353 | De novo | RNA library |
| 25838 | 7/2/2015 | 721_D | L5-r93 | TTMDV | Anelloviridae | 21.42 | 26 | 497 | 685 | 3198 | AB303554 | Virosaurus | DNA library |
| 25840 | 7/2/2015 | 722_D | L6-r93 | TTMDV | Anelloviridae | 70.93 | 2 | 35 | 1891 | 2666 | MN775390 | De novo | DNA library |
| 25841 | 7/2/2015 | 723_D | L7-r93 | TTMDV | Anelloviridae | 11.93 | 1 | 7 | 379 | 3176 | AB303560 | Virosaurus | DNA library |
| 25842 | 7/2/2015 | 724_D | L8-r93 | TTMDV | Anelloviridae | 16.14 | 7 | 79 | 516 | 3198 | AB303554 | Virosaurus | DNA library |
| 25843 | 7/3/2015 | 725_D | L4-r96 | TTMDV | Anelloviridae | 23.94 | 4 | 65 | 777 | 3246 | NC_009225 | Virosaurus | DNA library |
| 25850 | 7/3/2015 | 726_D | L5-r96 | TTMDV | Anelloviridae | 56.91 | 2 | 197 | 1820 | 3198 | AB303554 | Virosaurus | DNA library |
| 25851 | 7/6/2015 | 727_D | L6-r96 | TTMDV | Anelloviridae | 99.77 | 117 | 3206 | 2636 | 2642 | MN779956 | De novo | DNA library |
| 25865 | 7/8/2015 | 728_D | L7-r96 | TTMDV | Anelloviridae | 26.83 | 3 | 209 | 865 | 3224 | AB303558 | Virosaurus | DNA library |
| 25873 | 7/10/2015 | 618_R | L008-r723 | TTMDV | Anelloviridae | 16.41 | 5 | 24 | 448 | 2730 | MN777961 | De novo | RNA library |
| 25873 | 7/10/2015 | 618_D | L3-r89 | TTMDV | Anelloviridae | 17.57 | 16 | 387 | 562 | 3198 | AB303554 | Virosaurus | DNA library |
| 25874 | 7/9/2015 | 776_D | L4-r113 | TTMDV | Anelloviridae | 18.96 | 1 | 11 | 607 | 3201 | AB303556 | Virosaurus | DNA library |
| 25876 | 7/9/2015 | 729_D | L6-r93 | TTMDV | Anelloviridae | 10.01 | 4 | 15 | 320 | 3198 | AB303554 | Virosaurus | DNA library |
| 25880 | 7/9/2015 | 669_D | L3-r91 | TTMDV | Anelloviridae | 21.98 | 2 | 15 | 698 | 3176 | AB303560 | Virosaurus | DNA library |
| 25882 | 7/10/2015 | 730_D | L7-r93 | TTMDV | Anelloviridae | 28.18 | 4 | 42 | 895 | 3176 | AB303560 | Virosaurus | DNA library |
| 25883 | 7/10/2015 | 731_D | L8-r93 | TTMDV | Anelloviridae | 89.95 | 5 | 277 | 2417 | 2687 | KP343821 | Virosaurus | DNA library |
| 25885 | 7/13/2015 | 732_D | L4-r96 | TTMDV | Anelloviridae | 12.59 | 1 | 5 | 400 | 3176 | AB303560 | Virosaurus | DNA library |
| 25886 | 7/10/2015 | 733_D | L5-r96 | TTMDV | Anelloviridae | 88.01 | 131 | 3265 | 2371 | 2694 | MN780471 | De novo | DNA library |
| 25893 | 7/10/2015 | 734_D | L6-r96 | TTMDV | Anelloviridae | 91.25 | 3 | 120 | 2452 | 2687 | KP343821 | Virosaurus | DNA library |
| 25895 | 7/13/2015 | 735_D | L7-r96 | TTMDV | Anelloviridae | 9.86 | 1 | 4 | 320 | 3246 | NC_009225 | Virosaurus | DNA library |
| 25897 | 7/13/2015 | 736_D | L5-r93 | TTMDV | Anelloviridae | 14.25 | 3 | 33 | 456 | 3199 | AB303553 | Virosaurus | DNA library |
| 25898 | 7/13/2015 | 737_D | L7-r93 | TTMDV | Anelloviridae | 98.33 | 154 | 4723 | 2997 | 3048 | MN780010 | De novo | DNA library |
| 25900 | 7/13/2015 | 738_D | L8-r93 | TTMDV | Anelloviridae | 24.84 | 3 | 25 | 789 | 3176 | AB303560 | Virosaurus | DNA library |
| 25910 | 7/16/2015 | 777_D | L5-r113 | TTMDV | Anelloviridae | 35.18 | 6 | 144 | 1126 | 3201 | AB303563 | Virosaurus | DNA library |
| 25923 | 7/16/2015 | 867_D | L3-r132 | TTMDV | Anelloviridae | 21.71 | 15 | 759 | 700 | 3224 | AB303558 | Virosaurus | DNA library |
| 25930 | 7/21/2015 | 622_D | L7-r88 | TTMDV | Anelloviridae | 17.57 | 7 | 51 | 562 | 3198 | AB303554 | Virosaurus | DNA library |
| 25938 | 7/21/2015 | 739_D | L4-r96 | TTMDV | Anelloviridae | 27.52 | 4 | 39 | 874 | 3176 | AB303560 | Virosaurus | DNA library |
| 25939 | 7/21/2015 | 740_D | L5-r96 | TTMDV | Anelloviridae | 19.99 | 4 | 76 | 640 | 3201 | AB303563 | Virosaurus | DNA library |
| 25940 | 7/21/2015 | 741_D | L6-r96 | TTMDV | Anelloviridae | 94.79 | 3 | 122 | 2547 | 2687 | KP343821 | Virosaurus | DNA library |
| 25942 | 7/21/2015 | 868_D | L4-r132 | TTMDV | Anelloviridae | 53.47 | 5 | 3739 | 1710 | 3198 | AB303554 | Virosaurus | DNA library |
| 25946 | 7/23/2015 | 628_R | L2-r724 | TTMDV | Anelloviridae | 22.45 | 2 | 26 | 563 | 2508 | MN777023 | De novo | RNA library |
| 25946 | 7/23/2015 | 628_D | L2-r92 | TTMDV | Anelloviridae | 83.75 | 13 | 857 | 2670 | 3188 | KF545587 | Virosaurus | DNA library |
| 25947 | 7/23/2015 | 630_D | L8-r96 | TTMDV | Anelloviridae | 54.78 | 3 | 58 | 1766 | 3224 | AB303566 | Virosaurus | DNA library |
| 25954 | 7/23/2015 | 629_D | L7-r88 | TTMDV | Anelloviridae | 17.46 | 2 | 20 | 563 | 3224 | AB303558 | Virosaurus | DNA library |
| 25955 | 7/23/2015 | 742_D | L7-r96 | TTMDV | Anelloviridae | 12.82 | 3 | 31 | 410 | 3198 | AB303554 | Virosaurus | DNA library |
| 25965 | 7/28/2015 | 631_D | L1-r91 | TTMDV | Anelloviridae | 81.76 | 2 | 56 | 2075 | 2538 | KP343822 | Virosaurus | DNA library |
| 25969 | 7/27/2015 | 743_D | L5-r93 | TTMDV | Anelloviridae | 59.96 | 3 | 60 | 1584 | 2642 | MN778250 | De novo | DNA library |
| 25980 | 7/28/2015 | 744_D | L6-r93 | TTMDV | Anelloviridae | 17.48 | 9 | 64 | 559 | 3198 | AB303554 | Virosaurus | DNA library |
| 25982 | 7/28/2015 | 745_D | L8-r93 | TTMDV | Anelloviridae | 14.54 | 3 | 20 | 465 | 3198 | AB303554 | Virosaurus | DNA library |
| 25987 | 7/30/2015 | 746_D | L4-r96 | TTMDV | Anelloviridae | 12.83 | 2 | 11 | 409 | 3188 | AB303562 | Virosaurus | DNA library |
| 25993 | 7/30/2015 | 747_D | L5-r96 | TTMDV | Anelloviridae | 16.95 | 15 | 104 | 542 | 3198 | AB303554 | Virosaurus | DNA library |
| 25995 | 7/30/2015 | 748_D | L6-r96 | TTMDV | Anelloviridae | 100 | 118 | 3591 | 2687 | 2687 | KP343821 | Virosaurus | DNA library |
| 26003 | 7/31/2015 | 635_D | L5-r91 | TTMDV | Anelloviridae | 27.55 | 2 | 77 | 881 | 3198 | AB303554 | Virosaurus | DNA library |
| 26004 | 8/3/2015 | 634_D | L4-r91 | TTMDV | Anelloviridae | 70.14 | 13 | 1046 | 2243 | 3198 | AB303554 | Virosaurus | DNA library |
| 26015 | 8/4/2015 | 749_D | L7-r96 | TTMDV | Anelloviridae | 56.71 | 2 | 54 | 1276 | 2250 | NC_007013 | Virosaurus | DNA library |
| 26019 | 8/5/2015 | 636_D | L6-r91 | TTMDV | Anelloviridae | 19.94 | 1 | 11 | 643 | 3224 | AB303566 | Virosaurus | DNA library |
| 26027 | 8/5/2015 | 750_D | L5-r93 | TTMDV | Anelloviridae | 48.44 | 1 | 60 | 1090 | 2250 | NC_007013 | Virosaurus | DNA library |
| 26028 | 8/5/2015 | 751_D | L6-r93 | TTMDV | Anelloviridae | 89.19 | 10 | 284 | 2433 | 2728 | MN778694 | De novo | DNA library |
| 26033 | 8/6/2015 | 752_D | L7-r93 | TTMDV | Anelloviridae | 54.57 | 7 | 132 | 1689 | 3095 | MN780372 | De novo | DNA library |
| 26040 | 8/6/2015 | 638_D | L8-r91 | TTMDV | Anelloviridae | 18.2 | 10 | 72 | 582 | 3198 | AB303554 | Virosaurus | DNA library |
| 26042 | 8/6/2015 | 637_D | L7-r91 | TTMDV | Anelloviridae | 71.83 | 3 | 61 | 1823 | 2538 | KP343822 | Virosaurus | DNA library |
| 26046 | 8/7/2015 | 753_D | L4-r96 | TTMDV | Anelloviridae | 11.5 | 1 | 7 | 368 | 3201 | AB303563 | Virosaurus | DNA library |
| 26047 | 8/14/2015 | 778_D | L6-r113 | TTMDV | Anelloviridae | 26 | 2 | 27 | 840 | 3231 | AB303564 | Virosaurus | DNA library |
| 26049 | 8/10/2015 | 754_D | L5-r96 | TTMDV | Anelloviridae | 36.7 | 2 | 70 | 1170 | 3188 | AB303562 | Virosaurus | DNA library |
| 26050 | 8/10/2015 | 641_D | L4-r91 | TTMDV | Anelloviridae | 19.1 | 8 | 285 | 611 | 3199 | AB303553 | Virosaurus | DNA library |
| 26052 | 8/10/2015 | 755_D | L6-r96 | TTMDV | Anelloviridae | 91.59 | 35 | 1021 | 2766 | 3020 | MN780374 | De novo | DNA library |
| 26063 | 8/11/2015 | 756_D | L7-r96 | TTMDV | Anelloviridae | 14.76 | 2 | 13 | 472 | 3198 | AB303554 | Virosaurus | DNA library |
| 26074 | 8/10/2015 | 780_D | L8-r113 | TTMDV | Anelloviridae | 17.45 | 26 | 213 | 558 | 3198 | AB303554 | Virosaurus | DNA library |
| 26076 | 8/13/2015 | 757_D | L5-r93 | TTMDV | Anelloviridae | 41.97 | 10 | 580 | 1356 | 3231 | AB303564 | Virosaurus | DNA library |
| 26077 | 8/13/2015 | 643_D | L6-r91 | TTMDV | Anelloviridae | 61.57 | 2 | 197 | 1969 | 3198 | AB303554 | Virosaurus | DNA library |
| 26078 | 8/13/2015 | 642_D | L5-r91 | TTMDV | Anelloviridae | 82.5 | 18 | 1146 | 2630 | 3188 | KF545587 | Virosaurus | DNA library |
| 26079 | 8/13/2015 | 674_D | L1-r91 | TTMDV | Anelloviridae | 13.88 | 3 | 13 | 444 | 3198 | AB303554 | Virosaurus | DNA library |
| 26082 | 8/13/2015 | 758_D | L6-r93 | TTMDV | Anelloviridae | 16.48 | 6 | 135 | 527 | 3198 | AB303554 | Virosaurus | DNA library |
| 26083 | 8/13/2015 | 802_D | L6-r111 | TTMDV | Anelloviridae | 15.17 | 3 | 22 | 485 | 3198 | AB303554 | Virosaurus | DNA library |
| 26089 | 8/14/2015 | 760_D | L8-r93 | TTMDV | Anelloviridae | 16.14 | 6 | 46 | 516 | 3198 | AB303554 | Virosaurus | DNA library |
| 26091 | 8/14/2015 | 761_R | L1-r737 | TTMDV | Anelloviridae | 30.9 | 6 | 60 | 956 | 3094 | MN776386 | De novo | RNA library |
| 26091 | 8/14/2015 | 761_D | L5-r96 | TTMDV | Anelloviridae | 48.08 | 2 | 70 | 1292 | 2687 | KP343821 | Virosaurus | DNA library |
| 26100 | 8/17/2015 | 762_R | L1-r737 | TTMDV | Anelloviridae | 12.19 | 8 | 32 | 327 | 2683 | MN776387 | De novo | RNA library |
| 26100 | 8/17/2015 | 762_D | L6-r96 | TTMDV | Anelloviridae | 16.92 | 7 | 56 | 541 | 3198 | AB303554 | Virosaurus | DNA library |
| 26101 | 9/9/2015 | 763_D | L7-r96 | TTMDV | Anelloviridae | 48.82 | 1 | 25 | 1287 | 2636 | NC_007014 | Virosaurus | DNA library |
| 26102 | 8/18/2015 | 764_D | L5-r93 | TTMDV | Anelloviridae | 11.73 | 2 | 9 | 375 | 3198 | AB303554 | Virosaurus | DNA library |
| 26104 | 8/18/2015 | 765_D | L6-r93 | TTMDV | Anelloviridae | 16.25 | 2 | 23 | 524 | 3224 | AB303558 | Virosaurus | DNA library |
| 26107 | 8/18/2015 | 803_D | L7-r111 | TTMDV | Anelloviridae | 27.98 | 1 | 112 | 892 | 3188 | KF545587 | Virosaurus | DNA library |
| 26108 | 8/18/2015 | 804_D | L8-r111 | TTMDV | Anelloviridae | 28.36 | 1 | 12 | 792 | 2793 | KT163882 | Virosaurus | DNA library |
| 26111 | 8/18/2015 | 805_D | L4-r113 | TTMDV | Anelloviridae | 22.74 | 5 | 76 | 733 | 3224 | AB303558 | Virosaurus | DNA library |
| 26114 | 8/19/2015 | 644_D | L7-r91 | TTMDV | Anelloviridae | 94.36 | 24 | 771 | 2644 | 2802 | KF545588 | Virosaurus | DNA library |
| 26124 | 8/21/2015 | 806_D | L6-r113 | TTMDV | Anelloviridae | 89.96 | 62 | 1955 | 2777 | 3087 | MN776317 | De novo | DNA library |
| 26129 | 8/21/2015 | 807_D | L7-r113 | TTMDV | Anelloviridae | 18.23 | 6 | 166 | 581 | 3188 | AB303562 | Virosaurus | DNA library |
| 26130 | 8/24/2015 | 808_D | L8-r113 | TTMDV | Anelloviridae | 12.66 | 3 | 15 | 408 | 3224 | AB303558 | Virosaurus | DNA library |
| 26131 | 8/24/2015 | 645_D | L8-r91 | TTMDV | Anelloviridae | 99.17 | 10 | 604 | 2991 | 3016 | MN780396 | De novo | DNA library |
| 26134 | 8/24/2015 | 809_D | L6-r111 | TTMDV | Anelloviridae | 24.95 | 2 | 90 | 798 | 3199 | AB303553 | Virosaurus | DNA library |
| 26140 | 8/25/2015 | 647_D | L3-r91 | TTMDV | Anelloviridae | 23.82 | 9 | 376 | 762 | 3199 | AB303553 | Virosaurus | DNA library |
| 26141 | 8/25/2015 | 646_D | L1-r91 | TTMDV | Anelloviridae | 20.51 | 14 | 1253 | 656 | 3199 | AB303553 | Virosaurus | DNA library |
| 26142 | 8/25/2015 | 810_D | L7-r111 | TTMDV | Anelloviridae | 96.42 | 17 | 498 | 2693 | 2793 | KT163882 | Virosaurus | DNA library |
| 26144 | 8/25/2015 | 811_D | L4-r122 | TTMDV | Anelloviridae | 94.17 | 239 | 6223 | 2390 | 2538 | KP343822 | Virosaurus | DNA library |
| 26145 | 8/25/2015 | 812_D | L5-r122 | TTMDV | Anelloviridae | 30.98 | 9 | 100 | 984 | 3176 | AB303560 | Virosaurus | DNA library |
| 26147 | 8/25/2015 | 813_D | L6-r122 | TTMDV | Anelloviridae | 17.92 | 36 | 302 | 573 | 3198 | AB303554 | Virosaurus | DNA library |
| 26149 | 8/24/2015 | 648_D | L4-r91 | TTMDV | Anelloviridae | 52.48 | 2 | 100 | 1673 | 3188 | KF545587 | Virosaurus | DNA library |
| 26159 | 8/26/2015 | 815_D | L8-r122 | TTMDV | Anelloviridae | 62.17 | 2 | 280 | 1982 | 3188 | KF545587 | Virosaurus | DNA library |
| 26163 | 8/27/2015 | 817_D | L6-r124 | TTMDV | Anelloviridae | 26.79 | 1 | 146 | 854 | 3188 | KF545587 | Virosaurus | DNA library |
| 26165 | 8/26/2015 | 818_D | L7-r124 | TTMDV | Anelloviridae | 16.32 | 8 | 130 | 522 | 3198 | AB303554 | Virosaurus | DNA library |
| 26173 | 8/27/2015 | 819_D | L5-r122 | TTMDV | Anelloviridae | 25.95 | 1 | 14 | 727 | 2802 | KF545588 | Virosaurus | DNA library |
| 26174 | 8/27/2015 | 820_D | L6-r122 | TTMDV | Anelloviridae | 12.21 | 2 | 20 | 328 | 2687 | KP343821 | Virosaurus | DNA library |
| 26176 | 8/28/2015 | 649_D | L5-r91 | TTMDV | Anelloviridae | 17.95 | 21 | 184 | 574 | 3198 | AB303554 | Virosaurus | DNA library |
| 26177 | 8/28/2015 | 821_R | L1-r741 | TTMDV | Anelloviridae | 29.67 | 2 | 106 | 818 | 2757 | MN778837 | De novo | RNA library |
| 26177 | 8/28/2015 | 821_D | L7-r122 | TTMDV | Anelloviridae | 53.67 | 38 | 7303 | 1734 | 3231 | AB303564 | Virosaurus | DNA library |
| 26178 | 8/28/2015 | 822_D | L8-r122 | TTMDV | Anelloviridae | 17.32 | 84 | 589 | 554 | 3198 | AB303554 | Virosaurus | DNA library |
| 26179 | 8/28/2015 | 823_D | L5-r124 | TTMDV | Anelloviridae | 55.13 | 2 | 114 | 1763 | 3198 | AB303554 | Virosaurus | DNA library |
| 26181 | 8/28/2015 | 824_D | L6-r124 | TTMDV | Anelloviridae | 100 | 1801 | 55664 | 3019 | 3019 | MN776048 | De novo | DNA library |
| 26186 | 8/31/2015 | 826_D | L4-r122 | TTMDV | Anelloviridae | 33.04 | 4 | 120 | 1052 | 3184 | AB303565 | Virosaurus | DNA library |
| 26193 | 8/31/2015 | 827_D | L6-r122 | TTMDV | Anelloviridae | 15.73 | 10 | 55 | 503 | 3198 | AB303554 | Virosaurus | DNA library |
| 26194 | 8/31/2015 | 828_D | L7-r122 | TTMDV | Anelloviridae | 28.17 | 8 | 93 | 910 | 3231 | AB303564 | Virosaurus | DNA library |
| 26198 | 9/1/2015 | 829_D | L8-r122 | TTMDV | Anelloviridae | 14.32 | 7 | 51 | 458 | 3198 | AB303554 | Virosaurus | DNA library |
| 26200 | 9/1/2015 | 830_D | L5-r124 | TTMDV | Anelloviridae | 100 | 40 | 1261 | 2687 | 2687 | KP343821 | Virosaurus | DNA library |
| 26203 | 9/1/2015 | 831_D | L6-r124 | TTMDV | Anelloviridae | 99.22 | 1217 | 37141 | 3162 | 3187 | MN780434 | De novo | DNA library |
| 26204 | 9/1/2015 | 832_D | L7-r124 | TTMDV | Anelloviridae | 46.15 | 7 | 103 | 1488 | 3224 | AB303566 | Virosaurus | DNA library |
| 26208 | 9/2/2015 | 652_D | L8-r91 | TTMDV | Anelloviridae | 18.7 | 39 | 428 | 598 | 3198 | AB303554 | Virosaurus | DNA library |
| 26210 | 9/2/2015 | 833_R | L4-r741 | TTMDV | Anelloviridae | 89.06 | 31 | 1049 | 2393 | 2687 | MN777142 | De novo | RNA library |
| 26210 | 9/2/2015 | 833_D | L4-r122 | TTMDV | Anelloviridae | 96.31 | 15 | 540 | 2690 | 2793 | KT163882 | Virosaurus | DNA library |
| 26213 | 9/2/2015 | 834_D | L5-r122 | TTMDV | Anelloviridae | 17.88 | 3 | 63 | 572 | 3199 | AB303553 | Virosaurus | DNA library |
| 26214 | 9/2/2015 | 651_D | L7-r91 | TTMDV | Anelloviridae | 65.67 | 22 | 3676 | 2100 | 3198 | AB303554 | Virosaurus | DNA library |
| 26215 | 9/3/2015 | 835_D | L7-r122 | TTMDV | Anelloviridae | 31.88 | 4 | 108 | 809 | 2538 | KP343822 | Virosaurus | DNA library |
| 26219 | 9/3/2015 | 836_D | L8-r122 | TTMDV | Anelloviridae | 23.74 | 1 | 11 | 760 | 3201 | AB303556 | Virosaurus | DNA library |
| 26221 | 9/4/2015 | 837_D | L5-r124 | TTMDV | Anelloviridae | 69.01 | 4 | 375 | 2200 | 3188 | KF545587 | Virosaurus | DNA library |
| 26222 | 9/4/2015 | 838_R | L5-r741 | TTMDV | Anelloviridae | 14.95 | 7 | 32 | 456 | 3050 | MN779786 | De novo | RNA library |
| 26222 | 9/4/2015 | 838_D | L6-r124 | TTMDV | Anelloviridae | 92.6 | 317 | 11523 | 2952 | 3188 | KF545587 | Virosaurus | DNA library |
| 26224 | 9/4/2015 | 839_D | L7-r124 | TTMDV | Anelloviridae | 24.72 | 4 | 31 | 785 | 3176 | AB303560 | Virosaurus | DNA library |
| 26225 | 9/4/2015 | 840_D | L4-r122 | TTMDV | Anelloviridae | 100 | 74 | 2048 | 2638 | 2638 | MN780064 | De novo | DNA library |
| 26229 | 9/4/2015 | 653_D | L1-r91 | TTMDV | Anelloviridae | 15.43 | 8 | 58 | 501 | 3246 | NC_009225 | Virosaurus | DNA library |
| 26234 | 9/7/2015 | 841_D | L5-r122 | TTMDV | Anelloviridae | 86.53 | 46 | 1487 | 2609 | 3015 | MN777000 | De novo | DNA library |
| 26236 | 9/7/2015 | 842_D | L6-r122 | TTMDV | Anelloviridae | 82.48 | 12 | 316 | 2311 | 2802 | KF545588 | Virosaurus | DNA library |
| 26237 | 9/7/2015 | 843_D | L8-r122 | TTMDV | Anelloviridae | 100 | 13 | 429 | 2687 | 2687 | KP343821 | Virosaurus | DNA library |
| 26242 | 9/7/2015 | 844_D | L5-r124 | TTMDV | Anelloviridae | 25.6 | 2 | 26 | 815 | 3184 | AB303565 | Virosaurus | DNA library |
| 26243 | 9/7/2015 | 869_D | L5-r132 | TTMDV | Anelloviridae | 80.26 | 6 | 153 | 2249 | 2802 | KF545588 | Virosaurus | DNA library |
| 26244 | 9/7/2015 | 845_D | L6-r124 | TTMDV | Anelloviridae | 99.64 | 816 | 22865 | 3037 | 3048 | MN780010 | De novo | DNA library |
| 26247 | 9/8/2015 | 846_D | L7-r124 | TTMDV | Anelloviridae | 12.63 | 2 | 34 | 404 | 3199 | AB303553 | Virosaurus | DNA library |
| 26250 | 9/8/2015 | 676_R | L1-r734 | TTMDV | Anelloviridae | 14.89 | 4 | 17 | 400 | 2687 | MN777142 | De novo | RNA library |
| 26250 | 9/8/2015 | 676_D | L3-r92 | TTMDV | Anelloviridae | 70.83 | 12 | 1778 | 2265 | 3198 | AB303554 | Virosaurus | DNA library |
| 26251 | 9/8/2015 | 847_D | L4-r122 | TTMDV | Anelloviridae | 38.97 | 4 | 117 | 1268 | 3254 | NC_014093 | Virosaurus | DNA library |
| 26253 | 9/8/2015 | 848_D | L5-r122 | TTMDV | Anelloviridae | 28.86 | 3 | 45 | 923 | 3198 | AB303554 | Virosaurus | DNA library |
| 26254 | 9/8/2015 | 849_D | L6-r122 | TTMDV | Anelloviridae | 32.96 | 5 | 519 | 1055 | 3201 | AB303563 | Virosaurus | DNA library |
| 26256 | 9/8/2015 | 850_D | L7-r122 | TTMDV | Anelloviridae | 28.82 | 8 | 103 | 931 | 3231 | AB303564 | Virosaurus | DNA library |
| 26257 | 9/9/2015 | 851_D | L5-r124 | TTMDV | Anelloviridae | 24.45 | 26 | 1193 | 657 | 2687 | KP343821 | Virosaurus | DNA library |
| 26259 | 9/9/2015 | 852_D | L6-r124 | TTMDV | Anelloviridae | 76.15 | 59 | 1477 | 2276 | 2989 | MN774969 | De novo | DNA library |
| 26260 | 9/9/2015 | 853_D | L7-r124 | TTMDV | Anelloviridae | 23.98 | 9 | 103 | 767 | 3198 | AB303554 | Virosaurus | DNA library |
| 26261 | 9/9/2015 | 654_D | L2-r91 | TTMDV | Anelloviridae | 29.69 | 3 | 40 | 943 | 3176 | AB303560 | Virosaurus | DNA library |
| 26262 | 9/9/2015 | 677_D | L4-r92 | TTMDV | Anelloviridae | 19.79 | 7 | 487 | 638 | 3224 | AB303558 | Virosaurus | DNA library |
| 26263 | 9/9/2015 | 854_D | L4-r122 | TTMDV | Anelloviridae | 64.05 | 5 | 147 | 2042 | 3188 | AB303559 | Virosaurus | DNA library |
| 26265 | 9/9/2015 | 855_D | L5-r122 | TTMDV | Anelloviridae | 15.8 | 5 | 38 | 503 | 3184 | AB303565 | Virosaurus | DNA library |
| 26267 | 9/9/2015 | 870_D | L6-r132 | TTMDV | Anelloviridae | 100 | 679 | 22972 | 2687 | 2687 | KP343821 | Virosaurus | DNA library |
| 26276 | 9/10/2015 | 656_R | L2-r725 | TTMDV | Anelloviridae | 13.59 | 6 | 29 | 395 | 2907 | MN775975 | De novo | RNA library |
| 26276 | 9/10/2015 | 656_D | L5-r91 | TTMDV | Anelloviridae | 77.27 | 5 | 146 | 2165 | 2802 | KF545588 | Virosaurus | DNA library |
| 26278 | 9/10/2015 | 657_D | L6-r91 | TTMDV | Anelloviridae | 29 | 1 | 46 | 935 | 3224 | AB303566 | Virosaurus | DNA library |
| 26279 | 9/10/2015 | 872_D | L1-r132 | TTMDV | Anelloviridae | 15.51 | 12 | 92 | 496 | 3198 | AB303554 | Virosaurus | DNA library |
| 26280 | 9/11/2015 | 659_D | L8-r91 | TTMDV | Anelloviridae | 66.78 | 4 | 217 | 2153 | 3224 | AB303566 | Virosaurus | DNA library |
| 26281 | 9/11/2015 | 658_D | L7-r91 | TTMDV | Anelloviridae | 78.77 | 4 | 138 | 2200 | 2793 | KT163882 | Virosaurus | DNA library |
| 26282 | 9/11/2015 | 873_D | L2-r132 | TTMDV | Anelloviridae | 98.97 | 527 | 17425 | 3154 | 3187 | MN780434 | De novo | DNA library |
| 26284 | 9/11/2015 | 874_D | L3-r132 | TTMDV | Anelloviridae | 39.26 | 2 | 183 | 1055 | 2687 | KP343821 | Virosaurus | DNA library |
| 26287 | 9/11/2015 | 876_D | L5-r132 | TTMDV | Anelloviridae | 15.76 | 3 | 38 | 504 | 3198 | AB303554 | Virosaurus | DNA library |
| 26293 | 9/14/2015 | 877_D | L6-r132 | TTMDV | Anelloviridae | 99.41 | 776 | 22924 | 3025 | 3043 | MN780398 | De novo | DNA library |
| 26294 | 9/14/2015 | 878_D | L8-r124 | TTMDV | Anelloviridae | 20.57 | 3 | 32 | 663 | 3224 | AB303558 | Virosaurus | DNA library |
| 26295 | 9/14/2015 | 879_D | L8-r127 | TTMDV | Anelloviridae | 13.04 | 2 | 17 | 417 | 3198 | AB303554 | Virosaurus | DNA library |
| 26299 | 9/14/2015 | 880_D | L2-r132 | TTMDV | Anelloviridae | 89.76 | 32 | 6469 | 2770 | 3086 | MN779902 | De novo | DNA library |
| 26301 | 9/15/2015 | 881_D | L3-r132 | TTMDV | Anelloviridae | 34.32 | 1 | 86 | 871 | 2538 | KP343822 | Virosaurus | DNA library |
| 26309 | 9/15/2015 | 882_D | L4-r132 | TTMDV | Anelloviridae | 98.11 | 61 | 12505 | 3168 | 3229 | MN774952 | De novo | DNA library |
| 26311 | 9/15/2015 | 883_D | L5-r132 | TTMDV | Anelloviridae | 18.39 | 4 | 143 | 593 | 3224 | AB303558 | Virosaurus | DNA library |
| 26312 | 9/15/2015 | 884_D | L6-r132 | TTMDV | Anelloviridae | 39.92 | 13 | 278 | 1268 | 3176 | AB303560 | Virosaurus | DNA library |
| 26317 | 9/16/2015 | 678_D | L5-r92 | TTMDV | Anelloviridae | 96.29 | 22 | 636 | 2801 | 2909 | MN776339 | De novo | DNA library |
| 26318 | 9/16/2015 | 885_D | L8-r124 | TTMDV | Anelloviridae | 53.82 | 1 | 29 | 1508 | 2802 | KF545588 | Virosaurus | DNA library |
| 26323 | 9/16/2015 | 886_D | L8-r127 | TTMDV | Anelloviridae | 24.89 | 2 | 26 | 810 | 3254 | NC_014093 | Virosaurus | DNA library |
| 26330 | 9/17/2015 | 887_D | L1-r132 | TTMDV | Anelloviridae | 58.38 | 2 | 75 | 1861 | 3188 | KF545587 | Virosaurus | DNA library |
| 26331 | 9/17/2015 | 888_D | L3-r132 | TTMDV | Anelloviridae | 37.53 | 31 | 630 | 1192 | 3176 | AB303560 | Virosaurus | DNA library |
| 26332 | 9/17/2015 | 889_D | L4-r132 | TTMDV | Anelloviridae | 27.21 | 1 | 1447 | 731 | 2687 | KP343821 | Virosaurus | DNA library |
| 26334 | 9/17/2015 | 890_D | L5-r132 | TTMDV | Anelloviridae | 18.64 | 6 | 67 | 601 | 3224 | AB303558 | Virosaurus | DNA library |
| 26336 | 9/17/2015 | 891_D | L6-r132 | TTMDV | Anelloviridae | 95.32 | 329 | 12318 | 2934 | 3078 | MN775845 | De novo | DNA library |
| 26340 | 9/17/2015 | 892_D | L8-r124 | TTMDV | Anelloviridae | 27.23 | 6 | 305 | 871 | 3199 | AB303553 | Virosaurus | DNA library |
| 26342 | 9/17/2015 | 893_D | L8-r127 | TTMDV | Anelloviridae | 32.37 | 29 | 505 | 1028 | 3176 | AB303560 | Virosaurus | DNA library |
| 26343 | 9/17/2015 | 894_D | L1-r132 | TTMDV | Anelloviridae | 77.84 | 6 | 152 | 2181 | 2802 | KF545588 | Virosaurus | DNA library |
| 26345 | 9/17/2015 | 679_D | L6-r92 | TTMDV | Anelloviridae | 30.77 | 7 | 420 | 992 | 3224 | AB303558 | Virosaurus | DNA library |
| 26346 | 9/17/2015 | 895_D | L2-r132 | TTMDV | Anelloviridae | 99.36 | 182 | 10968 | 3089 | 3109 | MN780399 | De novo | DNA library |
| 26351 | 9/21/2015 | 896_D | L4-r132 | TTMDV | Anelloviridae | 99.22 | 836 | 28012 | 3041 | 3065 | MN780385 | De novo | DNA library |
| 26353 | 9/21/2015 | 897_D | L5-r132 | TTMDV | Anelloviridae | 16.29 | 10 | 71 | 521 | 3198 | AB303554 | Virosaurus | DNA library |
| 26354 | 9/21/2015 | 898_D | L6-r132 | TTMDV | Anelloviridae | 38.92 | 12 | 279 | 1236 | 3176 | AB303560 | Virosaurus | DNA library |
| 26360 | 9/22/2015 | 899_D | L8-r124 | TTMDV | Anelloviridae | 55.52 | 1 | 137 | 1770 | 3188 | KF545587 | Virosaurus | DNA library |
| 26361 | 9/22/2015 | 900_D | L8-r127 | TTMDV | Anelloviridae | 15.6 | 4 | 44 | 503 | 3224 | AB303558 | Virosaurus | DNA library |
| 26368 | 9/22/2015 | 671_D | L6-r91 | TTMDV | Anelloviridae | 40.86 | 1 | 17 | 1145 | 2802 | KF545588 | Virosaurus | DNA library |
| 26399 | 9/29/2015 | 680_D | L1-r93 | TTMDV | Anelloviridae | 17.32 | 16 | 256 | 554 | 3198 | AB303554 | Virosaurus | DNA library |
| 26424 | 10/23/2015 | 1_D | L008-r54 | TTMDV | Anelloviridae | 18.31 | 2 | 79 | 586 | 3201 | AB303556 | Virosaurus | DNA library |
| 26425 | 10/23/2015 | 9_D | L002-r44 | TTMDV | Anelloviridae | 43.51 | 2 | 73 | 1387 | 3188 | AB303559 | Virosaurus | DNA library |
| 26432 | 10/26/2015 | 25_D | L8-r96 | TTMDV | Anelloviridae | 86.8 | 9 | 284 | 2669 | 3075 | MN776349 | De novo | DNA library |
| 26436 | 10/27/2015 | 44_D | L001-r44 | TTMDV | Anelloviridae | 98.84 | 2292 | 70472 | 3158 | 3195 | MN774944 | De novo | DNA library |
| 26439 | 10/28/2015 | 2_D | L002-r44 | TTMDV | Anelloviridae | 41.5 | 3 | 247 | 1327 | 3198 | AB303554 | Virosaurus | DNA library |
| 26446 | 10/29/2015 | 26_R | L8-r669 | TTMDV | Anelloviridae | 21.96 | 1 | 10 | 607 | 2764 | MN777134 | De novo | RNA library |
| 26446 | 10/29/2015 | 26_D | L005-r44 | TTMDV | Anelloviridae | 94.86 | 8 | 218 | 2622 | 2764 | MN777134 | De novo | DNA library |
| 26447 | 10/29/2015 | 18_R | L4-r669 | TTMDV | Anelloviridae | 13.07 | 1 | 8 | 382 | 2922 | MN776995 | De novo | RNA library |
| 26447 | 10/29/2015 | 18_D | L004-r44 | TTMDV | Anelloviridae | 53.8 | 3 | 234 | 1715 | 3188 | AB303559 | Virosaurus | DNA library |
| 26455 | 11/2/2015 | 27_D | L006-r44 | TTMDV | Anelloviridae | 36.59 | 6 | 98 | 1165 | 3184 | AB303565 | Virosaurus | DNA library |
| 26459 | 11/3/2015 | 4_D | L004-r44 | TTMDV | Anelloviridae | 56.23 | 8 | 272 | 1813 | 3224 | AB303566 | Virosaurus | DNA library |
| 26462 | 11/3/2015 | 12_D | L005-r44 | TTMDV | Anelloviridae | 21.15 | 6 | 121 | 682 | 3224 | AB303558 | Virosaurus | DNA library |
| 26466 | 11/6/2015 | 786_D | L7-r113 | TTMDV | Anelloviridae | 24.03 | 2 | 60 | 780 | 3246 | NC_009225 | Virosaurus | DNA library |
| 26472 | 11/6/2015 | 33_R | L001-r674 | TTMDV | Anelloviridae | 30.76 | 2 | 22 | 808 | 2627 | MN776101 | De novo | RNA library |
| 26472 | 11/6/2015 | 33_D | L005-r44 | TTMDV | Anelloviridae | 70.39 | 19 | 720 | 2244 | 3188 | AB303559 | Virosaurus | DNA library |
| 26479 | 11/10/2015 | 40_D | L004-r44 | TTMDV | Anelloviridae | 94.76 | 135 | 3534 | 2477 | 2614 | MN777449 | De novo | DNA library |
| 26482 | 11/10/2015 | 672_D | L7-r91 | TTMDV | Anelloviridae | 14.63 | 6 | 38 | 468 | 3198 | AB303554 | Virosaurus | DNA library |
| 26483 | 11/10/2015 | 21_R | L6-r669 | TTMDV | Anelloviridae | 22.7 | 3 | 20 | 604 | 2661 | MN777014 | De novo | RNA library |
| 26483 | 11/10/2015 | 21_D | L008-r54 | TTMDV | Anelloviridae | 86.23 | 16 | 1294 | 2749 | 3188 | KF545587 | Virosaurus | DNA library |
| 26485 | 11/11/2015 | 787_D | L8-r113 | TTMDV | Anelloviridae | 13.25 | 2 | 152 | 424 | 3199 | AB303553 | Virosaurus | DNA library |
| 26487 | 11/11/2015 | 6_D | L006-r44 | TTMDV | Anelloviridae | 67.67 | 11 | 456 | 2164 | 3198 | AB303554 | Virosaurus | DNA library |
| 26488 | 11/11/2015 | 15_D | L008-r44 | TTMDV | Anelloviridae | 25.09 | 5 | 216 | 809 | 3224 | AB303558 | Virosaurus | DNA library |
| 26492 | 11/12/2015 | 5_D | L005-r44 | TTMDV | Anelloviridae | 41.03 | 13 | 224 | 1303 | 3176 | AB303560 | Virosaurus | DNA library |
| 26493 | 11/12/2015 | 14_D | L004-r55 | TTMDV | Anelloviridae | 21.36 | 195 | 1712 | 683 | 3198 | AB303554 | Virosaurus | DNA library |
| 26494 | 11/12/2015 | 22_D | L008-r44 | TTMDV | Anelloviridae | 11.57 | 3 | 11 | 370 | 3198 | AB303554 | Virosaurus | DNA library |
| 26495 | 11/12/2015 | 28_D | L002-r55 | TTMDV | Anelloviridae | 19.29 | 6 | 100 | 622 | 3224 | AB303558 | Virosaurus | DNA library |
| 26498 | 11/13/2015 | 34_D | L006-r44 | TTMDV | Anelloviridae | 20.88 | 1 | 29 | 673 | 3224 | AB303566 | Virosaurus | DNA library |
| 26502 | 11/16/2015 | 673_R | L6-r725 | TTMDV | Anelloviridae | 58.16 | 9 | 232 | 1754 | 3016 | MN780396 | De novo | RNA library |
| 26502 | 11/16/2015 | 673_D | L8-r91 | TTMDV | Anelloviridae | 44.2 | 28 | 1781 | 1428 | 3231 | AB303564 | Virosaurus | DNA library |
| 26503 | 11/16/2015 | 41_D | L006-r44 | TTMDV | Anelloviridae | 52.35 | 4 | 217 | 1674 | 3198 | AB303554 | Virosaurus | DNA library |
| 26506 | 11/17/2015 | 7_D | L007-r44 | TTMDV | Anelloviridae | 40.09 | 5 | 219 | 1282 | 3198 | AB303554 | Virosaurus | DNA library |
| 26507 | 11/17/2015 | 30_D | L001-r44 | TTMDV | Anelloviridae | 100 | 9864 | 290485 | 2687 | 2687 | KP343821 | Virosaurus | DNA library |
| 26507 | 11/17/2015 | 30_R | L7-r669 | TTMDV | Anelloviridae | 29.48 | 1 | 8 | 792 | 2687 | KP343821 | Virosaurus | RNA library |
| 26512 | 11/19/2015 | 8_D | L005-r55 | TTMDV | Anelloviridae | 20.09 | 1 | 8 | 563 | 2802 | KF545588 | Virosaurus | DNA library |
| 26513 | 11/19/2015 | 17_D | L003-r44 | TTMDV | Anelloviridae | 46.66 | 16 | 348 | 1482 | 3176 | AB303560 | Virosaurus | DNA library |
| 26518 | 11/23/2015 | 790_D | L4-r113 | TTMDV | Anelloviridae | 16.34 | 1 | 7 | 523 | 3201 | AB303556 | Virosaurus | DNA library |
| 26520 | 11/23/2015 | 36_D | L001-r55 | TTMDV | Anelloviridae | 58.94 | 48 | 955 | 1800 | 3054 | MN778921 | De novo | DNA library |
| 26522 | 11/26/2015 | 661_D | L2-r91 | TTMDV | Anelloviridae | 67.08 | 21 | 565 | 2003 | 2986 | MN776999 | De novo | DNA library |
| 26524 | 11/23/2015 | 43_D | L008-r54 | TTMDV | Anelloviridae | 16.82 | 31 | 312 | 538 | 3198 | AB303554 | Virosaurus | DNA library |
| 26531 | 11/24/2015 | 77_D | L003-r46 | TTMDV | Anelloviridae | 86.89 | 32 | 1520 | 2770 | 3188 | KF545587 | Virosaurus | DNA library |
| 26533 | 11/24/2015 | 663_D | L5-r91 | TTMDV | Anelloviridae | 99.45 | 4712 | 143902 | 3078 | 3095 | MN780372 | De novo | DNA library |
| 26533 | 11/24/2015 | 663_R | L4-r725 | TTMDV | Anelloviridae | 10.47 | 12 | 39 | 324 | 3095 | MN780372 | De novo | RNA library |
| 26534 | 11/24/2015 | 69_D | L008-r54 | TTMDV | Anelloviridae | 31.92 | 2 | 109 | 1029 | 3224 | AB303566 | Virosaurus | DNA library |
| 26536 | 11/24/2015 | 52_D | L007-r46 | TTMDV | Anelloviridae | 86.41 | 10 | 264 | 2193 | 2538 | KP343822 | Virosaurus | DNA library |
| 26540 | 11/26/2015 | 87_D | L007-r46 | TTMDV | Anelloviridae | 85.69 | 26 | 704 | 2401 | 2802 | KF545588 | Virosaurus | DNA library |
| 26546 | 11/30/2015 | 70_D | L004-r46 | TTMDV | Anelloviridae | 40.86 | 2 | 248 | 1142 | 2795 | KP343824 | Virosaurus | DNA library |
| 26551 | 12/2/2015 | 88_D | L008-r46 | TTMDV | Anelloviridae | 94.99 | 16 | 511 | 2653 | 2793 | KT163882 | Virosaurus | DNA library |
| 26557 | 12/3/2015 | 54_D | L002-r46 | TTMDV | Anelloviridae | 16.26 | 6 | 43 | 520 | 3198 | AB303554 | Virosaurus | DNA library |
| 26558 | 12/4/2015 | 74_D | L008-r46 | TTMDV | Anelloviridae | 38.98 | 17 | 339 | 1238 | 3176 | AB303560 | Virosaurus | DNA library |
| 26559 | 12/7/2015 | 84_D | L003-r46 | TTMDV | Anelloviridae | 94.84 | 48 | 4067 | 3030 | 3195 | MN774944 | De novo | DNA library |
| 26562 | 12/10/2015 | 55_D | L003-r46 | TTMDV | Anelloviridae | 43.64 | 33 | 1296 | 1410 | 3231 | AB303564 | Virosaurus | DNA library |
| 21004 | 12/12/2014 | 91_D | L001-r49 | TTMV | Anelloviridae | 12.23 | 86 | 472 | 361 | 2953 | NC_014082 | Virosaurus | DNA library |
| 21006 | 1/16/2015 | 92_D | L002-r49 | TTMV | Anelloviridae | 78.23 | 11 | 266 | 2217 | 2834 | KP343848 | Virosaurus | DNA library |
| 21007 | 1/19/2015 | 794_D | L8-r113 | TTMV | Anelloviridae | 15.14 | 70 | 670 | 451 | 2978 | KP343854 | Virosaurus | DNA library |
| 21014 | 1/23/2015 | 93_D | L003-r49 | TTMV | Anelloviridae | 11.42 | 14 | 57 | 340 | 2978 | KP343854 | Virosaurus | DNA library |
| 21017 | 1/23/2015 | 766_R | L3-r737 | TTMV | Anelloviridae | 16.1 | 1 | 10 | 465 | 2889 | MN770009 | De novo | RNA library |
| 21017 | 1/23/2015 | 766_D | L6-r111 | TTMV | Anelloviridae | 94.75 | 73 | 2147 | 2763 | 2916 | JX134046 | Virosaurus | DNA library |
| 21018 | 1/26/2015 | 94_D | L004-r49 | TTMV | Anelloviridae | 81.27 | 46 | 1281 | 2321 | 2856 | NC_025727 | Virosaurus | DNA library |
| 21019 | 1/26/2015 | 95_D | L005-r49 | TTMV | Anelloviridae | 99.65 | 403 | 12001 | 2852 | 2862 | MN774292 | De novo | DNA library |
| 21021 | 1/26/2015 | 96_D | L006-r49 | TTMV | Anelloviridae | 100 | 19883 | 557082 | 2936 | 2936 | MN770873 | De novo | DNA library |
| 21026 | 1/28/2015 | 97_D | L007-r49 | TTMV | Anelloviridae | 12.16 | 6 | 94 | 362 | 2978 | KP343854 | Virosaurus | DNA library |
| 21027 | 1/28/2015 | 98_D | L008-r49 | TTMV | Anelloviridae | 31.48 | 3 | 36 | 917 | 2913 | EF538882 | Virosaurus | DNA library |
| 21028 | 1/28/2015 | 796_D | L7-r111 | TTMV | Anelloviridae | 86.03 | 6 | 191 | 2438 | 2834 | KP343848 | Virosaurus | DNA library |
| 21031 | 1/29/2015 | 586_D | L7-r88 | TTMV | Anelloviridae | 97.36 | 632 | 23391 | 2992 | 3073 | KF545582 | Virosaurus | DNA library |
| 21031 | 1/29/2015 | 586_R | L006-r722 | TTMV | Anelloviridae | 26.07 | 1 | 16 | 801 | 3073 | KF545582 | Virosaurus | RNA library |
| 21039 | 2/2/2015 | 99_D | L002-r49 | TTMV | Anelloviridae | 99.93 | 14 | 447 | 2829 | 2831 | KX810063 | Virosaurus | DNA library |
| 21041 | 2/2/2015 | 100_R | L003-r676 | TTMV | Anelloviridae | 11.46 | 2 | 15 | 337 | 2942 | MN770127 | De novo | RNA library |
| 21041 | 2/2/2015 | 100_D | L003-r55 | TTMV | Anelloviridae | 69.3 | 9 | 277 | 2020 | 2915 | NC_025726 | Virosaurus | DNA library |
| 21042 | 2/3/2015 | 101_D | L002-r55 | TTMV | Anelloviridae | 14.34 | 31 | 270 | 427 | 2978 | KP343854 | Virosaurus | DNA library |
| 21044 | 2/3/2015 | 102_D | L005-r49 | TTMV | Anelloviridae | 76.79 | 13 | 329 | 2193 | 2856 | NC_025727 | Virosaurus | DNA library |
| 21048 | 2/4/2015 | 103_D | L001-r55 | TTMV | Anelloviridae | 40.63 | 1 | 21 | 1043 | 2567 | KF545580 | Virosaurus | DNA library |
| 21053 | 2/6/2015 | 104_D | L007-r49 | TTMV | Anelloviridae | 78.19 | 67 | 1835 | 2280 | 2916 | JX134046 | Virosaurus | DNA library |
| 21058 | 2/10/2015 | 105_D | L008-r49 | TTMV | Anelloviridae | 12.35 | 47 | 176 | 369 | 2989 | KP343818 | Virosaurus | DNA library |
| 21060 | 2/13/2015 | 106_D | L001-r49 | TTMV | Anelloviridae | 92.11 | 9 | 290 | 2685 | 2915 | NC_025726 | Virosaurus | DNA library |
| 21074 | 2/19/2015 | 107_D | L003-r49 | TTMV | Anelloviridae | 42.4 | 2 | 37 | 1252 | 2953 | NC_014082 | Virosaurus | DNA library |
| 21077 | 2/19/2015 | 108_D | L004-r49 | TTMV | Anelloviridae | 37.27 | 1 | 23 | 1080 | 2898 | NC_014088 | Virosaurus | DNA library |
| 21080 | 2/20/2015 | 109_D | L005-r49 | TTMV | Anelloviridae | 99.63 | 66 | 4012 | 2926 | 2937 | MN771223 | De novo | DNA library |
| 21082 | 2/20/2015 | 110_D | L006-r49 | TTMV | Anelloviridae | 98.06 | 55 | 1644 | 2874 | 2931 | MN771395 | De novo | DNA library |
| 21085 | 2/23/2015 | 112_D | L008-r49 | TTMV | Anelloviridae | 88.44 | 13 | 391 | 2579 | 2916 | JX134046 | Virosaurus | DNA library |
| 21090 | 2/24/2015 | 113_D | L001-r49 | TTMV | Anelloviridae | 79.36 | 3 | 71 | 2249 | 2834 | KP343848 | Virosaurus | DNA library |
| 21092 | 2/27/2015 | 114_D | L002-r49 | TTMV | Anelloviridae | 25.38 | 3 | 29 | 716 | 2821 | KP343863 | Virosaurus | DNA library |
| 21099 | 2/26/2015 | 116_D | L005-r49 | TTMV | Anelloviridae | 87.88 | 552 | 16969 | 2488 | 2831 | KX810063 | Virosaurus | DNA library |
| 21102 | 2/27/2015 | 118_D | L007-r49 | TTMV | Anelloviridae | 43.25 | 5 | 88 | 1260 | 2913 | EF538882 | Virosaurus | DNA library |
| 21104 | 2/27/2015 | 119_D | L008-r49 | TTMV | Anelloviridae | 42.56 | 2 | 33 | 1252 | 2942 | KP343858 | Virosaurus | DNA library |
| 21105 | 3/2/2015 | 120_D | L001-r49 | TTMV | Anelloviridae | 41.77 | 3 | 42 | 1215 | 2909 | NC_014089 | Virosaurus | DNA library |
| 21109 | 3/11/2015 | 767_D | L7-r111 | TTMV | Anelloviridae | 98.43 | 12246 | 344515 | 2949 | 2996 | MN771181 | De novo | DNA library |
| 21110 | 3/3/2015 | 121_D | L002-r49 | TTMV | Anelloviridae | 30.11 | 1 | 18 | 878 | 2916 | JX134046 | Virosaurus | DNA library |
| 21115 | 3/4/2015 | 122_D | L003-r49 | TTMV | Anelloviridae | 47.89 | 9 | 204 | 1393 | 2909 | NC_014089 | Virosaurus | DNA library |
| 21116 | 3/4/2015 | 123_D | L005-r49 | TTMV | Anelloviridae | 53.14 | 8 | 198 | 1548 | 2913 | EF538882 | Virosaurus | DNA library |
| 21122 | 3/5/2015 | 124_R | L001-r677 | TTMV | Anelloviridae | 22.74 | 11 | 79 | 665 | 2924 | MN770144 | De novo | RNA library |
| 21122 | 3/5/2015 | 124_D | L006-r49 | TTMV | Anelloviridae | 98.01 | 1036 | 38940 | 2855 | 2913 | NC_020498 | Virosaurus | DNA library |
| 21124 | 3/16/2015 | 768_D | L8-r111 | TTMV | Anelloviridae | 97.49 | 148 | 3969 | 2874 | 2948 | MN769542 | De novo | DNA library |
| 21129 | 3/10/2015 | 125_D | L007-r49 | TTMV | Anelloviridae | 46.16 | 3 | 51 | 1346 | 2916 | JX134046 | Virosaurus | DNA library |
| 21138 | 3/11/2015 | 769_D | L4-r113 | TTMV | Anelloviridae | 18.11 | 2 | 8 | 501 | 2766 | NC_014086 | Virosaurus | DNA library |
| 21148 | 3/13/2015 | 126_D | L008-r49 | TTMV | Anelloviridae | 24.22 | 2 | 16 | 670 | 2766 | NC_014086 | Virosaurus | DNA library |
| 21154 | 3/16/2015 | 770_D | L5-r113 | TTMV | Anelloviridae | 94.41 | 14 | 479 | 2752 | 2915 | NC_025726 | Virosaurus | DNA library |
| 21158 | 3/17/2015 | 127_D | L001-r49 | TTMV | Anelloviridae | 34.78 | 4 | 55 | 1013 | 2913 | EF538882 | Virosaurus | DNA library |
| 21159 | 3/17/2015 | 128_D | L002-r49 | TTMV | Anelloviridae | 20.19 | 2 | 12 | 513 | 2541 | KT163888 | Virosaurus | DNA library |
| 21169 | 3/19/2015 | 129_D | L003-r49 | TTMV | Anelloviridae | 10.81 | 8 | 50 | 322 | 2978 | KP343854 | Virosaurus | DNA library |
| 21175 | 3/16/2015 | 130_D | L004-r49 | TTMV | Anelloviridae | 66.26 | 8 | 150 | 1701 | 2567 | KF545580 | Virosaurus | DNA library |
| 21176 | 3/16/2015 | 131_D | L006-r49 | TTMV | Anelloviridae | 97.47 | 528 | 20385 | 2922 | 2998 | MN770541 | De novo | DNA library |
| 21177 | 3/17/2015 | 132_D | L007-r49 | TTMV | Anelloviridae | 86.19 | 11 | 334 | 2440 | 2831 | KX810063 | Virosaurus | DNA library |
| 21180 | 3/13/2015 | 133_D | L008-r49 | TTMV | Anelloviridae | 38.96 | 8 | 107 | 1135 | 2913 | EF538882 | Virosaurus | DNA library |
| 21183 | 3/13/2015 | 134_D | L001-r49 | TTMV | Anelloviridae | 100 | 4579 | 133268 | 2859 | 2859 | MN769858 | De novo | DNA library |
| 21184 | 3/13/2015 | 135_D | L002-r49 | TTMV | Anelloviridae | 98.48 | 1747 | 52456 | 2784 | 2827 | MN771604 | De novo | DNA library |
| 21191 | 3/20/2015 | 50_D | L005-r46 | TTMV | Anelloviridae | 79.37 | 6 | 170 | 2312 | 2913 | EF538882 | Virosaurus | DNA library |
| 21195 | 12/11/2015 | 60_D | L008-r46 | TTMV | Anelloviridae | 54.53 | 8 | 170 | 1595 | 2925 | KP343850 | Virosaurus | DNA library |
| 21200 | 3/20/2015 | 61_D | L001-r46 | TTMV | Anelloviridae | 59.52 | 2 | 45 | 1735 | 2915 | NC_025726 | Virosaurus | DNA library |
| 21201 | 3/23/2015 | 67_D | L008-r46 | TTMV | Anelloviridae | 86.56 | 116 | 3829 | 2544 | 2939 | KX810064 | Virosaurus | DNA library |
| 21220 | 3/30/2015 | 213_D | L002-r57 | TTMV | Anelloviridae | 50.64 | 3 | 76 | 1417 | 2798 | EF538883 | Virosaurus | DNA library |
| 21234 | 4/8/2015 | 214_D | L003-r57 | TTMV | Anelloviridae | 13.94 | 30 | 560 | 406 | 2913 | NC_020498 | Virosaurus | DNA library |
| 21247 | 4/10/2015 | 215_D | L008-r67 | TTMV | Anelloviridae | 22.56 | 2 | 11 | 624 | 2766 | NC_014086 | Virosaurus | DNA library |
| 21253 | 4/13/2015 | 216_D | L005-r57 | TTMV | Anelloviridae | 97.94 | 117 | 5006 | 2853 | 2913 | NC_020498 | Virosaurus | DNA library |
| 21258 | 4/15/2015 | 217_D | L006-r55 | TTMV | Anelloviridae | 99.14 | 505 | 18791 | 2888 | 2913 | NC_020498 | Virosaurus | DNA library |
| 21259 | 4/15/2015 | 218_D | L007-r55 | TTMV | Anelloviridae | 94.79 | 29 | 855 | 2763 | 2915 | NC_025726 | Virosaurus | DNA library |
| 21262 | 4/15/2015 | 219_R | L006-r690 | TTMV | Anelloviridae | 23.42 | 4 | 30 | 683 | 2916 | MN770285 | De novo | RNA library |
| 21262 | 4/15/2015 | 219_D | L008-r55 | TTMV | Anelloviridae | 22.62 | 1 | 15 | 638 | 2821 | KP343863 | Virosaurus | DNA library |
| 21272 | 4/20/2015 | 220_D | L001-r57 | TTMV | Anelloviridae | 41.5 | 13 | 169 | 1209 | 2913 | EF538882 | Virosaurus | DNA library |
| 21277 | 4/20/2015 | 221_D | L003-r57 | TTMV | Anelloviridae | 29.7 | 2 | 14 | 701 | 2360 | KP343852 | Virosaurus | DNA library |
| 21280 | 4/21/2015 | 222_D | L004-r57 | TTMV | Anelloviridae | 42.53 | 4 | 70 | 1239 | 2913 | EF538882 | Virosaurus | DNA library |
| 21282 | 4/21/2015 | 797_D | L8-r111 | TTMV | Anelloviridae | 85.26 | 129 | 4437 | 2435 | 2856 | NC_025727 | Virosaurus | DNA library |
| 21291 | 4/27/2015 | 223_D | L005-r57 | TTMV | Anelloviridae | 99.42 | 6425 | 329026 | 2896 | 2913 | NC_020498 | Virosaurus | DNA library |
| 21291 | 4/27/2015 | 223_R | L007-r690 | TTMV | Anelloviridae | 24.03 | 1 | 10 | 700 | 2913 | NC_020498 | Virosaurus | RNA library |
| 21294 | 5/5/2015 | 224_R | L007-r690 | TTMV | Anelloviridae | 10.73 | 7 | 26 | 312 | 2908 | MN771081 | De novo | RNA library |
| 21294 | 5/5/2015 | 224_D | L006-r55 | TTMV | Anelloviridae | 50.43 | 2 | 332 | 1469 | 2913 | NC_020498 | Virosaurus | DNA library |
| 21297 | 5/5/2015 | 225_D | L007-r55 | TTMV | Anelloviridae | 15.35 | 11 | 75 | 457 | 2978 | KP343854 | Virosaurus | DNA library |
| 21303 | 5/6/2015 | 226_D | L001-r61 | TTMV | Anelloviridae | 98.04 | 3671 | 106837 | 2955 | 3014 | MN771139 | De novo | DNA library |
| 21323 | 5/7/2015 | 227_D | L002-r61 | TTMV | Anelloviridae | 14.54 | 5 | 97 | 433 | 2978 | KP343854 | Virosaurus | DNA library |
| 21332 | 5/11/2015 | 228_D | L003-r61 | TTMV | Anelloviridae | 27.29 | 2 | 21 | 771 | 2825 | KU243129 | Virosaurus | DNA library |
| 21333 | 5/11/2015 | 229_D | L004-r61 | TTMV | Anelloviridae | 10.21 | 44 | 165 | 304 | 2978 | KP343854 | Virosaurus | DNA library |
| 21339 | 5/12/2015 | 230_D | L005-r61 | TTMV | Anelloviridae | 14.54 | 200 | 2408 | 433 | 2978 | KP343854 | Virosaurus | DNA library |
| 21344 | 5/12/2015 | 231_D | L006-r61 | TTMV | Anelloviridae | 100 | 3624 | 105602 | 2893 | 2893 | MN770966 | De novo | DNA library |
| 21346 | 5/13/2015 | 856_D | L8-r124 | TTMV | Anelloviridae | 97.42 | 4 | 225 | 2912 | 2989 | KP343818 | Virosaurus | DNA library |
| 21349 | 5/13/2015 | 232_D | L007-r61 | TTMV | Anelloviridae | 85.06 | 23 | 751 | 2500 | 2939 | KX810064 | Virosaurus | DNA library |
| 21350 | 5/13/2015 | 233_D | L008-r61 | TTMV | Anelloviridae | 85.34 | 42 | 1276 | 2508 | 2939 | KX810064 | Virosaurus | DNA library |
| 21353 | 5/13/2015 | 234_D | L002-r61 | TTMV | Anelloviridae | 63.19 | 4 | 153 | 1785 | 2825 | KU243129 | Virosaurus | DNA library |
| 21360 | 5/13/2015 | 235_D | L003-r61 | TTMV | Anelloviridae | 100 | 26 | 1502 | 2831 | 2831 | KX810063 | Virosaurus | DNA library |
| 21362 | 5/14/2015 | 236_D | L004-r61 | TTMV | Anelloviridae | 97.51 | 1491 | 42955 | 2861 | 2934 | MN769864 | De novo | DNA library |
| 21368 | 5/14/2015 | 237_D | L005-r61 | TTMV | Anelloviridae | 34.35 | 4 | 47 | 950 | 2766 | NC_014086 | Virosaurus | DNA library |
| 21370 | 5/20/2015 | 590_D | L3-r89 | TTMV | Anelloviridae | 72.76 | 4 | 150 | 2062 | 2834 | KP343848 | Virosaurus | DNA library |
| 21377 | 5/18/2015 | 238_D | L006-r61 | TTMV | Anelloviridae | 35.98 | 4 | 75 | 1015 | 2821 | KP343863 | Virosaurus | DNA library |
| 21382 | 5/18/2015 | 239_D | L007-r61 | TTMV | Anelloviridae | 93.56 | 16 | 559 | 2730 | 2918 | KF545583 | Virosaurus | DNA library |
| 21384 | 5/18/2015 | 240_D | L008-r61 | TTMV | Anelloviridae | 42.43 | 8 | 131 | 1236 | 2913 | EF538882 | Virosaurus | DNA library |
| 21404 | 5/20/2015 | 241_D | L001-r61 | TTMV | Anelloviridae | 78.2 | 2 | 75 | 2403 | 3073 | KF545582 | Virosaurus | DNA library |
| 21405 | 5/20/2015 | 242_R | L006-r691 | TTMV | Anelloviridae | 11.94 | 2 | 18 | 326 | 2730 | MN768951 | De novo | RNA library |
| 21405 | 5/20/2015 | 242_D | L003-r61 | TTMV | Anelloviridae | 37.43 | 3 | 43 | 1116 | 2982 | KP343847 | Virosaurus | DNA library |
| 21413 | 5/21/2015 | 243_D | L004-r61 | TTMV | Anelloviridae | 11.86 | 10 | 72 | 345 | 2909 | NC_014089 | Virosaurus | DNA library |
| 21419 | 5/21/2015 | 244_R | L006-r691 | TTMV | Anelloviridae | 10.35 | 6 | 18 | 300 | 2900 | MN770367 | De novo | RNA library |
| 21419 | 5/21/2015 | 244_D | L005-r61 | TTMV | Anelloviridae | 75.86 | 76 | 1819 | 2212 | 2916 | JX134046 | Virosaurus | DNA library |
| 21420 | 5/21/2015 | 857_D | L8-r127 | TTMV | Anelloviridae | 56.81 | 3 | 919 | 1698 | 2989 | KP343818 | Virosaurus | DNA library |
| 21424 | 5/22/2015 | 245_D | L006-r61 | TTMV | Anelloviridae | 79.19 | 65 | 2064 | 2389 | 3017 | KP343864 | Virosaurus | DNA library |
| 21430 | 5/22/2015 | 246_D | L007-r61 | TTMV | Anelloviridae | 99.04 | 192 | 5374 | 2894 | 2922 | MN774049 | De novo | DNA library |
| 21433 | 5/22/2015 | 247_D | L008-r61 | TTMV | Anelloviridae | 47.9 | 7 | 185 | 1325 | 2766 | NC_014086 | Virosaurus | DNA library |
| 21437 | 5/22/2015 | 774_D | L7-r111 | TTMV | Anelloviridae | 37.35 | 4 | 46 | 1088 | 2913 | EF538882 | Virosaurus | DNA library |
| 21441 | 5/25/2015 | 248_D | L001-r61 | TTMV | Anelloviridae | 89.8 | 10 | 542 | 2616 | 2913 | NC_020498 | Virosaurus | DNA library |
| 21447 | 5/25/2015 | 249_D | L002-r61 | TTMV | Anelloviridae | 11.99 | 6 | 50 | 428 | 3571 | KF545584 | Virosaurus | DNA library |
| 21449 | 5/25/2015 | 250_D | L004-r61 | TTMV | Anelloviridae | 45.3 | 5 | 86 | 1151 | 2541 | KT163888 | Virosaurus | DNA library |
| 21453 | 5/26/2015 | 251_D | L005-r61 | TTMV | Anelloviridae | 91.25 | 10 | 301 | 2660 | 2915 | NC_025726 | Virosaurus | DNA library |
| 21461 | 6/2/2015 | 858_D | L1-r132 | TTMV | Anelloviridae | 100 | 969 | 32496 | 2989 | 2989 | KP343818 | Virosaurus | DNA library |
| 21468 | 5/27/2015 | 252_R | L008-r691 | TTMV | Anelloviridae | 11.74 | 1 | 6 | 340 | 2897 | MN769838 | De novo | RNA library |
| 21468 | 5/27/2015 | 252_D | L006-r61 | TTMV | Anelloviridae | 93.17 | 10 | 369 | 2811 | 3017 | KP343864 | Virosaurus | DNA library |
| 21469 | 5/27/2015 | 593_R | L001-r723 | TTMV | Anelloviridae | 36.92 | 4 | 60 | 1077 | 2917 | MN769583 | De novo | RNA library |
| 21469 | 5/27/2015 | 593_D | L2-r92 | TTMV | Anelloviridae | 87.28 | 169 | 4396 | 2471 | 2831 | KX810063 | Virosaurus | DNA library |
| 21471 | 5/28/2015 | 253_D | L007-r61 | TTMV | Anelloviridae | 72.8 | 2 | 56 | 2237 | 3073 | KF545582 | Virosaurus | DNA library |
| 21472 | 5/28/2015 | 254_R | L001-r692 | TTMV | Anelloviridae | 42.19 | 4 | 86 | 1253 | 2970 | MN769077 | De novo | RNA library |
| 21472 | 5/28/2015 | 254_D | L008-r61 | TTMV | Anelloviridae | 60.21 | 5 | 155 | 1701 | 2825 | KU243129 | Virosaurus | DNA library |
| 21476 | 5/28/2015 | 255_D | L001-r61 | TTMV | Anelloviridae | 10.71 | 2 | 7 | 319 | 2978 | KP343854 | Virosaurus | DNA library |
| 21477 | 5/28/2015 | 256_D | L002-r61 | TTMV | Anelloviridae | 83.09 | 51 | 1572 | 2442 | 2939 | KX810064 | Virosaurus | DNA library |
| 21478 | 5/28/2015 | 257_D | L003-r61 | TTMV | Anelloviridae | 12.26 | 4 | 22 | 365 | 2978 | KP343854 | Virosaurus | DNA library |
| 21482 | 5/28/2015 | 258_D | L005-r61 | TTMV | Anelloviridae | 95.86 | 9 | 269 | 2822 | 2944 | MN770282 | De novo | DNA library |
| 21488 | 5/29/2015 | 259_D | L006-r61 | TTMV | Anelloviridae | 56.31 | 2 | 37 | 1699 | 3017 | KP343864 | Virosaurus | DNA library |
| 21491 | 5/29/2015 | 260_D | L007-r61 | TTMV | Anelloviridae | 96.97 | 59 | 2050 | 2980 | 3073 | KF545582 | Virosaurus | DNA library |
| 21493 | 5/29/2015 | 261_D | L008-r61 | TTMV | Anelloviridae | 27.53 | 1 | 15 | 809 | 2939 | KX810064 | Virosaurus | DNA library |
| 21504 | 6/1/2015 | 262_D | L001-r61 | TTMV | Anelloviridae | 14.51 | 10 | 111 | 414 | 2854 | KF764701 | Virosaurus | DNA library |
| 21508 | 6/1/2015 | 263_D | L002-r61 | TTMV | Anelloviridae | 39.71 | 3 | 122 | 1009 | 2541 | KT163888 | Virosaurus | DNA library |
| 21513 | 6/1/2015 | 264_D | L003-r61 | TTMV | Anelloviridae | 98.31 | 157 | 5577 | 2910 | 2960 | MN769746 | De novo | DNA library |
| 21514 | 6/1/2015 | 265_D | L004-r61 | TTMV | Anelloviridae | 10.71 | 7 | 67 | 319 | 2978 | KP343854 | Virosaurus | DNA library |
| 21518 | 6/3/2015 | 266_D | L006-r61 | TTMV | Anelloviridae | 50.88 | 2 | 44 | 1535 | 3017 | KP343864 | Virosaurus | DNA library |
| 21519 | 6/3/2015 | 267_D | L007-r61 | TTMV | Anelloviridae | 36.58 | 7 | 201 | 1044 | 2854 | KF764701 | Virosaurus | DNA library |
| 21521 | 6/4/2015 | 268_D | L008-r61 | TTMV | Anelloviridae | 32.1 | 4 | 53 | 900 | 2804 | NC_030297 | Virosaurus | DNA library |
| 21523 | 6/4/2015 | 269_D | L001-r61 | TTMV | Anelloviridae | 99.97 | 2073 | 62345 | 2957 | 2958 | MN770359 | De novo | DNA library |
| 21526 | 6/4/2015 | 270_D | L002-r61 | TTMV | Anelloviridae | 12.16 | 3 | 34 | 362 | 2978 | KP343854 | Virosaurus | DNA library |
| 21528 | 6/5/2015 | 271_D | L1-r65 | TTMV | Anelloviridae | 99.83 | 231 | 6761 | 2915 | 2920 | MN771089 | De novo | DNA library |
| 21533 | 6/5/2015 | 272_D | L2-r65 | TTMV | Anelloviridae | 41.42 | 5 | 65 | 1235 | 2982 | KP343847 | Virosaurus | DNA library |
| 21534 | 6/5/2015 | 273_D | L3-r65 | TTMV | Anelloviridae | 99.29 | 227 | 7721 | 2942 | 2963 | MN769311 | De novo | DNA library |
| 21542 | 6/8/2015 | 274_D | L4-r65 | TTMV | Anelloviridae | 88.75 | 32 | 948 | 2643 | 2978 | KP343854 | Virosaurus | DNA library |
| 21548 | 6/9/2015 | 275_D | L5-r65 | TTMV | Anelloviridae | 91.25 | 47 | 1329 | 2586 | 2834 | KP343848 | Virosaurus | DNA library |
| 21556 | 6/10/2015 | 276_D | L6-r65 | TTMV | Anelloviridae | 37.93 | 4 | 55 | 1105 | 2913 | EF538882 | Virosaurus | DNA library |
| 21557 | 6/11/2015 | 595_D | L1-r89 | TTMV | Anelloviridae | 96.15 | 45 | 1177 | 2725 | 2834 | KP343848 | Virosaurus | DNA library |
| 21562 | 6/11/2015 | 277_D | L7-r65 | TTMV | Anelloviridae | 65.77 | 4 | 84 | 1864 | 2834 | KP343848 | Virosaurus | DNA library |
| 21567 | 6/12/2015 | 278_D | L007-r67 | TTMV | Anelloviridae | 95.73 | 1547 | 45695 | 2911 | 3041 | MN771643 | De novo | DNA library |
| 21568 | 6/12/2015 | 279_D | L2-r65 | TTMV | Anelloviridae | 34.03 | 34 | 568 | 960 | 2821 | KP343863 | Virosaurus | DNA library |
| 21571 | 6/15/2015 | 280_D | L3-r65 | TTMV | Anelloviridae | 16.15 | 7 | 168 | 481 | 2978 | KP343854 | Virosaurus | DNA library |
| 21573 | 6/15/2015 | 281_D | L4-r65 | TTMV | Anelloviridae | 91.99 | 13 | 378 | 2607 | 2834 | KP343848 | Virosaurus | DNA library |
| 21574 | 6/15/2015 | 282_D | L5-r65 | TTMV | Anelloviridae | 87.18 | 5 | 140 | 2489 | 2855 | MN772360 | De novo | DNA library |
| 21584 | 6/17/2015 | 283_D | L6-r65 | TTMV | Anelloviridae | 43.05 | 1 | 17 | 1255 | 2915 | NC_025726 | Virosaurus | DNA library |
| 21591 | 6/17/2015 | 596_D | L2-r89 | TTMV | Anelloviridae | 98.69 | 2318 | 63694 | 2931 | 2970 | MN769310 | De novo | DNA library |
| 21601 | 6/18/2015 | 284_D | L7-r65 | TTMV | Anelloviridae | 44.14 | 2 | 89 | 1287 | 2916 | JX134046 | Virosaurus | DNA library |
| 21602 | 6/19/2015 | 285_D | L007-r67 | TTMV | Anelloviridae | 98.24 | 310 | 9319 | 2848 | 2899 | MN770502 | De novo | DNA library |
| 21606 | 6/22/2015 | 598_D | L4-r89 | TTMV | Anelloviridae | 100 | 6022 | 177978 | 2919 | 2919 | MN769524 | De novo | DNA library |
| 21607 | 6/22/2015 | 286_D | L1-r65 | TTMV | Anelloviridae | 94.61 | 18 | 621 | 2721 | 2876 | MN771627 | De novo | DNA library |
| 21609 | 6/22/2015 | 287_D | L007-r67 | TTMV | Anelloviridae | 99.16 | 13106 | 370409 | 2938 | 2963 | MN771228 | De novo | DNA library |
| 21610 | 6/22/2015 | 288_D | L4-r65 | TTMV | Anelloviridae | 34.87 | 2 | 41 | 1020 | 2925 | KP343850 | Virosaurus | DNA library |
| 21622 | 6/24/2015 | 289_D | L5-r65 | TTMV | Anelloviridae | 86.94 | 28 | 867 | 2464 | 2834 | KP343848 | Virosaurus | DNA library |
| 21627 | 6/24/2015 | 602_R | L004-r723 | TTMV | Anelloviridae | 18.84 | 4 | 37 | 546 | 2898 | MN769442 | De novo | RNA library |
| 21627 | 6/24/2015 | 602_D | L1-r89 | TTMV | Anelloviridae | 62.67 | 2 | 44 | 1810 | 2888 | EF538880 | Virosaurus | DNA library |
| 21644 | 6/29/2015 | 611_D | L3-r89 | TTMV | Anelloviridae | 60.08 | 3 | 64 | 1750 | 2913 | EF538882 | Virosaurus | DNA library |
| 21648 | 6/29/2015 | 291_D | L7-r65 | TTMV | Anelloviridae | 10 | 2 | 35 | 357 | 3571 | KF545584 | Virosaurus | DNA library |
| 21649 | 6/29/2015 | 292_D | L8-r65 | TTMV | Anelloviridae | 86.52 | 10 | 309 | 2483 | 2870 | MN769870 | De novo | DNA library |
| 21656 | 7/1/2015 | 294_D | L2-r65 | TTMV | Anelloviridae | 13.46 | 6 | 143 | 384 | 2854 | KF764701 | Virosaurus | DNA library |
| 21661 | 7/1/2015 | 614_D | L2-r92 | TTMV | Anelloviridae | 82.8 | 94 | 2290 | 2450 | 2959 | KT163898 | Virosaurus | DNA library |
| 21664 | 7/1/2015 | 615_R | L007-r723 | TTMV | Anelloviridae | 52.2 | 7 | 190 | 1425 | 2730 | MN768951 | De novo | RNA library |
| 21664 | 7/1/2015 | 615_D | L7-r88 | TTMV | Anelloviridae | 61.62 | 1 | 1351 | 1859 | 3017 | KP343864 | Virosaurus | DNA library |
| 21668 | 7/3/2015 | 616_D | L8-r88 | TTMV | Anelloviridae | 65.6 | 2 | 128 | 1928 | 2939 | KX810064 | Virosaurus | DNA library |
| 21674 | 7/3/2015 | 290_D | L6-r65 | TTMV | Anelloviridae | 10.78 | 1 | 10 | 385 | 3571 | KF545584 | Virosaurus | DNA library |
| 21675 | 7/3/2015 | 295_D | L4-r65 | TTMV | Anelloviridae | 86.8 | 41 | 1044 | 2460 | 2834 | KP343848 | Virosaurus | DNA library |
| 21676 | 7/3/2015 | 296_D | L5-r65 | TTMV | Anelloviridae | 13.57 | 16 | 69 | 404 | 2978 | KP343854 | Virosaurus | DNA library |
| 21691 | 7/8/2015 | 297_D | L6-r65 | TTMV | Anelloviridae | 25.49 | 1 | 14 | 732 | 2872 | EF538881 | Virosaurus | DNA library |
| 21701 | 7/9/2015 | 298_D | L7-r65 | TTMV | Anelloviridae | 96.83 | 757 | 22837 | 2900 | 2995 | MN769201 | De novo | DNA library |
| 21703 | 7/9/2015 | 617_D | L1-r89 | TTMV | Anelloviridae | 99.24 | 250 | 7898 | 2886 | 2908 | MN770776 | De novo | DNA library |
| 21707 | 7/9/2015 | 299_D | L007-r67 | TTMV | Anelloviridae | 99.93 | 565 | 16852 | 2929 | 2931 | MN769159 | De novo | DNA library |
| 21710 | 7/9/2015 | 300_D | L1-r65 | TTMV | Anelloviridae | 99.66 | 106 | 3229 | 2918 | 2928 | MN769983 | De novo | DNA library |
| 21712 | 7/9/2015 | 301_D | L2-r65 | TTMV | Anelloviridae | 82.56 | 118 | 3446 | 2358 | 2856 | NC_025727 | Virosaurus | DNA library |
| 21713 | 7/9/2015 | 302_D | L3-r65 | TTMV | Anelloviridae | 39.28 | 8 | 121 | 998 | 2541 | KT163888 | Virosaurus | DNA library |
| 21715 | 7/10/2015 | 303_D | L5-r65 | TTMV | Anelloviridae | 23.82 | 10 | 91 | 693 | 2909 | NC_014089 | Virosaurus | DNA library |
| 21718 | 7/10/2015 | 304_D | L6-r65 | TTMV | Anelloviridae | 37.96 | 2 | 53 | 1062 | 2798 | EF538883 | Virosaurus | DNA library |
| 21721 | 7/10/2015 | 305_D | L7-r65 | TTMV | Anelloviridae | 98.88 | 1033 | 28747 | 2919 | 2952 | MN770862 | De novo | DNA library |
| 21722 | 7/10/2015 | 306_D | L007-r67 | TTMV | Anelloviridae | 99.35 | 1638 | 59429 | 2894 | 2913 | NC_020498 | Virosaurus | DNA library |
| 21737 | 7/14/2015 | 307_D | L1-r65 | TTMV | Anelloviridae | 100 | 2815 | 79636 | 2947 | 2947 | MN770562 | De novo | DNA library |
| 21751 | 7/16/2015 | 308_D | L2-r65 | TTMV | Anelloviridae | 100 | 171 | 5070 | 3020 | 3020 | MN770161 | De novo | DNA library |
| 21755 | 7/16/2015 | 619_D | L4-r89 | TTMV | Anelloviridae | 13.16 | 14 | 61 | 392 | 2978 | KP343854 | Virosaurus | DNA library |
| 21758 | 7/21/2015 | 624_R | L1-r724 | TTMV | Anelloviridae | 15.84 | 4 | 20 | 455 | 2872 | MN770239 | De novo | RNA library |
| 21758 | 7/21/2015 | 624_D | L1-r89 | TTMV | Anelloviridae | 43.26 | 4 | 84 | 1290 | 2982 | KP343847 | Virosaurus | DNA library |
| 21759 | 7/21/2015 | 309_D | L3-r65 | TTMV | Anelloviridae | 97.2 | 163 | 5828 | 2987 | 3073 | KF545582 | Virosaurus | DNA library |
| 21761 | 7/21/2015 | 623_R | L1-r724 | TTMV | Anelloviridae | 12.18 | 6 | 23 | 356 | 2924 | MN770144 | De novo | RNA library |
| 21761 | 7/21/2015 | 623_D | L8-r88 | TTMV | Anelloviridae | 86.73 | 95 | 2639 | 2549 | 2939 | KX810064 | Virosaurus | DNA library |
| 21762 | 7/21/2015 | 310_D | L4-r65 | TTMV | Anelloviridae | 10.57 | 1 | 16 | 316 | 2989 | KP343818 | Virosaurus | DNA library |
| 21766 | 7/21/2015 | 621_R | L008-r723 | TTMV | Anelloviridae | 43.2 | 17 | 492 | 1252 | 2898 | MN769442 | De novo | RNA library |
| 21766 | 7/21/2015 | 621_D | L2-r92 | TTMV | Anelloviridae | 58.54 | 14 | 461 | 1703 | 2909 | NC_014089 | Virosaurus | DNA library |
| 21770 | 7/21/2015 | 625_R | L1-r724 | TTMV | Anelloviridae | 15.8 | 6 | 22 | 394 | 2493 | MN773307 | De novo | RNA library |
| 21770 | 7/21/2015 | 625_D | L2-r89 | TTMV | Anelloviridae | 48.78 | 3 | 100 | 1421 | 2913 | EF538882 | Virosaurus | DNA library |
| 21771 | 7/21/2015 | 311_R | L008-r693 | TTMV | Anelloviridae | 10.62 | 1 | 20 | 319 | 3004 | MN771238 | De novo | RNA library |
| 21771 | 7/21/2015 | 311_D | L6-r65 | TTMV | Anelloviridae | 24.79 | 3 | 34 | 695 | 2804 | NC_030297 | Virosaurus | DNA library |
| 21780 | 7/24/2015 | 626_R | L2-r724 | TTMV | Anelloviridae | 80.49 | 19 | 702 | 2327 | 2891 | MN771179 | De novo | RNA library |
| 21780 | 7/24/2015 | 626_D | L4-r89 | TTMV | Anelloviridae | 96.91 | 59 | 1899 | 2978 | 3073 | KF545582 | Virosaurus | DNA library |
| 21789 | 7/23/2015 | 627_D | L1-r92 | TTMV | Anelloviridae | 23.69 | 3 | 16 | 690 | 2913 | EF538882 | Virosaurus | DNA library |
| 21801 | 7/24/2015 | 312_D | L7-r65 | TTMV | Anelloviridae | 47.92 | 3 | 290 | 1394 | 2909 | NC_014089 | Virosaurus | DNA library |
| 21812 | 7/28/2015 | 313_D | L007-r67 | TTMV | Anelloviridae | 99.53 | 262 | 8451 | 2954 | 2968 | MN769112 | De novo | DNA library |
| 21816 | 7/28/2015 | 632_D | L2-r91 | TTMV | Anelloviridae | 92.14 | 16 | 630 | 2744 | 2978 | KP343854 | Virosaurus | DNA library |
| 21818 | 7/30/2015 | 633_D | L3-r91 | TTMV | Anelloviridae | 40.64 | 6 | 83 | 1137 | 2798 | EF538883 | Virosaurus | DNA library |
| 21819 | 7/30/2015 | 314_D | L1-r65 | TTMV | Anelloviridae | 95.82 | 2311 | 66635 | 2794 | 2916 | JX134046 | Virosaurus | DNA library |
| 21819 | 7/30/2015 | 314_R | L008-r693 | TTMV | Anelloviridae | 13.82 | 1 | 7 | 403 | 2916 | JX134046 | Virosaurus | RNA library |
| 21837 | 8/3/2015 | 315_D | L2-r65 | TTMV | Anelloviridae | 36.66 | 3 | 41 | 1068 | 2913 | EF538882 | Virosaurus | DNA library |
| 21838 | 8/3/2015 | 316_R | L002-r698 | TTMV | Anelloviridae | 17.65 | 4 | 22 | 482 | 2731 | MN770251 | De novo | RNA library |
| 21838 | 8/3/2015 | 316_D | L1-r66 | TTMV | Anelloviridae | 55.77 | 3 | 114 | 1647 | 2953 | NC_014082 | Virosaurus | DNA library |
| 21839 | 8/3/2015 | 317_D | L2-r66 | TTMV | Anelloviridae | 98.88 | 370 | 10348 | 2919 | 2952 | MN770862 | De novo | DNA library |
| 21843 | 8/3/2015 | 318_D | L3-r66 | TTMV | Anelloviridae | 86.53 | 4 | 143 | 2543 | 2939 | KX810064 | Virosaurus | DNA library |
| 21849 | 8/4/2015 | 319_R | L002-r698 | TTMV | Anelloviridae | 14.27 | 2 | 7 | 369 | 2586 | MN770567 | De novo | RNA library |
| 21849 | 8/4/2015 | 319_D | L4-r66 | TTMV | Anelloviridae | 92.42 | 8 | 278 | 2694 | 2915 | NC_025726 | Virosaurus | DNA library |
| 21851 | 8/4/2015 | 320_D | L5-r66 | TTMV | Anelloviridae | 97.14 | 18 | 643 | 2887 | 2972 | MN771177 | De novo | DNA library |
| 21858 | 8/5/2015 | 321_D | L6-r66 | TTMV | Anelloviridae | 99.53 | 3009 | 85964 | 2975 | 2989 | MN770908 | De novo | DNA library |
| 21871 | 8/6/2015 | 323_R | L003-r698 | TTMV | Anelloviridae | 14.05 | 4 | 14 | 412 | 2933 | MN769192 | De novo | RNA library |
| 21871 | 8/6/2015 | 323_D | L8-r66 | TTMV | Anelloviridae | 92.42 | 7 | 228 | 2669 | 2888 | EF538880 | Virosaurus | DNA library |
| 21875 | 8/6/2015 | 639_R | L6-r724 | TTMV | Anelloviridae | 14.64 | 3 | 13 | 420 | 2869 | MN771376 | De novo | RNA library |
| 21875 | 8/6/2015 | 639_D | L2-r91 | TTMV | Anelloviridae | 81.65 | 19 | 603 | 2332 | 2856 | NC_025727 | Virosaurus | DNA library |
| 21877 | 8/7/2015 | 640_R | L6-r724 | TTMV | Anelloviridae | 19.31 | 8 | 55 | 562 | 2910 | MN769372 | De novo | RNA library |
| 21877 | 8/7/2015 | 640_D | L3-r91 | TTMV | Anelloviridae | 54.27 | 51 | 1018 | 1581 | 2913 | EF538882 | Virosaurus | DNA library |
| 21879 | 8/7/2015 | 324_D | L2-r66 | TTMV | Anelloviridae | 99.79 | 2039 | 59754 | 2873 | 2879 | MN768666 | De novo | DNA library |
| 21884 | 8/7/2015 | 325_D | L3-r66 | TTMV | Anelloviridae | 56.88 | 3 | 64 | 1460 | 2567 | KF545580 | Virosaurus | DNA library |
| 21894 | 8/14/2015 | 779_D | L7-r113 | TTMV | Anelloviridae | 72.27 | 6 | 144 | 2064 | 2856 | NC_025727 | Virosaurus | DNA library |
| 21902 | 8/10/2015 | 326_R | L004-r698 | TTMV | Anelloviridae | 17.42 | 2 | 12 | 522 | 2996 | MN771181 | De novo | RNA library |
| 21902 | 8/10/2015 | 326_D | L4-r66 | TTMV | Anelloviridae | 84.42 | 92 | 2824 | 2411 | 2856 | NC_025727 | Virosaurus | DNA library |
| 21903 | 8/10/2015 | 327_D | L5-r66 | TTMV | Anelloviridae | 100 | 2024 | 55554 | 2935 | 2935 | MN770849 | De novo | DNA library |
| 21911 | 8/13/2015 | 322_D | L7-r66 | TTMV | Anelloviridae | 97.83 | 248 | 7222 | 2881 | 2945 | MN769191 | De novo | DNA library |
| 21920 | 8/14/2015 | 328_D | L6-r66 | TTMV | Anelloviridae | 96.29 | 16 | 471 | 2825 | 2934 | MN769188 | De novo | DNA library |
| 21926 | 8/14/2015 | 329_D | L7-r66 | TTMV | Anelloviridae | 99.32 | 74 | 2152 | 2902 | 2922 | MN769900 | De novo | DNA library |
| 21937 | 8/18/2015 | 330_R | L005-r698 | TTMV | Anelloviridae | 39.99 | 8 | 89 | 1164 | 2911 | MN770543 | De novo | RNA library |
| 21937 | 8/18/2015 | 330_D | L8-r66 | TTMV | Anelloviridae | 31.69 | 2 | 31 | 923 | 2913 | EF538882 | Virosaurus | DNA library |
| 21938 | 8/18/2015 | 331_R | L005-r698 | TTMV | Anelloviridae | 58.61 | 14 | 279 | 1706 | 2911 | MN770543 | De novo | RNA library |
| 21938 | 8/18/2015 | 331_D | L1-r66 | TTMV | Anelloviridae | 41.2 | 7 | 98 | 1200 | 2913 | EF538882 | Virosaurus | DNA library |
| 21948 | 8/19/2015 | 332_D | L3-r66 | TTMV | Anelloviridae | 27.84 | 2 | 113 | 804 | 2888 | EF538880 | Virosaurus | DNA library |
| 21949 | 8/19/2015 | 333_D | L4-r66 | TTMV | Anelloviridae | 51.95 | 8 | 176 | 1549 | 2982 | KP343847 | Virosaurus | DNA library |
| 21973 | 8/21/2015 | 334_D | L5-r66 | TTMV | Anelloviridae | 82.5 | 29 | 727 | 2338 | 2834 | KP343848 | Virosaurus | DNA library |
| 21975 | 8/24/2015 | 335_D | L6-r66 | TTMV | Anelloviridae | 99.9 | 89 | 2631 | 2897 | 2900 | MN770928 | De novo | DNA library |
| 21975 | 8/24/2015 | 335_R | L006-r698 | TTMV | Anelloviridae | 11.36 | 5 | 22 | 326 | 2869 | MN771968 | De novo | RNA library |
| 21982 | 8/24/2015 | 336_D | L7-r66 | TTMV | Anelloviridae | 99.7 | 2087 | 60954 | 2943 | 2952 | MN769998 | De novo | DNA library |
| 21987 | 8/25/2015 | 337_D | L8-r66 | TTMV | Anelloviridae | 11.62 | 20 | 145 | 346 | 2978 | KP343854 | Virosaurus | DNA library |
| 21988 | 8/25/2015 | 338_R | L007-r698 | TTMV | Anelloviridae | 21.48 | 4 | 25 | 640 | 2979 | MN769209 | De novo | RNA library |
| 21988 | 8/25/2015 | 338_D | L1-r66 | TTMV | Anelloviridae | 37.27 | 1 | 82 | 1055 | 2831 | KX810063 | Virosaurus | DNA library |
| 22001 | 8/26/2015 | 339_D | L2-r66 | TTMV | Anelloviridae | 96.65 | 14 | 525 | 2970 | 3073 | KF545582 | Virosaurus | DNA library |
| 22013 | 8/27/2015 | 340_D | L4-r66 | TTMV | Anelloviridae | 37.88 | 3 | 51 | 1088 | 2872 | EF538881 | Virosaurus | DNA library |
| 22038 | 9/1/2015 | 341_R | L008-r698 | TTMV | Anelloviridae | 66.82 | 12 | 400 | 1986 | 2972 | MN771177 | De novo | RNA library |
| 22038 | 9/1/2015 | 341_D | L5-r66 | TTMV | Anelloviridae | 97.02 | 564 | 17778 | 2828 | 2915 | NC_025726 | Virosaurus | DNA library |
| 22041 | 9/1/2015 | 342_D | L6-r66 | TTMV | Anelloviridae | 99.97 | 4762 | 130180 | 2895 | 2896 | MN770554 | De novo | DNA library |
| 22042 | 9/1/2015 | 650_D | L6-r91 | TTMV | Anelloviridae | 97.83 | 5225 | 148838 | 2923 | 2988 | MN770490 | De novo | DNA library |
| 22049 | 9/2/2015 | 343_D | L7-r66 | TTMV | Anelloviridae | 99.58 | 190 | 5176 | 2617 | 2628 | MN773209 | De novo | DNA library |
| 22055 | 9/2/2015 | 344_D | L8-r66 | TTMV | Anelloviridae | 92.58 | 22 | 620 | 2721 | 2939 | KX810064 | Virosaurus | DNA library |
| 22062 | 9/3/2015 | 345_R | L001-r702 | TTMV | Anelloviridae | 12.99 | 2 | 10 | 367 | 2825 | MN769651 | De novo | RNA library |
| 22062 | 9/3/2015 | 345_D | L1-r66 | TTMV | Anelloviridae | 13.3 | 25 | 417 | 396 | 2978 | KP343854 | Virosaurus | DNA library |
| 22067 | 9/3/2015 | 346_D | L2-r66 | TTMV | Anelloviridae | 76.99 | 4 | 160 | 2175 | 2825 | KU243129 | Virosaurus | DNA library |
| 22068 | 9/3/2015 | 347_D | L3-r66 | TTMV | Anelloviridae | 86.46 | 5 | 189 | 2497 | 2888 | EF538880 | Virosaurus | DNA library |
| 22071 | 9/3/2015 | 348_D | L5-r66 | TTMV | Anelloviridae | 72.73 | 11 | 241 | 1867 | 2567 | KF545580 | Virosaurus | DNA library |
| 22079 | 9/7/2015 | 349_D | L6-r66 | TTMV | Anelloviridae | 100 | 34909 | 1120647 | 2913 | 2913 | NC_020498 | Virosaurus | DNA library |
| 22079 | 9/7/2015 | 349_R | L002-r702 | TTMV | Anelloviridae | 41.57 | 2 | 30 | 1211 | 2913 | NC_020498 | Virosaurus | RNA library |
| 22084 | 9/7/2015 | 350_D | L7-r66 | TTMV | Anelloviridae | 81.86 | 52 | 1484 | 2338 | 2856 | NC_025727 | Virosaurus | DNA library |
| 22087 | 9/7/2015 | 351_R | L002-r702 | TTMV | Anelloviridae | 16.78 | 6 | 38 | 474 | 2825 | MN770671 | De novo | RNA library |
| 22087 | 9/7/2015 | 351_D | L8-r66 | TTMV | Anelloviridae | 60 | 12 | 260 | 1755 | 2925 | KP343850 | Virosaurus | DNA library |
| 22093 | 9/8/2015 | 859_D | L2-r132 | TTMV | Anelloviridae | 81.79 | 54 | 1613 | 2336 | 2856 | NC_025727 | Virosaurus | DNA library |
| 22095 | 9/8/2015 | 860_D | L3-r132 | TTMV | Anelloviridae | 39.21 | 2 | 62 | 1158 | 2953 | NC_014082 | Virosaurus | DNA library |
| 22107 | 9/9/2015 | 352_R | L003-r702 | TTMV | Anelloviridae | 10.77 | 3 | 18 | 311 | 2888 | MN771837 | De novo | RNA library |
| 22107 | 9/9/2015 | 352_D | L1-r66 | TTMV | Anelloviridae | 38.83 | 2 | 42 | 1131 | 2913 | EF538882 | Virosaurus | DNA library |
| 22108 | 9/9/2015 | 353_R | L003-r702 | TTMV | Anelloviridae | 19.93 | 3 | 18 | 578 | 2900 | MN769244 | De novo | RNA library |
| 22108 | 9/9/2015 | 353_D | L2-r66 | TTMV | Anelloviridae | 100 | 314 | 9512 | 2831 | 2831 | KX810063 | Virosaurus | DNA library |
| 22110 | 9/9/2015 | 354_D | L3-r66 | TTMV | Anelloviridae | 93.52 | 14 | 391 | 2726 | 2915 | NC_025726 | Virosaurus | DNA library |
| 22111 | 9/9/2015 | 355_R | L003-r702 | TTMV | Anelloviridae | 12.21 | 1 | 8 | 357 | 2923 | MN769832 | De novo | RNA library |
| 22111 | 9/9/2015 | 355_D | L4-r66 | TTMV | Anelloviridae | 64.27 | 2 | 56 | 1871 | 2911 | NC_014068 | Virosaurus | DNA library |
| 22117 | 9/9/2015 | 655_D | L4-r91 | TTMV | Anelloviridae | 77.33 | 39 | 967 | 2255 | 2916 | JX134046 | Virosaurus | DNA library |
| 22126 | 9/10/2015 | 356_D | L6-r66 | TTMV | Anelloviridae | 99.83 | 1603 | 43836 | 2882 | 2887 | MN771042 | De novo | DNA library |
| 22152 | 9/14/2015 | 660_D | L1-r91 | TTMV | Anelloviridae | 99.29 | 634 | 17620 | 2926 | 2947 | MN770562 | De novo | DNA library |
| 22152 | 9/14/2015 | 660_R | L3-r725 | TTMV | Anelloviridae | 10.96 | 3 | 20 | 323 | 2947 | MN770562 | De novo | RNA library |
| 22163 | 9/15/2015 | 357_D | L7-r66 | TTMV | Anelloviridae | 96.19 | 32 | 1019 | 2800 | 2911 | NC_014068 | Virosaurus | DNA library |
| 22172 | 9/16/2015 | 358_D | L8-r66 | TTMV | Anelloviridae | 25.21 | 3 | 45 | 707 | 2804 | NC_030297 | Virosaurus | DNA library |
| 22179 | 9/17/2015 | 667_R | L5-r725 | TTMV | Anelloviridae | 21.27 | 5 | 28 | 632 | 2971 | MN773568 | De novo | RNA library |
| 22179 | 9/17/2015 | 667_D | L1-r91 | TTMV | Anelloviridae | 61.79 | 2 | 71 | 1751 | 2834 | KP343848 | Virosaurus | DNA library |
| 22185 | 9/17/2015 | 359_D | L1-r66 | TTMV | Anelloviridae | 31.02 | 4 | 55 | 858 | 2766 | NC_014086 | Virosaurus | DNA library |
| 22190 | 9/18/2015 | 781_D | L6-r111 | TTMV | Anelloviridae | 69.97 | 16 | 280 | 1796 | 2567 | KF545580 | Virosaurus | DNA library |
| 22191 | 9/18/2015 | 360_D | L2-r66 | TTMV | Anelloviridae | 99.19 | 164 | 5872 | 2942 | 2966 | MN771027 | De novo | DNA library |
| 22195 | 9/18/2015 | 361_D | L001-r71 | TTMV | Anelloviridae | 40.19 | 3 | 58 | 1063 | 2645 | KP343849 | Virosaurus | DNA library |
| 22196 | 9/18/2015 | 362_D | L002-r71 | TTMV | Anelloviridae | 15.02 | 8 | 380 | 449 | 2989 | KP343818 | Virosaurus | DNA library |
| 22198 | 9/18/2015 | 363_D | L003-r71 | TTMV | Anelloviridae | 12.31 | 18 | 110 | 368 | 2989 | KP343818 | Virosaurus | DNA library |
| 22201 | 9/21/2015 | 364_D | L004-r71 | TTMV | Anelloviridae | 77.94 | 88 | 2594 | 2226 | 2856 | NC_025727 | Virosaurus | DNA library |
| 22203 | 9/21/2015 | 365_D | L001-r73 | TTMV | Anelloviridae | 55.65 | 2 | 84 | 1621 | 2913 | EF538882 | Virosaurus | DNA library |
| 22206 | 9/21/2015 | 366_D | L002-r73 | TTMV | Anelloviridae | 29 | 3 | 39 | 833 | 2872 | EF538881 | Virosaurus | DNA library |
| 22211 | 9/22/2015 | 367_R | L007-r702 | TTMV | Anelloviridae | 19.29 | 4 | 38 | 577 | 2992 | MN770170 | De novo | RNA library |
| 22211 | 9/22/2015 | 367_D | L003-r73 | TTMV | Anelloviridae | 81.28 | 158 | 4392 | 2370 | 2916 | JX134046 | Virosaurus | DNA library |
| 22212 | 9/22/2015 | 368_D | L004-r73 | TTMV | Anelloviridae | 59.8 | 4 | 102 | 1749 | 2925 | KP343850 | Virosaurus | DNA library |
| 22215 | 9/22/2015 | 369_R | L008-r702 | TTMV | Anelloviridae | 26.44 | 4 | 50 | 771 | 2916 | MN770056 | De novo | RNA library |
| 22215 | 9/22/2015 | 369_D | L002-r71 | TTMV | Anelloviridae | 80.24 | 17 | 429 | 2274 | 2834 | KP343848 | Virosaurus | DNA library |
| 22232 | 9/22/2015 | 370_D | L003-r71 | TTMV | Anelloviridae | 13.12 | 7 | 78 | 392 | 2989 | KP343818 | Virosaurus | DNA library |
| 22246 | 9/28/2015 | 371_D | L004-r71 | TTMV | Anelloviridae | 90.34 | 9 | 250 | 2599 | 2877 | MN771811 | De novo | DNA library |
| 22250 | 9/28/2015 | 372_D | L001-r73 | TTMV | Anelloviridae | 36.25 | 2 | 25 | 1056 | 2913 | EF538882 | Virosaurus | DNA library |
| 22251 | 9/28/2015 | 373_D | L002-r73 | TTMV | Anelloviridae | 98.4 | 240 | 6616 | 2883 | 2930 | MN770913 | De novo | DNA library |
| 22259 | 9/29/2015 | 374_D | L003-r73 | TTMV | Anelloviridae | 34.51 | 5 | 69 | 1000 | 2898 | NC_014088 | Virosaurus | DNA library |
| 22262 | 9/29/2015 | 375_D | L004-r73 | TTMV | Anelloviridae | 99.72 | 70 | 1922 | 2496 | 2503 | MN773556 | De novo | DNA library |
| 22274 | 10/9/2015 | 376_D | L001-r71 | TTMV | Anelloviridae | 94.96 | 137 | 4036 | 2769 | 2916 | JX134046 | Virosaurus | DNA library |
| 22276 | 10/9/2015 | 377_D | L003-r71 | TTMV | Anelloviridae | 34.06 | 4 | 52 | 942 | 2766 | NC_014086 | Virosaurus | DNA library |
| 22277 | 10/12/2015 | 782_R | L7-r737 | TTMV | Anelloviridae | 33.52 | 9 | 88 | 959 | 2861 | MN774811 | De novo | RNA library |
| 22277 | 10/12/2015 | 782_D | L8-r111 | TTMV | Anelloviridae | 97.73 | 82 | 2824 | 2847 | 2913 | NC_020498 | Virosaurus | DNA library |
| 22278 | 10/15/2015 | 783_D | L4-r113 | TTMV | Anelloviridae | 66.48 | 3 | 69 | 1884 | 2834 | KP343848 | Virosaurus | DNA library |
| 22283 | 10/19/2015 | 784_D | L5-r113 | TTMV | Anelloviridae | 74.06 | 32 | 704 | 2064 | 2787 | MN770728 | De novo | DNA library |
| 22285 | 10/15/2015 | 378_D | L004-r71 | TTMV | Anelloviridae | 58.19 | 2 | 39 | 1695 | 2913 | EF538882 | Virosaurus | DNA library |
| 22292 | 10/19/2015 | 380_D | L002-r73 | TTMV | Anelloviridae | 84.62 | 68 | 3518 | 2475 | 2925 | KP343850 | Virosaurus | DNA library |
| 22295 | 10/20/2015 | 382_D | L004-r73 | TTMV | Anelloviridae | 12.06 | 6 | 142 | 359 | 2978 | KP343854 | Virosaurus | DNA library |
| 22296 | 10/20/2015 | 383_D | L001-r71 | TTMV | Anelloviridae | 98.39 | 172 | 5173 | 2868 | 2915 | MN770590 | De novo | DNA library |
| 22297 | 10/20/2015 | 384_D | L002-r71 | TTMV | Anelloviridae | 99.93 | 47 | 1289 | 2683 | 2685 | MN772780 | De novo | DNA library |
| 22301 | 10/12/2015 | 385_D | L004-r71 | TTMV | Anelloviridae | 76.61 | 4 | 121 | 2171 | 2834 | KP343848 | Virosaurus | DNA library |
| 22305 | 10/13/2015 | 386_D | L001-r73 | TTMV | Anelloviridae | 78.79 | 3 | 73 | 2233 | 2834 | KP343848 | Virosaurus | DNA library |
| 22319 | 10/20/2015 | 387_D | L002-r73 | TTMV | Anelloviridae | 99.76 | 65 | 1858 | 2959 | 2966 | MN770750 | De novo | DNA library |
| 22321 | 10/20/2015 | 388_D | L003-r73 | TTMV | Anelloviridae | 97.05 | 156 | 4982 | 2829 | 2915 | NC_025726 | Virosaurus | DNA library |
| 22326 | 10/22/2015 | 389_D | L004-r73 | TTMV | Anelloviridae | 33.75 | 1 | 12 | 964 | 2856 | NC_025727 | Virosaurus | DNA library |
| 22346 | 11/2/2015 | 32_D | L003-r44 | TTMV | Anelloviridae | 99.97 | 3507 | 100046 | 2922 | 2923 | MN769189 | De novo | DNA library |
| 22349 | 11/2/2015 | 11_D | L004-r44 | TTMV | Anelloviridae | 76.75 | 31 | 947 | 2245 | 2925 | KP343850 | Virosaurus | DNA library |
| 22359 | 11/6/2015 | 390_D | L001-r71 | TTMV | Anelloviridae | 54.06 | 3 | 53 | 1532 | 2834 | KP343848 | Virosaurus | DNA library |
| 22382 | 11/18/2015 | 391_D | L002-r71 | TTMV | Anelloviridae | 97.04 | 35 | 1172 | 2982 | 3073 | KF545582 | Virosaurus | DNA library |
| 22383 | 11/20/2015 | 789_D | L7-r111 | TTMV | Anelloviridae | 46.44 | 8 | 192 | 1180 | 2541 | KT163888 | Virosaurus | DNA library |
| 22401 | 10/21/2015 | 392_D | L003-r71 | TTMV | Anelloviridae | 33.13 | 1 | 15 | 988 | 2982 | KP343847 | Virosaurus | DNA library |
| 22405 | 10/22/2015 | 393_D | L001-r73 | TTMV | Anelloviridae | 74.37 | 1 | 43 | 2165 | 2911 | NC_014068 | Virosaurus | DNA library |
| 22406 | 10/22/2015 | 394_D | L002-r73 | TTMV | Anelloviridae | 49.28 | 1 | 156 | 1473 | 2989 | KP343818 | Virosaurus | DNA library |
| 22408 | 10/23/2015 | 395_D | L003-r73 | TTMV | Anelloviridae | 98.17 | 91 | 2638 | 2739 | 2790 | MN774699 | De novo | DNA library |
| 22409 | 10/23/2015 | 396_D | L004-r73 | TTMV | Anelloviridae | 67.62 | 9 | 204 | 2040 | 3017 | KP343864 | Virosaurus | DNA library |
| 22411 | 10/27/2015 | 31_D | L002-r44 | TTMV | Anelloviridae | 43.62 | 1 | 143 | 1299 | 2978 | KP343854 | Virosaurus | DNA library |
| 22413 | 10/27/2015 | 37_D | L001-r44 | TTMV | Anelloviridae | 96.36 | 50 | 3204 | 3441 | 3571 | KF545584 | Virosaurus | DNA library |
| 22414 | 10/30/2015 | 45_D | L002-r44 | TTMV | Anelloviridae | 96.88 | 70 | 2065 | 2824 | 2915 | NC_025726 | Virosaurus | DNA library |
| 22415 | 10/28/2015 | 10_D | L003-r44 | TTMV | Anelloviridae | 100 | 1353 | 43918 | 2989 | 2989 | KP343818 | Virosaurus | DNA library |
| 22418 | 10/29/2015 | 668_D | L2-r91 | TTMV | Anelloviridae | 87.55 | 4 | 156 | 2617 | 2989 | KP343818 | Virosaurus | DNA library |
| 22419 | 10/29/2015 | 785_D | L6-r113 | TTMV | Anelloviridae | 93.79 | 8 | 262 | 2882 | 3073 | KF545582 | Virosaurus | DNA library |
| 22423 | 11/2/2015 | 38_D | L002-r44 | TTMV | Anelloviridae | 40.42 | 2 | 57 | 1069 | 2645 | KP343849 | Virosaurus | DNA library |
| 22424 | 11/2/2015 | 19_D | L005-r44 | TTMV | Anelloviridae | 97.27 | 787 | 29401 | 2989 | 3073 | KF545582 | Virosaurus | DNA library |
| 22428 | 11/3/2015 | 20_D | L006-r44 | TTMV | Anelloviridae | 74.31 | 38 | 948 | 1978 | 2662 | MN774220 | De novo | DNA library |
| 22431 | 11/4/2015 | 397_D | L001-r71 | TTMV | Anelloviridae | 98.78 | 109 | 3064 | 2837 | 2872 | MN769342 | De novo | DNA library |
| 22432 | 11/4/2015 | 398_D | L002-r71 | TTMV | Anelloviridae | 73.75 | 6 | 154 | 2090 | 2834 | KP343848 | Virosaurus | DNA library |
| 22435 | 11/6/2015 | 39_D | L003-r44 | TTMV | Anelloviridae | 99.97 | 36 | 1206 | 2976 | 2977 | KP343834 | Virosaurus | DNA library |
| 22436 | 11/6/2015 | 13_D | L006-r44 | TTMV | Anelloviridae | 70.27 | 10 | 238 | 1985 | 2825 | KU243129 | Virosaurus | DNA library |
| 22437 | 11/6/2015 | 399_R | L007-r703 | TTMV | Anelloviridae | 18.6 | 9 | 49 | 547 | 2941 | MN769404 | De novo | RNA library |
| 22437 | 11/6/2015 | 399_D | L003-r71 | TTMV | Anelloviridae | 38.69 | 4 | 42 | 1127 | 2913 | EF538882 | Virosaurus | DNA library |
| 22442 | 11/11/2015 | 23_D | L001-r44 | TTMV | Anelloviridae | 99.93 | 1373 | 36143 | 2872 | 2874 | MN769055 | De novo | DNA library |
| 22444 | 11/11/2015 | 29_D | L008-r44 | TTMV | Anelloviridae | 43.43 | 2 | 44 | 1295 | 2982 | KP343847 | Virosaurus | DNA library |
| 22448 | 11/12/2015 | 788_D | L6-r111 | TTMV | Anelloviridae | 41.85 | 4 | 68 | 1219 | 2913 | EF538882 | Virosaurus | DNA library |
| 22453 | 11/16/2015 | 400_R | L007-r703 | TTMV | Anelloviridae | 10.57 | 1 | 5 | 301 | 2849 | MN769347 | De novo | RNA library |
| 22453 | 11/16/2015 | 400_D | L004-r71 | TTMV | Anelloviridae | 11.08 | 7 | 45 | 330 | 2978 | KP343854 | Virosaurus | DNA library |
| 22457 | 11/16/2015 | 401_D | L002-r73 | TTMV | Anelloviridae | 100 | 12 | 473 | 2831 | 2831 | KX810063 | Virosaurus | DNA library |
| 22460 | 11/17/2015 | 16_D | L001-r44 | TTMV | Anelloviridae | 98.86 | 167 | 5125 | 2960 | 2994 | MN770458 | De novo | DNA library |
| 22461 | 11/17/2015 | 24_D | L002-r44 | TTMV | Anelloviridae | 92.61 | 20 | 575 | 2605 | 2813 | MN772120 | De novo | DNA library |
| 22462 | 11/18/2015 | 35_R | L001-r674 | TTMV | Anelloviridae | 67.61 | 2 | 96 | 1847 | 2732 | MN772004 | De novo | RNA library |
| 22462 | 11/18/2015 | 35_D | L007-r44 | TTMV | Anelloviridae | 91.54 | 38 | 1856 | 3269 | 3571 | KF545584 | Virosaurus | DNA library |
| 22463 | 11/18/2015 | 42_D | L007-r44 | TTMV | Anelloviridae | 100 | 1316 | 38010 | 2907 | 2907 | MN770800 | De novo | DNA library |
| 22473 | 11/23/2015 | 402_D | L003-r73 | TTMV | Anelloviridae | 100 | 19 | 717 | 2831 | 2831 | KX810063 | Virosaurus | DNA library |
| 22476 | 11/24/2015 | 46_D | L001-r46 | TTMV | Anelloviridae | 99.9 | 317 | 9677 | 2959 | 2962 | MN769968 | De novo | DNA library |
| 22481 | 11/24/2015 | 662_D | L3-r91 | TTMV | Anelloviridae | 24.38 | 2 | 12 | 682 | 2798 | EF538883 | Virosaurus | DNA library |
| 22482 | 11/27/2015 | 791_D | L5-r113 | TTMV | Anelloviridae | 95.95 | 28 | 947 | 2797 | 2915 | NC_025726 | Virosaurus | DNA library |
| 22484 | 11/26/2015 | 82_D | L001-r46 | TTMV | Anelloviridae | 10.57 | 10 | 36 | 308 | 2913 | NC_020498 | Virosaurus | DNA library |
| 22485 | 11/26/2015 | 53_D | L008-r46 | TTMV | Anelloviridae | 8.65 | 105 | 474 | 308 | 3559 | KP343833 | Virosaurus | DNA library |
| 22486 | 8/6/2015 | 59_D | L007-r46 | TTMV | Anelloviridae | 95.87 | 121 | 3331 | 2574 | 2685 | MN772780 | De novo | DNA library |
| 22488 | 11/26/2015 | 47_D | L002-r46 | TTMV | Anelloviridae | 36.59 | 3 | 56 | 1026 | 2804 | NC_030297 | Virosaurus | DNA library |
| 22494 | 12/1/2015 | 78_D | L005-r46 | TTMV | Anelloviridae | 60.98 | 74 | 2760 | 1774 | 2909 | NC_014089 | Virosaurus | DNA library |
| 22495 | 11/24/2015 | 403_D | L004-r73 | TTMV | Anelloviridae | 49.5 | 4 | 82 | 1476 | 2982 | KP343847 | Virosaurus | DNA library |
| 22499 | 12/2/2015 | 83_D | L002-r46 | TTMV | Anelloviridae | 57.16 | 2 | 53 | 1664 | 2911 | NC_014068 | Virosaurus | DNA library |
| 22516 | 12/2/2015 | 48_D | L003-r46 | TTMV | Anelloviridae | 95.29 | 90 | 2663 | 2894 | 3037 | MN771852 | De novo | DNA library |
| 22518 | 12/3/2015 | 792_D | L6-r113 | TTMV | Anelloviridae | 14.24 | 15 | 114 | 424 | 2978 | KP343854 | Virosaurus | DNA library |
| 22531 | 12/11/2015 | 65_D | L006-r46 | TTMV | Anelloviridae | 97.61 | 15 | 517 | 2819 | 2888 | EF538880 | Virosaurus | DNA library |
| 22601 | 12/4/2015 | 664_D | L6-r91 | TTMV | Anelloviridae | 85.79 | 50 | 1371 | 2409 | 2808 | MN771465 | De novo | DNA library |
| 22603 | 12/3/2015 | 71_D | L005-r46 | TTMV | Anelloviridae | 62.17 | 2 | 53 | 1762 | 2834 | KP343848 | Virosaurus | DNA library |
| 22605 | 12/3/2015 | 64_D | L005-r46 | TTMV | Anelloviridae | 97.77 | 147 | 5112 | 2848 | 2913 | NC_020498 | Virosaurus | DNA library |
| 22606 | 12/4/2015 | 404_D | L001-r71 | TTMV | Anelloviridae | 98.83 | 131 | 3868 | 2945 | 2980 | MN771734 | De novo | DNA library |
| 22610 | 12/7/2015 | 89_D | L001-r46 | TTMV | Anelloviridae | 96.81 | 46 | 1534 | 2820 | 2913 | NC_020498 | Virosaurus | DNA library |
| 22618 | 12/10/2015 | 49_D | L004-r46 | TTMV | Anelloviridae | 95.27 | 11 | 346 | 2777 | 2915 | NC_025726 | Virosaurus | DNA library |
| 22622 | 1/8/2016 | 72_D | L006-r46 | TTMV | Anelloviridae | 41.06 | 8 | 112 | 1160 | 2825 | KU243129 | Virosaurus | DNA library |
| 22623 | 1/7/2016 | 79_D | L006-r46 | TTMV | Anelloviridae | 31.1 | 1 | 21 | 906 | 2913 | EF538882 | Virosaurus | DNA library |
| 22624 | 1/7/2016 | 75_D | L001-r46 | TTMV | Anelloviridae | 38.93 | 2 | 36 | 1134 | 2913 | EF538882 | Virosaurus | DNA library |
| 22628 | 1/11/2016 | 793_D | L7-r113 | TTMV | Anelloviridae | 17.2 | 1 | 7 | 503 | 2925 | KP343850 | Virosaurus | DNA library |
| 22639 | 1/18/2016 | 51_D | L006-r46 | TTMV | Anelloviridae | 48.71 | 10 | 201 | 1419 | 2913 | EF538882 | Virosaurus | DNA library |
| 22644 | 1/18/2016 | 56_D | L002-r55 | TTMV | Anelloviridae | 13.94 | 4 | 47 | 415 | 2978 | KP343854 | Virosaurus | DNA library |
| 22645 | 1/21/2016 | 405_D | L002-r71 | TTMV | Anelloviridae | 100 | 3308 | 92252 | 2891 | 2891 | MN769358 | De novo | DNA library |
| 22648 | 1/19/2016 | 80_D | L007-r46 | TTMV | Anelloviridae | 84.14 | 13 | 323 | 2382 | 2831 | KX810063 | Virosaurus | DNA library |
| 22652 | 1/20/2016 | 66_D | L007-r46 | TTMV | Anelloviridae | 16.98 | 3 | 25 | 476 | 2804 | NC_030297 | Virosaurus | DNA library |
| 22653 | 1/21/2016 | 73_D | L007-r46 | TTMV | Anelloviridae | 85.74 | 48 | 1222 | 2520 | 2939 | KX810064 | Virosaurus | DNA library |
| 22654 | 1/21/2016 | 665_R | L4-r725 | TTMV | Anelloviridae | 33.45 | 15 | 160 | 981 | 2933 | MN769192 | De novo | RNA library |
| 22654 | 1/21/2016 | 665_D | L7-r91 | TTMV | Anelloviridae | 45.83 | 3 | 60 | 1335 | 2913 | EF538882 | Virosaurus | DNA library |
| 22663 | 1/27/2016 | 57_R | L007-r674 | TTMV | Anelloviridae | 13.03 | 14 | 60 | 381 | 2924 | MN770144 | De novo | RNA library |
| 22663 | 1/27/2016 | 57_D | L005-r46 | TTMV | Anelloviridae | 80.6 | 41 | 1081 | 2302 | 2856 | NC_025727 | Virosaurus | DNA library |
| 22665 | 1/27/2016 | 86_D | L006-r46 | TTMV | Anelloviridae | 95.03 | 44 | 1480 | 2773 | 2918 | KF545583 | Virosaurus | DNA library |
| 22667 | 3/20/2015 | 62_D | L003-r46 | TTMV | Anelloviridae | 98.19 | 107 | 3120 | 2933 | 2987 | MN769702 | De novo | DNA library |
| 22668 | 1/28/2016 | 85_D | L004-r46 | TTMV | Anelloviridae | 40.69 | 2 | 27 | 1034 | 2541 | KT163888 | Virosaurus | DNA library |
| 22671 | 1/29/2016 | 406_R | L002-r704 | TTMV | Anelloviridae | 28.62 | 3 | 32 | 842 | 2942 | MN771277 | De novo | RNA library |
| 22671 | 1/29/2016 | 406_D | L005-r73 | TTMV | Anelloviridae | 52.44 | 1 | 20 | 1529 | 2916 | JX134046 | Virosaurus | DNA library |
| 22672 | 2/1/2016 | 666_R | L4-r725 | TTMV | Anelloviridae | 19.16 | 13 | 59 | 557 | 2907 | MN770328 | De novo | RNA library |
| 22672 | 2/1/2016 | 666_D | L8-r91 | TTMV | Anelloviridae | 77.7 | 22 | 553 | 2195 | 2825 | KU243129 | Virosaurus | DNA library |
| 22673 | 2/1/2016 | 90_D | L002-r46 | TTMV | Anelloviridae | 78.49 | 3 | 82 | 2226 | 2836 | MN769756 | De novo | DNA library |
| 22675 | 2/2/2016 | 81_D | L008-r46 | TTMV | Anelloviridae | 48.47 | 3 | 68 | 1426 | 2942 | KP343858 | Virosaurus | DNA library |
| 22680 | 2/3/2016 | 76_D | L002-r46 | TTMV | Anelloviridae | 12.36 | 10 | 84 | 368 | 2978 | KP343854 | Virosaurus | DNA library |
| 22685 | 2/5/2016 | 58_D | L006-r46 | TTMV | Anelloviridae | 62.92 | 11 | 273 | 1783 | 2834 | KP343848 | Virosaurus | DNA library |
| 22686 | 2/9/2016 | 63_D | L004-r46 | TTMV | Anelloviridae | 98.96 | 1673 | 46382 | 2840 | 2870 | MN769870 | De novo | DNA library |
| 22689 | 2/9/2016 | 68_D | L001-r46 | TTMV | Anelloviridae | 91.35 | 10 | 324 | 2756 | 3017 | KP343864 | Virosaurus | DNA library |
| 25003 | 12/11/2014 | 407_D | L006-r73 | TTMV | Anelloviridae | 19.64 | 3 | 26 | 564 | 2872 | EF538881 | Virosaurus | DNA library |
| 25004 | 1/15/2015 | 408_D | L001-r75 | TTMV | Anelloviridae | 99.57 | 43 | 1395 | 2976 | 2989 | KP343818 | Virosaurus | DNA library |
| 25008 | 1/15/2015 | 409_D | L002-r75 | TTMV | Anelloviridae | 75.18 | 2 | 154 | 2247 | 2989 | KP343818 | Virosaurus | DNA library |
| 25015 | 1/19/2015 | 410_D | L003-r75 | TTMV | Anelloviridae | 25.54 | 3 | 27 | 744 | 2913 | KP343851 | Virosaurus | DNA library |
| 25016 | 1/19/2015 | 411_D | L004-r75 | TTMV | Anelloviridae | 79.69 | 26 | 687 | 2276 | 2856 | NC_025727 | Virosaurus | DNA library |
| 25023 | 1/20/2015 | 412_D | L005-r75 | TTMV | Anelloviridae | 99.08 | 5 | 172 | 2805 | 2831 | KX810063 | Virosaurus | DNA library |
| 25025 | 1/21/2015 | 413_D | L006-r75 | TTMV | Anelloviridae | 97.2 | 75 | 2598 | 2987 | 3073 | KF545582 | Virosaurus | DNA library |
| 25026 | 1/21/2015 | 414_D | L006-r73 | TTMV | Anelloviridae | 49.92 | 4 | 94 | 1460 | 2925 | KP343850 | Virosaurus | DNA library |
| 25029 | 1/21/2015 | 795_D | L6-r111 | TTMV | Anelloviridae | 35.15 | 1 | 24 | 1028 | 2925 | KP343850 | Virosaurus | DNA library |
| 25033 | 1/22/2015 | 415_D | L001-r75 | TTMV | Anelloviridae | 22.38 | 2 | 16 | 652 | 2913 | KP343851 | Virosaurus | DNA library |
| 25034 | 1/22/2015 | 416_D | L002-r75 | TTMV | Anelloviridae | 19.46 | 1 | 10 | 564 | 2898 | NC_014088 | Virosaurus | DNA library |
| 25037 | 1/23/2015 | 417_D | L003-r75 | TTMV | Anelloviridae | 96.79 | 15 | 429 | 2802 | 2895 | MN769940 | De novo | DNA library |
| 25055 | 1/30/2015 | 418_D | L004-r75 | TTMV | Anelloviridae | 39.41 | 1 | 21 | 1117 | 2834 | KP343848 | Virosaurus | DNA library |
| 25056 | 1/30/2015 | 419_D | L005-r75 | TTMV | Anelloviridae | 98.53 | 265 | 7581 | 2749 | 2790 | MN773692 | De novo | DNA library |
| 25081 | 2/10/2015 | 420_D | L006-r75 | TTMV | Anelloviridae | 100 | 4006 | 116956 | 2923 | 2923 | MN770193 | De novo | DNA library |
| 25083 | 2/11/2015 | 421_D | L005-r73 | TTMV | Anelloviridae | 43.64 | 5 | 93 | 1284 | 2942 | KP343858 | Virosaurus | DNA library |
| 25086 | 2/12/2015 | 423_D | L002-r75 | TTMV | Anelloviridae | 97.81 | 713 | 19543 | 2907 | 2972 | MN770770 | De novo | DNA library |
| 25089 | 2/16/2015 | 424_D | L003-r75 | TTMV | Anelloviridae | 92.99 | 18 | 578 | 2679 | 2881 | MN771106 | De novo | DNA library |
| 25090 | 2/16/2015 | 425_D | L004-r75 | TTMV | Anelloviridae | 16.96 | 33 | 262 | 505 | 2978 | KP343854 | Virosaurus | DNA library |
| 25093 | 2/16/2015 | 426_D | L005-r75 | TTMV | Anelloviridae | 17.19 | 1 | 5 | 482 | 2804 | NC_030297 | Virosaurus | DNA library |
| 25097 | 2/17/2015 | 427_D | L006-r75 | TTMV | Anelloviridae | 97.23 | 33 | 1134 | 2988 | 3073 | KF545582 | Virosaurus | DNA library |
| 25099 | 2/17/2015 | 428_D | L005-r73 | TTMV | Anelloviridae | 33.12 | 4 | 45 | 916 | 2766 | NC_014086 | Virosaurus | DNA library |
| 25103 | 2/18/2015 | 429_D | L006-r73 | TTMV | Anelloviridae | 14.55 | 23 | 158 | 435 | 2989 | KP343818 | Virosaurus | DNA library |
| 25107 | 2/18/2015 | 430_D | L002-r75 | TTMV | Anelloviridae | 92.86 | 5 | 161 | 2744 | 2955 | MN770630 | De novo | DNA library |
| 25110 | 2/19/2015 | 431_D | L003-r75 | TTMV | Anelloviridae | 52.63 | 5 | 87 | 1351 | 2567 | KF545580 | Virosaurus | DNA library |
| 25111 | 2/19/2015 | 432_D | L004-r75 | TTMV | Anelloviridae | 99.33 | 255 | 8441 | 2957 | 2977 | MN769977 | De novo | DNA library |
| 25114 | 2/20/2015 | 434_D | L006-r75 | TTMV | Anelloviridae | 14.59 | 8 | 199 | 436 | 2989 | KP343818 | Virosaurus | DNA library |
| 25117 | 2/20/2015 | 435_D | L005-r73 | TTMV | Anelloviridae | 42.96 | 3 | 50 | 1202 | 2798 | EF538883 | Virosaurus | DNA library |
| 25121 | 2/23/2015 | 436_D | L006-r73 | TTMV | Anelloviridae | 15.39 | 1 | 4 | 391 | 2541 | KT163888 | Virosaurus | DNA library |
| 25137 | 2/26/2015 | 437_D | L001-r75 | TTMV | Anelloviridae | 60.27 | 2 | 53 | 1547 | 2567 | KF545580 | Virosaurus | DNA library |
| 25145 | 3/2/2015 | 438_D | L003-r75 | TTMV | Anelloviridae | 10.07 | 8 | 39 | 300 | 2978 | KP343854 | Virosaurus | DNA library |
| 25146 | 3/2/2015 | 439_R | L002-r705 | TTMV | Anelloviridae | 23.83 | 15 | 108 | 686 | 2879 | MN770901 | De novo | RNA library |
| 25146 | 3/2/2015 | 439_D | L004-r75 | TTMV | Anelloviridae | 75.37 | 2 | 67 | 2215 | 2939 | KX810064 | Virosaurus | DNA library |
| 25147 | 3/2/2015 | 440_D | L005-r75 | TTMV | Anelloviridae | 70.44 | 12 | 482 | 2052 | 2913 | EF538882 | Virosaurus | DNA library |
| 25150 | 3/3/2015 | 441_D | L006-r75 | TTMV | Anelloviridae | 58.05 | 21 | 576 | 1731 | 2982 | KP343847 | Virosaurus | DNA library |
| 25151 | 3/3/2015 | 442_D | L005-r73 | TTMV | Anelloviridae | 43.04 | 9 | 201 | 1271 | 2953 | NC_014082 | Virosaurus | DNA library |
| 25156 | 3/4/2015 | 443_D | L006-r73 | TTMV | Anelloviridae | 96.86 | 23 | 718 | 2864 | 2957 | MN770128 | De novo | DNA library |
| 25160 | 3/5/2015 | 444_D | L001-r75 | TTMV | Anelloviridae | 99.1 | 87 | 2338 | 2876 | 2902 | MN770563 | De novo | DNA library |
| 25161 | 3/5/2015 | 587_D | L8-r88 | TTMV | Anelloviridae | 67.67 | 5 | 124 | 1737 | 2567 | KF545580 | Virosaurus | DNA library |
| 25162 | 3/5/2015 | 445_D | L002-r75 | TTMV | Anelloviridae | 43.14 | 5 | 118 | 1207 | 2798 | EF538883 | Virosaurus | DNA library |
| 25163 | 3/5/2015 | 446_D | L004-r75 | TTMV | Anelloviridae | 74.99 | 25 | 571 | 1925 | 2567 | KF545580 | Virosaurus | DNA library |
| 25164 | 3/5/2015 | 447_D | L005-r75 | TTMV | Anelloviridae | 84.2 | 1 | 45 | 2457 | 2918 | KF545583 | Virosaurus | DNA library |
| 25169 | 3/6/2015 | 448_D | L006-r75 | TTMV | Anelloviridae | 45.93 | 7 | 143 | 1288 | 2804 | NC_030297 | Virosaurus | DNA library |
| 25174 | 3/6/2015 | 449_D | L005-r73 | TTMV | Anelloviridae | 40.67 | 3 | 44 | 1201 | 2953 | NC_014082 | Virosaurus | DNA library |
| 25179 | 3/9/2015 | 450_D | L006-r73 | TTMV | Anelloviridae | 65.1 | 5 | 106 | 1671 | 2567 | KF545580 | Virosaurus | DNA library |
| 25194 | 3/12/2015 | 452_D | L001-r77 | TTMV | Anelloviridae | 96.84 | 801 | 23869 | 2819 | 2911 | NC_014068 | Virosaurus | DNA library |
| 25195 | 3/12/2015 | 453_D | L002-r77 | TTMV | Anelloviridae | 15.59 | 10 | 334 | 466 | 2989 | KP343818 | Virosaurus | DNA library |
| 25196 | 3/13/2015 | 454_D | L003-r77 | TTMV | Anelloviridae | 50.84 | 6 | 168 | 1516 | 2982 | KP343847 | Virosaurus | DNA library |
| 25199 | 3/12/2015 | 455_D | L004-r77 | TTMV | Anelloviridae | 100 | 2127 | 67183 | 2942 | 2942 | MN770127 | De novo | DNA library |
| 25203 | 3/17/2015 | 456_D | L005-r77 | TTMV | Anelloviridae | 97.35 | 19 | 546 | 2868 | 2946 | MN770305 | De novo | DNA library |
| 25204 | 3/17/2015 | 457_D | L001-r80 | TTMV | Anelloviridae | 21.59 | 1 | 18 | 620 | 2872 | EF538881 | Virosaurus | DNA library |
| 25207 | 3/17/2015 | 458_D | L8-r96 | TTMV | Anelloviridae | 23.09 | 3 | 20 | 558 | 2417 | KT163914 | Virosaurus | DNA library |
| 25212 | 3/18/2015 | 459_D | L001-r77 | TTMV | Anelloviridae | 83.06 | 48 | 1320 | 2343 | 2821 | MN770104 | De novo | DNA library |
| 25213 | 3/19/2015 | 460_D | L002-r77 | TTMV | Anelloviridae | 9.69 | 4 | 36 | 346 | 3571 | KF545584 | Virosaurus | DNA library |
| 25216 | 3/19/2015 | 461_D | L003-r77 | TTMV | Anelloviridae | 47.03 | 16 | 323 | 1370 | 2913 | EF538882 | Virosaurus | DNA library |
| 25217 | 3/19/2015 | 462_D | L004-r77 | TTMV | Anelloviridae | 21.04 | 2 | 19 | 613 | 2913 | EF538882 | Virosaurus | DNA library |
| 25220 | 3/19/2015 | 463_D | L005-r77 | TTMV | Anelloviridae | 99.35 | 749 | 20177 | 2881 | 2900 | MN769639 | De novo | DNA library |
| 25222 | 3/20/2015 | 464_D | L001-r80 | TTMV | Anelloviridae | 49.19 | 6 | 80 | 1310 | 2663 | MN768858 | De novo | DNA library |
| 25223 | 3/23/2015 | 771_D | L6-r113 | TTMV | Anelloviridae | 58.05 | 4 | 115 | 1640 | 2825 | KU243129 | Virosaurus | DNA library |
| 25233 | 3/24/2015 | 465_D | L002-r80 | TTMV | Anelloviridae | 89.05 | 6 | 159 | 2594 | 2913 | MN770403 | De novo | DNA library |
| 25247 | 3/26/2015 | 468_D | L003-r77 | TTMV | Anelloviridae | 92.38 | 21 | 683 | 2693 | 2915 | NC_025726 | Virosaurus | DNA library |
| 25253 | 3/27/2015 | 469_R | L6-r707 | TTMV | Anelloviridae | 14.86 | 58 | 286 | 429 | 2887 | MN771042 | De novo | RNA library |
| 25253 | 3/27/2015 | 469_D | L004-r77 | TTMV | Anelloviridae | 11.99 | 7 | 41 | 357 | 2978 | KP343854 | Virosaurus | DNA library |
| 25258 | 3/30/2015 | 470_D | L005-r77 | TTMV | Anelloviridae | 31.69 | 3 | 59 | 840 | 2651 | KT163876 | Virosaurus | DNA library |
| 25267 | 4/9/2015 | 471_D | L001-r80 | TTMV | Anelloviridae | 86.75 | 2 | 71 | 2527 | 2913 | NC_020498 | Virosaurus | DNA library |
| 25273 | 4/9/2015 | 472_D | L002-r80 | TTMV | Anelloviridae | 53.66 | 15 | 450 | 1561 | 2909 | NC_014089 | Virosaurus | DNA library |
| 25276 | 4/9/2015 | 473_D | L007-r75 | TTMV | Anelloviridae | 100 | 407 | 12652 | 2943 | 2943 | MN771329 | De novo | DNA library |
| 25282 | 4/10/2015 | 474_D | L001-r77 | TTMV | Anelloviridae | 93.47 | 8 | 272 | 2660 | 2846 | MN771220 | De novo | DNA library |
| 25283 | 4/10/2015 | 475_D | L003-r77 | TTMV | Anelloviridae | 19.52 | 4 | 68 | 697 | 3571 | KF545584 | Virosaurus | DNA library |
| 25286 | 4/10/2015 | 476_D | L004-r77 | TTMV | Anelloviridae | 60.4 | 28 | 1668 | 1757 | 2909 | NC_014089 | Virosaurus | DNA library |
| 25289 | 4/10/2015 | 477_D | L005-r77 | TTMV | Anelloviridae | 36.17 | 7 | 119 | 1012 | 2798 | EF538883 | Virosaurus | DNA library |
| 25299 | 4/13/2015 | 478_D | L001-r80 | TTMV | Anelloviridae | 66.07 | 2 | 72 | 1696 | 2567 | KF545580 | Virosaurus | DNA library |
| 25300 | 4/13/2015 | 479_R | L1-r713 | TTMV | Anelloviridae | 22.09 | 12 | 91 | 660 | 2988 | MN770490 | De novo | RNA library |
| 25300 | 4/13/2015 | 479_D | L002-r80 | TTMV | Anelloviridae | 21.99 | 4 | 129 | 582 | 2647 | KP343856 | Virosaurus | DNA library |
| 25302 | 4/16/2015 | 480_D | L007-r75 | TTMV | Anelloviridae | 94.68 | 178 | 5252 | 2761 | 2916 | JX134046 | Virosaurus | DNA library |
| 25317 | 4/15/2015 | 481_D | L001-r77 | TTMV | Anelloviridae | 99.97 | 1341 | 38656 | 2971 | 2972 | MN770349 | De novo | DNA library |
| 25323 | 4/15/2015 | 482_D | L002-r77 | TTMV | Anelloviridae | 52.71 | 25 | 542 | 1489 | 2825 | KU243129 | Virosaurus | DNA library |
| 25325 | 4/15/2015 | 483_R | L2-r713 | TTMV | Anelloviridae | 11.8 | 3 | 30 | 346 | 2933 | MN769192 | De novo | RNA library |
| 25325 | 4/15/2015 | 483_D | L004-r77 | TTMV | Anelloviridae | 12.91 | 2 | 105 | 461 | 3571 | KF545584 | Virosaurus | DNA library |
| 25329 | 4/16/2015 | 484_R | L2-r713 | TTMV | Anelloviridae | 14.9 | 8 | 41 | 439 | 2946 | MN769598 | De novo | RNA library |
| 25329 | 4/16/2015 | 484_D | L005-r77 | TTMV | Anelloviridae | 90.6 | 37 | 1650 | 2698 | 2978 | KP343854 | Virosaurus | DNA library |
| 25334 | 4/16/2015 | 485_D | L001-r80 | TTMV | Anelloviridae | 54.52 | 3 | 60 | 1545 | 2834 | KP343848 | Virosaurus | DNA library |
| 25342 | 4/16/2015 | 772_D | L7-r113 | TTMV | Anelloviridae | 48.07 | 3 | 106 | 1345 | 2798 | EF538883 | Virosaurus | DNA library |
| 25343 | 4/17/2015 | 486_D | L002-r80 | TTMV | Anelloviridae | 97.23 | 156 | 5315 | 2988 | 3073 | KF545582 | Virosaurus | DNA library |
| 25345 | 4/17/2015 | 487_D | L007-r75 | TTMV | Anelloviridae | 99.48 | 628 | 19348 | 2892 | 2907 | MN769849 | De novo | DNA library |
| 25353 | 4/21/2015 | 488_D | L001-r77 | TTMV | Anelloviridae | 99.7 | 311 | 11776 | 2951 | 2960 | MN770081 | De novo | DNA library |
| 25355 | 4/21/2015 | 489_D | L002-r77 | TTMV | Anelloviridae | 14.16 | 2 | 21 | 397 | 2804 | NC_030297 | Virosaurus | DNA library |
| 25361 | 4/22/2015 | 490_D | L003-r77 | TTMV | Anelloviridae | 73.89 | 2 | 62 | 2134 | 2888 | EF538880 | Virosaurus | DNA library |
| 25365 | 4/22/2015 | 491_D | L005-r77 | TTMV | Anelloviridae | 61.02 | 32 | 2009 | 1775 | 2909 | NC_014089 | Virosaurus | DNA library |
| 25368 | 4/22/2015 | 492_D | L001-r80 | TTMV | Anelloviridae | 65.5 | 1 | 34 | 1950 | 2977 | KP343834 | Virosaurus | DNA library |
| 25374 | 4/23/2015 | 493_D | L002-r80 | TTMV | Anelloviridae | 96.19 | 58 | 2093 | 2827 | 2939 | KX810064 | Virosaurus | DNA library |
| 25376 | 4/24/2015 | 798_D | L5-r113 | TTMV | Anelloviridae | 38.87 | 6 | 80 | 1137 | 2925 | KP343850 | Virosaurus | DNA library |
| 25380 | 4/27/2015 | 494_D | L007-r75 | TTMV | Anelloviridae | 99.56 | 137 | 3818 | 2937 | 2950 | MN770435 | De novo | DNA library |
| 25385 | 4/28/2015 | 495_D | L001-r77 | TTMV | Anelloviridae | 92.52 | 36 | 1137 | 2697 | 2915 | NC_025726 | Virosaurus | DNA library |
| 25396 | 4/28/2015 | 496_D | L003-r80 | TTMV | Anelloviridae | 98.12 | 99 | 2882 | 2929 | 2985 | MN770131 | De novo | DNA library |
| 25408 | 4/30/2015 | 497_D | L004-r80 | TTMV | Anelloviridae | 99.65 | 142 | 4094 | 2883 | 2893 | MN771421 | De novo | DNA library |
| 25409 | 4/30/2015 | 498_D | L005-r80 | TTMV | Anelloviridae | 100 | 111 | 3573 | 2827 | 2827 | KF545585 | Virosaurus | DNA library |
| 25413 | 5/5/2015 | 499_D | L006-r80 | TTMV | Anelloviridae | 27.74 | 2 | 23 | 804 | 2898 | NC_014088 | Virosaurus | DNA library |
| 25423 | 5/6/2015 | 500_D | L5-r82 | TTMV | Anelloviridae | 73.77 | 18 | 1555 | 2146 | 2909 | NC_014089 | Virosaurus | DNA library |
| 25424 | 5/6/2015 | 501_D | L6-r82 | TTMV | Anelloviridae | 33.26 | 2 | 20 | 920 | 2766 | NC_014086 | Virosaurus | DNA library |
| 25425 | 5/6/2015 | 502_D | L005-r84 | TTMV | Anelloviridae | 99.97 | 1598 | 46480 | 2921 | 2922 | MN770863 | De novo | DNA library |
| 25426 | 5/6/2015 | 503_D | L006-r84 | TTMV | Anelloviridae | 14.84 | 1 | 8 | 430 | 2898 | NC_014088 | Virosaurus | DNA library |
| 25432 | 5/6/2015 | 504_D | L004-r80 | TTMV | Anelloviridae | 30.98 | 3 | 45 | 857 | 2766 | NC_014086 | Virosaurus | DNA library |
| 25434 | 5/7/2015 | 505_D | L005-r80 | TTMV | Anelloviridae | 99.41 | 489 | 14642 | 3022 | 3040 | MN769985 | De novo | DNA library |
| 25435 | 5/7/2015 | 506_D | L006-r80 | TTMV | Anelloviridae | 96.62 | 51 | 1568 | 2828 | 2927 | MN769348 | De novo | DNA library |
| 25438 | 5/7/2015 | 507_D | L5-r82 | TTMV | Anelloviridae | 99.01 | 45 | 1285 | 2908 | 2937 | MN771847 | De novo | DNA library |
| 25441 | 5/7/2015 | 589_D | L2-r89 | TTMV | Anelloviridae | 50.5 | 8 | 160 | 1471 | 2913 | EF538882 | Virosaurus | DNA library |
| 25446 | 5/8/2015 | 508_D | L6-r82 | TTMV | Anelloviridae | 12.86 | 6 | 32 | 383 | 2978 | KP343854 | Virosaurus | DNA library |
| 25449 | 5/8/2015 | 509_D | L005-r84 | TTMV | Anelloviridae | 99.97 | 334 | 9226 | 2893 | 2894 | MN769087 | De novo | DNA library |
| 25455 | 5/11/2015 | 510_D | L006-r84 | TTMV | Anelloviridae | 97.25 | 11 | 412 | 2833 | 2913 | NC_020498 | Virosaurus | DNA library |
| 25459 | 5/12/2015 | 511_D | L003-r80 | TTMV | Anelloviridae | 98.96 | 35 | 1010 | 2863 | 2893 | MN769447 | De novo | DNA library |
| 25463 | 5/12/2015 | 512_D | L005-r80 | TTMV | Anelloviridae | 54.09 | 4 | 87 | 1582 | 2925 | KP343850 | Virosaurus | DNA library |
| 25467 | 5/12/2015 | 513_D | L006-r80 | TTMV | Anelloviridae | 99.97 | 1591 | 43529 | 2953 | 2954 | MN771257 | De novo | DNA library |
| 25468 | 5/12/2015 | 514_R | L2-r714 | TTMV | Anelloviridae | 28.66 | 8 | 79 | 828 | 2889 | MN770032 | De novo | RNA library |
| 25468 | 5/12/2015 | 514_D | L5-r82 | TTMV | Anelloviridae | 59.14 | 2 | 65 | 1724 | 2915 | NC_025726 | Virosaurus | DNA library |
| 25470 | 5/12/2015 | 515_D | L6-r82 | TTMV | Anelloviridae | 23.18 | 1 | 16 | 678 | 2925 | KP343850 | Virosaurus | DNA library |
| 25472 | 5/11/2015 | 516_D | L005-r84 | TTMV | Anelloviridae | 100 | 5731 | 165867 | 2945 | 2945 | MN770407 | De novo | DNA library |
| 25482 | 5/14/2015 | 517_D | L006-r84 | TTMV | Anelloviridae | 62.74 | 3 | 54 | 1778 | 2834 | KP343848 | Virosaurus | DNA library |
| 25484 | 5/14/2015 | 518_D | L003-r80 | TTMV | Anelloviridae | 98.47 | 208 | 6268 | 2904 | 2949 | MN769867 | De novo | DNA library |
| 25488 | 5/15/2015 | 519_D | L004-r80 | TTMV | Anelloviridae | 32.02 | 3 | 51 | 847 | 2645 | KP343849 | Virosaurus | DNA library |
| 25491 | 5/15/2015 | 520_D | L006-r80 | TTMV | Anelloviridae | 25.66 | 1 | 15 | 725 | 2825 | KU243129 | Virosaurus | DNA library |
| 25492 | 5/15/2015 | 521_D | L5-r82 | TTMV | Anelloviridae | 32.24 | 4 | 44 | 926 | 2872 | EF538881 | Virosaurus | DNA library |
| 25512 | 5/19/2015 | 591_D | L4-r89 | TTMV | Anelloviridae | 99.9 | 370 | 11063 | 2896 | 2899 | MN774790 | De novo | DNA library |
| 25515 | 5/19/2015 | 522_D | L6-r82 | TTMV | Anelloviridae | 12.96 | 15 | 211 | 386 | 2978 | KP343854 | Virosaurus | DNA library |
| 25517 | 5/19/2015 | 523_D | L005-r84 | TTMV | Anelloviridae | 98.18 | 981 | 35946 | 2860 | 2913 | NC_020498 | Virosaurus | DNA library |
| 25528 | 5/20/2015 | 524_D | L006-r84 | TTMV | Anelloviridae | 11.89 | 7 | 24 | 354 | 2978 | KP343854 | Virosaurus | DNA library |
| 25532 | 5/20/2015 | 525_D | L003-r80 | TTMV | Anelloviridae | 98.75 | 616 | 19309 | 2912 | 2949 | MN774547 | De novo | DNA library |
| 25535 | 5/21/2015 | 526_D | L004-r80 | TTMV | Anelloviridae | 82.04 | 93 | 2753 | 2343 | 2856 | NC_025727 | Virosaurus | DNA library |
| 25545 | 5/21/2015 | 773_D | L8-r113 | TTMV | Anelloviridae | 30.09 | 1 | 16 | 880 | 2925 | KP343850 | Virosaurus | DNA library |
| 25550 | 5/21/2015 | 527_D | L005-r80 | TTMV | Anelloviridae | 95.99 | 29 | 899 | 2798 | 2915 | NC_025726 | Virosaurus | DNA library |
| 25551 | 5/22/2015 | 528_D | L5-r82 | TTMV | Anelloviridae | 98.28 | 1996 | 60163 | 2919 | 2970 | MN769310 | De novo | DNA library |
| 25551 | 5/22/2015 | 528_R | L6-r714 | TTMV | Anelloviridae | 17.04 | 4 | 27 | 506 | 2970 | MN769310 | De novo | RNA library |
| 25556 | 5/22/2015 | 592_D | L1-r92 | TTMV | Anelloviridae | 25.34 | 2 | 13 | 701 | 2766 | NC_014086 | Virosaurus | DNA library |
| 25557 | 5/22/2015 | 775_D | L8-r111 | TTMV | Anelloviridae | 11.89 | 7 | 119 | 354 | 2978 | KP343854 | Virosaurus | DNA library |
| 25558 | 5/22/2015 | 529_D | L6-r82 | TTMV | Anelloviridae | 34.62 | 2 | 23 | 980 | 2831 | KX810063 | Virosaurus | DNA library |
| 25560 | 5/25/2015 | 861_R | L4-r745 | TTMV | Anelloviridae | 24.22 | 2 | 18 | 710 | 2932 | MN769690 | De novo | RNA library |
| 25560 | 5/25/2015 | 861_D | L4-r132 | TTMV | Anelloviridae | 95.43 | 127 | 5083 | 2842 | 2978 | KP343854 | Virosaurus | DNA library |
| 25563 | 5/25/2015 | 530_D | L005-r84 | TTMV | Anelloviridae | 99.65 | 791 | 21982 | 2865 | 2875 | MN769465 | De novo | DNA library |
| 25563 | 5/25/2015 | 530_R | L6-r714 | TTMV | Anelloviridae | 12.8 | 7 | 31 | 368 | 2875 | MN769465 | De novo | RNA library |
| 25569 | 5/26/2015 | 531_D | L006-r84 | TTMV | Anelloviridae | 14.23 | 20 | 279 | 406 | 2854 | KF764701 | Virosaurus | DNA library |
| 25572 | 5/26/2015 | 532_D | L003-r80 | TTMV | Anelloviridae | 100 | 1263 | 40592 | 2831 | 2831 | KX810063 | Virosaurus | DNA library |
| 25574 | 5/26/2015 | 533_D | L004-r80 | TTMV | Anelloviridae | 99.97 | 1572 | 44778 | 2940 | 2941 | MN769124 | De novo | DNA library |
| 25575 | 5/26/2015 | 534_R | L7-r714 | TTMV | Anelloviridae | 18.45 | 9 | 45 | 533 | 2889 | MN770009 | De novo | RNA library |
| 25575 | 5/26/2015 | 534_D | L005-r80 | TTMV | Anelloviridae | 59.56 | 8 | 226 | 1742 | 2925 | KP343850 | Virosaurus | DNA library |
| 25581 | 5/27/2015 | 535_D | L006-r80 | TTMV | Anelloviridae | 57.3 | 38 | 1019 | 1669 | 2913 | EF538882 | Virosaurus | DNA library |
| 25583 | 5/27/2015 | 536_D | L6-r82 | TTMV | Anelloviridae | 17.15 | 2 | 9 | 486 | 2834 | KP343848 | Virosaurus | DNA library |
| 25584 | 5/27/2015 | 537_D | L005-r84 | TTMV | Anelloviridae | 97.7 | 388 | 11786 | 2886 | 2954 | MN770532 | De novo | DNA library |
| 25586 | 5/27/2015 | 862_D | L5-r132 | TTMV | Anelloviridae | 15.86 | 1 | 7 | 462 | 2913 | EF538882 | Virosaurus | DNA library |
| 25589 | 5/27/2015 | 538_D | L006-r84 | TTMV | Anelloviridae | 45.65 | 2 | 35 | 1331 | 2916 | JX134046 | Virosaurus | DNA library |
| 25592 | 5/27/2015 | 539_D | L003-r80 | TTMV | Anelloviridae | 100 | 148729 | 4198696 | 2979 | 2979 | MN769209 | De novo | DNA library |
| 25594 | 5/28/2015 | 540_D | L004-r80 | TTMV | Anelloviridae | 32.07 | 5 | 72 | 921 | 2872 | EF538881 | Virosaurus | DNA library |
| 25596 | 5/28/2015 | 541_D | L1-r85 | TTMV | Anelloviridae | 99.21 | 352 | 10506 | 2900 | 2923 | MN774560 | De novo | DNA library |
| 25598 | 5/28/2015 | 542_D | L2-r85 | TTMV | Anelloviridae | 36.28 | 15 | 180 | 1042 | 2872 | EF538881 | Virosaurus | DNA library |
| 25599 | 5/28/2015 | 543_D | L3-r85 | TTMV | Anelloviridae | 97.92 | 14 | 468 | 2828 | 2888 | EF538880 | Virosaurus | DNA library |
| 25600 | 5/28/2015 | 544_D | L4-r85 | TTMV | Anelloviridae | 11.95 | 1 | 19 | 356 | 2978 | KP343854 | Virosaurus | DNA library |
| 25601 | 5/28/2015 | 545_D | L5-r85 | TTMV | Anelloviridae | 84.52 | 70 | 1955 | 2414 | 2856 | NC_025727 | Virosaurus | DNA library |
| 25602 | 5/28/2015 | 546_D | L6-r85 | TTMV | Anelloviridae | 99.93 | 968 | 27289 | 2951 | 2953 | MN769351 | De novo | DNA library |
| 25603 | 5/29/2015 | 547_D | L7-r85 | TTMV | Anelloviridae | 67.42 | 2 | 55 | 1964 | 2913 | EF538882 | Virosaurus | DNA library |
| 25604 | 5/29/2015 | 548_D | L8-r85 | TTMV | Anelloviridae | 98.98 | 117 | 3714 | 2909 | 2939 | KX810064 | Virosaurus | DNA library |
| 25606 | 5/29/2015 | 549_D | L2-r85 | TTMV | Anelloviridae | 48.34 | 12 | 169 | 1408 | 2913 | EF538882 | Virosaurus | DNA library |
| 25607 | 5/29/2015 | 550_D | L3-r85 | TTMV | Anelloviridae | 32.75 | 3 | 28 | 954 | 2913 | EF538882 | Virosaurus | DNA library |
| 25610 | 6/1/2015 | 552_R | L4-r717 | TTMV | Anelloviridae | 13.57 | 16 | 48 | 396 | 2918 | MN772158 | De novo | RNA library |
| 25610 | 6/1/2015 | 552_D | L5-r85 | TTMV | Anelloviridae | 56.64 | 54 | 1172 | 1650 | 2913 | EF538882 | Virosaurus | DNA library |
| 25611 | 6/1/2015 | 553_D | L6-r85 | TTMV | Anelloviridae | 99.73 | 1343 | 37300 | 2900 | 2908 | MN771081 | De novo | DNA library |
| 25613 | 6/1/2015 | 554_D | L7-r85 | TTMV | Anelloviridae | 19.3 | 1 | 10 | 540 | 2798 | EF538883 | Virosaurus | DNA library |
| 25617 | 6/2/2015 | 555_D | L8-r85 | TTMV | Anelloviridae | 79.97 | 9 | 226 | 2264 | 2831 | KX810063 | Virosaurus | DNA library |
| 25619 | 6/2/2015 | 556_D | L1-r85 | TTMV | Anelloviridae | 99.73 | 2835 | 78644 | 2920 | 2928 | MN769926 | De novo | DNA library |
| 25621 | 6/2/2015 | 863_R | L4-r745 | TTMV | Anelloviridae | 18.95 | 1 | 19 | 501 | 2644 | MN768859 | De novo | RNA library |
| 25621 | 6/2/2015 | 863_D | L6-r132 | TTMV | Anelloviridae | 27.51 | 2 | 18 | 665 | 2417 | KT163914 | Virosaurus | DNA library |
| 25625 | 6/2/2015 | 557_D | L3-r85 | TTMV | Anelloviridae | 41.47 | 7 | 89 | 1208 | 2913 | EF538882 | Virosaurus | DNA library |
| 25626 | 6/2/2015 | 594_D | L8-r88 | TTMV | Anelloviridae | 26.37 | 3 | 37 | 768 | 2913 | KP343851 | Virosaurus | DNA library |
| 25630 | 6/2/2015 | 558_R | L6-r717 | TTMV | Anelloviridae | 13.9 | 11 | 55 | 403 | 2900 | MN773734 | De novo | RNA library |
| 25630 | 6/2/2015 | 558_D | L4-r85 | TTMV | Anelloviridae | 33.04 | 4 | 50 | 949 | 2872 | EF538881 | Virosaurus | DNA library |
| 25632 | 6/3/2015 | 559_D | L5-r85 | TTMV | Anelloviridae | 99.68 | 499 | 13046 | 2837 | 2846 | MN771220 | De novo | DNA library |
| 25634 | 6/3/2015 | 560_D | L6-r85 | TTMV | Anelloviridae | 98.28 | 7047 | 204804 | 2962 | 3014 | MN771139 | De novo | DNA library |
| 25635 | 6/3/2015 | 561_D | L7-r85 | TTMV | Anelloviridae | 13.99 | 5 | 26 | 413 | 2953 | NC_014082 | Virosaurus | DNA library |
| 25636 | 6/3/2015 | 562_D | L8-r85 | TTMV | Anelloviridae | 99.77 | 5703 | 172632 | 3033 | 3040 | MN769985 | De novo | DNA library |
| 25639 | 6/3/2015 | 563_D | L1-r85 | TTMV | Anelloviridae | 100 | 212 | 8294 | 2989 | 2989 | KP343818 | Virosaurus | DNA library |
| 25643 | 6/4/2015 | 799_D | L6-r113 | TTMV | Anelloviridae | 76.7 | 48 | 1590 | 2314 | 3017 | KP343864 | Virosaurus | DNA library |
| 25644 | 6/4/2015 | 564_D | L2-r85 | TTMV | Anelloviridae | 12.99 | 1 | 6 | 373 | 2872 | EF538881 | Virosaurus | DNA library |
| 25650 | 6/4/2015 | 800_D | L7-r113 | TTMV | Anelloviridae | 71.03 | 5 | 141 | 2143 | 3017 | KP343864 | Virosaurus | DNA library |
| 25652 | 6/4/2015 | 801_D | L8-r113 | TTMV | Anelloviridae | 38.83 | 2 | 27 | 1131 | 2913 | EF538882 | Virosaurus | DNA library |
| 25653 | 6/4/2015 | 565_D | L4-r85 | TTMV | Anelloviridae | 69.62 | 36 | 866 | 1973 | 2834 | KP343848 | Virosaurus | DNA library |
| 25654 | 6/5/2015 | 566_D | L5-r85 | TTMV | Anelloviridae | 100 | 215 | 6706 | 2831 | 2831 | KX810063 | Virosaurus | DNA library |
| 25656 | 6/5/2015 | 567_D | L6-r85 | TTMV | Anelloviridae | 80.56 | 9 | 641 | 2399 | 2978 | KP343854 | Virosaurus | DNA library |
| 25657 | 6/5/2015 | 568_D | L7-r85 | TTMV | Anelloviridae | 100 | 673 | 19842 | 2968 | 2968 | MN773566 | De novo | DNA library |
| 25671 | 6/8/2015 | 569_D | L8-r85 | TTMV | Anelloviridae | 46.86 | 6 | 118 | 1314 | 2804 | NC_030297 | Virosaurus | DNA library |
| 25672 | 6/8/2015 | 570_D | L1-r85 | TTMV | Anelloviridae | 96.3 | 8 | 232 | 2781 | 2888 | MN770280 | De novo | DNA library |
| 25675 | 6/9/2015 | 571_D | L2-r85 | TTMV | Anelloviridae | 28 | 31 | 279 | 790 | 2821 | KP343863 | Virosaurus | DNA library |
| 25676 | 6/9/2015 | 572_R | L001-r722 | TTMV | Anelloviridae | 26.01 | 4 | 45 | 752 | 2891 | MN771179 | De novo | RNA library |
| 25676 | 6/9/2015 | 572_D | L3-r85 | TTMV | Anelloviridae | 67.97 | 21 | 576 | 1980 | 2913 | EF538882 | Virosaurus | DNA library |
| 25678 | 6/9/2015 | 573_D | L5-r85 | TTMV | Anelloviridae | 96.97 | 71 | 2138 | 2748 | 2834 | KP343848 | Virosaurus | DNA library |
| 25679 | 6/9/2015 | 574_D | L6-r85 | TTMV | Anelloviridae | 99.12 | 4679 | 125733 | 2926 | 2952 | MN770091 | De novo | DNA library |
| 25680 | 6/9/2015 | 575_D | L7-r85 | TTMV | Anelloviridae | 18.41 | 5 | 37 | 487 | 2645 | KP343849 | Virosaurus | DNA library |
| 25681 | 6/9/2015 | 864_D | L8-r127 | TTMV | Anelloviridae | 26.97 | 2 | 17 | 746 | 2766 | NC_014086 | Virosaurus | DNA library |
| 25683 | 6/9/2015 | 576_D | L8-r85 | TTMV | Anelloviridae | 98.78 | 11736 | 318669 | 2916 | 2952 | MN770091 | De novo | DNA library |
| 25685 | 6/9/2015 | 577_D | L1-r85 | TTMV | Anelloviridae | 99.83 | 178 | 8929 | 2986 | 2991 | MN770685 | De novo | DNA library |
| 25689 | 6/10/2015 | 578_D | L2-r85 | TTMV | Anelloviridae | 63.13 | 2 | 87 | 1839 | 2913 | NC_020498 | Virosaurus | DNA library |
| 25690 | 6/10/2015 | 579_D | L3-r85 | TTMV | Anelloviridae | 37.72 | 24 | 443 | 1064 | 2821 | KP343863 | Virosaurus | DNA library |
| 25691 | 6/10/2015 | 580_D | L4-r85 | TTMV | Anelloviridae | 10.39 | 8 | 53 | 371 | 3571 | KF545584 | Virosaurus | DNA library |
| 25692 | 6/10/2015 | 581_R | L004-r722 | TTMV | Anelloviridae | 31.97 | 5 | 41 | 938 | 2934 | MN769188 | De novo | RNA library |
| 25692 | 6/10/2015 | 581_D | L6-r85 | TTMV | Anelloviridae | 95.13 | 828 | 26227 | 2773 | 2915 | NC_025726 | Virosaurus | DNA library |
| 25694 | 6/11/2015 | 582_R | L004-r722 | TTMV | Anelloviridae | 12.16 | 4 | 20 | 357 | 2937 | MN771223 | De novo | RNA library |
| 25694 | 6/11/2015 | 582_D | L7-r85 | TTMV | Anelloviridae | 61.17 | 28 | 821 | 1782 | 2913 | EF538882 | Virosaurus | DNA library |
| 25700 | 6/11/2015 | 583_R | L004-r722 | TTMV | Anelloviridae | 17.52 | 6 | 39 | 504 | 2876 | MN771366 | De novo | RNA library |
| 25700 | 6/11/2015 | 583_D | L8-r85 | TTMV | Anelloviridae | 70.99 | 99 | 5914 | 2065 | 2909 | NC_014089 | Virosaurus | DNA library |
| 25710 | 6/15/2015 | 584_D | L1-r85 | TTMV | Anelloviridae | 100 | 23334 | 743235 | 2989 | 2989 | KP343818 | Virosaurus | DNA library |
| 25710 | 6/15/2015 | 584_R | L004-r722 | TTMV | Anelloviridae | 84.41 | 3 | 145 | 2523 | 2989 | KP343818 | Virosaurus | RNA library |
| 25715 | 6/15/2015 | 585_D | L2-r85 | TTMV | Anelloviridae | 18.64 | 1 | 14 | 557 | 2989 | KP343818 | Virosaurus | DNA library |
| 25718 | 6/15/2015 | 681_D | L2-r93 | TTMV | Anelloviridae | 76.22 | 3 | 123 | 2240 | 2939 | KX810064 | Virosaurus | DNA library |
| 25721 | 6/16/2015 | 682_D | L3-r93 | TTMV | Anelloviridae | 98.95 | 121 | 3555 | 2927 | 2958 | MN770359 | De novo | DNA library |
| 25721 | 6/16/2015 | 682_R | L2-r734 | TTMV | Anelloviridae | 25.7 | 4 | 42 | 748 | 2911 | MN769676 | De novo | RNA library |
| 25722 | 6/16/2015 | 683_R | L2-r734 | TTMV | Anelloviridae | 21.48 | 3 | 26 | 628 | 2924 | MN770144 | De novo | RNA library |
| 25722 | 6/16/2015 | 683_D | L4-r93 | TTMV | Anelloviridae | 12.61 | 7 | 49 | 377 | 2989 | KP343818 | Virosaurus | DNA library |
| 25723 | 6/16/2015 | 684_D | L4-r92 | TTMV | Anelloviridae | 65.17 | 10 | 224 | 1841 | 2825 | KU243129 | Virosaurus | DNA library |
| 25729 | 6/17/2015 | 685_D | L5-r92 | TTMV | Anelloviridae | 99.93 | 1870 | 51917 | 2975 | 2977 | MN770393 | De novo | DNA library |
| 25730 | 6/17/2015 | 686_D | L6-r92 | TTMV | Anelloviridae | 14.65 | 23 | 929 | 438 | 2989 | KP343818 | Virosaurus | DNA library |
| 25731 | 6/17/2015 | 687_R | L3-r734 | TTMV | Anelloviridae | 14.81 | 2 | 10 | 419 | 2829 | MN771589 | De novo | RNA library |
| 25731 | 6/17/2015 | 687_D | L1-r93 | TTMV | Anelloviridae | 16.28 | 7 | 103 | 493 | 3028 | KP343862 | Virosaurus | DNA library |
| 25733 | 6/18/2015 | 865_D | L1-r132 | TTMV | Anelloviridae | 98.82 | 24703 | 695837 | 2934 | 2969 | MN769748 | De novo | DNA library |
| 25733 | 6/18/2015 | 865_R | L5-r745 | TTMV | Anelloviridae | 11.22 | 1 | 12 | 333 | 2969 | MN769748 | De novo | RNA library |
| 25734 | 6/18/2015 | 688_D | L2-r93 | TTMV | Anelloviridae | 62.6 | 6 | 144 | 1607 | 2567 | KF545580 | Virosaurus | DNA library |
| 25736 | 6/18/2015 | 866_R | L5-r745 | TTMV | Anelloviridae | 34.29 | 3 | 69 | 990 | 2887 | MN769158 | De novo | RNA library |
| 25736 | 6/18/2015 | 866_D | L2-r132 | TTMV | Anelloviridae | 78.59 | 31 | 1122 | 2371 | 3017 | KP343864 | Virosaurus | DNA library |
| 25740 | 6/18/2015 | 689_D | L3-r93 | TTMV | Anelloviridae | 95.85 | 367 | 9986 | 2796 | 2917 | MN771514 | De novo | DNA library |
| 25742 | 6/19/2015 | 690_D | L4-r93 | TTMV | Anelloviridae | 91.74 | 4 | 216 | 2677 | 2918 | KF545583 | Virosaurus | DNA library |
| 25746 | 6/19/2015 | 691_D | L3-r92 | TTMV | Anelloviridae | 32.68 | 3 | 55 | 956 | 2925 | KP343850 | Virosaurus | DNA library |
| 25747 | 6/22/2015 | 692_D | L5-r92 | TTMV | Anelloviridae | 99.97 | 444 | 14834 | 2991 | 2992 | MN770170 | De novo | DNA library |
| 25753 | 6/22/2015 | 693_D | L6-r92 | TTMV | Anelloviridae | 98.46 | 16 | 505 | 2883 | 2928 | MN769926 | De novo | DNA library |
| 25756 | 6/22/2015 | 599_D | L1-r92 | TTMV | Anelloviridae | 71.08 | 3 | 96 | 2089 | 2939 | KX810064 | Virosaurus | DNA library |
| 25757 | 6/22/2015 | 597_D | L3-r89 | TTMV | Anelloviridae | 97.12 | 72 | 2257 | 2829 | 2913 | NC_020498 | Virosaurus | DNA library |
| 25758 | 6/23/2015 | 694_D | L1-r93 | TTMV | Anelloviridae | 99.31 | 11 | 364 | 2895 | 2915 | MN771115 | De novo | DNA library |
| 25761 | 6/23/2015 | 601_D | L7-r88 | TTMV | Anelloviridae | 99.62 | 499 | 15530 | 2888 | 2899 | MN769301 | De novo | DNA library |
| 25762 | 6/23/2015 | 600_D | L2-r92 | TTMV | Anelloviridae | 99.52 | 1023 | 28557 | 2905 | 2919 | MN769076 | De novo | DNA library |
| 25764 | 6/23/2015 | 695_D | L2-r93 | TTMV | Anelloviridae | 10.7 | 1 | 3 | 300 | 2804 | NC_030297 | Virosaurus | DNA library |
| 25766 | 6/24/2015 | 696_D | L3-r93 | TTMV | Anelloviridae | 96.84 | 855 | 27789 | 2819 | 2911 | NC_014068 | Virosaurus | DNA library |
| 25767 | 6/24/2015 | 603_D | L2-r89 | TTMV | Anelloviridae | 61.36 | 1 | 224 | 1834 | 2989 | KP343818 | Virosaurus | DNA library |
| 25768 | 6/24/2015 | 605_D | L4-r89 | TTMV | Anelloviridae | 51.61 | 3 | 91 | 1505 | 2916 | JX134046 | Virosaurus | DNA library |
| 25772 | 6/24/2015 | 697_D | L4-r93 | TTMV | Anelloviridae | 81.92 | 9 | 257 | 2424 | 2959 | KT163898 | Virosaurus | DNA library |
| 25773 | 6/24/2015 | 698_D | L3-r92 | TTMV | Anelloviridae | 59.25 | 2 | 54 | 1679 | 2834 | KP343848 | Virosaurus | DNA library |
| 25774 | 6/24/2015 | 606_D | L1-r92 | TTMV | Anelloviridae | 51.72 | 4 | 120 | 1447 | 2798 | EF538883 | Virosaurus | DNA library |
| 25777 | 6/24/2015 | 604_R | L004-r723 | TTMV | Anelloviridae | 16.21 | 11 | 47 | 467 | 2881 | MN771153 | De novo | RNA library |
| 25777 | 6/24/2015 | 604_D | L3-r89 | TTMV | Anelloviridae | 49.91 | 14 | 316 | 1454 | 2913 | EF538882 | Virosaurus | DNA library |
| 25778 | 6/25/2015 | 699_D | L4-r92 | TTMV | Anelloviridae | 64.85 | 6 | 152 | 1832 | 2825 | KU243129 | Virosaurus | DNA library |
| 25779 | 6/25/2015 | 700_D | L6-r92 | TTMV | Anelloviridae | 35.37 | 4 | 44 | 1025 | 2898 | NC_014088 | Virosaurus | DNA library |
| 25783 | 6/25/2015 | 607_D | L2-r92 | TTMV | Anelloviridae | 97.12 | 26 | 767 | 2902 | 2988 | MN770490 | De novo | DNA library |
| 25784 | 6/25/2015 | 701_D | L1-r93 | TTMV | Anelloviridae | 91.63 | 67 | 4310 | 3272 | 3571 | KF545584 | Virosaurus | DNA library |
| 25786 | 6/24/2015 | 702_D | L2-r93 | TTMV | Anelloviridae | 97.59 | 104 | 2836 | 2675 | 2741 | MN771835 | De novo | DNA library |
| 25787 | 6/25/2015 | 703_D | L3-r93 | TTMV | Anelloviridae | 96.51 | 23 | 778 | 2878 | 2982 | MN769044 | De novo | DNA library |
| 25788 | 6/25/2015 | 704_D | L4-r93 | TTMV | Anelloviridae | 33.81 | 3 | 32 | 985 | 2913 | EF538882 | Virosaurus | DNA library |
| 25789 | 6/25/2015 | 705_D | L3-r92 | TTMV | Anelloviridae | 45.49 | 7 | 107 | 1325 | 2913 | EF538882 | Virosaurus | DNA library |
| 25790 | 6/26/2015 | 706_D | L4-r92 | TTMV | Anelloviridae | 97.3 | 14 | 443 | 2915 | 2996 | MN771181 | De novo | DNA library |
| 25792 | 6/26/2015 | 707_D | L5-r92 | TTMV | Anelloviridae | 99.97 | 1050 | 29080 | 2915 | 2916 | MN769051 | De novo | DNA library |
| 25798 | 6/26/2015 | 709_D | L2-r93 | TTMV | Anelloviridae | 19.24 | 1 | 49 | 575 | 2989 | KP343818 | Virosaurus | DNA library |
| 25799 | 6/29/2015 | 609_D | L8-r88 | TTMV | Anelloviridae | 35.65 | 7 | 98 | 1033 | 2898 | NC_014088 | Virosaurus | DNA library |
| 25801 | 6/29/2015 | 608_D | L7-r88 | TTMV | Anelloviridae | 98.83 | 87 | 2756 | 2870 | 2904 | MN770663 | De novo | DNA library |
| 25803 | 6/29/2015 | 710_D | L3-r93 | TTMV | Anelloviridae | 98.52 | 80 | 2657 | 2924 | 2968 | MN771048 | De novo | DNA library |
| 25805 | 6/29/2015 | 610_D | L2-r89 | TTMV | Anelloviridae | 74.07 | 4 | 118 | 2099 | 2834 | KP343848 | Virosaurus | DNA library |
| 25807 | 6/29/2015 | 711_D | L4-r93 | TTMV | Anelloviridae | 23.39 | 2 | 13 | 647 | 2766 | NC_014086 | Virosaurus | DNA library |
| 25812 | 6/30/2015 | 613_D | L1-r92 | TTMV | Anelloviridae | 29.42 | 2 | 22 | 845 | 2872 | EF538881 | Virosaurus | DNA library |
| 25813 | 6/30/2015 | 612_D | L4-r89 | TTMV | Anelloviridae | 99.86 | 1460 | 40336 | 2915 | 2919 | MN769524 | De novo | DNA library |
| 25814 | 6/30/2015 | 712_D | L3-r92 | TTMV | Anelloviridae | 74.16 | 23 | 649 | 2095 | 2825 | KU243129 | Virosaurus | DNA library |
| 25819 | 6/30/2015 | 714_D | L5-r92 | TTMV | Anelloviridae | 100 | 3681 | 110563 | 2947 | 2947 | MN770562 | De novo | DNA library |
| 25823 | 7/1/2015 | 715_R | L2-r735 | TTMV | Anelloviridae | 10.31 | 1 | 8 | 307 | 2977 | MN769977 | De novo | RNA library |
| 25823 | 7/1/2015 | 715_D | L6-r92 | TTMV | Anelloviridae | 31.45 | 2 | 22 | 916 | 2913 | EF538882 | Virosaurus | DNA library |
| 25826 | 7/1/2015 | 716_D | L2-r93 | TTMV | Anelloviridae | 55.08 | 3 | 91 | 1611 | 2925 | KP343850 | Virosaurus | DNA library |
| 25829 | 7/1/2015 | 717_D | L3-r93 | TTMV | Anelloviridae | 99.6 | 172 | 5035 | 2955 | 2967 | MN770049 | De novo | DNA library |
| 25829 | 7/1/2015 | 717_R | L3-r735 | TTMV | Anelloviridae | 10.42 | 3 | 8 | 300 | 2879 | MN768666 | De novo | RNA library |
| 25830 | 7/1/2015 | 718_D | L4-r93 | TTMV | Anelloviridae | 34.6 | 4 | 64 | 957 | 2766 | NC_014086 | Virosaurus | DNA library |
| 25831 | 7/1/2015 | 719_D | L3-r92 | TTMV | Anelloviridae | 14.1 | 8 | 94 | 420 | 2978 | KP343854 | Virosaurus | DNA library |
| 25837 | 7/2/2015 | 720_D | L4-r92 | TTMV | Anelloviridae | 27.88 | 2 | 27 | 790 | 2834 | KP343848 | Virosaurus | DNA library |
| 25838 | 7/2/2015 | 721_D | L5-r93 | TTMV | Anelloviridae | 93.79 | 17 | 560 | 2734 | 2915 | NC_025726 | Virosaurus | DNA library |
| 25840 | 7/2/2015 | 722_D | L6-r93 | TTMV | Anelloviridae | 99.63 | 471 | 13194 | 2930 | 2941 | MN769124 | De novo | DNA library |
| 25841 | 7/2/2015 | 723_D | L7-r93 | TTMV | Anelloviridae | 17.3 | 2 | 8 | 506 | 2925 | KP343850 | Virosaurus | DNA library |
| 25842 | 7/2/2015 | 724_D | L8-r93 | TTMV | Anelloviridae | 100 | 5000 | 147649 | 2974 | 2974 | MN770044 | De novo | DNA library |
| 25843 | 7/3/2015 | 725_D | L4-r96 | TTMV | Anelloviridae | 98.85 | 356 | 10740 | 2921 | 2955 | MN769711 | De novo | DNA library |
| 25843 | 7/3/2015 | 725_R | L6-r735 | TTMV | Anelloviridae | 10.71 | 1 | 14 | 311 | 2904 | MN770663 | De novo | RNA library |
| 25850 | 7/3/2015 | 726_D | L5-r96 | TTMV | Anelloviridae | 22.56 | 2 | 16 | 660 | 2925 | KP343850 | Virosaurus | DNA library |
| 25851 | 7/6/2015 | 727_D | L6-r96 | TTMV | Anelloviridae | 24.7 | 2 | 100 | 691 | 2798 | EF538883 | Virosaurus | DNA library |
| 25865 | 7/8/2015 | 728_D | L7-r96 | TTMV | Anelloviridae | 44.6 | 5 | 76 | 1312 | 2942 | KP343858 | Virosaurus | DNA library |
| 25873 | 7/10/2015 | 618_D | L3-r89 | TTMV | Anelloviridae | 59.94 | 23 | 458 | 1746 | 2913 | EF538882 | Virosaurus | DNA library |
| 25874 | 7/9/2015 | 776_D | L4-r113 | TTMV | Anelloviridae | 97.98 | 13 | 400 | 2864 | 2923 | MN770193 | De novo | DNA library |
| 25876 | 7/9/2015 | 729_D | L6-r93 | TTMV | Anelloviridae | 63.23 | 2 | 82 | 1790 | 2831 | KX810063 | Virosaurus | DNA library |
| 25880 | 7/9/2015 | 669_D | L3-r91 | TTMV | Anelloviridae | 98.57 | 5 | 174 | 2885 | 2927 | MN769118 | De novo | DNA library |
| 25882 | 7/10/2015 | 730_D | L7-r93 | TTMV | Anelloviridae | 66.01 | 2 | 91 | 1923 | 2913 | EF538882 | Virosaurus | DNA library |
| 25883 | 7/10/2015 | 731_D | L8-r93 | TTMV | Anelloviridae | 52.93 | 2 | 45 | 1500 | 2834 | KP343848 | Virosaurus | DNA library |
| 25885 | 7/13/2015 | 732_D | L4-r96 | TTMV | Anelloviridae | 92.35 | 92 | 3158 | 2763 | 2992 | MN770170 | De novo | DNA library |
| 25886 | 7/10/2015 | 733_D | L5-r96 | TTMV | Anelloviridae | 13.23 | 1 | 5 | 371 | 2804 | NC_030297 | Virosaurus | DNA library |
| 25893 | 7/10/2015 | 734_D | L6-r96 | TTMV | Anelloviridae | 99.75 | 848 | 22556 | 2745 | 2752 | MN772319 | De novo | DNA library |
| 25895 | 7/13/2015 | 735_D | L7-r96 | TTMV | Anelloviridae | 99.65 | 3616 | 102151 | 2851 | 2861 | MN771305 | De novo | DNA library |
| 25897 | 7/13/2015 | 736_D | L5-r93 | TTMV | Anelloviridae | 11.17 | 1 | 9 | 334 | 2989 | KP343818 | Virosaurus | DNA library |
| 25898 | 7/13/2015 | 737_D | L7-r93 | TTMV | Anelloviridae | 14.96 | 2 | 10 | 422 | 2821 | KP343863 | Virosaurus | DNA library |
| 25900 | 7/13/2015 | 738_D | L8-r93 | TTMV | Anelloviridae | 12.53 | 3 | 36 | 373 | 2978 | KP343854 | Virosaurus | DNA library |
| 25910 | 7/16/2015 | 777_D | L5-r113 | TTMV | Anelloviridae | 55.8 | 3 | 104 | 1632 | 2925 | KP343850 | Virosaurus | DNA library |
| 25922 | 7/16/2015 | 620_D | L1-r92 | TTMV | Anelloviridae | 100 | 3247 | 90001 | 2976 | 2976 | MN771904 | De novo | DNA library |
| 25923 | 7/16/2015 | 867_D | L3-r132 | TTMV | Anelloviridae | 31.79 | 1 | 31 | 926 | 2913 | KP343851 | Virosaurus | DNA library |
| 25930 | 7/21/2015 | 622_D | L7-r88 | TTMV | Anelloviridae | 96.72 | 1003 | 29981 | 2921 | 3020 | MN770161 | De novo | DNA library |
| 25938 | 7/21/2015 | 739_D | L4-r96 | TTMV | Anelloviridae | 90.29 | 3 | 140 | 2556 | 2831 | KX810063 | Virosaurus | DNA library |
| 25939 | 7/21/2015 | 740_D | L5-r96 | TTMV | Anelloviridae | 99.8 | 260 | 8143 | 2928 | 2934 | MN771043 | De novo | DNA library |
| 25940 | 7/21/2015 | 741_D | L6-r96 | TTMV | Anelloviridae | 10.63 | 2 | 6 | 308 | 2898 | NC_014088 | Virosaurus | DNA library |
| 25942 | 7/21/2015 | 868_D | L4-r132 | TTMV | Anelloviridae | 92.78 | 92 | 5296 | 3313 | 3571 | KF545584 | Virosaurus | DNA library |
| 25942 | 7/21/2015 | 868_R | L6-r745 | TTMV | Anelloviridae | 11.2 | 1 | 6 | 400 | 3571 | KF545584 | Virosaurus | RNA library |
| 25946 | 7/23/2015 | 628_R | L2-r724 | TTMV | Anelloviridae | 50.9 | 8 | 171 | 1521 | 2988 | MN770490 | De novo | RNA library |
| 25946 | 7/23/2015 | 628_D | L2-r92 | TTMV | Anelloviridae | 44.46 | 6 | 1317 | 1313 | 2953 | NC_014082 | Virosaurus | DNA library |
| 25947 | 7/23/2015 | 630_D | L8-r88 | TTMV | Anelloviridae | 100 | 89 | 2650 | 2827 | 2827 | KF545585 | Virosaurus | DNA library |
| 25947 | 7/23/2015 | 630_R | L3-r724 | TTMV | Anelloviridae | 16.1 | 1 | 9 | 455 | 2827 | KF545585 | Virosaurus | RNA library |
| 25954 | 7/23/2015 | 629_D | L7-r88 | TTMV | Anelloviridae | 96.84 | 477 | 13327 | 2819 | 2911 | NC_014068 | Virosaurus | DNA library |
| 25955 | 7/23/2015 | 742_D | L7-r96 | TTMV | Anelloviridae | 10.11 | 6 | 35 | 301 | 2978 | KP343854 | Virosaurus | DNA library |
| 25965 | 7/28/2015 | 631_D | L1-r91 | TTMV | Anelloviridae | 47.27 | 4 | 73 | 1377 | 2913 | EF538882 | Virosaurus | DNA library |
| 25969 | 7/27/2015 | 743_D | L5-r93 | TTMV | Anelloviridae | 98.97 | 68 | 2007 | 2880 | 2910 | MN769372 | De novo | DNA library |
| 25980 | 7/28/2015 | 744_D | L6-r93 | TTMV | Anelloviridae | 21.8 | 2 | 24 | 626 | 2872 | EF538881 | Virosaurus | DNA library |
| 25982 | 7/28/2015 | 745_D | L8-r93 | TTMV | Anelloviridae | 20.98 | 2 | 10 | 611 | 2913 | EF538882 | Virosaurus | DNA library |
| 25987 | 7/30/2015 | 746_D | L4-r96 | TTMV | Anelloviridae | 100 | 354 | 11040 | 2831 | 2831 | KX810063 | Virosaurus | DNA library |
| 25993 | 7/30/2015 | 747_D | L5-r96 | TTMV | Anelloviridae | 14.22 | 2 | 9 | 416 | 2925 | KP343850 | Virosaurus | DNA library |
| 25995 | 7/30/2015 | 748_D | L6-r96 | TTMV | Anelloviridae | 90.06 | 480 | 13280 | 2600 | 2887 | MN769047 | De novo | DNA library |
| 26003 | 7/31/2015 | 635_D | L5-r91 | TTMV | Anelloviridae | 98.27 | 13 | 387 | 2833 | 2883 | MN770332 | De novo | DNA library |
| 26004 | 8/3/2015 | 634_D | L4-r91 | TTMV | Anelloviridae | 76.85 | 223 | 6123 | 2241 | 2916 | JX134046 | Virosaurus | DNA library |
| 26015 | 8/4/2015 | 749_D | L7-r96 | TTMV | Anelloviridae | 88.61 | 6 | 194 | 2583 | 2915 | NC_025726 | Virosaurus | DNA library |
| 26019 | 8/5/2015 | 636_D | L6-r91 | TTMV | Anelloviridae | 37.24 | 6 | 66 | 1030 | 2766 | NC_014086 | Virosaurus | DNA library |
| 26027 | 8/5/2015 | 750_D | L5-r93 | TTMV | Anelloviridae | 27.71 | 2 | 18 | 704 | 2541 | KT163888 | Virosaurus | DNA library |
| 26028 | 8/5/2015 | 751_D | L6-r93 | TTMV | Anelloviridae | 98.65 | 146 | 4082 | 2779 | 2817 | MN770506 | De novo | DNA library |
| 26033 | 8/6/2015 | 752_D | L7-r93 | TTMV | Anelloviridae | 95.88 | 25 | 757 | 2724 | 2841 | MN771398 | De novo | DNA library |
| 26040 | 8/6/2015 | 638_R | L5-r724 | TTMV | Anelloviridae | 10.1 | 1 | 4 | 300 | 2970 | MN769310 | De novo | RNA library |
| 26040 | 8/6/2015 | 638_D | L8-r91 | TTMV | Anelloviridae | 91.46 | 7 | 228 | 2666 | 2915 | NC_025726 | Virosaurus | DNA library |
| 26042 | 8/6/2015 | 637_D | L7-r91 | TTMV | Anelloviridae | 96.64 | 362 | 10665 | 2820 | 2918 | MN772158 | De novo | DNA library |
| 26046 | 8/7/2015 | 753_D | L4-r96 | TTMV | Anelloviridae | 97.61 | 57 | 2063 | 2855 | 2925 | MN771738 | De novo | DNA library |
| 26047 | 8/14/2015 | 778_D | L6-r113 | TTMV | Anelloviridae | 100 | 1811 | 51078 | 2925 | 2925 | MN773813 | De novo | DNA library |
| 26049 | 8/10/2015 | 754_D | L5-r96 | TTMV | Anelloviridae | 71.7 | 3 | 66 | 2032 | 2834 | KP343848 | Virosaurus | DNA library |
| 26050 | 8/10/2015 | 641_D | L4-r91 | TTMV | Anelloviridae | 99.05 | 2048 | 56579 | 2810 | 2837 | MN769619 | De novo | DNA library |
| 26052 | 8/10/2015 | 755_D | L6-r96 | TTMV | Anelloviridae | 18.44 | 1 | 11 | 510 | 2766 | NC_014086 | Virosaurus | DNA library |
| 26063 | 8/11/2015 | 756_D | L7-r96 | TTMV | Anelloviridae | 100 | 2276 | 61964 | 2974 | 2974 | MN770044 | De novo | DNA library |
| 26074 | 8/10/2015 | 780_D | L8-r113 | TTMV | Anelloviridae | 39.27 | 2 | 45 | 1144 | 2913 | EF538882 | Virosaurus | DNA library |
| 26076 | 8/13/2015 | 757_D | L5-r93 | TTMV | Anelloviridae | 15.98 | 40 | 246 | 476 | 2978 | KP343854 | Virosaurus | DNA library |
| 26077 | 8/13/2015 | 643_D | L6-r91 | TTMV | Anelloviridae | 87.41 | 138 | 4079 | 2569 | 2939 | KX810064 | Virosaurus | DNA library |
| 26078 | 8/13/2015 | 642_R | L6-r724 | TTMV | Anelloviridae | 11.23 | 7 | 24 | 313 | 2787 | MN771867 | De novo | RNA library |
| 26078 | 8/13/2015 | 642_D | L5-r91 | TTMV | Anelloviridae | 70.29 | 20 | 940 | 2096 | 2982 | KP343847 | Virosaurus | DNA library |
| 26079 | 8/13/2015 | 674_D | L1-r91 | TTMV | Anelloviridae | 42.36 | 7 | 115 | 1234 | 2913 | EF538882 | Virosaurus | DNA library |
| 26082 | 8/13/2015 | 758_D | L6-r93 | TTMV | Anelloviridae | 97.01 | 54 | 1766 | 2981 | 3073 | KF545582 | Virosaurus | DNA library |
| 26082 | 8/13/2015 | 758_R | L8-r736 | TTMV | Anelloviridae | 11.28 | 1 | 4 | 322 | 2856 | NC_025727 | Virosaurus | RNA library |
| 26083 | 8/13/2015 | 802_D | L6-r111 | TTMV | Anelloviridae | 41.37 | 2 | 34 | 1205 | 2913 | EF538882 | Virosaurus | DNA library |
| 26089 | 8/14/2015 | 760_D | L8-r93 | TTMV | Anelloviridae | 12.25 | 5 | 45 | 366 | 2989 | KP343818 | Virosaurus | DNA library |
| 26091 | 8/14/2015 | 761_D | L5-r96 | TTMV | Anelloviridae | 37.1 | 2 | 20 | 1048 | 2825 | KU243129 | Virosaurus | DNA library |
| 26100 | 8/17/2015 | 762_D | L6-r96 | TTMV | Anelloviridae | 13.82 | 1 | 5 | 412 | 2982 | KP343847 | Virosaurus | DNA library |
| 26101 | 9/9/2015 | 763_R | L1-r737 | TTMV | Anelloviridae | 17.44 | 3 | 19 | 510 | 2924 | MN770144 | De novo | RNA library |
| 26101 | 9/9/2015 | 763_D | L7-r96 | TTMV | Anelloviridae | 18.16 | 89 | 686 | 558 | 3073 | KF545582 | Virosaurus | DNA library |
| 26102 | 8/18/2015 | 764_D | L5-r93 | TTMV | Anelloviridae | 10.95 | 5 | 42 | 326 | 2978 | KP343854 | Virosaurus | DNA library |
| 26104 | 8/18/2015 | 765_R | L2-r737 | TTMV | Anelloviridae | 19.95 | 2 | 26 | 584 | 2928 | MN769926 | De novo | RNA library |
| 26104 | 8/18/2015 | 765_D | L6-r93 | TTMV | Anelloviridae | 28.88 | 2 | 33 | 837 | 2898 | NC_014088 | Virosaurus | DNA library |
| 26107 | 8/18/2015 | 803_D | L7-r111 | TTMV | Anelloviridae | 69.37 | 40 | 2683 | 2018 | 2909 | NC_014089 | Virosaurus | DNA library |
| 26108 | 8/18/2015 | 804_D | L8-r111 | TTMV | Anelloviridae | 85.94 | 2 | 81 | 2641 | 3073 | KF545582 | Virosaurus | DNA library |
| 26111 | 8/18/2015 | 805_D | L4-r113 | TTMV | Anelloviridae | 15.82 | 9 | 113 | 471 | 2978 | KP343854 | Virosaurus | DNA library |
| 26114 | 8/19/2015 | 644_D | L7-r91 | TTMV | Anelloviridae | 80.27 | 14 | 477 | 2359 | 2939 | KX810064 | Virosaurus | DNA library |
| 26124 | 8/21/2015 | 806_D | L6-r113 | TTMV | Anelloviridae | 90.74 | 48 | 1367 | 2664 | 2936 | MN769585 | De novo | DNA library |
| 26129 | 8/21/2015 | 807_D | L7-r113 | TTMV | Anelloviridae | 37.57 | 4 | 73 | 1079 | 2872 | EF538881 | Virosaurus | DNA library |
| 26130 | 8/24/2015 | 808_R | L5-r740 | TTMV | Anelloviridae | 13.5 | 6 | 29 | 394 | 2918 | MN772158 | De novo | RNA library |
| 26130 | 8/24/2015 | 808_D | L8-r113 | TTMV | Anelloviridae | 60.06 | 35 | 929 | 1747 | 2909 | NC_014089 | Virosaurus | DNA library |
| 26131 | 8/24/2015 | 645_D | L8-r91 | TTMV | Anelloviridae | 94.12 | 10 | 315 | 2738 | 2909 | MN770897 | De novo | DNA library |
| 26134 | 8/24/2015 | 809_D | L6-r111 | TTMV | Anelloviridae | 32.09 | 2 | 29 | 944 | 2942 | KP343858 | Virosaurus | DNA library |
| 26140 | 8/25/2015 | 647_D | L3-r91 | TTMV | Anelloviridae | 33.61 | 3 | 37 | 979 | 2913 | EF538882 | Virosaurus | DNA library |
| 26141 | 8/25/2015 | 646_D | L1-r91 | TTMV | Anelloviridae | 16.22 | 17 | 345 | 479 | 2953 | NC_014082 | Virosaurus | DNA library |
| 26142 | 8/25/2015 | 810_D | L7-r111 | TTMV | Anelloviridae | 94.07 | 26 | 835 | 2742 | 2915 | NC_025726 | Virosaurus | DNA library |
| 26144 | 8/25/2015 | 811_D | L4-r122 | TTMV | Anelloviridae | 26.39 | 2 | 22 | 772 | 2925 | KP343850 | Virosaurus | DNA library |
| 26145 | 8/25/2015 | 812_D | L5-r122 | TTMV | Anelloviridae | 97.2 | 169 | 5773 | 2987 | 3073 | KF545582 | Virosaurus | DNA library |
| 26147 | 8/25/2015 | 813_D | L6-r122 | TTMV | Anelloviridae | 49.85 | 2 | 34 | 1532 | 3073 | KF545582 | Virosaurus | DNA library |
| 26149 | 8/24/2015 | 648_D | L4-r91 | TTMV | Anelloviridae | 99.93 | 630 | 18807 | 2939 | 2941 | MN769124 | De novo | DNA library |
| 26159 | 8/26/2015 | 815_D | L8-r122 | TTMV | Anelloviridae | 64.64 | 2 | 62 | 1826 | 2825 | KU243129 | Virosaurus | DNA library |
| 26163 | 8/27/2015 | 817_D | L6-r124 | TTMV | Anelloviridae | 100 | 2418 | 86824 | 2913 | 2913 | NC_020498 | Virosaurus | DNA library |
| 26163 | 8/27/2015 | 817_R | L8-r740 | TTMV | Anelloviridae | 16.44 | 1 | 6 | 479 | 2913 | NC_020498 | Virosaurus | RNA library |
| 26165 | 8/26/2015 | 818_D | L7-r124 | TTMV | Anelloviridae | 32.71 | 4 | 52 | 966 | 2953 | NC_014082 | Virosaurus | DNA library |
| 26173 | 8/27/2015 | 819_D | L5-r122 | TTMV | Anelloviridae | 84.07 | 5 | 312 | 2449 | 2913 | EF538882 | Virosaurus | DNA library |
| 26174 | 8/27/2015 | 820_R | L1-r741 | TTMV | Anelloviridae | 13.45 | 4 | 27 | 396 | 2945 | MN770204 | De novo | RNA library |
| 26174 | 8/27/2015 | 820_D | L6-r122 | TTMV | Anelloviridae | 76.07 | 4 | 106 | 2216 | 2913 | EF538882 | Virosaurus | DNA library |
| 26176 | 8/28/2015 | 649_R | L8-r724 | TTMV | Anelloviridae | 22.1 | 13 | 101 | 657 | 2973 | MN771916 | De novo | RNA library |
| 26176 | 8/28/2015 | 649_D | L5-r91 | TTMV | Anelloviridae | 87.68 | 333 | 10811 | 2577 | 2939 | KX810064 | Virosaurus | DNA library |
| 26177 | 8/28/2015 | 821_D | L7-r122 | TTMV | Anelloviridae | 99.32 | 1763 | 53216 | 2926 | 2946 | MN770564 | De novo | DNA library |
| 26177 | 8/28/2015 | 821_R | L1-r741 | TTMV | Anelloviridae | 12.7 | 5 | 52 | 355 | 2795 | MN770115 | De novo | RNA library |
| 26178 | 8/28/2015 | 822_D | L8-r122 | TTMV | Anelloviridae | 26.54 | 4 | 63 | 734 | 2766 | NC_014086 | Virosaurus | DNA library |
| 26179 | 8/28/2015 | 823_D | L5-r124 | TTMV | Anelloviridae | 78.13 | 6 | 212 | 2276 | 2913 | EF538882 | Virosaurus | DNA library |
| 26181 | 8/28/2015 | 824_D | L6-r124 | TTMV | Anelloviridae | 99.35 | 1187 | 31934 | 2912 | 2931 | MN770733 | De novo | DNA library |
| 26182 | 8/28/2015 | 825_D | L7-r124 | TTMV | Anelloviridae | 99.97 | 1327 | 38733 | 2926 | 2927 | MN769348 | De novo | DNA library |
| 26186 | 8/31/2015 | 826_D | L4-r122 | TTMV | Anelloviridae | 13.84 | 5 | 25 | 412 | 2978 | KP343854 | Virosaurus | DNA library |
| 26193 | 8/31/2015 | 827_D | L6-r122 | TTMV | Anelloviridae | 82.63 | 88 | 2861 | 2360 | 2856 | NC_025727 | Virosaurus | DNA library |
| 26194 | 8/31/2015 | 828_R | L3-r741 | TTMV | Anelloviridae | 36.12 | 14 | 190 | 1016 | 2813 | MN772635 | De novo | RNA library |
| 26194 | 8/31/2015 | 828_D | L7-r122 | TTMV | Anelloviridae | 97.17 | 161 | 5656 | 2986 | 3073 | KF545582 | Virosaurus | DNA library |
| 26198 | 9/1/2015 | 829_R | L3-r741 | TTMV | Anelloviridae | 41.42 | 7 | 86 | 1209 | 2919 | MN769234 | De novo | RNA library |
| 26198 | 9/1/2015 | 829_D | L8-r122 | TTMV | Anelloviridae | 24.12 | 2 | 14 | 583 | 2417 | KT163914 | Virosaurus | DNA library |
| 26200 | 9/1/2015 | 830_D | L5-r124 | TTMV | Anelloviridae | 49.95 | 6 | 94 | 1411 | 2825 | KU243129 | Virosaurus | DNA library |
| 26203 | 9/1/2015 | 831_D | L6-r124 | TTMV | Anelloviridae | 95.07 | 35 | 1186 | 2774 | 2918 | KF545583 | Virosaurus | DNA library |
| 26204 | 9/1/2015 | 832_D | L7-r124 | TTMV | Anelloviridae | 25.45 | 2 | 14 | 615 | 2417 | KT163914 | Virosaurus | DNA library |
| 26208 | 9/2/2015 | 652_D | L8-r91 | TTMV | Anelloviridae | 49.5 | 5 | 99 | 1448 | 2925 | KP343850 | Virosaurus | DNA library |
| 26210 | 9/2/2015 | 833_R | L4-r741 | TTMV | Anelloviridae | 26.11 | 10 | 100 | 768 | 2942 | MN770127 | De novo | RNA library |
| 26210 | 9/2/2015 | 833_D | L4-r122 | TTMV | Anelloviridae | 55.17 | 40 | 1173 | 1605 | 2909 | NC_014089 | Virosaurus | DNA library |
| 26213 | 9/2/2015 | 834_D | L5-r122 | TTMV | Anelloviridae | 100 | 31120 | 930214 | 2925 | 2925 | MN770781 | De novo | DNA library |
| 26213 | 9/2/2015 | 834_R | L4-r741 | TTMV | Anelloviridae | 10.85 | 9 | 29 | 312 | 2876 | MN771366 | De novo | RNA library |
| 26214 | 9/2/2015 | 651_D | L7-r91 | TTMV | Anelloviridae | 54.33 | 8 | 285 | 1620 | 2982 | KP343847 | Virosaurus | DNA library |
| 26215 | 9/3/2015 | 835_D | L7-r122 | TTMV | Anelloviridae | 98.92 | 41 | 1259 | 2839 | 2870 | MN769870 | De novo | DNA library |
| 26219 | 9/3/2015 | 836_D | L8-r122 | TTMV | Anelloviridae | 99.6 | 23 | 1213 | 2977 | 2989 | KP343818 | Virosaurus | DNA library |
| 26221 | 9/4/2015 | 837_D | L5-r124 | TTMV | Anelloviridae | 21.84 | 2 | 18 | 604 | 2766 | NC_014086 | Virosaurus | DNA library |
| 26222 | 9/4/2015 | 838_D | L6-r124 | TTMV | Anelloviridae | 99.1 | 3294 | 93773 | 2958 | 2985 | MN770131 | De novo | DNA library |
| 26224 | 9/4/2015 | 839_D | L7-r124 | TTMV | Anelloviridae | 25.18 | 1 | 16 | 751 | 2982 | KP343847 | Virosaurus | DNA library |
| 26225 | 9/4/2015 | 840_D | L4-r122 | TTMV | Anelloviridae | 29.07 | 2 | 21 | 815 | 2804 | NC_030297 | Virosaurus | DNA library |
| 26229 | 9/4/2015 | 653_D | L1-r91 | TTMV | Anelloviridae | 34.74 | 2 | 40 | 972 | 2798 | EF538883 | Virosaurus | DNA library |
| 26234 | 9/7/2015 | 841_D | L5-r122 | TTMV | Anelloviridae | 98.91 | 759 | 23983 | 2913 | 2945 | MN770204 | De novo | DNA library |
| 26236 | 9/7/2015 | 842_R | L6-r741 | TTMV | Anelloviridae | 17.09 | 2 | 9 | 500 | 2925 | MN770781 | De novo | RNA library |
| 26236 | 9/7/2015 | 842_D | L6-r122 | TTMV | Anelloviridae | 47.25 | 12 | 326 | 1325 | 2804 | NC_030297 | Virosaurus | DNA library |
| 26237 | 9/7/2015 | 843_D | L8-r122 | TTMV | Anelloviridae | 28.74 | 1 | 21 | 804 | 2798 | EF538883 | Virosaurus | DNA library |
| 26241 | 9/7/2015 | 675_D | L2-r91 | TTMV | Anelloviridae | 41.66 | 13 | 163 | 1177 | 2825 | KU243129 | Virosaurus | DNA library |
| 26242 | 9/7/2015 | 844_D | L5-r124 | TTMV | Anelloviridae | 9.83 | 1 | 9 | 351 | 3571 | KF545584 | Virosaurus | DNA library |
| 26243 | 9/7/2015 | 869_D | L5-r132 | TTMV | Anelloviridae | 26.48 | 2 | 16 | 748 | 2825 | KU243129 | Virosaurus | DNA library |
| 26244 | 9/7/2015 | 845_D | L6-r124 | TTMV | Anelloviridae | 92.53 | 14 | 359 | 2699 | 2917 | MN769583 | De novo | DNA library |
| 26247 | 9/8/2015 | 846_D | L7-r124 | TTMV | Anelloviridae | 94.11 | 32 | 1009 | 2750 | 2922 | MN774049 | De novo | DNA library |
| 26250 | 9/8/2015 | 676_D | L3-r92 | TTMV | Anelloviridae | 80.82 | 10 | 292 | 2356 | 2915 | NC_025726 | Virosaurus | DNA library |
| 26251 | 9/8/2015 | 847_D | L4-r122 | TTMV | Anelloviridae | 92.66 | 9 | 321 | 2701 | 2915 | NC_025726 | Virosaurus | DNA library |
| 26253 | 9/8/2015 | 848_D | L5-r122 | TTMV | Anelloviridae | 88.74 | 18 | 993 | 3169 | 3571 | KF545584 | Virosaurus | DNA library |
| 26254 | 9/8/2015 | 849_D | L6-r122 | TTMV | Anelloviridae | 89.6 | 5 | 150 | 2610 | 2913 | EF538882 | Virosaurus | DNA library |
| 26256 | 9/8/2015 | 850_D | L7-r122 | TTMV | Anelloviridae | 98.86 | 834 | 26253 | 2936 | 2970 | MN769077 | De novo | DNA library |
| 26257 | 9/9/2015 | 851_D | L5-r124 | TTMV | Anelloviridae | 59.26 | 1 | 42 | 1821 | 3073 | KF545582 | Virosaurus | DNA library |
| 26259 | 9/9/2015 | 852_D | L6-r124 | TTMV | Anelloviridae | 99.69 | 370 | 11161 | 2891 | 2900 | MN770367 | De novo | DNA library |
| 26260 | 9/9/2015 | 853_D | L7-r124 | TTMV | Anelloviridae | 29.44 | 1 | 31 | 878 | 2982 | KP343847 | Virosaurus | DNA library |
| 26261 | 9/9/2015 | 654_D | L2-r91 | TTMV | Anelloviridae | 50.98 | 7 | 115 | 1485 | 2913 | EF538882 | Virosaurus | DNA library |
| 26262 | 9/9/2015 | 677_D | L4-r92 | TTMV | Anelloviridae | 21.27 | 5 | 39 | 600 | 2821 | KP343863 | Virosaurus | DNA library |
| 26263 | 9/9/2015 | 854_R | L1-r745 | TTMV | Anelloviridae | 13.48 | 5 | 23 | 395 | 2931 | MN770413 | De novo | RNA library |
| 26263 | 9/9/2015 | 854_D | L4-r122 | TTMV | Anelloviridae | 15.85 | 57 | 343 | 472 | 2978 | KP343854 | Virosaurus | DNA library |
| 26265 | 9/9/2015 | 855_D | L5-r122 | TTMV | Anelloviridae | 97.96 | 459 | 13954 | 2935 | 2996 | MN771181 | De novo | DNA library |
| 26267 | 9/9/2015 | 870_D | L6-r132 | TTMV | Anelloviridae | 98.1 | 36 | 1439 | 2883 | 2939 | KX810064 | Virosaurus | DNA library |
| 26273 | 9/10/2015 | 871_D | L8-r124 | TTMV | Anelloviridae | 99.54 | 219 | 6125 | 2831 | 2844 | MN771995 | De novo | DNA library |
| 26276 | 9/10/2015 | 656_D | L5-r91 | TTMV | Anelloviridae | 99.19 | 1380 | 39626 | 2955 | 2979 | MN769209 | De novo | DNA library |
| 26276 | 9/10/2015 | 656_R | L2-r725 | TTMV | Anelloviridae | 10.51 | 2 | 8 | 302 | 2873 | MN770933 | De novo | RNA library |
| 26278 | 9/10/2015 | 657_R | L2-r725 | TTMV | Anelloviridae | 19 | 10 | 56 | 547 | 2879 | MN770901 | De novo | RNA library |
| 26278 | 9/10/2015 | 657_D | L6-r91 | TTMV | Anelloviridae | 40.68 | 3 | 61 | 1213 | 2982 | KP343847 | Virosaurus | DNA library |
| 26279 | 9/10/2015 | 872_D | L1-r132 | TTMV | Anelloviridae | 100 | 1749 | 47737 | 2941 | 2941 | MN769457 | De novo | DNA library |
| 26280 | 9/11/2015 | 659_D | L8-r91 | TTMV | Anelloviridae | 88.47 | 6 | 198 | 2579 | 2915 | NC_025726 | Virosaurus | DNA library |
| 26281 | 9/11/2015 | 658_R | L2-r725 | TTMV | Anelloviridae | 15.77 | 10 | 52 | 456 | 2891 | MN769358 | De novo | RNA library |
| 26281 | 9/11/2015 | 658_D | L7-r91 | TTMV | Anelloviridae | 71.86 | 10 | 314 | 2030 | 2825 | KU243129 | Virosaurus | DNA library |
| 26282 | 9/11/2015 | 873_D | L2-r132 | TTMV | Anelloviridae | 66.44 | 10 | 244 | 1877 | 2825 | KU243129 | Virosaurus | DNA library |
| 26284 | 9/11/2015 | 874_R | L7-r745 | TTMV | Anelloviridae | 13.39 | 8 | 40 | 391 | 2921 | MN770342 | De novo | RNA library |
| 26284 | 9/11/2015 | 874_D | L3-r132 | TTMV | Anelloviridae | 96.78 | 30 | 1159 | 2974 | 3073 | KF545582 | Virosaurus | DNA library |
| 26287 | 9/11/2015 | 876_D | L5-r132 | TTMV | Anelloviridae | 21.81 | 1 | 20 | 638 | 2925 | KP343850 | Virosaurus | DNA library |
| 26293 | 9/14/2015 | 877_D | L6-r132 | TTMV | Anelloviridae | 99.97 | 188 | 5375 | 2898 | 2899 | MN771134 | De novo | DNA library |
| 26294 | 9/14/2015 | 878_R | L8-r745 | TTMV | Anelloviridae | 13.21 | 10 | 58 | 389 | 2944 | MN770282 | De novo | RNA library |
| 26294 | 9/14/2015 | 878_D | L8-r124 | TTMV | Anelloviridae | 42.23 | 1 | 29 | 1073 | 2541 | KT163888 | Virosaurus | DNA library |
| 26295 | 9/14/2015 | 879_R | L8-r745 | TTMV | Anelloviridae | 17.01 | 11 | 73 | 496 | 2916 | MN770056 | De novo | RNA library |
| 26295 | 9/14/2015 | 879_D | L8-r127 | TTMV | Anelloviridae | 44.92 | 4 | 88 | 1314 | 2925 | KP343850 | Virosaurus | DNA library |
| 26299 | 9/14/2015 | 880_D | L2-r132 | TTMV | Anelloviridae | 93.25 | 150 | 4746 | 2816 | 3020 | MN770161 | De novo | DNA library |
| 26301 | 9/15/2015 | 881_D | L3-r132 | TTMV | Anelloviridae | 75.6 | 10 | 250 | 2159 | 2856 | NC_025727 | Virosaurus | DNA library |
| 26309 | 9/15/2015 | 882_D | L4-r132 | TTMV | Anelloviridae | 98.1 | 304 | 9055 | 2884 | 2940 | MN770581 | De novo | DNA library |
| 26311 | 9/15/2015 | 883_D | L5-r132 | TTMV | Anelloviridae | 30.2 | 2 | 126 | 862 | 2854 | KF764701 | Virosaurus | DNA library |
| 26312 | 9/15/2015 | 884_D | L6-r132 | TTMV | Anelloviridae | 98.36 | 292 | 8515 | 2874 | 2922 | MN770213 | De novo | DNA library |
| 26317 | 9/16/2015 | 678_D | L5-r92 | TTMV | Anelloviridae | 96.84 | 640 | 19161 | 2819 | 2911 | NC_014068 | Virosaurus | DNA library |
| 26318 | 9/16/2015 | 885_D | L8-r124 | TTMV | Anelloviridae | 57.62 | 4 | 69 | 1479 | 2567 | KF545580 | Virosaurus | DNA library |
| 26323 | 9/16/2015 | 886_D | L8-r127 | TTMV | Anelloviridae | 31.28 | 1 | 62 | 915 | 2925 | KP343850 | Virosaurus | DNA library |
| 26330 | 9/17/2015 | 887_D | L1-r132 | TTMV | Anelloviridae | 99.68 | 311 | 8426 | 2817 | 2826 | MN769843 | De novo | DNA library |
| 26331 | 9/17/2015 | 888_D | L3-r132 | TTMV | Anelloviridae | 30.22 | 2 | 26 | 836 | 2766 | NC_014086 | Virosaurus | DNA library |
| 26332 | 9/17/2015 | 889_D | L4-r132 | TTMV | Anelloviridae | 98.4 | 109 | 3509 | 2884 | 2931 | MN770834 | De novo | DNA library |
| 26333 | 9/17/2015 | 670_D | L4-r91 | TTMV | Anelloviridae | 100 | 2351 | 71129 | 2945 | 2945 | MN770204 | De novo | DNA library |
| 26333 | 9/17/2015 | 670_R | L5-r725 | TTMV | Anelloviridae | 16.57 | 4 | 20 | 479 | 2891 | MN770153 | De novo | RNA library |
| 26334 | 9/17/2015 | 890_D | L5-r132 | TTMV | Anelloviridae | 25.5 | 2 | 27 | 753 | 2953 | NC_014082 | Virosaurus | DNA library |
| 26336 | 9/17/2015 | 891_D | L6-r132 | TTMV | Anelloviridae | 91.79 | 6 | 156 | 2671 | 2910 | MN769480 | De novo | DNA library |
| 26340 | 9/17/2015 | 892_D | L8-r124 | TTMV | Anelloviridae | 47.31 | 3 | 63 | 1378 | 2913 | EF538882 | Virosaurus | DNA library |
| 26342 | 9/17/2015 | 893_D | L8-r127 | TTMV | Anelloviridae | 23.92 | 4 | 31 | 687 | 2872 | EF538881 | Virosaurus | DNA library |
| 26343 | 9/17/2015 | 894_D | L1-r132 | TTMV | Anelloviridae | 91.64 | 6 | 724 | 2739 | 2989 | KP343818 | Virosaurus | DNA library |
| 26345 | 9/17/2015 | 679_D | L6-r92 | TTMV | Anelloviridae | 68.25 | 4 | 95 | 1752 | 2567 | KF545580 | Virosaurus | DNA library |
| 26346 | 9/17/2015 | 895_D | L2-r132 | TTMV | Anelloviridae | 26.21 | 2 | 309 | 774 | 2953 | NC_014082 | Virosaurus | DNA library |
| 26351 | 9/21/2015 | 896_D | L4-r132 | TTMV | Anelloviridae | 99.4 | 52 | 1762 | 2814 | 2831 | KX810063 | Virosaurus | DNA library |
| 26353 | 9/21/2015 | 897_D | L5-r132 | TTMV | Anelloviridae | 50.81 | 30 | 669 | 1478 | 2909 | NC_014089 | Virosaurus | DNA library |
| 26354 | 9/21/2015 | 898_D | L6-r132 | TTMV | Anelloviridae | 36.92 | 1 | 33 | 1080 | 2925 | KP343850 | Virosaurus | DNA library |
| 26360 | 9/22/2015 | 899_R | L5-r746 | TTMV | Anelloviridae | 21.43 | 7 | 59 | 637 | 2972 | MN771177 | De novo | RNA library |
| 26360 | 9/22/2015 | 899_D | L8-r124 | TTMV | Anelloviridae | 48.39 | 4 | 81 | 1443 | 2982 | KP343847 | Virosaurus | DNA library |
| 26361 | 9/22/2015 | 900_D | L8-r127 | TTMV | Anelloviridae | 37.19 | 2 | 19 | 1054 | 2834 | KP343848 | Virosaurus | DNA library |
| 26368 | 9/22/2015 | 671_D | L6-r91 | TTMV | Anelloviridae | 30.84 | 4 | 48 | 902 | 2925 | KP343850 | Virosaurus | DNA library |
| 26399 | 9/29/2015 | 680_D | L1-r93 | TTMV | Anelloviridae | 12.59 | 30 | 383 | 375 | 2978 | KP343854 | Virosaurus | DNA library |
| 26424 | 10/23/2015 | 1_D | L008-r54 | TTMV | Anelloviridae | 99.97 | 2620 | 75856 | 2919 | 2920 | MN770323 | De novo | DNA library |
| 26425 | 10/23/2015 | 9_D | L002-r44 | TTMV | Anelloviridae | 93.78 | 18 | 562 | 2730 | 2911 | NC_014068 | Virosaurus | DNA library |
| 26432 | 10/26/2015 | 25_D | L8-r96 | TTMV | Anelloviridae | 99.21 | 179 | 5146 | 2877 | 2900 | MN769136 | De novo | DNA library |
| 26436 | 10/27/2015 | 44_D | L001-r44 | TTMV | Anelloviridae | 100 | 597 | 18395 | 2989 | 2989 | KP343818 | Virosaurus | DNA library |
| 26439 | 10/28/2015 | 2_D | L002-r44 | TTMV | Anelloviridae | 49.46 | 3 | 48 | 1475 | 2982 | KP343847 | Virosaurus | DNA library |
| 26446 | 10/29/2015 | 26_R | L8-r669 | TTMV | Anelloviridae | 19.51 | 5 | 31 | 571 | 2927 | MN769348 | De novo | RNA library |
| 26446 | 10/29/2015 | 26_D | L005-r44 | TTMV | Anelloviridae | 96.96 | 21 | 589 | 2838 | 2927 | MN769348 | De novo | DNA library |
| 26447 | 10/29/2015 | 18_D | L004-r44 | TTMV | Anelloviridae | 100 | 228 | 6489 | 2827 | 2827 | KF545585 | Virosaurus | DNA library |
| 26455 | 11/2/2015 | 27_D | L006-r44 | TTMV | Anelloviridae | 12.4 | 14 | 69 | 354 | 2854 | KF764701 | Virosaurus | DNA library |
| 26459 | 11/3/2015 | 4_D | L004-r44 | TTMV | Anelloviridae | 74.88 | 17 | 856 | 2233 | 2982 | KP343847 | Virosaurus | DNA library |
| 26462 | 11/3/2015 | 12_D | L008-r54 | TTMV | Anelloviridae | 99.49 | 48 | 1722 | 2951 | 2966 | MN770750 | De novo | DNA library |
| 26466 | 11/6/2015 | 786_R | L8-r737 | TTMV | Anelloviridae | 14.66 | 8 | 36 | 438 | 2988 | MN770490 | De novo | RNA library |
| 26466 | 11/6/2015 | 786_D | L7-r113 | TTMV | Anelloviridae | 21.98 | 2 | 33 | 608 | 2766 | NC_014086 | Virosaurus | DNA library |
| 26472 | 11/6/2015 | 33_D | L005-r44 | TTMV | Anelloviridae | 99.93 | 784 | 27491 | 2927 | 2929 | MN770345 | De novo | DNA library |
| 26479 | 11/10/2015 | 40_D | L004-r44 | TTMV | Anelloviridae | 97.23 | 120 | 3936 | 2988 | 3073 | KF545582 | Virosaurus | DNA library |
| 26482 | 11/10/2015 | 672_R | L6-r725 | TTMV | Anelloviridae | 14.52 | 6 | 32 | 426 | 2934 | MN769818 | De novo | RNA library |
| 26482 | 11/10/2015 | 672_D | L7-r91 | TTMV | Anelloviridae | 100 | 122 | 4199 | 2989 | 2989 | KP343818 | Virosaurus | DNA library |
| 26483 | 11/10/2015 | 21_D | L008-r54 | TTMV | Anelloviridae | 99.83 | 157 | 6178 | 2984 | 2989 | KP343818 | Virosaurus | DNA library |
| 26485 | 11/11/2015 | 787_D | L8-r113 | TTMV | Anelloviridae | 99.33 | 8 | 288 | 2812 | 2831 | KX810063 | Virosaurus | DNA library |
| 26487 | 11/11/2015 | 6_D | L006-r44 | TTMV | Anelloviridae | 100 | 76 | 2398 | 2977 | 2977 | KP343834 | Virosaurus | DNA library |
| 26488 | 11/11/2015 | 15_D | L008-r44 | TTMV | Anelloviridae | 77.49 | 17 | 454 | 2196 | 2834 | KP343848 | Virosaurus | DNA library |
| 26492 | 11/12/2015 | 5_D | L004-r55 | TTMV | Anelloviridae | 69.84 | 10 | 332 | 2107 | 3017 | KP343864 | Virosaurus | DNA library |
| 26493 | 11/12/2015 | 14_R | L5-r669 | TTMV | Anelloviridae | 14.4 | 2 | 8 | 412 | 2861 | MN774811 | De novo | RNA library |
| 26493 | 11/12/2015 | 14_D | L004-r55 | TTMV | Anelloviridae | 99.93 | 10 | 400 | 2829 | 2831 | KX810063 | Virosaurus | DNA library |
| 26494 | 11/12/2015 | 22_R | L5-r669 | TTMV | Anelloviridae | 14.69 | 1 | 7 | 428 | 2913 | MN769409 | De novo | RNA library |
| 26494 | 11/12/2015 | 22_D | L008-r44 | TTMV | Anelloviridae | 16.74 | 1 | 9 | 488 | 2916 | JX134046 | Virosaurus | DNA library |
| 26495 | 11/12/2015 | 28_D | L002-r55 | TTMV | Anelloviridae | 31.56 | 2 | 38 | 885 | 2804 | NC_030297 | Virosaurus | DNA library |
| 26498 | 11/13/2015 | 34_D | L006-r44 | TTMV | Anelloviridae | 30.06 | 2 | 33 | 841 | 2798 | EF538883 | Virosaurus | DNA library |
| 26502 | 11/16/2015 | 673_R | L6-r725 | TTMV | Anelloviridae | 24.45 | 7 | 74 | 721 | 2949 | MN769828 | De novo | RNA library |
| 26502 | 11/16/2015 | 673_D | L8-r91 | TTMV | Anelloviridae | 13.52 | 3 | 80 | 481 | 3559 | KP343833 | Virosaurus | DNA library |
| 26503 | 11/16/2015 | 41_D | L006-r44 | TTMV | Anelloviridae | 33.04 | 2 | 36 | 949 | 2872 | EF538881 | Virosaurus | DNA library |
| 26506 | 11/17/2015 | 7_D | L007-r44 | TTMV | Anelloviridae | 52.31 | 9 | 179 | 1560 | 2982 | KP343847 | Virosaurus | DNA library |
| 26507 | 11/17/2015 | 30_D | L001-r44 | TTMV | Anelloviridae | 96.94 | 1338 | 40460 | 2948 | 3041 | MN771643 | De novo | DNA library |
| 26512 | 11/19/2015 | 8_D | L005-r55 | TTMV | Anelloviridae | 32.24 | 2 | 37 | 939 | 2913 | EF538882 | Virosaurus | DNA library |
| 26513 | 11/19/2015 | 17_D | L003-r44 | TTMV | Anelloviridae | 78.03 | 244 | 27520 | 2270 | 2909 | NC_014089 | Virosaurus | DNA library |
| 26518 | 11/23/2015 | 790_D | L4-r113 | TTMV | Anelloviridae | 82.87 | 3 | 201 | 2477 | 2989 | KP343818 | Virosaurus | DNA library |
| 26520 | 11/23/2015 | 36_D | L001-r55 | TTMV | Anelloviridae | 95.05 | 97 | 3145 | 2786 | 2931 | MN771395 | De novo | DNA library |
| 26522 | 11/26/2015 | 661_D | L2-r91 | TTMV | Anelloviridae | 26.98 | 1 | 15 | 755 | 2798 | EF538883 | Virosaurus | DNA library |
| 26524 | 11/23/2015 | 43_D | L008-r54 | TTMV | Anelloviridae | 99.9 | 1266 | 36358 | 2890 | 2893 | MN771421 | De novo | DNA library |
| 26531 | 11/24/2015 | 77_D | L003-r46 | TTMV | Anelloviridae | 99.31 | 1437 | 56606 | 2893 | 2913 | NC_020498 | Virosaurus | DNA library |
| 26533 | 11/24/2015 | 663_D | L5-r91 | TTMV | Anelloviridae | 91.9 | 3 | 201 | 2747 | 2989 | KP343818 | Virosaurus | DNA library |
| 26534 | 11/24/2015 | 69_D | L008-r54 | TTMV | Anelloviridae | 100 | 205 | 6393 | 2827 | 2827 | KF545585 | Virosaurus | DNA library |
| 26536 | 11/24/2015 | 52_D | L007-r46 | TTMV | Anelloviridae | 17.86 | 2 | 15 | 494 | 2766 | NC_014086 | Virosaurus | DNA library |
| 26540 | 11/26/2015 | 87_D | L007-r46 | TTMV | Anelloviridae | 88.21 | 39 | 1308 | 2492 | 2825 | KU243129 | Virosaurus | DNA library |
| 26546 | 11/30/2015 | 70_R | L003-r675 | TTMV | Anelloviridae | 11.48 | 9 | 36 | 342 | 2979 | MN769209 | De novo | RNA library |
| 26546 | 11/30/2015 | 70_D | L004-r46 | TTMV | Anelloviridae | 66.19 | 17 | 325 | 1699 | 2567 | KF545580 | Virosaurus | DNA library |
| 26551 | 12/2/2015 | 88_D | L008-r46 | TTMV | Anelloviridae | 79.93 | 23 | 610 | 2258 | 2825 | KU243129 | Virosaurus | DNA library |
| 26557 | 12/3/2015 | 54_D | L002-r46 | TTMV | Anelloviridae | 98.97 | 276 | 8830 | 2886 | 2916 | MN769089 | De novo | DNA library |
| 26558 | 12/4/2015 | 74_D | L008-r46 | TTMV | Anelloviridae | 100 | 23 | 910 | 2913 | 2913 | NC_020498 | Virosaurus | DNA library |
| 26559 | 12/7/2015 | 84_D | L003-r46 | TTMV | Anelloviridae | 96.36 | 35 | 1129 | 2805 | 2911 | NC_014068 | Virosaurus | DNA library |
| 26562 | 12/10/2015 | 55_D | L003-r46 | TTMV | Anelloviridae | 99.16 | 180 | 5276 | 2952 | 2977 | MN769977 | De novo | DNA library |
| 26562 | 12/10/2015 | 55_R | L007-r674 | TTMV | Anelloviridae | 17.46 | 10 | 55 | 519 | 2972 | MN771177 | De novo | RNA library |
| 21004 | 12/12/2014 | 91_R | L001-r676 | TTV | Anelloviridae | 11.03 | 2 | 8 | 388 | 3519 | MN768497 | De novo | RNA library |
| 21004 | 12/12/2014 | 91_D | L001-r49 | TTV | Anelloviridae | 78.69 | 6 | 203 | 2946 | 3744 | AJ620216 | Virosaurus | DNA library |
| 21006 | 1/16/2015 | 92_D | L002-r49 | TTV | Anelloviridae | 98.38 | 28382 | 1014898 | 3759 | 3821 | MN767226 | De novo | DNA library |
| 21007 | 1/19/2015 | 794_D | L8-r113 | TTV | Anelloviridae | 98.51 | 46 | 1645 | 3642 | 3697 | MN765797 | De novo | DNA library |
| 21014 | 1/23/2015 | 93_D | L003-r49 | TTV | Anelloviridae | 86.3 | 34 | 1009 | 2665 | 3088 | AY449524 | Virosaurus | DNA library |
| 21017 | 1/23/2015 | 766_R | L3-r737 | TTV | Anelloviridae | 15.34 | 2 | 9 | 547 | 3566 | MN765977 | De novo | RNA library |
| 21017 | 1/23/2015 | 766_D | L6-r111 | TTV | Anelloviridae | 83.5 | 41 | 1654 | 3224 | 3861 | FR751490 | Virosaurus | DNA library |
| 21018 | 1/26/2015 | 94_D | L004-r49 | TTV | Anelloviridae | 78.43 | 33 | 1237 | 2923 | 3727 | AB064596 | Virosaurus | DNA library |
| 21019 | 1/26/2015 | 95_D | L005-r49 | TTV | Anelloviridae | 82.22 | 35 | 1277 | 3103 | 3774 | AM711976 | Virosaurus | DNA library |
| 21021 | 1/26/2015 | 96_R | L002-r676 | TTV | Anelloviridae | 11.5 | 6 | 32 | 427 | 3713 | MN768503 | De novo | RNA library |
| 21021 | 1/26/2015 | 96_D | L006-r49 | TTV | Anelloviridae | 94.2 | 429 | 15593 | 3511 | 3727 | AB064596 | Virosaurus | DNA library |
| 21026 | 1/28/2015 | 97_D | L007-r49 | TTV | Anelloviridae | 98.82 | 233 | 8777 | 3588 | 3631 | MN765827 | De novo | DNA library |
| 21027 | 1/28/2015 | 98_D | L008-r49 | TTV | Anelloviridae | 77.29 | 10 | 371 | 2984 | 3861 | FR751490 | Virosaurus | DNA library |
| 21028 | 1/28/2015 | 796_D | L7-r111 | TTV | Anelloviridae | 83.21 | 10 | 369 | 3196 | 3841 | AF122918 | Virosaurus | DNA library |
| 21031 | 1/29/2015 | 586_D | L7-r88 | TTV | Anelloviridae | 86.3 | 13 | 549 | 3219 | 3730 | NC_014074 | Virosaurus | DNA library |
| 21039 | 2/2/2015 | 99_D | L002-r49 | TTV | Anelloviridae | 64.3 | 28 | 783 | 2223 | 3457 | KF545586 | Virosaurus | DNA library |
| 21041 | 2/2/2015 | 100_D | L003-r55 | TTV | Anelloviridae | 60.6 | 3 | 65 | 1955 | 3226 | KT163880 | Virosaurus | DNA library |
| 21042 | 2/3/2015 | 101_D | L002-r55 | TTV | Anelloviridae | 38.15 | 1 | 25 | 1473 | 3861 | FR751490 | Virosaurus | DNA library |
| 21044 | 2/3/2015 | 102_D | L005-r49 | TTV | Anelloviridae | 84.65 | 300 | 11629 | 2950 | 3485 | KP343838 | Virosaurus | DNA library |
| 21048 | 2/4/2015 | 103_D | L001-r55 | TTV | Anelloviridae | 90.85 | 118 | 4039 | 3347 | 3684 | MN768092 | De novo | DNA library |
| 21053 | 2/6/2015 | 104_D | L007-r49 | TTV | Anelloviridae | 99.19 | 35860 | 1319375 | 3669 | 3699 | MN765674 | De novo | DNA library |
| 21060 | 2/13/2015 | 106_D | L001-r49 | TTV | Anelloviridae | 71.58 | 48 | 1959 | 2632 | 3677 | AB038621 | Virosaurus | DNA library |
| 21074 | 2/19/2015 | 107_D | L003-r49 | TTV | Anelloviridae | 90.42 | 44 | 1485 | 3229 | 3571 | AB064597 | Virosaurus | DNA library |
| 21077 | 2/19/2015 | 108_D | L004-r49 | TTV | Anelloviridae | 85.86 | 82 | 2991 | 3151 | 3670 | AB064599 | Virosaurus | DNA library |
| 21080 | 2/20/2015 | 109_D | L005-r49 | TTV | Anelloviridae | 77.75 | 24 | 1174 | 2953 | 3798 | FR751491 | Virosaurus | DNA library |
| 21082 | 2/20/2015 | 110_D | L006-r49 | TTV | Anelloviridae | 84.43 | 6 | 223 | 3015 | 3571 | AB064597 | Virosaurus | DNA library |
| 21083 | 2/20/2015 | 111_D | L007-r49 | TTV | Anelloviridae | 96.6 | 94 | 3947 | 3519 | 3643 | MN768370 | De novo | DNA library |
| 21085 | 2/23/2015 | 112_D | L008-r49 | TTV | Anelloviridae | 96.2 | 20 | 771 | 3489 | 3627 | AB064601 | Virosaurus | DNA library |
| 21090 | 2/24/2015 | 113_D | L001-r49 | TTV | Anelloviridae | 83.84 | 23 | 811 | 3231 | 3854 | AF122914 | Virosaurus | DNA library |
| 21092 | 2/27/2015 | 114_D | L002-r49 | TTV | Anelloviridae | 91.02 | 16 | 611 | 3364 | 3696 | MN767302 | De novo | DNA library |
| 21099 | 2/26/2015 | 116_D | L005-r49 | TTV | Anelloviridae | 94.68 | 66 | 2219 | 3381 | 3571 | AB064597 | Virosaurus | DNA library |
| 21102 | 2/27/2015 | 118_D | L007-r49 | TTV | Anelloviridae | 99.03 | 226 | 8699 | 3586 | 3621 | MN767511 | De novo | DNA library |
| 21104 | 2/27/2015 | 119_D | L008-r49 | TTV | Anelloviridae | 96.28 | 20 | 785 | 3492 | 3627 | AB064601 | Virosaurus | DNA library |
| 21105 | 3/2/2015 | 120_D | L001-r49 | TTV | Anelloviridae | 96.36 | 383 | 14891 | 3601 | 3737 | NC_014080 | Virosaurus | DNA library |
| 21109 | 3/11/2015 | 767_D | L7-r111 | TTV | Anelloviridae | 98.3 | 673 | 24767 | 3591 | 3653 | MN766439 | De novo | DNA library |
| 21110 | 3/3/2015 | 121_R | L008-r676 | TTV | Anelloviridae | 10.33 | 9 | 34 | 389 | 3765 | MN768049 | De novo | RNA library |
| 21110 | 3/3/2015 | 121_D | L002-r49 | TTV | Anelloviridae | 68.19 | 12 | 350 | 2604 | 3819 | NC_014091 | Virosaurus | DNA library |
| 21115 | 3/4/2015 | 122_R | L008-r676 | TTV | Anelloviridae | 14.89 | 3 | 24 | 539 | 3619 | MN767968 | De novo | RNA library |
| 21115 | 3/4/2015 | 122_D | L003-r49 | TTV | Anelloviridae | 60.35 | 578 | 24945 | 1985 | 3289 | AF345522 | Virosaurus | DNA library |
| 21116 | 3/4/2015 | 123_D | L005-r49 | TTV | Anelloviridae | 92.13 | 143 | 4712 | 3290 | 3571 | AB064597 | Virosaurus | DNA library |
| 21122 | 3/5/2015 | 124_R | L001-r677 | TTV | Anelloviridae | 68.77 | 25 | 766 | 2554 | 3714 | MN768153 | De novo | RNA library |
| 21122 | 3/5/2015 | 124_D | L006-r49 | TTV | Anelloviridae | 93.1 | 110 | 5094 | 3307 | 3552 | KP343839 | Virosaurus | DNA library |
| 21124 | 3/16/2015 | 768_D | L8-r111 | TTV | Anelloviridae | 80.27 | 3 | 113 | 2946 | 3670 | AB064599 | Virosaurus | DNA library |
| 21129 | 3/10/2015 | 125_D | L007-r49 | TTV | Anelloviridae | 94.97 | 587 | 25987 | 3643 | 3836 | MN768007 | De novo | DNA library |
| 21138 | 3/11/2015 | 769_D | L4-r113 | TTV | Anelloviridae | 93.64 | 47 | 1634 | 3344 | 3571 | AB064597 | Virosaurus | DNA library |
| 21148 | 3/13/2015 | 126_D | L008-r49 | TTV | Anelloviridae | 70.43 | 5 | 164 | 2625 | 3727 | AB064596 | Virosaurus | DNA library |
| 21154 | 3/16/2015 | 770_D | L5-r113 | TTV | Anelloviridae | 90.72 | 98 | 3438 | 3342 | 3684 | KP343845 | Virosaurus | DNA library |
| 21158 | 3/17/2015 | 127_D | L001-r49 | TTV | Anelloviridae | 82.59 | 39 | 1463 | 3130 | 3790 | HM449773 | Virosaurus | DNA library |
| 21159 | 3/17/2015 | 128_D | L002-r49 | TTV | Anelloviridae | 91.87 | 1004 | 34819 | 3426 | 3729 | FR751466 | Virosaurus | DNA library |
| 21169 | 3/19/2015 | 129_D | L003-r49 | TTV | Anelloviridae | 67.18 | 12 | 370 | 2505 | 3729 | MN765936 | De novo | DNA library |
| 21175 | 3/16/2015 | 130_D | L004-r49 | TTV | Anelloviridae | 92.71 | 85 | 2865 | 3156 | 3404 | KF545578 | Virosaurus | DNA library |
| 21176 | 3/16/2015 | 131_D | L006-r49 | TTV | Anelloviridae | 82.37 | 49 | 1700 | 3144 | 3817 | FR751499 | Virosaurus | DNA library |
| 21177 | 3/17/2015 | 132_D | L007-r49 | TTV | Anelloviridae | 98.86 | 1546 | 61963 | 3732 | 3775 | DQ361268 | Virosaurus | DNA library |
| 21177 | 3/17/2015 | 132_R | L003-r677 | TTV | Anelloviridae | 26.46 | 1 | 13 | 999 | 3775 | DQ361268 | Virosaurus | RNA library |
| 21180 | 3/13/2015 | 133_D | L008-r49 | TTV | Anelloviridae | 81.77 | 89 | 3622 | 3157 | 3861 | FR751490 | Virosaurus | DNA library |
| 21183 | 3/13/2015 | 134_D | L001-r49 | TTV | Anelloviridae | 85.76 | 9 | 315 | 2709 | 3159 | KT163904 | Virosaurus | DNA library |
| 21184 | 3/13/2015 | 135_D | L002-r49 | TTV | Anelloviridae | 98.09 | 455 | 16175 | 3540 | 3609 | MN768396 | De novo | DNA library |
| 21191 | 3/20/2015 | 50_D | L005-r46 | TTV | Anelloviridae | 83.89 | 221 | 7368 | 3202 | 3817 | FR751499 | Virosaurus | DNA library |
| 21195 | 12/11/2015 | 60_D | L008-r46 | TTV | Anelloviridae | 81.23 | 13 | 411 | 2981 | 3670 | AB064599 | Virosaurus | DNA library |
| 21200 | 3/20/2015 | 61_D | L001-r46 | TTV | Anelloviridae | 89.86 | 28 | 910 | 3209 | 3571 | AB064597 | Virosaurus | DNA library |
| 21201 | 3/23/2015 | 67_D | L008-r46 | TTV | Anelloviridae | 69.4 | 5 | 150 | 2649 | 3817 | FR751499 | Virosaurus | DNA library |
| 21220 | 3/30/2015 | 213_R | L005-r690 | TTV | Anelloviridae | 13.85 | 4 | 16 | 498 | 3596 | MN767828 | De novo | RNA library |
| 21220 | 3/30/2015 | 213_D | L002-r57 | TTV | Anelloviridae | 62 | 408 | 19679 | 2039 | 3289 | AF345522 | Virosaurus | DNA library |
| 21234 | 4/8/2015 | 214_D | L003-r57 | TTV | Anelloviridae | 94.77 | 205 | 6899 | 3226 | 3404 | KF545578 | Virosaurus | DNA library |
| 21234 | 4/8/2015 | 214_R | L005-r690 | TTV | Anelloviridae | 9.27 | 1 | 4 | 341 | 3677 | AB038621 | Virosaurus | RNA library |
| 21247 | 4/10/2015 | 215_D | L008-r67 | TTV | Anelloviridae | 90.13 | 72 | 2393 | 3359 | 3727 | AB064596 | Virosaurus | DNA library |
| 21253 | 4/13/2015 | 216_D | L005-r57 | TTV | Anelloviridae | 92.62 | 121 | 5713 | 2988 | 3226 | KT163880 | Virosaurus | DNA library |
[truncated: 106,444 more chars]
