# Supplementary figures and images for: Blood virosphere in febrile Tanzanian children
Source: Emerg Microbes Infect. 2021 May 28;10(1):982–93. doi: 10.1080/22221751.2021.1925161 (PMC8171259; doi:10.1080/22221751.2021.1925161)

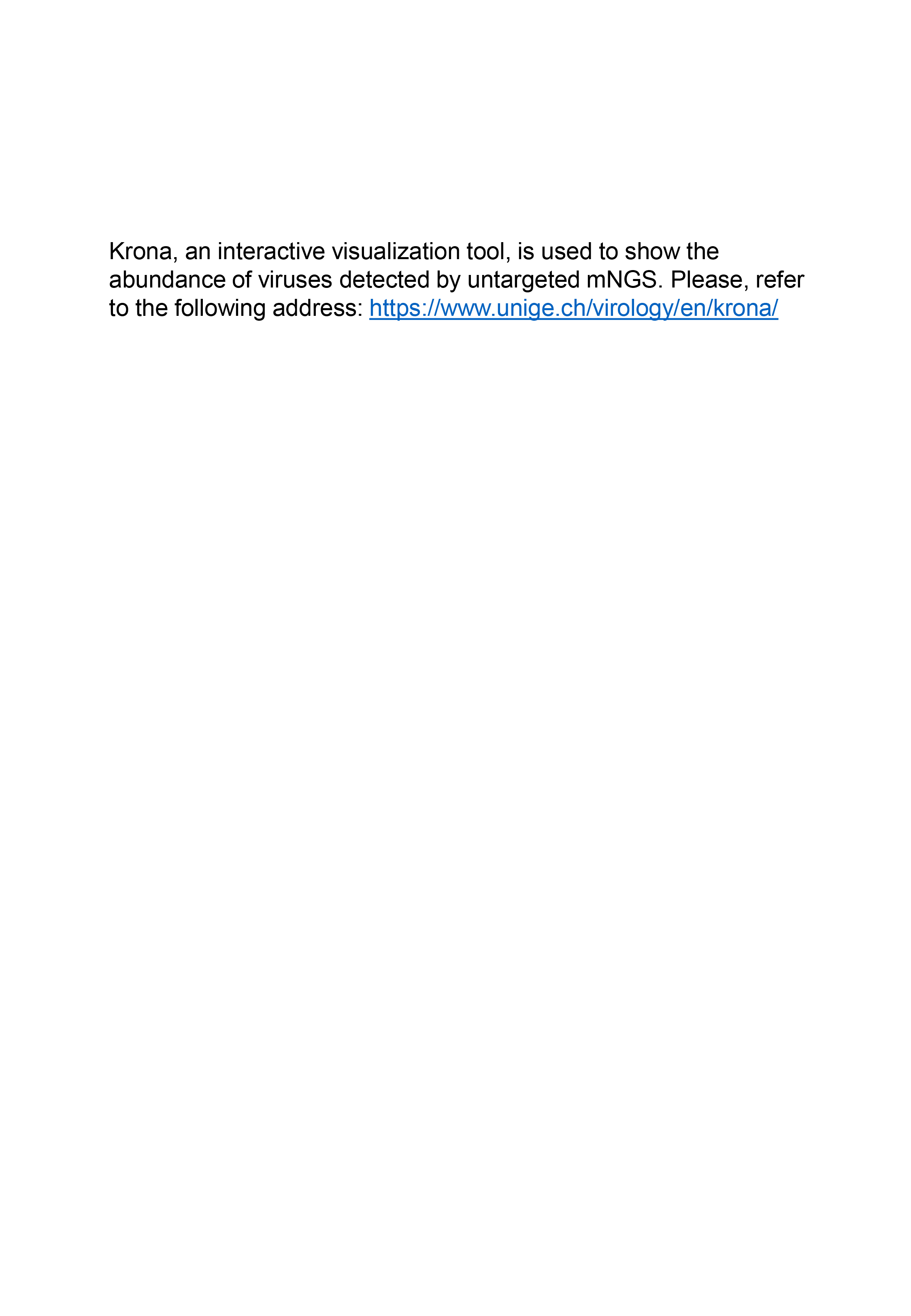

Supplement: Supplemental Material [file TEMI_A_1925161_SM1971.zip › Suppl files/Supplementary Figure S1.jpg]

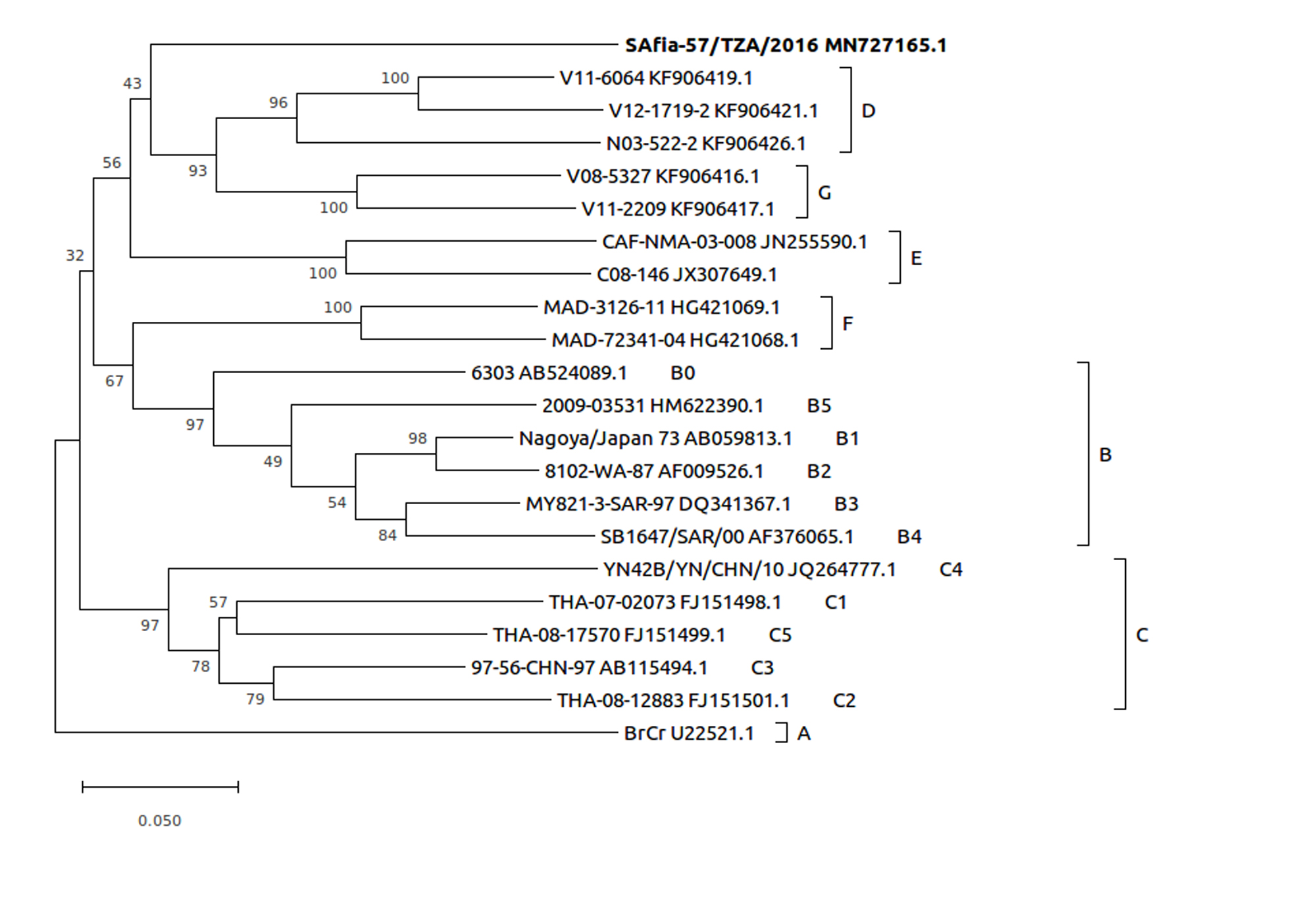

Supplement: Supplemental Material [file TEMI_A_1925161_SM1971.zip › Suppl files/Supplementary Figure S2.jpg]

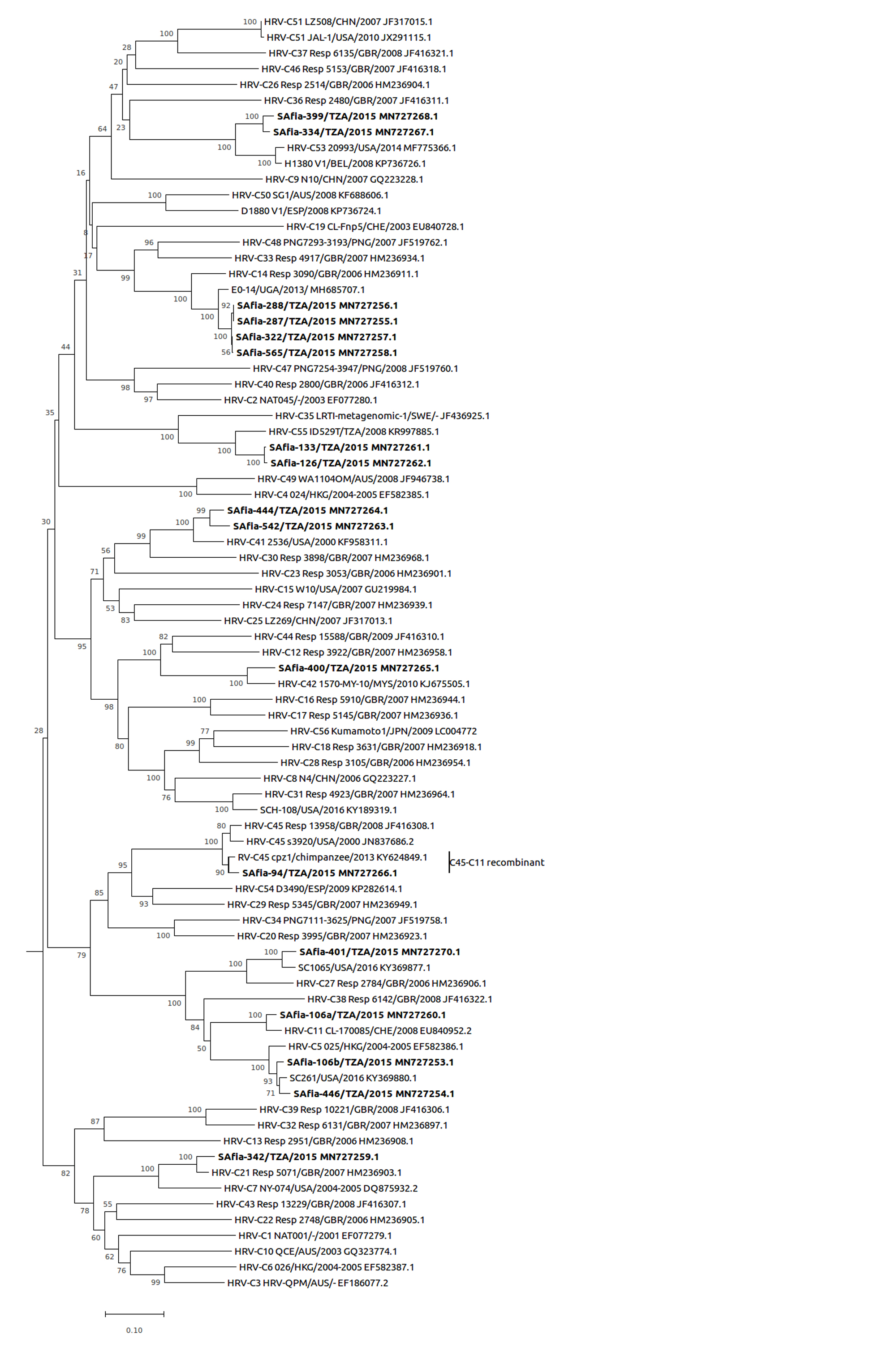

Supplement: Supplemental Material [file TEMI_A_1925161_SM1971.zip › Suppl files/Supplementary Figure S3.jpg]

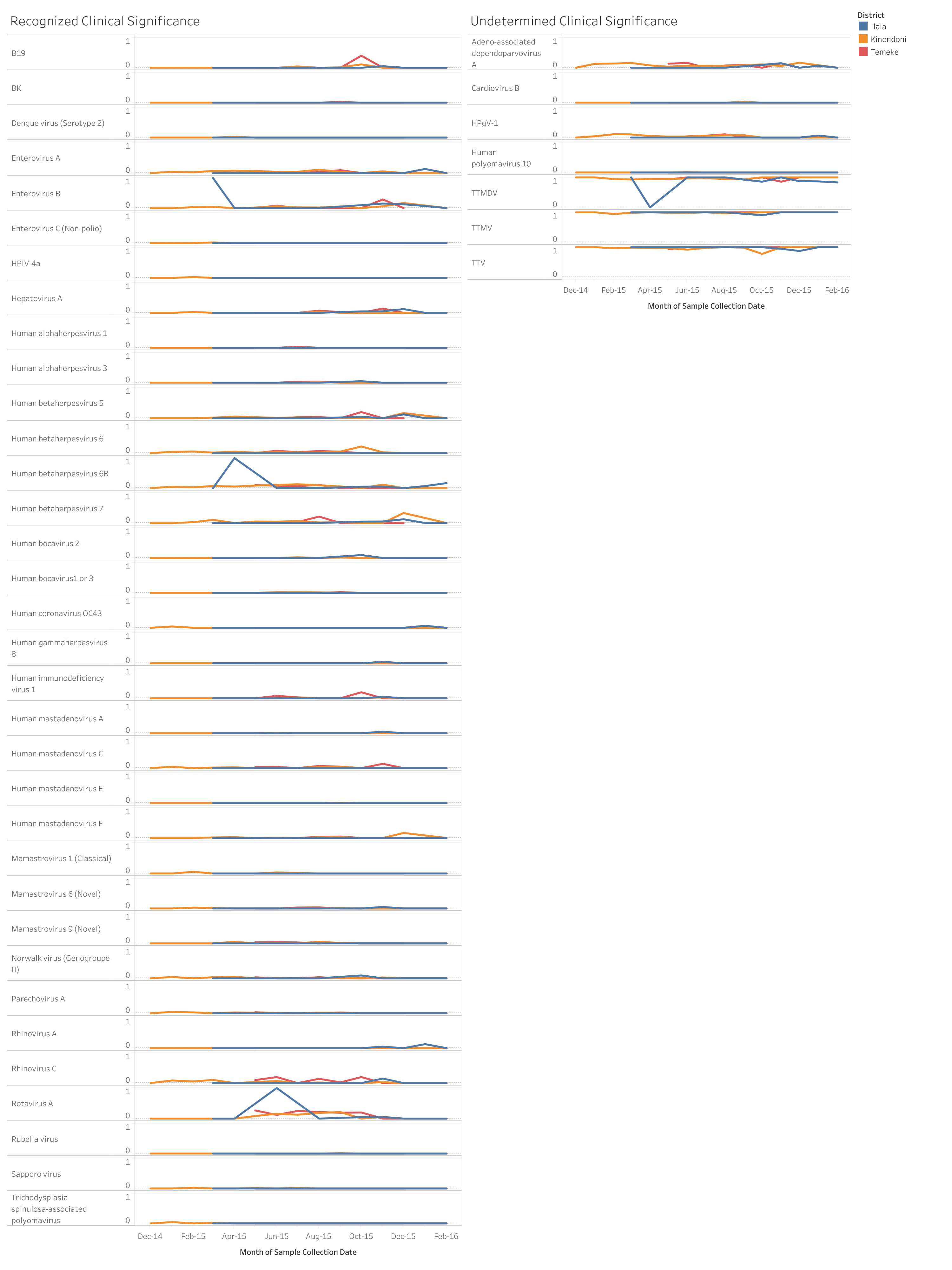

Supplement: Supplemental Material [file TEMI_A_1925161_SM1971.zip › Suppl files/Supplementary Figure S4.jpg]
